# Usual On-therapy Ranges of Drug Concentrations in Patients with Atrial Fibrillation Treated with Direct Oral Anticoagulants: A Systematic Review and Meta-analysis

**DOI:** 10.1055/a-2446-1348

**Published:** 2024-11-21

**Authors:** Tim A.C. de Vries, Imaad U. Mallick, Vinai C. Bhagirath, John W. Eikelboom, Calvin Gomes, Qilong Yi, Sean McGrath, Jack Hirsh, Noel C. Chan

**Affiliations:** 1 Department of Clinical and Experimental Cardiology and Cardiothoracic Surgery, Amsterdam University Medical Centers, University of Amsterdam, Amsterdam, The Netherlands; 2 Amsterdam Cardiovascular Sciences, Heart Failure and Arrhythmias, Amsterdam, The Netherlands; 3 Department of Cardiology, Amsterdam University Medical Centers, Vrije Universiteit, Amsterdam, The Netherlands; 4 Department of Cardiology, Rijnstate Hospital, Arnhem, The Netherlands; 5 School of Medicine, St. George's University, Grenada, West Indies; 6 Division of Hematology and Thromboembolism, Department of Medicine, Population Health Research Institute, Hamilton, Ontario, Canada; 7 Division of Hematology and Thromboembolism, Department of Medicine, McMaster University, Hamilton, Ontario, Canada; 8 Thrombosis and Atherosclerosis Research Institute, Hamilton, Ontario, Canada; 9 School of Medicine, Poznan University of Medical Sciences, Poznan, Poland; 10 School of Epidemiology and Public Health, University of Ottawa, Ottawa, Ontario, Canada; 11 Department of Biostatistics, Harvard T.H. Chan School of Public Health, Boston, Massachusetts, United States

**Keywords:** direct oral anticoagulant, blood coagulation tests, drug monitoring, biological variation, population, factor Xa inhibitors, anticoagulants, hemorrhage

## Abstract

**Background:**

Although most patients with atrial fibrillation (AF) receiving a direct oral anticoagulant (DOAC) do not require drug concentration measurements, there are situations where such information could be useful. Existing guidance documents provide usual on-therapy ranges for drug concentrations, but these have important limitations.

**Methods:**

This is a systematic review and meta-analysis of studies reporting trough and peak levels of DOAC regimens approved for stroke prevention in AF. We used random effects models and the quantile estimation method to estimate the median and a usual on-therapy range (10 ^th^ and 90 ^th^ percentiles).

**Results:**

Of 4,822 unique publications, 53 studies met eligibility (29,266 trough and 12,103 peak levels). Usual on-therapy ranges for trough levels were 38 to 155 and 58 to 206 ng/mL for apixaban 2.5 and 5 mg twice daily; 35 to 138 and 33 to 151 ng/mL for dabigatran 110 and 150 mg twice daily; 8 to 54 and 13 to 66 ng/mL for edoxaban 30 and 60 mg daily; and 16 to 74 and 19 to 72 ng/mL for rivaroxaban 15 and 20 mg daily. The corresponding range for peak levels were 96 to 251 and 132 to 343; 65 to 223 and 76 to 285; 57 to 219 and 127 to 407; 131 to 384, and 169 to 313 ng/mL, respectively.

**Conclusion:**

This systematic review and meta-analysis provides updated and more representative usual on-therapy ranges of DOAC levels in patients with AF.

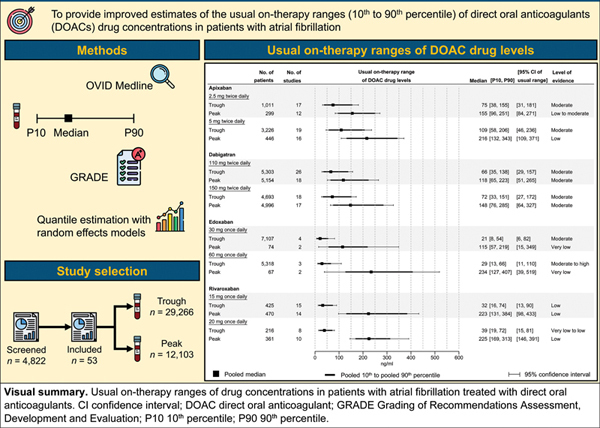

## Introduction

Anticoagulant therapy has been revolutionized by the replacement of drugs requiring dose adjustments based on results of coagulation assays (heparin and vitamin K antagonists), with anticoagulants that do not require laboratory monitoring. 1 The shift, which began in the 1990s with the introduction of low molecular weight heparin, improved the convenience of anticoagulant therapy, and reduced cost by minimizing the need for in-hospital treatment. 1 The introduction of direct oral anticoagulants (DOACs) has further simplified anticoagulation therapy. All four available DOACs (i.e., apixaban, dabigatran, edoxaban, and rivaroxaban), when used in fixed doses without laboratory monitoring of anticoagulant activity, are at least as effective and safe as warfarin for the prevention of ischemic strokes in patients with nonvalvular atrial fibrillation (AF). 1 2 3 4 As a result, DOACs are used in fixed doses, either in a high dose or low dose, depending on the patient's characteristics. 1 2 3

Although most patients treated with fixed doses of DOACs do not need laboratory monitoring, there are clinical circumstances in which measuring a drug level might be desirable. 1 5 6 In the absence of therapeutic ranges, less reliable metrics such as usual on-therapy ranges (10 ^th^ to 90 ^th^ or 5 ^th^ to 95 ^th^ percentile ranges) of trough and peak drug concentrations have been proposed to guide physicians if they consider it necessary to measure drug levels. 3 5 The previously published usual on-therapy ranges were derived from a few studies of limited applicability to patients with AF (e.g., derived from healthy subjects, patients with other indications for treatment with a DOAC), or reduced validity due to reliance on pharmacokinetic modelling. 3 5 Since the initial publication of the guidance documents and product monographs, over 50 clinical studies have been published examining drug levels in patients taking DOACs for stroke prevention in AF. 6 7 8 9 10 11 12 13 14 15 16 17 18 19 20 21 22 23 24 25 26 27 28 29 30 31 32 33 34 35 36 37 38 39 40 41 42 43 44 45 46 47 48 49 50 51 52 53 54 55 56 57 58

In this systematic review and meta-analysis, we provide updated estimates for the 10 ^th^ to 90 ^th^ percentile ranges (i.e., middle 80% of drug levels) for trough and peak concentrations of the four approved DOACs when used in patients with AF using assays commonly used in clinical practice. This update provides clinicians with more representative estimates of the usual on-therapy ranges of DOACs than previously reported, to help guide their decision-making.

## Methods

### Protocol

This report adheres to the Preferred Reporting Items for Systematic Reviews and Meta-Analyses (PRISMA) recommendations (PRISMA checklist provided in Supplementary Table S1 of Supporting Information File 1 , available in the online version). 59 Our protocol is provided in Supporting Information File 2 (available in the online version).

### Data Sources and Searches

We electronically searched MEDLINE via Ovid for articles reporting on plasma concentrations of DOACs in patients with AF treated with DOACs to prevent ischemic strokes published between the inception of the database and January 2023. The full search strategy is presented in Supplementary Table S2 of Supporting Information File 1 (available in the online version). This search was supplemented by manual review of the reference list of articles identified from the initial search.

### Study Selection

Studies were eligible for inclusion if they reported either or both trough and peak drug levels in ng/mL (or in units that allowed for direct conversion to ng/mL) for DOACs (apixaban, dabigatran, edoxaban, rivaroxaban) approved for stroke prevention in AF. If there was overlap in study populations among primary studies, we avoided double counting by using the data of interest from the more comprehensive publication.

Eligible studies included randomized controlled trials and observational studies that collected blood samples and reported cross-sectional data on drug levels. We excluded case reports and case series, pharmacokinetic simulations, studies that did not separately report DOAC drug levels of patients with AF from those who were treated for other indications (e.g., treatment or prevention of venous thromboembolism), as well as reports that did not differentiate levels by DOAC type and administered dose. We further excluded studies written in a non-English language and those with total sample size of <10 patients. After deduplication, all hits were screened for eligibility by two reviewers (I.U.M. and C.G.). In the instance of disagreement, the final decision was determined by a third reviewer (T.A.C.dV. or N.C.C.).

### Data Extraction and Estimation of Nonreported Percentiles of Drug Concentrations

Two reviewers (I.U.M. and C.G.) independently extracted data using a standardized case report form that included the following variables: the number of patients, the DOAC dose and frequency, measures of distribution (mean, median, standard deviation, percentiles, interquartile range) of trough and peak DOAC drug concentrations for each DOAC regimen, and the laboratory method used to determine levels. The percentiles of interest were the 50 ^th^ (i.e., the median), 10 ^th^ , and 90 ^th^ percentiles. If not reported in the individual studies, these percentiles were calculated directly from the original dataset if the study was published by the authors of the current review, 6 15 42 or in most situations, were estimated using simulations based on the published measures of central tendency and dispersion. More details regarding these simulation strategies are described in Supporting Information File 2 (available in online version only).

### Outcome Measures

The outcomes of interest were the 50 ^th^ , 10 ^th^ , and 90 ^th^ percentile drug concentration for each DOAC regimen at trough and at peak. We defined the usual on-therapy range as the range spanning between the pooled 10 ^th^ and 90 ^th^ percentiles of drug level. We also report the lower bound of the 95% confidence interval (CI) of the pooled 10 ^th^ percentile value and the upper bound of the 95% CI of the pooled 90 ^th^ percentile value to provide a more conservative estimate of the usual on-therapy range.

### Statistical Analyses

We estimated the pooled 50 ^th^ , 10 ^th^ and 90 ^th^ percentile of DOAC drug concentration in ng/mL for each respective dose using the quantile estimation (QE) method. 60 61 This method estimates the variance of the study-specific medians from the reported summary statistics and then performs an inverse–variance weighted meta-analysis of medians. 60 61 We applied the same estimation strategy to estimate the pooled 10 ^th^ and the pooled 90 ^th^ percentile of trough and peak levels for each DOAC dosing regimen.

In all models, we prioritized summary statistics reported in the primary studies over simulated ones. Because the distributions of DOAC drug levels are right-skewed, we did not use the reported mean and standard deviation of some studies because the QE method would assume that data were normally distributed. Instead, for these studies, we used the simulated median, 25 ^th^ and 75 ^th^ percentile values. Given the method of data collection and the likely heterogeneity between studies due to differences in populations and methods of measurement, we used the random effects model (REM) in all analyses. 62 More details on the performed statistical analyses are provided in Supporting Information File 2 (available in online version only).

### Sensitivity Analyses

To assess the robustness of our findings on 10 ^th^ and 90 ^th^ percentile values, we performed two sets of prespecified sensitivity analyses for DOAC dosing regimen and one post hoc defined analysis. As defined in our protocol, we only considered these analyses whenever at least 10 studies provided data on the percentile of interest ( Supporting Information File 2 , available in online version only). 63

In these analyses, we redetermined the on-therapy ranges (i.e., 10 ^th^ and 90 ^th^ percentiles) selecting only the studies (1) at low risk of bias and low concern of inapplicability to our review question, (2) for which the 10 ^th^ and 90 ^th^ percentile values were provided in the report, and (3) that determined levels with liquid chromatography–mass spectrometry/mass spectrometry.

We used mixed-effects models to assess for significant differences between the subsets of studies using the dichotomized variable as a potential modifier (i.e., low risk of bias and low concern of inapplicability vs. other studies, reported vs. simulated percentile of interest, or liquid chromatography–mass spectrometry/mass spectrometry vs. chromogenic assays). We kept Tau constant if there were five or fewer studies in either subset 63 and defined a significant difference between the subsets as a Wald test producing a two-tailed *p* -value < 0.05.

### Quality Assessments

Assessing the quality of evidence of systematic reviews reporting on usual on-therapy ranges is less well established. We adopted the Grading of Recommendations Assessment, Development and Evaluation (GRADE) system to rate the quality of evidence for each outcome (i.e., 50 ^th^ , 10 ^th^ , and 90 ^th^ percentile) of each DOAC dosing regimen. 64 To rate each study on their risk of bias and our concern of indirectness, we answered several prespecified signaling questions. These signaling questions sought to assess the risk of bias due to patient selection (domain 1 of QUADAS-2 tool) and due to method of drug concentration measurement (domain 2 through 4 of QUADAS-2 tool), as well as our concern of indirectness due to patient selection ( Supplementary Tables S1 – S4 in Supporting Information File 3 , available in the online version). 65 The considerations and criteria for performing the quality assessments and rating of each domain (i.e., risk of bias, indirectness, inconsistency, and precision), including our rationale to not perform a formal publication bias assessment, are presented in Supplementary Table S5 of Supporting Information File 3 (available in the online version). The interpretation of the level of evidence ratings are described in Supplementary Table S6 of Supporting Information File 3 (available in the online version).

## Results

### Study Selection

**Fig. 1.**
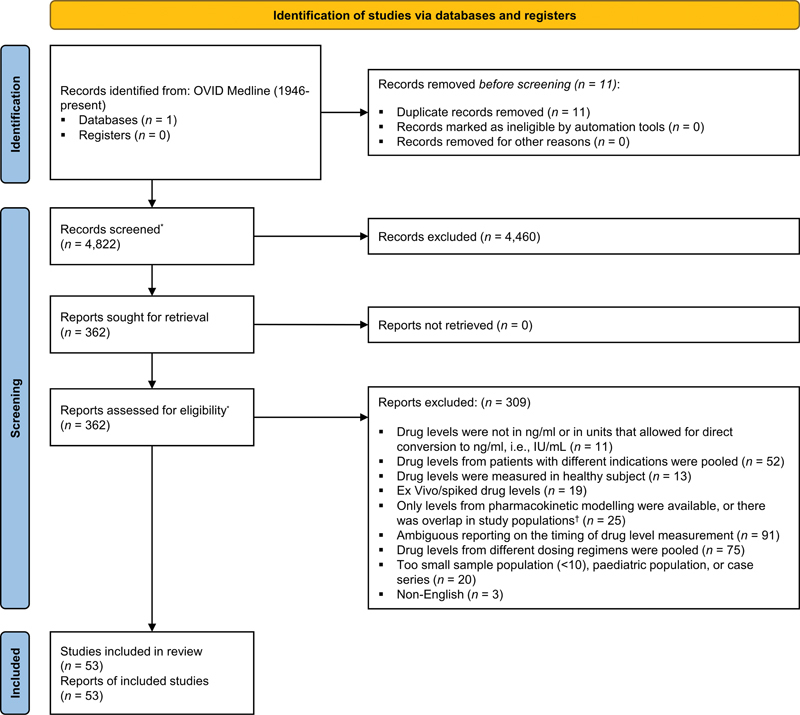
Flow of study selection. This flow diagram illustrates the flow of study selection. * After deduplication, all hits were screened for eligibility by two reviewers (I.U.M. and C.G.). In the instance of disagreement, the final decision was determined by a third reviewer (T.A.C.dV. or N.C.C.); ^†^ If there was overlap in study populations among primary studies, we avoided double counting by using the data of interest from the more comprehensive publication.

We identified 4,833 publications of which 11 were duplicates. We screened the title and abstract of the 4,822 unique hits, of which 4,460 were excluded because they were irrelevant to our review question. After reading the full text, we excluded another 309 from the remaining 362 hits and were left with a total of 53 studies ( Fig. 1 ). 6 7 8 9 10 11 12 13 14 15 16 17 18 19 20 21 22 23 24 25 26 27 28 29 30 31 32 33 34 35 36 37 38 39 40 41 42 43 44 45 46 47 48 49 50 51 52 53 54 55 56 57 58 These included studies collectively reported on a total of 29,266 trough levels and 12,103 of peak levels. The characteristics of the included studies are presented in Supplementary Table S1 of Supporting Information File 4 (available in the online version).

### Usual On-Therapy Ranges

In this section we present the usual on-therapy ranges of trough and peak levels of DOAC doses approved in most regulatory regions. A more detailed description of the results of each meta-analytic model is provided in Supporting Information File 4 (available in the online version), which includes the number of studies and patients available, measures of heterogeneity, as well as the ranges of the less commonly used dosing regimens.

**Fig. 2:**
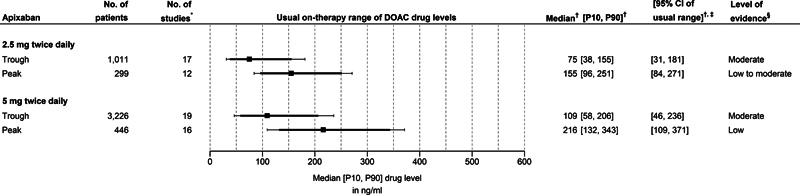
Median (and 10 ^th^ –90 ^th^ percentiles) of drug levels for apixaban. The squares represent the pooled median values, the solid bold lines the pooled estimates for the 10 ^th^ to 90 ^th^ percentile range, and the whiskers the interval from the lower bound of the 95% CI of the pooled 10 ^th^ percentile (left side) to the upper bound of the 95% CI of the pooled 90 ^th^ percentile value (right side). P10 10 ^th^ percentile; P90 90 ^th^ percentile; CI, confidence interval; DOAC, direct oral anticoagulant; No., number. *Some studies reported on multiple subgroups of patients. Each subgroup was then considered a unique study; ^†^ Estimated with random effects models using the (modified) QE method; 60 61 ^‡^ The interval from the lower bound of the 95% CI of the pooled 10 ^th^ percentile to the upper bound of the 95% CI of the pooled 90 ^th^ percentile value; ^§^ Level of evidence following the GRADE-framework and determined for each outcome of interest (i.e., median, 10 ^th^ percentile, and 90 ^th^ percentiles). 64

Apixaban: Fig. 2 illustrates the usual on-therapy ranges of both trough and peak concentrations for the two approved dosing regimens of apixaban.

For the 2.5 mg twice daily dose of apixaban, the pooled estimate for the usual on-therapy range (10 ^th^ to 90 ^th^ percentile range) of trough levels is 38 to 155 ng/mL, with lower and upper bounds of the 95% CIs for 10 ^th^ and 90 ^th^ percentiles, respectively of 31 and 181 ng/mL. The corresponding range for peak levels is 96 to 251 ng/mL with lower and upper bounds of the 95% CIs for 10 ^th^ and 90 ^th^ percentiles of 84 and 271 ng/mL, respectively.

For the 5 mg twice daily dose of apixaban, the pooled estimate for the usual on-therapy range (10 ^th^ to 90 ^th^ percentile range) of trough levels is 58 to 206 ng/mL, with lower and upper bounds of the 95% CI for 10 ^th^ and 90 ^th^ percentiles, respectively, of 46 and 236 ng/mL. The corresponding range for peak levels is 132 to 343 ng/mL, with lower and upper bounds of the 95% CIs for 10 ^th^ and 90 ^th^ percentiles, respectively, of 109 and 371 ng/mL.

**Fig. 3:**
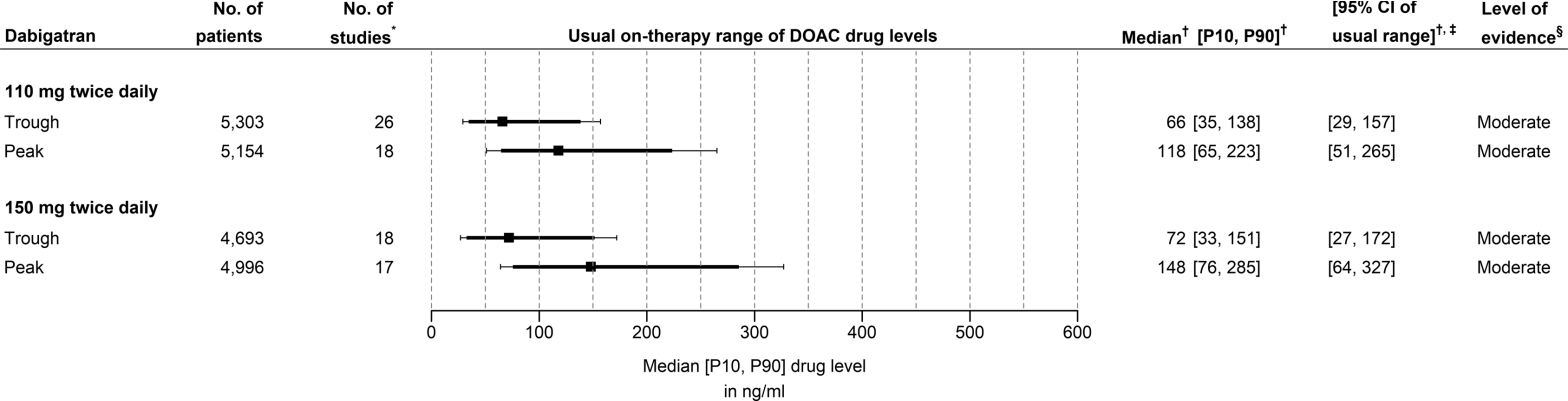
Median (and 10 ^th^ –90 ^th^ percentiles) of drug levels for dabigatran. The usual on-therapy ranges of the 75 mg twice daily dose are presented in Supplementary Fig. S2 of Supporting Information File 4 [available in the online version]. The squares represent the pooled median values, the solid bold lines the pooled estimates for the 10 ^th^ to 90 ^th^ percentile range, and the whiskers the interval from the lower bound of the 95% CI of the pooled 10 ^th^ percentile (left side) to the upper bound of the 95% CI of the pooled 90 ^th^ percentile value (right side). P10 10 ^th^ percentile; P90 90 ^th^ percentile; CI confidence interval; DOAC direct oral anticoagulant; No. number. *Some studies reported on multiple subgroups of patients. Each subgroup was then considered a unique study; ^†^ Estimated with random effects models using the (modified) QE-method; 60 61 ^‡^ The interval from the lower bound of the 95% CI of the pooled 10 ^th^ percentile to the upper bound of the 95% CI of the pooled 90 ^th^ percentile value; ^§^ Level of evidence following the GRADE-framework and determined for each outcome of interest (i.e., median, 10 ^th^ percentile, and 90 ^th^ percentile). 64

Dabigatran: Fig. 3 illustrates the usual on-therapy ranges of both trough and peak concentrations for the two commonly approved dosing regimens of dabigatran (i.e., 110 mg twice daily and 150 mg twice daily). The usual on-therapy ranges of all three available dabigatran dosing regimens, which includes the 75 mg twice daily dose, are presented in Fig S2 of Supporting Information File 4 (available in the online version).

For the 110 mg twice daily dose of dabigatran, the pooled estimate for the usual on-therapy range (10 ^th^ to 90 ^th^ percentile range) of trough levels is 35 to 138 ng/mL, with lower and upper bounds of the 95% CIs for 10 ^th^ and 90 ^th^ percentiles, respectively, of 29 and 157 ng/mL. The corresponding range for peak levels is 65 to 223 ng/mL, with lower and upper bounds of the 95% CIs for 10 ^th^ and 90 ^th^ percentiles, respectively, of 51 and 265 ng/mL.

For the 150 mg twice daily dose of dabigatran, the pooled estimate for the usual on-therapy range (10 ^th^ to 90 ^th^ percentile range) of trough levels is 33 to 151 ng/mL, with lower and upper bounds of the 95% CIs for 10 ^th^ and 90 ^th^ percentiles, respectively, of 27 and 172 ng/mL. The corresponding range for peak levels is 76 to 285 ng/mL with lower and upper bounds of the 95% CIs for 10 ^th^ and 90 ^th^ percentiles, respectively, of 64 and 327 ng/mL.

**Fig. 4:**
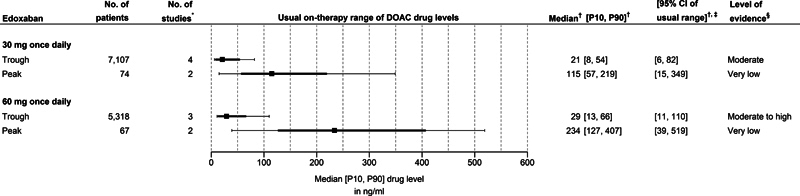
Median (and 10 ^th^ –90 ^th^ percentiles) of drug levels for edoxaban. The usual on-therapy ranges of the 15 mg once daily dose are presented in Supplementary Fig. S3 of Supporting Information File 4 [available in the online version]. The squares represent the pooled median values, the solid bold lines the pooled estimates for the 10 ^th^ to 90 ^th^ percentile range, and the whiskers the interval from the lower bound of the 95% CI of the pooled 10 ^th^ percentile (left side) to the upper bound of the 95% CI of the pooled 90 ^th^ percentile value (right side). *P10* 10 ^th^ percentile; *P90* 90 ^th^ percentile; *CI* confidence interval; *DOAC* direct oral anticoagulant; *No.* number. * Some studies reported on multiple subgroups of patients. Each subgroup was then considered a unique study; ^†^ Estimated with random effects models using the (modified) QE-method; 60 61 ^‡^ The interval from the lower bound of the 95% CI of the pooled 10 ^th^ percentile to the upper bound of the 95% CI of the pooled 90 ^th^ percentile value; ^§^ Level of evidence following the GRADE-framework and determined for each outcome of interest (i.e., median, 10 ^th^ percentile, and 90 ^th^ percentile). 64

Edoxaban: Fig. 4 illustrates the usual on-therapy ranges of both trough and peak concentrations for the two commonly approved dosing regimens of edoxaban (i.e., 30 mg once daily and 60 mg once daily). The usual on-therapy ranges of all three available edoxaban dosing regimens, which includes the 15 mg once daily dose, are presented in Fig S3 of Supporting Information File 4 (available in the online version).

For the 30 mg once daily dose of edoxaban, the pooled estimate for the usual on-therapy range (10 ^th^ to 90 ^th^ percentile range) of trough levels is 8 to 54 ng/mL with lower and upper bounds of the 95% CIs for 10 ^th^ and 90 ^th^ percentiles, respectively, of 6 and 82 ng/mL. The corresponding range for peak levels is 57 to 219 ng/mL, with lower and upper bounds of the 95% CIs for 10 ^th^ and 90 ^th^ percentiles, respectively, of 15 and 349 ng/mL.

For the 60 mg once daily dose of edoxaban, the pooled estimate for the usual on-therapy range (10 ^th^ to 90 ^th^ percentile range) of trough levels is 13 to 66 ng/mL, with lower and upper bounds of the 95% CIs for 10 ^th^ and 90 ^th^ percentiles, respectively, of 11 and 110 ng/mL. The corresponding range for peak levels is 127 to 407 ng/mL, with lower and upper bounds of the 95% CIs for 10 ^th^ and 90 ^th^ percentiles, respectively, of 39 and 519 ng/mL.

**Fig. 5.**
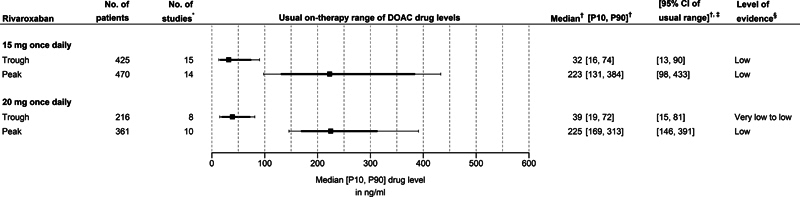
Median (and 10 ^th^ – 90 ^th^ percentiles) of drug levels for rivaroxaban. The usual on-therapy ranges of the 10 mg once daily dose are presented in Supplementary Fig. S4 of Supporting Information File 4 (available in the online version). The squares represent the pooled median values, the solid bold lines the pooled estimates for the 10 ^th^ to 90 ^th^ percentile range, and the whiskers the interval from the lower bound of the 95% CI of the pooled 10 ^th^ percentile (left side) to the upper bound of the 95% CI of the pooled 90 ^th^ percentile value (right side). *P10* 10 ^th^ percentile; *P90* 90 ^th^ percentile; *CI* confidence interval; *DOAC* direct oral anticoagulant; *No.* number. * Some studies reported on multiple subgroups of patients. Each subgroup was then considered a unique study; ^†^ Estimated with random effects models using the (modified) QE-method; 60 61 ^‡^ The interval from the lower bound of the 95% CI of the pooled 10 ^th^ percentile to the upper bound of the 95% CI of the pooled 90 ^th^ percentile value; ^§^ Level of evidence following the GRADE-framework and determined for each outcome of interest (i.e., median, 10 ^th^ percentile, and 90 ^th^ percentile). 64

Rivaroxaban: Fig. 5 illustrates the usual on-therapy ranges of both trough and peak concentrations for the two commonly approved dosing regimens of rivaroxaban (i.e., 15 mg once daily and 20 mg once daily). The usual on-therapy ranges of all three available rivaroxaban dosing regimens, which includes the 10 mg once daily dose, are presented in Fig. S4 of Supporting Information File 4 (available in the online version).

For the 15 mg once daily dose of rivaroxaban, the pooled estimate for the usual on-therapy range (10 ^th^ to 90 ^th^ percentile range) of trough levels is 16 to 74 ng/mL with lower and upper bounds of the 95% CIs for 10 ^th^ and 90 ^th^ percentiles, respectively, of 13 and 90 ng/mL. The corresponding range for peak levels is 131 to 384 ng/mL, with lower and upper bounds of the 95% CIs for 10 ^th^ and 90 ^th^ percentiles, respectively, of 98 and 433 ng/mL.

For the 20 mg once daily dose of rivaroxaban, the pooled estimate for the usual on-therapy range (10 ^th^ to 90 ^th^ percentile range) of trough levels is 19 to 72 ng/mL with lower and upper bounds of the 95% CIs for 10 ^th^ and 90 ^th^ percentiles, respectively, of 15 and 81 ng/mL. The corresponding range for peak levels is 169 to 313 ng/mL with lower and upper bounds of the 95% CIs for 10 ^th^ and 90 ^th^ percentiles, respectively, of 146 and 391 ng/mL.

The level of evidence for all outcomes (i.e., median, 10 ^th^ percentile, 90 ^th^ percentile) of each DOAC dosing regimen ranged from high to very low as reported in Supplementary Table S12 of Supporting Information File 3 (available in the online version). The level of evidence was based on our assessments of the risk of bias, indirectness, inconsistency, and imprecision assessments (summarized in Supplementary Tables S7 – S11 of Supporting Information File 3 , available in the online version).

### Sensitivity Analyses

Performing the sensitivity analysis was impossible (because only a single study was available or all studies fell into the same category) or noninformative because fewer than 10 studies were available in 30 (68%) of analyses on risk of bias and concern of inapplicability, 22 (50%) on method of data extraction, and 22 (50%) on the laboratory methods used to determine levels.

For the DOAC regimens for which these analyses were feasible, rather than including all eligible studies, selecting only the studies at low risk of bias and concern of inapplicability studies ( Supplementary Table S1 [available in the online version] and Supplementary Figs. S1 – S4 of Supporting Information File 5 [available in the online version]), those studies that provided the 10 ^th^ and 90 ^th^ percentile values in their report ( Supplementary Table S2 and Figs. S5–S8 of Supporting Information File 5 [available in the online version]), or studies that used liquid chromatography–mass spectrometry/mass spectrometry to determine levels ( Supplementary Table S3 and Supplementary Figs. S9–S12 of Supporting Information File 5 [available in the online version]) did not result in consistently higher or lower 10 ^th^ or 90 ^th^ percentile values.

## Discussion

In this systematic review and meta-analysis, we provide clinicians with the best-available information on the usual on-therapy range of drug levels in patients taking DOACs for stroke prevention in nonvalvular AF. We pooled data from 53 studies reporting on a total of 29,266 trough levels and 12,103 peak levels to generate estimates of the usual on-therapy range for trough or peak levels for approved dosing regimens of four DOACs. 6 7 8 9 10 11 12 13 14 15 16 17 18 19 20 21 22 23 24 25 26 27 28 29 30 31 32 33 34 35 36 37 38 39 40 41 42 43 44 45 46 47 48 49 50 51 52 53 54 55 56 57 58 Our estimates overlap those reported in guidelines and monographs 3 5 but are more representative because these were derived from a more comprehensive dataset that included only patients with AF and excluded pharmacokinetic modelling studies. Despite the methodological limitations of our study and of those of the included studies, our usual on-therapy ranges are an improvement on those currently reported. Our estimates better reflect trough and peak levels of all approved dosing regimens in AF, because we took indication and timing of sample collection into consideration.

These usual on-therapy ranges for trough and peak levels of DOACs can help clinicians who decide to measure levels to manage their patients who experience unexpected bleeding as well as those who were ineligible for inclusion or were underrepresented in the randomized trials. About 50% of patients treated in clinics who are prescribed the lower dose of a DOAC do not meet the recommended dose reduction criteria (i.e., off-label dose reduction). 66 67 Clinicians who use off-label dosing mainly select the lower dose and use this dose in patients who they suspect are at high risk of bleeding or drug overexposure. For example, patients with severe comorbidities (e.g., advanced kidney or liver disease), those with extreme clinical characteristics (e.g., extremes of body weight or age), and patients who are taking drugs that are known to interact with a DOAC. 3 5 In such patients, the risk of underdosing could be mitigated if the dose reduction is limited to patients with consistently high drug levels. 6 42 Although the use of off-label dose adjustment in selected patients is debated, 3 66 67 68 there is evidence that drug levels are associated with thromboembolic and bleeding events, 10 34 36 69 70 71 and that use of the dosages currently approved for patients with AF is likely to result in unacceptably high bleeding risks in selected patients. 72 73

### Strengths and Limitations

The main strengths are the comprehensive search with inclusion of more than 50 studies of patients with AF from across the globe. As a result, our estimates of the usual on-therapy ranges are more comprehensive and representative than those currently reported in guidelines and product monographs.

Limitations of our study include (1) the relative paucity of data for some dosing regimens, (2) the fact that usual on-therapy ranges do not represent therapeutic ranges, (3) the variation in the outcomes among studies (heterogeneity). In addition, despite attempts to perform sensitivity analyses, these analyses were mostly inconclusive, and we were unable to definitively explore the impact of risk of bias, concern of inapplicability, and differences in laboratory methods on the ranges of drug levels. It is possible that heterogeneity is in part contributed to by differences in the studied populations and methods of measurement. Consequently, it is important for diagnostic laboratories to assess measurement of uncertainty for their assays to guide the interpretation of drug levels. 74 75 76 If usual on-therapy ranges are used to help in making decisions, it is recommended that laboratories use validated assays that have been calibrated according to current standards. 5 74 75 76 When providing test results, laboratories may consider taking uncertainty about our usual on-therapy ranges into consideration, for instance, by reporting whether levels fall within the usual on-therapy range, within its 95% CIs, or outside either range. 74 Finally, if the dose is modified because the test falls outside the usual range, we suggest that the assay should be repeated to determine that the dose response is appropriate.

## Conclusion

In this systematic review and meta-analysis, we pooled data from 53 studies that collectively included over 30,000 patients to provide updated estimates for the 10 ^th^ to 90 ^th^ percentile ranges (i.e., middle 80% of drug levels) for trough and peak concentrations of the four approved DOACs when used in patients with AF using methods commonly used in clinical practice. This update provides clinicians with more representative estimates of the usual on-therapy ranges of DOACs than previously reported, to help guide their decision-making.

## Notes

**What is known about this topic?**

- DOACs do not require routine monitoring of drug levels, but measuring a drug level might be desirable in some situations.
- In the absence of therapeutic ranges, usual on-therapy ranges (10 ^th^ to 90 ^th^ ) of drug concentrations are used to interpret measurements.
- Existing guidance documents provide usual on-therapy ranges for drug concentrations, but these have important limitations.

**What does this paper add?**

- This systematic review and meta-analysis provides updated and more representative usual on-therapy ranges of DOAC levels in patients with AF.
