# Supplementary material for: Usual On-therapy Ranges of Drug Concentrations in Patients with Atrial Fibrillation Treated with Direct Oral Anticoagulants: A Systematic Review and Meta-analysis
Source: Thromb Haemost. 2024 Nov 21;125(6):563–73. doi: 10.1055/a-2446-1348 (PMC12115550; doi:10.1055/a-2446-1348)
Supplement: Supplementary file 1 — Supporting Information File 1 [file 10-1055-a-2446-1348-s24030110-1.pdf]

Supporting Information File 1 — PRISMA checklist and literature search

Usual On-therapy Ranges of Drug Concentrations in Patients With Atrial Fibrillation Treated With Direct Oral Anticoagulants: a Systematic Review and Meta-analysis

Last updated on: August 7, 2024

Table of Contents

Table S1. PRISMA checklist 3

Table S2. Search strategy employed in MEDLINE through Ovid 9

References 10

Table S1. PRISMA checklist

Section and Topic	Item #	Checklist item	Location where item is reported
**TITLE**			
Title	1	Identify the report as a systematic review.	Page 1
**ABSTRACT**			
Abstract	2	See the PRISMA 2020 for Abstracts checklist.	Page 3
**INTRODUCTION**			
Rationale	3	Describe the rationale for the review in the context of existing knowledge.	Page 4-5
Objectives	4	Provide an explicit statement of the objective(s) or question(s) the review addresses.	Page 5
**METHODS**			
Eligibility criteria	5	Specify the inclusion and exclusion criteria for the review and how studies were grouped for the syntheses.	Page 5-6
Information sources	6	Specify all databases, registers, websites, organisations, reference lists and other sources searched or consulted to identify studies. Specify the date when each source was last searched or consulted.	Page 5
Search strategy	7	Present the full search strategies for all databases, registers and websites, including any filters and limits used.	Page 5, and Table S2 in the Supporting Information File 1
Selection process	8	Specify the methods used to decide whether a study met the inclusion criteria of the review, including how many reviewers screened each record and each report retrieved, whether they worked independently, and if applicable, details of automation tools used in the process.	Page 6, and in the study protocol in Supporting Information File 2
Data collection process	9	Specify the methods used to collect data from reports, including how many reviewers collected data from each report, whether they worked independently, any processes for obtaining or confirming data from study investigators, and if applicable, details of automation tools used in the process.	Page 6-7, and in the study protocol in Supporting Information File 2
Data items	10a	List and define all outcomes for which data were sought. Specify whether all results that	Page 7, and in the

Section and Topic	Item #	Checklist item	Location where item is reported
		were compatible with each outcome domain in each study were sought (e.g. for all measures, time points, analyses), and if not, the methods used to decide which results to collect.	study protocol in Supporting Information File 2
	10b	List and define all other variables for which data were sought (e.g. participant and intervention characteristics, funding sources). Describe any assumptions made about any missing or unclear information.	Page 6-7, and in the study protocol in Supporting Information File 2
Study risk of bias assessment	11	Specify the methods used to assess risk of bias in the included studies, including details of the tool(s) used, how many reviewers assessed each study and whether they worked independently, and if applicable, details of automation tools used in the process.	Page 8-9, the study protocol in Supporting Information File 2, and Supporting Information File 3
Effect measures	12	Specify for each outcome the effect measure(s) (e.g. risk ratio, mean difference) used in the synthesis or presentation of results.	Page 6-7, and in the study protocol in Supporting Information File 2
Synthesis methods	13a	Describe the processes used to decide which studies were eligible for each synthesis (e.g. tabulating the study intervention characteristics and comparing against the planned groups for each synthesis (item #5)).	Page 6-8, and in the study protocol in Supporting Information File 2
	13b	Describe any methods required to prepare the data for presentation or synthesis, such as handling of missing summary statistics, or data conversions.	Page 6-7, and in the study protocol in Supporting Information File 2
	13c	Describe any methods used to tabulate or visually display results of individual studies and syntheses.	Study protocol in Supporting Information File 2
	13d	Describe any methods used to synthesize results and provide a rationale for the choice(s). If meta-analysis was performed, describe the model(s), method(s) to identify the presence and extent of statistical heterogeneity, and software package(s) used.	Page 7, the study protocol in Supporting Information File 2, and

Section and Topic	Item #	Checklist item	Location where item is reported
			Supporting Information File 3*
	13e	Describe any methods used to explore possible causes of heterogeneity among study results (e.g. subgroup analysis, meta-regression).	Page 8 and Supporting Information File 2 (though formally a sensitivity analysis)
	13f	Describe any sensitivity analyses conducted to assess robustness of the synthesized results.	Page 8 and Supporting Information File 2
Reporting bias assessment	14	Describe any methods used to assess risk of bias due to missing results in a synthesis (arising from reporting biases).	NA†
Certainty assessment	15	Describe any methods used to assess certainty (or confidence) in the body of evidence for an outcome.	Page 8-9, the study protocol in Supporting Information File 2, and Supporting Information File 3
**RESULTS**			
Study selection	16a	Describe the results of the search and selection process, from the number of records identified in the search to the number of studies included in the review, ideally using a flow diagram.	Page 9 and Fig 1
	16b	Cite studies that might appear to meet the inclusion criteria, but which were excluded, and explain why they were excluded.	Table S1 in Supporting Information File 4
Study characteristics	17	Cite each included study and present its characteristics.	Table S1 in Supporting Information File 4
Risk of bias in studies	18	Present assessments of risk of bias for each included study.	'Results on usual on-therapy ranges in more detail' in Supporting Information File 4, and Table S7-S8 in Supporting

Section and Topic	Item #	Checklist item	Location where item is reported
			Information File 3
Results of individual studies	19	For all outcomes, present, for each study: (a) summary statistics for each group (where appropriate) and (b) an effect estimate and its precision (e.g. confidence/credible interval), ideally using structured tables or plots.	Page 10-13, and Supporting Information File 4
Results of syntheses	20a	For each synthesis, briefly summarise the characteristics and risk of bias among contributing studies.	'Results on usual on-therapy ranges in more detail' in Supporting Information File 4, and Page 10-13, and Supporting Information File 4
	20b	Present results of all statistical syntheses conducted. If meta-analysis was done, present for each the summary estimate and its precision (e.g. confidence/credible interval) and measures of statistical heterogeneity. If comparing groups, describe the direction of the effect.	Page 10-13, and Supporting Information File 4
	20c	Present results of all investigations of possible causes of heterogeneity among study results.	Page 13, and Supporting Information File 5 (though formally a sensitivity analysis)
	20d	Present results of all sensitivity analyses conducted to assess the robustness of the synthesized results.	Page 13, and Supporting Information File 5
Reporting biases	21	Present assessments of risk of bias due to missing results (arising from reporting biases) for each synthesis assessed.	NA†
Certainty of evidence	22	Present assessments of certainty (or confidence) in the body of evidence for each outcome assessed.	Page 13, 'Results on usual on-therapy ranges in more detail' in Supporting Information File 4, and Table S12 in

Section and Topic	Item #	Checklist item	Location where item is reported
			Supporting Information File 3†
**DISCUSSION**			
Discussion	23a	Provide a general interpretation of the results in the context of other evidence.	Page 14-15
	23b	Discuss any limitations of the evidence included in the review.	Page 15
	23c	Discuss any limitations of the review processes used.	Page 15
	23d	Discuss implications of the results for practice, policy, and future research.	Page 14-15
**OTHER INFORMATION**			
Registration and protocol	24a	Provide registration information for the review, including register name and registration number, or state that the review was not registered.	NA‡
	24b	Indicate where the review protocol can be accessed, or state that a protocol was not prepared.	Supporting Information File 2
	24c	Describe and explain any amendments to information provided at registration or in the protocol.	Page 8
Support	25	Describe sources of financial or non-financial support for the review, and the role of the funders or sponsors in the review.	Page 17
Competing interests	26	Declare any competing interests of review authors.	Page 17-18
Availability of data, code and other materials	27	Report which of the following are publicly available and where they can be found: template data collection forms; data extracted from included studies; data used for all analyses; analytic code; any other materials used in the review.	Page 17§

This table provides an overview of Preferred Reporting Items for Systematic Reviews and Meta-Analyses (PRISMA).[1]

NA not applicable.

[*] All analyses were performed in R version 4.4.1 on a Windows device;[2] [†] We did not assess for potential publication bias (rationale described in **Table S5 of Supporting Information File 3**); [‡] We did not pre-register our protocol at PROSPERO because we initiated data collection in parallel with the quality assessments. The reviewers primarily responsible for data collection were not involved with the quality assessments and vice verse. Registration after commencement of data collection is not in accordance with the PROSPERO guidelines.[3] However, our protocol, including a detailed description of our statistical analysis plan, is provided in **Supporting Information File 2**; [§] The data underlying this article are in **Supporting Information File 6** and the other supporting information files. The R-syntax underlying this paper will be shared on reasonable request to the corresponding author.

Table S2. Search strategy employed in MEDLINE through Ovid

PICO[4] *	Terms
Population of interest†	(“atrial fibrillation” OR “venous thromboembolism”)
	AND
Investigated test	(“apixaban” OR “dabigatran” OR “edoxaban” OR "rivaroxaban” OR “NOAC” OR “DOAC”)
	AND
Outcome	(“pharmacokinetic*” OR “pharmacodynamic*” OR “coagulation test*” OR “drug level*” OR “HP/LC” OR “chromatography” OR “anti-Xa activity” OR “plasma drug level*” OR “trough” OR “peak”)

This table shows the search strategy as employed in MEDLINE through Ovid. The strategy was constructed with the help of a medical librarian who, because of the large number of hits, advised us to use fewer yet specific search terms to cast a wider net for studies that reported drug levels. To ensure the search terms were adequate, we ensured that our search string captured major papers of similar themes to the research question.

* As we do not compare the accuracy of one test to another, the C-category (i.e., control/compared test) of the PICO is not applicable to our review.

† We included venous thromboembolism in our literature search because our goal is to perform a similar study on patients using direct oral anticoagulants for the treatment or prevention of venous thromboembolisms in the future.

References

1. Moher D, Liberati A, Tetzlaff J, Altman DG. Preferred reporting items for systematic reviews and meta-analyses: the PRISMA statement. *PLoS Med*. 2009;6(7):e1000097
2. (2022). R: A language and environment for statistical computing. [Computer Program]. R Foundation for Statistical Computing, Vienna, Austria.
3. PROSPERO. Guidance Notes. https://www.crd.york.ac.uk/prospero/#guidancenotes. Accessed
4. Luijendijk HJ. How to create PICO questions about diagnostic tests. *BMJ Evidence-Based Medicine*. 2021;26(4):155
