# Supplementary material for: Usual On-therapy Ranges of Drug Concentrations in Patients with Atrial Fibrillation Treated with Direct Oral Anticoagulants: A Systematic Review and Meta-analysis
Source: Thromb Haemost. 2024 Nov 21;125(6):563–73. doi: 10.1055/a-2446-1348 (PMC12115550; doi:10.1055/a-2446-1348)
Supplement: Supplementary file 2 — Supporting Information File 2 [file 10-1055-a-2446-1348-s24030110-2.pdf]

Supporting Information File 2

Protocol and Statistical Analysis Plan

Usual On-therapy Ranges of Drug Concentrations in Patients With Atrial Fibrillation Treated With Direct Oral Anticoagulants: a Systematic Review and Meta-analysis

Last updated on: 29 November 2023

Protocol version: 5 January 2023

Table of Contents

Background.. 3
Methods 4
Data sources and searches:.. 4
Study selection: 4
Data extraction: 5
Estimation of non-reported percentiles based on available distribution measures: 6
Meta-analyses 6
Sensitivity analyses: Assessing the robustness of the pooled 10th and 90th percentile intervals of drug levels 8
Quality assessments: 8
Risk of bias and indirectness assessments: 9
Imprecision assessment: 10
Inconsistency assessment: 10
Publication bias assessment: 11
GRADING the evidence: 11
References 12

Background

Current guidelines recommend the use of direct oral anticoagulants (DOACs) over vitamin K antagonists in most patients with atrial fibrillation (AF) at risk of ischemic stroke, and advocate against routine laboratory monitoring.[1,2] However, there are patient populations in which measuring drug levels might be desirable to guide dosing.[3-6] These population include those that were not well-represented in the randomized controlled trials (RCTs) in which current dosing recommendations were tested (i.e., apixaban, rivaroxaban, edoxaban) or from which they were derived (i.e., dabigatran).[7-12].Examples of patients who were underrepresented in the RCTs are those who recently experienced a thromboembolic or bleeding event while on treatment and dosed accordance with the labelled dosing recommendations, those at high-risk of underexposure or overexposure to DOACs due to other clinical characteristics (e.g., severe renal or liver insufficiency, extreme body weight, frail or the very elderly, use of drugs that interact with DOACs), and those who require urgent surgical interventions or procedures associated with extreme risks of bleeding.[2,3,13-15]

Because a dose adjustments strategy based on drug level measurements has not yet been tested in a randomized trial, it is uncertain if such a strategy improves clinical outcomes, and is cost effective. However, we believe that selected patients may benefit from a drug level measurement to optimize dosing.[3,8,13-18] Supporting this assertion are the findings of a strong relationship between increasing drug levels and higher bleeding risk, and a weaker and inverse relationship between drug levels and thrombosis in pharmacokinetic substudies of the pivotal phase III trials of the DOACs.[3,8,13-18]

In the absence of therapeutic ranges for the DOACs, the concept of usual on-therapy ranges (10th to 90th percentile intervals of trough or peak levels) has been proposed as a guide for interpretation of drug levels.[2,19]

In selected patients in whom there is uncertainty about the DOAC dose, clinicians could measure drug levels and use the usual on-treatment range to interpret. Such a comparison could support the decision to continue or to adjust (increase or lower) the current dose.

Usual on-therapy ranges of DOACs have been published in guidance documents, but were derived from a limited number of studies and not always from patients with atrial fibrillation.[2] In this systematic review and meta-analysis, we aim to provide clinicians with an improved usual on-therapy range (median with 10th to 90th percentile interval) of both trough and peak levels of the four available DOACs when given in conventional doses to patients with AF.

Methods

Data sources and searches:

We will electronically search MEDLINE via OVID for articles reporting on plasma concentrations of DOACs in patients with AF treated with DOACs to prevent ischemic strokes published between the inception of the database till the date of search. Throughout this report we use drug levels and plasma concentrations interchangeably. The full search strategy is presented in **Supporting Information File 1**.

Study selection:

Studies were eligible for inclusion if they reported on measures of distribution of either or both DOAC trough and peak levels in ng/ml (or in units that allowed for direct conversion to ng/ml), measured in patients with AF who were being treated with any of the four direct oral anticoagulants at the time of the measurement. Only studies that reported on DOAC doses that are approved for use in clinical practice for the prevention of strokes in AF will be included. We will avoid double counting by using data of interest from the more comprehensive publication.

We will exclude case reports or care series, studies on animals or healthy subjects, pharmacokinetic simulation studies, studies that did not provide levels for patients AF

independently from those who were treated with DOACs for other indications (e.g., treatment or prevention of venous thromboembolism), as well as studies that did not report drug levels by DOAC type and administered dose. Our rationale for excluding studies that do not differentiate between the reduced and standard doses is that prior reports consistently show that those treated with a reduced dose have lower levels than those using standard doses, despite such patients having factors that increase the exposure of DOACs.[3,8,17,18,20,21] We will further exclude non-English studies, and those which included fewer than ten patients for each DOAC dosing subgroups to reduce the effect of extreme distribution of drug levels by chance. After deduplication, all hits will be screened for eligibility in duplication (IM and CG). In the instance of disagreement, the final decision will be determined by a third reviewer (TdV or NC).

Data extraction:

From all included studies, per DOAC type and administered dose, we will extract the number of patients and the measures of distribution of trough and peak DOAC drug levels (i.e., the minimum, 5th percentile, 10th percentile, 25th percentile, median, 75th percentile, 90th percentile, 95th percentile, maximum, mean, and standard deviation [SD]).

We will further extract study characteristics, which includes (A) the design of the study into which the cross-sectional analyses to determine drug levels was embedded (i.e., randomized controlled trial or observational study), (B) the assay used to measure DOAC drug levels, and (C) key clinical attributes of the population under study. We defined such attributes as the inclusion and exclusion criteria used to define the target population. We will not request authors of included studies to provide us with further information if data are unavailable, but we will collect unreported data on drug levels from primary studies authored by the authors of this review.[13,22,23]

Estimation of non-reported percentiles based on available distribution measures:

For primary studies reporting insufficient parameters for them to be included in our analyses to determine pooled estimates, we will first estimate non-reported percentiles using simulations based on parameters of the observed distribution. We will employ various simulation strategies, outlined below.

First, in studies which report the median and other distribution measures (e.g., min, max, 25th percentile, 75th percentile) but not all those of interest, random data will be generated from lognormal distributions using a mean and SD estimated based on the available distribution measures. The assumed mean and SD were refined in subsequent iterations until the generated data are a reasonable fit of the reported measures.

Second, for studies that provide a mean with other distribution measures, but no SD, a SD will be assumed to generate data iteratively by revising the SD until the simulated data are a reasonable fit of the reported measures.

Third, for studies that provide both a mean and a SD only, required percentiles will be calculated directly from a lognormal distribution which are to be specified by the reported mean and SD. If a study also provides other measures, such as the 5th, 50th, and 95th percentiles, simulations will be performed to find a distribution with the same median and percentiles as those reported. We accept differences between the reported and simulated means and SDs.

We define a reasonable fit as a difference of <1 ng/ml between fitted and reported values. If the lognormal distribution fails to converge following the mentioned criteria, we repeat the described steps but instead assume a normal distribution.

Meta-analyses

In our first main analysis we will estimate the pooled median trough or peak level for each DOAC dose using the quantile estimation (QE) method. This method allows for valid pooling of medians when different combinations of summary statistics are available from primary

studies.[24] Specifically, this method estimates the variance of the study-specific medians from the reported summary statistics and then performs an inverse-variance weighted meta-analysis of medians. The QE method considers that each study reports one of the following combinations of summary statistics: (i) mean and SD; (ii) median with the minimum, 25th percentile, 75th percentile, and maximum; (iii) median with the 25th and 75th percentiles; and (iv) median with the minimum and maximum values.[24,25] This method assumes that the DOAC drug levels in each study follows one of several candidate parametric distributions (e.g., normal distribution, log-normal distribution).

We will prioritize reported over simulated values, and also the combination with the most summary statistics whenever multiple combinations are reported. However, because prior reports indicate the distributions of DOAC drug levels are right-skewed,[13,17,22,26,27] we decided to not include the reported mean and SD into the QE method because it assumes that data are normally distributed in this case. Accordingly, for studies reporting either only a mean and SD or a combination of summary statistics not considered by the QE method, we will use the simulated median, 25th and 75th percentile values instead.

In our second main analysis, we adapted the QE method to estimate the pooled 10th and the pooled 90th percentile levels of trough and peak levels for each DOAC dosing regimen.[24,25] Similar to the application of QE method to meta-analyze medians, we will estimate the variance of the study-specific 10th and 90th percentiles from the reported summary statistics in order to perform an inverse variance weighted meta-analysis of the 10th and 90th percentiles. We will apply the same prioritization strategy as described to estimate the pooled median drug levels.

Given our aim, target population, and method of sampling the literature, we will use random effects models (REMs) in all models.[28] All findings will be presented in forest plots. We will assess these forest for potential heterogeneity of the results by visual examination of the point estimates and their 95% confidence intervals (CIs); tested for statistically significant differences using the chi-square (X^2) test; and quantified with the I^2 statistic. In addition, if

studies reported on multiple subgroups on the same DOAC type and dosing regimen, we will consider these subgroups unique populations.

Sensitivity analyses: Assessing the robustness of the pooled 10th and 90th percentile intervals of drug levels

To assess the robustness of our findings on the pooled 10th and 90th percentiles, we will perform two sets of sensitivity analyses for DOAC dosing regimen. We will only perform these analyses whenever at least ten studies reported on the value of interest.[29]

First, we will estimate the pooled 10th and 90th percentile values focusing only on the studies that reported such values. We will then repeat this but selecting only the studies for which we had to simulate the 10th and 90th percentile values. Second, we will also reperform all estimations selecting only the studies at low risk of bias and low concern of inapplicability to our review question, and then repeat this but selected the studies not fitting both these criteria.

In both sets of sensitivity analyses, we will use mixed-effects models to assess for significant differences between the subsets using the dichotomized variable as a potential modifier (i.e., reported vs. simulated values, or low risk of bias and low concern of inapplicability vs. other studies). We will keep Tau constant if there are five or fewer studies in either subset,[29] and define a significant difference between the subsets as a Wald-test producing a two-tailed p-value <0.05.

Quality assessments:

We adopt the Grading of Recommendations Assessment, Development and Evaluation system (GRADE) and will rate the quality of evidence for the pooled median and pooled 10th to 90th percentile ranges of each DOAC dosing regimen.[30] Our considerations and criteria for the quality assessment are summarized in **Supporting Information File 3** and discussed below.

Risk of bias and indirectness assessments:

Through iterative discussion and refinements after piloting in a selection of included studies, we developed an instrument based on the QUADAS-2 and ROBINS-I tool (**Supporting Information File 3**) to categorize studies according to their risk of bias (i.e., risk of bias assessment) and our concerns regarding their applicability to our review question (i.e., indirectness assessment).[31,32] The QUADAS-2 tool assesses these attributes in four domains (i.e., patient selection, index test, reference standard, and flow and timing).[32] We combined domain two (index test), three (reference standard), and four (flow and timing) into a single domain (i.e., test) because we do not compare an index test to a reference test.

Using this tool, half of included studies will be reviewed independently by two assessors (TdV and NC) and the other half by two different assessors (JE and VB). Any disagreement in the final judgement among one pair will be solved by discussion between the paired reviewers. If consensus cannot be reached, a co-author of the other pair will be involved to reach a final decision.

Studies will be classified as at high, low, or unclear risk of bias according to criteria mentioned in the tailored QUADAS-2 tool (**Supporting Information File 3**). We will consider a study to be at high risk of bias if at least one signalling question is answered with 'No', indicating high risk of bias. Studies will be labelled as being at unclear risk of bias if none of the signalling question indicated high risk of bias, but at least one signalling question indicates unclear risk of bias. For each outcome of interest, the level of evidence will be rated down by one grade if ≥25% of the included studies were at high risk of bias. We will also rate the level of evidence down by one grade if ≥50% of the included studies were at either high or unclear risk of bias.

Studies will be classified also as at high, low, or unclear concern of inapplicability to our review question according to criteria mentioned in the tailored QUADAS-2 tool (**Supporting Information File 3**). We consider a study to be at high concern of inapplicability to our review question if at least one signalling question is answered with 'No', indicating high concern.

Studies will be labelled as at unclear risk of concern if none of signalling question indicate high concern of inapplicability, but at least one signalling question indicates unclear concern. For each outcome of interest, the level of evidence will be rated down by one grade if ≥25% of the included studies were at high risk of indirectness. We will also rate the level of evidence down by one grade if ≥50% of the included studies were at either high or unclear risk of indirectness.

Imprecision assessment:

We will rate down for imprecision by three levels if the total number of patients included in the analyses was ≤25, by two levels if this number was ≤50, and by one level if ≤250 patients are available. We upgraded the level of evidence by one level if the total number of patients included in an analysis was ≥1000.

Hence, to detect a d-type effect size of 0.8 (large effect), 0.5 (moderate effect), 0.2 (small effect), or 0.1 (very small effect) standard deviations, with an alpha of 5% and a power of 80% using a two-tailed one-sample Wilcoxon signed rank test and assuming a min ARE distribution, approximately 17, 39, 230, and 911 patients, respectively, are required. These calculations were performed with G*Power version 3.1.9.7.[33]

We decided to not consider the width of the 95% confidence intervals to rate down for imprecision because we wanted to avoid rating down for imprecision when the wide confidence intervals were driven by inconsistency in the point estimates of the included primary studies. We believed this to be appropriate because we (i) used random effects models to meta-analyse the available data, and (ii) we anticipated to include heterogenous populations.[34]

Inconsistency assessment:

In all analyses, limited consistency of point estimates and the overlap of 95% confidence intervals, a two-tailed p-value of <0.10 of the X^2-test and/or an I^2 of ≥50% are considered signals of substantial heterogeneity. If all these assessments are in agreement, evidence will

be rated down one level due to inconsistent results. If any of these assessments are in disagreement, results will be discussed among two of the co-authors (TdV and NC) to arrive at a final conclusion to rate down the level of evidence one grade for inconsistency.

Publication bias assessment:

We decided not to perform an assessment to screen for potential publication bias because, for this type of meta-analysis, it is challenging to assess the degree of non-reporting bias.

First, because we anticipated it unlikely for entire reports, or particular results, of smaller studies to remain unpublished because of their findings on interpatient variation of DOAC drug levels.[35]

Second, because we expected studies on patients with more extreme characteristics to be smaller in sample size than studies on more typical populations. Differences in outcomes of interest between smaller and larger studies would therefore primarily be due to the differences between the populations and not due to publication bias.

GRADING the evidence:

Confidence in the pooled effects estimates (i.e., median, 10th percentile, and 90th percentile) were evaluated using the GRADE approach.[30] The quality of evidence will be considered high for each outcome of interest but will be rated down according to the assessments on the risk of bias, imprecision, inconsistency, indirectness, and publication bias.

References

1. Hindricks G, Potpara T, Dagres N*, et al.* 2020 ESC Guidelines for the diagnosis and management of atrial fibrillation developed in collaboration with the European Association for Cardio-Thoracic Surgery (EACTS): The Task Force for the diagnosis and management of atrial fibrillation of the European Society of Cardiology (ESC) Developed with the special contribution of the European Heart Rhythm Association (EHRA) of the ESC. *Eur Heart J*. 2021;42(5):373-498
2. Steffel J, Collins R, Antz M*, et al.* 2021 European Heart Rhythm Association Practical Guide on the Use of Non-Vitamin K Antagonist Oral Anticoagulants in Patients with Atrial Fibrillation. *Europace*. 2021;23(10):1612-1676
3. Toorop MMA, Lijfering WM, Scheres LJJ. The relationship between DOAC levels and clinical outcomes: The measures tell the tale. *J Thromb Haemost*. 2020;18(12):3163-3168
4. Chan N, Sager PT, Lawrence J*, et al.* Is there a role for pharmacokinetic/pharmacodynamic-guided dosing for novel oral anticoagulants? *Am Heart J*. 2018;199:59-67
5. Douxfils J, Adcock DM, Bates SM*, et al.* 2021 Update of the International Council for Standardization in Haematology Recommendations for Laboratory Measurement of Direct Oral Anticoagulants. *Thromb Haemost*. 2021;121(08):1008-1020
6. Douxfils J, Ageno W, Samama CM*, et al.* Laboratory testing in patients treated with direct oral anticoagulants: a practical guide for clinicians. *J Thromb Haemost*. 2018;16(2):209-219
7. Connolly SJ, Ezekowitz MD, Yusuf S*, et al.* Dabigatran versus Warfarin in Patients with Atrial Fibrillation. 2009;361(12):1139-1151
8. European Medicines Agency. Assessment report. INN/active substance: direct oral anticoagulants (DOACs). *Committee for Medicinal Products for Human Use (CHMP)*; March 26, 2020, 2020.
9. Connolly SJ, Eikelboom J, Joyner C*, et al.* Apixaban in patients with atrial fibrillation. *N Engl J Med*. 2011;364(9):806-817
10. Giugliano RP, Ruff CT, Braunwald E*, et al.* Edoxaban versus Warfarin in Patients with Atrial Fibrillation. *N Engl J Med*. 2013;369(22):2093-2104
11. Granger CB, Alexander JH, McMurray JJ*, et al.* Apixaban versus warfarin in patients with atrial fibrillation. *N Engl J Med*. 2011;365(11):981-992
12. Patel MR, Mahaffey KW, Garg J*, et al.* Rivaroxaban versus Warfarin in Nonvalvular Atrial Fibrillation. *N Engl J Med*. 2011;365(10):883-891

13. de Vries TAC, Hirsh J, Bhagirath VC*, et al.* Can a Single Measurement of Apixaban Levels Identify Patients at Risk of Overexposure? A Prospective Cohort Study. *TH Open*. 2022;06(01):e10-e17
14. de Vries TAC, Hirsh J, Chan NC. Letter by de Vries et al Regarding Article "Off-Label Under- and Overdosing of Direct Oral Anticoagulants in Patients With Atrial Fibrillation: A Meta-Analysis". *Circ Cardiovasc Qual Outcomes*. 2022;15(5):e008982
15. de Vries TAC, Hirsh J, Xu K*, et al.* Apixaban for Stroke Prevention in Atrial Fibrillation: Why are Event Rates Higher in Clinical Practice than in Randomized Trials?-A Systematic Review. *Thromb Haemost*. 2020;120(9):1323-1329
16. Bhagirath V, Eikelboom J, Hirsh J*, et al.* Apixaban-Calibrated Anti-FXa Activity in Relation to Outcome Events and Clinical Characteristics in Patients with Atrial Fibrillation: Results from the AVERROES Trial. *TH Open*. 2017;1(2):e139–e145
17. Ruff CT, Giugliano RP, Braunwald E*, et al.* Association between edoxaban dose, concentration, anti-Factor Xa activity, and outcomes: an analysis of data from the randomised, double-blind ENGAGE AF-TIMI 48 trial. *Lancet*. 2015;385(9984):2288-2295
18. Zhang L, Yan X, Fox KAA*, et al.* Associations between model-predicted rivaroxaban exposure and patient characteristics and efficacy and safety outcomes in patients with non-valvular atrial fibrillation. *J Thromb Thrombolysis*. 2020;50(1):20-29
19. Reda S, Rudde E, Müller J*, et al.* Variation in Plasma Levels of Apixaban and Rivaroxaban in Clinical Routine Treatment of Venous Thromboembolism. *Life (Basel)*. 2022;12(5)
20. Reilly PA, Lehr T, Haertter S*, et al.* The effect of dabigatran plasma concentrations and patient characteristics on the frequency of ischemic stroke and major bleeding in atrial fibrillation patients: the RE-LY Trial (Randomized Evaluation of Long-Term Anticoagulation Therapy). *J Am Coll Cardiol*. 2014;63(4):321-328
21. Testa S, Legnani C, Antonucci E*, et al.* Drug levels and bleeding complications in atrial fibrillation patients treated with direct oral anticoagulants. *J Thromb Haemost*. 2019;17(7):1064-1072
22. Shyamkumar K, Hirsh J, Bhagirath VC*, et al.* Plasma Rivaroxaban Level to Identify Patients at Risk of Drug Overexposure: Is a Single Measurement of Drug Level Reliable? *TH Open*. 2021;5(1):e84-e88
23. Bhagirath VC, Chan N, Hirsh J*, et al.* Plasma Apixaban Levels in Patients Treated Off Label With the Lower Dose. *J Am Coll Cardiol*. 2020;76(24):2906-2907
24. McGrath S, Sohn H, Steele R, Benedetti A. Meta-analysis of the difference of medians. *Biom J*. 2020;62(1):69-98

25. McGrath S, Zhao X, Ozturk O*, et al.* metamedian: An R package for meta-analyzing studies reporting medians. *Res Synth Methods, in press*. 2023
26. Chan NC, Coppens M, Hirsh J*, et al.* Real-world variability in dabigatran levels in patients with atrial fibrillation. *J Thromb Haemost*. 2015;13(3):353-359
27. Testa S, Tripodi A, Legnani C*, et al.* Plasma levels of direct oral anticoagulants in real life patients with atrial fibrillation: Results observed in four anticoagulation clinics. *Thromb Res*. 2016;137:178-183
28. Borenstein M, Hedges LV, Higgins JP, Rothstein HR. A basic introduction to fixed-effect and random-effects models for meta-analysis. *Res Synth Methods*. 2010;1(2):97-111
29. Harrer M, Cuijpers P, Furukawa TA, Ebert DD. Doing Meta-Analysis With R: A Hands-On Guide. 1st ed. Boca Raton, FL and London: Chapman & Hall/CRC Press; 2021
30. Guyatt GH, Oxman AD, Schünemann HJ, Tugwell P, Knottnerus A. GRADE guidelines: A new series of articles in the Journal of Clinical Epidemiology. *Journal of Clinical Epidemiology*. 2011;64(4):380-382
31. Sterne JA, Hernán MA, Reeves BC*, et al.* ROBINS-I: a tool for assessing risk of bias in non-randomised studies of interventions. *Bmj*. 2016;355:i4919
32. Whiting PF, Rutjes AW, Westwood ME*, et al.* QUADAS-2: a revised tool for the quality assessment of diagnostic accuracy studies. *Ann Intern Med*. 2011;155(8):529-536
33. Faul F, Erdfelder E, Lang A-G, Buchner A. G*Power 3: A flexible statistical power analysis program for the social, behavioral, and biomedical sciences. *Behavior Research Methods*. 2007;39(2):175-191
34. Schünemann HJ, Neumann I, Hultcrantz M*, et al.* GRADE guidance 35: update on rating imprecision for assessing contextualized certainty of evidence and making decisions. *J Clin Epidemiol*. 2022;150:225-242
35. Page MJ, Higgins JPT, Sterne JAC. Chapter 13: Assessing risk of bias due to missing results in a synthesis. In: Higgins JPT, Thomas J, Chandler J, Cumpston M, Li T, Page MJ, Welch VA (editors). *Cochrane Handbook for Systematic Reviews of Interventions version 63 (updated February 2022) Cochrane, 2022 Available from wwwtrainingcochraneorg/handbook*. 2022
