# Supplementary material for: Usual On-therapy Ranges of Drug Concentrations in Patients with Atrial Fibrillation Treated with Direct Oral Anticoagulants: A Systematic Review and Meta-analysis
Source: Thromb Haemost. 2024 Nov 21;125(6):563–73. doi: 10.1055/a-2446-1348 (PMC12115550; doi:10.1055/a-2446-1348)
Supplement: Supplementary file 3 — Supporting Information File 3 [file 10-1055-a-2446-1348-s24030110-3.pdf]

Supporting Information File 3 — Quality assessments and GRADEing the evidence

Usual On-therapy Ranges of Drug Concentrations in Patients With Atrial Fibrillation Treated With Direct Oral Anticoagulants: a Systematic Review and Meta-analysis

Last updated on: 07 March 2024

Table of Contents

Development of the quality assessment tool to assess the risk of bias and risk of indirectness of included studies 4

Background 4

Phase 1: State the review question: 4

Phase 2: Draw a flow diagram for the primary study 7

Figure S1. The flow diagram of the ideal study design to measure trough and peak levels in the target population* 7

Phase 3: Risk of bias and applicability judgments 8

Table S1. Risk of bias due to patient selection 8

Table S2. Indirectness due to patient selection 9

Table S3. Risk of bias due to the laboratory test or the timing of measurement 10

Table S4. Characteristics related to pharmacokinetic properties of the individual direct oral anticoagulants 11

Criteria and considerations for each quality assessment and the interpretation of level of evidence ratings 12

Table S5. Criteria and considerations for each quality assessment 12

Table S6. Level of evidence ratings and their interpretation 16

Results of each quality assessment 17

Table S7. Final judgements of each domain of the risk of bias and indirectness assessments (using a tailored QUADAS-2 tool) of each primary study by direct oral anticoagulant type and dosing regimen 17

Table S8. Results of the risk of bias assessment per outcome of interest 23

Trough levels 23

Peak levels 26

Table S9. Results of indirectness assessment per outcome of interest 29

Trough levels 29

Peak levels 32

Table S10. Results of the inconsistency assessment of each outcome of interest 35

A. Trough levels 35

B. Peak levels 38

Table S11. Results of the imprecision assessment per outcome of interest 41

Level of evidence profiles using the GRADE criteria for all outcomes of interest 42

Table S12. GRADE evidence profile: median and 10th to 90th percentile interval of direct oral anticoagulant drug levels in patients with atrial fibrillation 42

A. Trough levels 42

A. Peak levels 45

References 48

Development of the quality assessment tool to assess the risk of bias and risk of indirectness of included studies

Background

For each study, we assessed the risk of bias and risk of inapplicability to our review question. We adapted the QUADAS-2 tool designed for diagnostic studies to our review question [1]. We combined domain two (index test), three (reference standard), and four (flow and timing) into a single domain,[1] because we are not comparing an index test to an available reference test. The main objective of this study is to pool data on the distribution of drug levels measured with a reference test to provide valid and precise estimates of an on-treatment range (10th to 90th percentiles) of drug levels of DOACs. We added selected questions from the ROBINS-I designed to assess the quality of non-randomized studies of interventions because we considered them to be relevant to our aim.[2] In this document we summarized the phases of the QUADAS-2 tool to tailor the tool to our review question.

Phase 1: State the review question:

What is the on-treatment range (median with 10th to 90th percentile interval) of drug levels at peak and trough in patients with atrial fibrillation (AF) taking approved doses of DOACs for the prevention of stroke in the community?

This question is being asked to provide clinicians who are considering measurement of DOAC drug levels with a reference range to compare the levels observed in their patients to support a decision to continue the current dosing regimen or to adjust (increase or lower) the dose. Increasing the dose may be considered in patients who are treated with a reduced dosing regimen off-label (i.e., in discordance with the labelled dosing recommendations) and who have unusually low drug levels, while reducing the dose may be considered in patients with unusually high drug levels who are treated with the standard dose.

It needs to be acknowledged that a dose adjustments strategy based on drug level measurements has not been tested in a randomized trial. It is uncertain if such a strategy improves clinical outcomes, or is cost efficient. However, selected patients, such as patients who recently experienced a thromboembolic or bleeding event and those at high-risk of under- or overexposure to DOACs, may benefit from a drug level measurement to optimize dosing.[3-8]

**Patients (setting, intended use of index test, presentation, prior testing):**
<u>Target population of our review:</u><br>The target population of our review are patients with atrial fibrillation who are treated with any of the four DOACs in doses approved for use in the prevention of stroke in AF.<br><br><u>Target population of our reference range:</u><br>The on-treatment ranges that follow from our review may be used to detect unusually high or low drug levels in a broad population of patients with atrial fibrillation.<br><br>Drug level measurements are not routinely performed, but are sometimes considered in selected subgroups of patients with AF, in particular those who recently developed a bleeding or thromboembolic event while on treatment, or those who are considered to be at risk of over- or underexposure to DOACs.

**Index test(s):**<br>Not applicable because the aim of our study is not to compare one test to another but to pool estimates derived from valid laboratory tests. We refer to such tests as the reference standard.
**Reference standard and target condition:**<br>Valid methods to measure DOAC drug levels either measure the plasma concentration directly (e.g., liquid chromatography-mass spectrometry) or indirectly by measuring anticoagulant activity (e.g., anti-Xa, diluted thrombin time) and referencing to a calibration curve to report results in drug concentration.[9-11]<br><br>If the test under study is valid (i.e., measures plasma levels directly or has strong agreement with liquid chromatography-mass spectrometry, which is considered the gold standard assay), results on DOAC drug levels are expected to be generalizable to clinical practice, regardless of its availability in practice.<br><br>The outcome of interest is not a target condition (e.g., an unusually high or low drug level) but the absolute value of DOAC drug levels.

Phase 2: Draw a flow diagram for the primary study

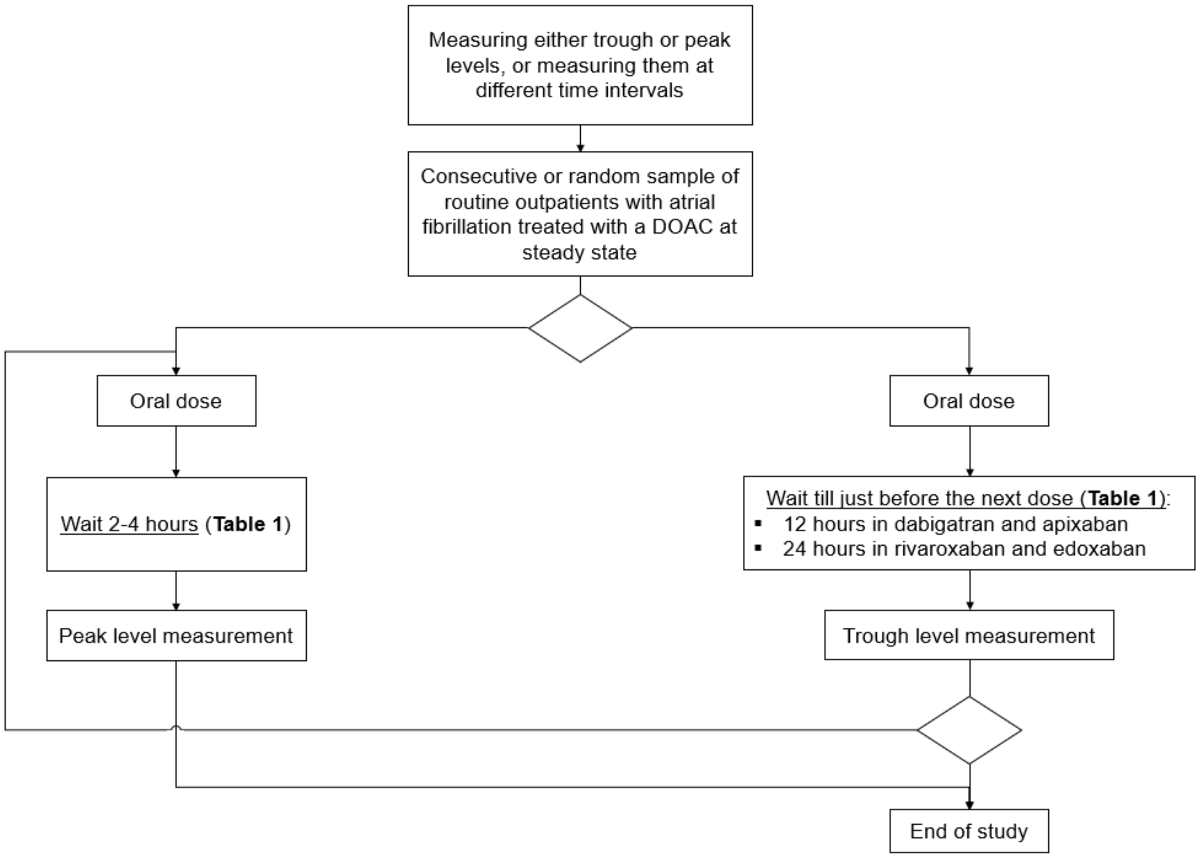

Figure S1. The flow diagram of the ideal study design to measure trough and peak levels in the target population[*]

[*] We decided to not draw flow diagrams for each primary study because all primary studies of interest to our review were likely to follow a patient flow similar to the one described above. We defined signaling questions to assess for relevant deviations from this ideal design.

Phase 3: Risk of bias and applicability judgments

QUADAS-2 is structured so that four domains are each rated in terms of the risk of bias and the concern regarding applicability (if applicable) to the research question. Each key domain has a set of signalling questions to optimize reproducibility in the assessment of bias and applicability.[1] We combined domain two (index test), three (reference standard), and four (flow and timing) into a single domain because most of the questions in each domain did not apply to our review question because we do not compare an index test to a reference test.

Table S1. Risk of bias due to patient selection

**DOMAIN 1: PATIENT SELECTION**	
**A. Risk of Bias**	
Describe method of patient selection:	
❖ Was a consecutive or random sample of patients enrolled?	Yes/No/Unclear
▪ Did the study avoid inappropriate exclusions or inclusions (of special population that could skew drug level distribution)?*	Yes/No/Unclear
▪ Were <5% participants excluded from the analyses of interest due to missing data?†	Yes/No/Unclear
**Could the selection of patients have introduced bias?**	**RISK: LOW/HIGH/UNCLEAR**

DOAC direct oral anticoagulant.

* For example, a study that aims to determine the interpatient variability of DOAC drug levels in a typical population treated in the community is likely to be biased if only patients with a recent bleeding visiting a specialized outpatient clinic are asked to participate. Another example that may lead to bias is systematic exclusion of patients for whom measuring DOAC drug levels in the setting of clinical research may be considered too onerous (e.g., frail elderly or those working full-time).

† For the sake of feasibility, we considered any type of missing data to be a reflection of a higher risk of bias if sufficiently prevalent. However, when observations of a variable are ‘missing completely at random’, results will be less precise but unbiased.[12]

Table S2. Indirectness due to patient selection

**DOMAIN 1: PATIENT SELECTION (continued)**
**B. Concerns regarding applicability**<br><br>Describe included patients (presentation, intended use of test and setting):<br><br><br><br>❖ Is the study population representative of a typical population treated in the community? — Yes/No/Unclear<br>We defined such a population as any population not selected because they were at high risk of under- or overexposure to DOACs.<br><br>❖ Were the patients under study treated with a DOAC for an adequate duration for them to be at a 'steady state' at the time of the measurement(s)? — Yes/No/Unclear<br>We defined steady state at least 4 days of use, which is just over five times the half-life of the DOAC with the longest half-life (**Table S4**).<br><br>**Is there concern that the included patients do not match the review question?** — **CONCERN: LOW/HIGH/UNCLEAR**

DOAC direct oral anticoagulant.

Table S3. Risk of bias due to the laboratory test or the timing of measurement

**DOMAIN 2-4: TEST** *, †	
**Risk of Bias**	
Describe the laboratory test used to measure drug levels and how it was conducted and interpreted:	
❖ Was the test used to measure DOAC drug levels valid?<br>Valid methods to measure DOAC drug levels either measure the plasma concentration directly (e.g., liquid chromatography-mass spectrometry) or indirectly by measuring anticoagulant activity (e.g., anti-Xa, diluted thrombin time) and referencing to a calibration curve to report results in drug concentration.[9-11]	Yes/No/Unclear
❖ Was the interval between the administered dose and the trough level measurement appropriate in (nearly) all patients?<br>We defined appropriate as between 9-15 hours for twice daily DOACs and 21-27 hours for once daily DOACs in ≥90% of the patients (**Table S4**). If unavailable, we approximated this proportion by using the measures of distribution of this time interval (e.g., mean and standard deviation, or median and interquartile interval).	Yes/No/Unclear/NA
❖ Was the interval between the administered dose and the peak level measurement appropriate in (nearly) all patients?<br>We defined an appropriate interval as between 1-5 hours in ≥90% of patients (**Table S4**). If unavailable, we approximated this proportion by using the measures of distribution of this time interval (e.g., mean and standard deviation, or median and interquartile interval).	Yes/No/Unclear/NA
**Could the conduct or interpretation of the test have introduced bias?**	**RISK: LOW/HIGH/UNCLEAR**

* In our study we do not compare an index test to an available reference test. Instead, we pool the measures of distribution of drug levels measured with a reference test to provide an on-treatment range of drug levels of DOACs. To this end, we combined domain 2 (index test), 3 (reference standard), and 4 (flow and timing) of the QUADAS-2 checklist into a single domain.

† If the test under study is valid, results on DOAC drug levels are expected to be generalizable to clinical practice, regardless of its availability in practice. We therefore did not consider inapplicability to our review question in this domain.

Table S4. Characteristics related to pharmacokinetic properties of the individual direct oral anticoagulants

	**Dabigatran**	**Rivaroxaban**	**Apixaban**	**Edoxaban**
Half-life [13]	12-17 hours	5-13 hours	12 hours	10-14 hours
Time to Cmin*	12 hours (twice daily dosing)	24 hours (once daily dosing)	12 hours (twice daily dosing)	24 hours (once daily dosing)
Time to Cmax† [13,14]	2-4 hours	2-4 hours	2-4 hours	2-4 hours
Labelled dose reduction criteria in Europe [14]	110 mg twice daily if age ≥80 y or concomitant verapamil or if high bleeding risk: age 75–80 y or moderate renal impairment or predisposition to gastro-intestinal bleeding	15 mg once daily if CrCl 15–49 mL/min	2.5 mg twice daily if at least two of the following: age ≥80 y, weight ≤60 kg, or serum creatinine ≥ 133 micromol/l	30 mg once daily if one or more of the following: CrCl 15–50 mL/min, weight ≤60 kg or concomitant use of potent P-gp inhibitor

Cmax maximum plasma concentration*; Cmin* minimum plasma concentration*; CrCl* Creatinine Clearance; *P-gp* P-glycoprotein.

* The best estimates of true trough levels of DOACs are those measured just before 12 or 24 hours after the last administered oral dose. We defined an appropriate interval as between 9-15 hours for twice daily DOACs and 21-27 hours for once daily DOACs in ≥90% of the studied patients. If unavailable, we approximated this proportion by using the measures of distribution of this time interval (e.g., mean and standard deviation, or median and interquartile interval).

† The best estimates of true peak levels of DOACs are those measured in accordance with the pharmacokinetic properties of the DOAC under study. We defined an appropriate interval as between 1-5 hours in ≥90% of patients. If unavailable, we approximated this proportion by using the measures of distribution of this time interval (e.g., mean and standard deviation, or median and interquartile interval). Because the time to maximum concentration of each DOAC is reported inconsistently between studies,[13,14] and because these properties are quite similar between DOACs, we decided to simplify by using the same interval for all DOACs.

Criteria and considerations for each quality assessment and the interpretation of level of evidence ratings

Table S5. Criteria and considerations for each quality assessment

Domains	Considerations	Criteria for judgement
Risk of bias	Threat to the validity of our inferences is greatest if studies are flawed in their design or conduct.	Studies were classified as at high, low, or unclear risk of bias according to criteria mentioned in the tailored QUADAS-2 tool (**Table S1-S3**).<br><br>We considered a study to be at high risk of bias if at least one signalling question is answered with ‘No’, indicating high risk of bias. Studies will be labelled as being at unclear risk of bias if none of the signalling question indicated high risk of bias, but at least one signalling question indicates unclear risk of bias.<br><br>For each outcome of interest, the level of evidence will be rated down by one grade if ≥25% of the included studies were at high risk of bias. We will also rate the level of evidence down by one grade if ≥50% of the included studies were at either high or unclear risk of bias.
Indirectness	Evidence is most certain when studies measured drug levels in the population of interest, that is, patients with atrial fibrillation treated with any of the four direct oral anticoagulants in doses approved for use in this indication. At the time of the measurements, patients should be at ‘steady state’ and representative of a typical population treated in the community.	Studies were classified as at high, low, or unclear concern of inapplicability to our review question according to criteria mentioned in the tailored QUADAS-2 tool (**Table S1-S3**).<br><br>We consider a study to be at high concern of inapplicability to our review question if at least one signalling question is answered with ‘No’, indicating high concern. Studies will be labelled as at unclear risk of concern if none of signalling question indicate high concern of inapplicability, but at least one signalling question indicates unclear concern.<br><br>For each outcome of interest, the level of evidence will be rated down by one grade if ≥25% of the included studies were at high risk of indirectness. We will also

Domains	Considerations	Criteria for judgement
		rate the level of evidence down by one grade if ≥50% of the included studies were at either high or unclear risk of indirectness.<br><br>However, to avoid penalizing for the same issue twice, we post-hoc decided to not rate down by one grade for indirectness if the outcome of interest was already downgraded due to high risk of bias. The rationale for this decision is that most studies at high risk of bias were given this label due to inappropriate inclusion or exclusion of special populations that could skew the drug level distribution. All such studies were then also labelled as at high concern of indirectness because the population was not sufficiently representative of the typical population.
Inconsistency	Certainty in a body of evidence is highest when there are multiple studies that show consistent effects.	In all analyses, limited consistency of point estimates or a lack of overlap of 95% confidence intervals, a two-tailed p-value of <0.10 of the X^2-test, and an I^2 of ≥50% were considered signals for substantial heterogeneity. If all these assessments were in agreement, evidence was graded down one level due to inconsistency.<br><br>If any of these assessments were in disagreement, results were discussed among two of the co-authors (TdV and NC) to arrive at a final decision to rate down the level of evidence one grade for inconsistency.
Imprecision	Precision of our summary estimates will be modest if the sum of the sample sizes of the included studies is small.	We rated down for imprecision by three levels if the total number of patients included in the analyses was ≤25, by two levels if this number was ≤50, and by one level if ≤250 patients are available. We upgraded the level of evidence by one level if the total number of patients included in an analysis was ≥1000.<br><br>Hence, to detect a d-type effect size of 0.8 (large effect), 0.5 (moderate effect), 0.2 (small effect), or 0.1 (very small effect) standard deviations, with an alpha of 5% and a power of 80% using a two-tailed one-sample Wilcoxon signed rank test and assuming a min ARE distribution, approximately 17, 39, 230, and 911 patients,

Domains	Considerations	Criteria for judgement
		respectively, are required. These calculations were performed with G*Power version 3.1.9.7.[15]<br><br>We decided to not consider the width of the 95% confidence intervals to rate down for imprecision because we wanted to avoid rating down for imprecision when the wide confidence intervals were driven by inconsistency in the point estimates of the included primary studies. We believed this to be appropriate because we (i) used random effects models to meta-analyse the available data, and (ii) we anticipated to include heterogenous populations.[16]
Publication bias	We decided not to perform an assessment to screen for potential publication bias because, for this type of meta-analysis, it is challenging to assess the degree of non-reporting bias.<br><br>First, because we anticipated it to be unlikely for entire reports, or particular results, of smaller studies to remain unpublished because of their findings on interpatient variation of DOAC drug levels [17].<br><br>Second, because we expected studies on patients with more extreme characteristics to be smaller in sample size than studies on more typical populations. Differences in outcomes of	In a post-hoc assessment, we visually assessed Fig S5 through S10 from Supporting Information File 4 and we observed no consistent relationship between study weight and the outcome for most plots. In a few plots, there were possible trends of systematically higher drug levels for the smaller studies, however, the populations had more extreme clinical characteristics compared to those from the other studies.[18-25] The latter was the most likely cause for the higher drug levels (rather than the inclusion of a biased sample of smaller studies).[17]

Domains	Considerations	Criteria for judgement
	interest between smaller and larger studies would therefore primarily be due to the differences between the populations and not due to publication bias.	

Table S6. Level of evidence ratings and their interpretation

Rating	Interpretation
High	We are confident that the true median, 10th or 90th percentile lies close to that of the corresponding pooled estimate.
Moderate	We are moderately confident that the true median, 10th or 90th percentile is likely to be close to the corresponding pooled estimate, but there is a possibility that they are substantially different.
Low	Our confidence in the pooled estimate is limited; the true median, 10th or 90th percentile may be substantially different from the estimate.
Very low	We have very little confidence in the estimate; the true median, 10th or 90th percentile is likely to be substantially different from the estimate.

Results of each quality assessment

Table S7. Final judgements of each domain of the risk of bias and indirectness assessments (using a tailored QUADAS-2 tool) of each primary study by direct oral anticoagulant type and dosing regimen

Study (year)[*]	Initial agreement or disagreement on all domains?	Risk of bias[†]			Indirectness[‡] (applicability concerns)
			Test		
		Patient selection	Trough	Peak	Patient selection
**Apixaban**					
2.5 mg twice daily					
Bánovčin Jr et al. (2017) [26]	Disagreement	?	☺	☺	☹
Bhagirath et al. (2017) [27]	Disagreement	?	?	NA	☹
Bhagirath et al. (2020) [28]	Disagreement	☹	☺	NA	☹
Bolek et al. (2019) [23]	Agreement	☹	☺	☺	☹
de Vries et al. (2022) [10]	Disagreement	☺	☺	☺	☹
Lin et al. (2020) [29]	Disagreement	☺	☺	☺	☹
Mavri et al. (2021) [30]	Disagreement	?	☺	☺	☺
Mukai et al. (2017) [31]	Disagreement	?	?	NA	☹
Nissan et al. (2019) [25]	Disagreement	☹	☺	☺	☹
Shin et al. (2018) [32]	Agreement	☹	☺	☺	☹
Skeppholm et al. (2015) [33]	Disagreement	?	☺	NA	?
Suwa et al. (2019) [34]	Agreement	☹	☺	☺	☹
Suzuki et al. (2020) [35]	Agreement	☹	☺	NA	☹
Takatsuki et al. (2017) [36]	Disagreement	?	☹	☹	☹
Testa et al. (2016) [11]	Disagreement	☹	☺	☺	☺
5 mg twice daily					
Bhagirath et al. (2017) [27]	Disagreement	?	?	NA	☹

Study (year)*	Initial agreement or disagreement on all domains?	Risk of bias†			Indirectness‡ (applicability concerns)
			Test		
		Patient selection	Trough	Peak	Patient selection
Bhagirath et al. (2020) [28]	Disagreement	☹	☺	NA	☹
Bolek et al. (2019) [23]	Agreement	☹	☺	☺	☹
de Vries et al. (2022) [10]	Disagreement	☺	☺	☺	☹
Harenberg et al. (2016) [37]	Disagreement	?	NA	☺	?
Lin et al. (2020) [29]	Disagreement	☺	☺	☺	☹
Mavri et al. (2021) [30]	Disagreement	?	☺	☺	☺
Mukai et al. (2017) [31]	Disagreement	?	?	NA	☹
Nissan et al. (2019) [25]	Disagreement	?	☺	☺	☹
Nosáľ et al. (2022) [38]	Agreement	☹	☺	☺	☹
Roşian et al. (2020) [39]	Disagreement	☹	☺	☺	☹
Samoš et al. (2018a) [24]	Disagreement	☹	☺	☺	☹
Shin et al. (2018) [32]	Agreement	☹	☺	☺	☹
Skeppholm et al. (2015) [33]	Disagreement	?	☺	NA	?
Suwa et al. (2019) [34]	Agreement	☹	☺	☺	☹
Suzuki et al. (2020) [35]	Agreement	☹	☺	NA	☹
Takatsuki et al. (2017) [36]	Disagreement	?	☹	☹	☹
Testa et al. (2016) [11]	Disagreement	☹	☺	☺	☺
**Dabigatran**					
<u>75 mg twice daily</u>					
Martin et al. (2018) [40]	Disagreement	?	☺	☺	☹
<u>110 mg twice daily</u>					
Bolek et al. (2018) [41]	Agreement	☹	☺	☺	☹
Bolek et al. (2021) [42]	Disagreement	☺	☺	?	☹
Chan et al. (2015) [9]	Agreement	☺	☺	☺	☺

Study (year)[*]	Initial agreement or disagreement on all domains?	Risk of bias[†]			Indirectness[‡] (applicability concerns)
			Test		
		Patient selection	Trough	Peak	Patient selection
Chang et al. (2016) [43]	Disagreement	☺	☺	NA	?
Chaussade et al. (2018) [44]	Agreement	☹	☺	☺	☹
Ji et al. (2020) [45]	Disagreement	☹	☺	☺	☹
Lin et al. (2019) [46]	Disagreement	☺	☺	☺	☹
Liu et al. (2020) [47]	Agreement	☹	☺	☺	☹
Mochalina et al. (2015) [48]	Agreement	☹	☺	NA	☹
Reilly et al. (2014) [49]	Agreement	☹	☺	☺	☹
Samoš et al. (2018a) [24]	Disagreement	☹	☺	☺	☹
Samoš et al. (2015) [20]	Disagreement	?	☺	☺	☹
Schnierer et al. (2020) [18]	Agreement	☺	☺	☺	☹
Šinigoj et al. (2015) [50]	Disagreement	?	☺	☺	☺
Skeppholm et al. (2014) [51]	Disagreement	?	☺	NA	☺
Skripka et al. (2020) [52]	Agreement	☹	?	NA	☹
Testa et al. (2016) [11]	Disagreement	☹	☺	☺	☺
Tomita et al. (2016) [53]	Disagreement	☹	☺	NA	☹
Zhang et al. (2018) [54]	Disagreement	?	☺	NA	☹
Zhu et al. (2021) [55]	Disagreement	☹	?	?	☹
<u>150 mg twice daily</u>					
Bolek et al. (2018) [41]	Agreement	☹	☺	☺	☹
Bolek et al. (2021) [42]	Disagreement	☺	☺	?	☹
Boonen et al. (2017) [56]	Disagreement	☹	☺	☺	☺
Chan et al. (2015) [9]	Agreement	☺	☺	☺	☺
Chang et al. (2016) [43]	Disagreement	☺	☺	NA	?
Harenberg et al. (2016) [37]	Disagreement	?	NA	☺	?
Lin et al. (2019) [46]	Disagreement	☺	☺	☺	☹
Nosáľ et al. (2022) [38]	Agreement	☹	☺	☺	☹

Study (year)*	Initial agreement or disagreement on all domains?	Risk of bias†			Indirectness‡ (applicability concerns)
			Test		
		Patient selection	Trough	Peak	Patient selection
Reilly et al. (2014) [49]	Agreement	☹	☺	☺	☹
Samoš et al. (2015) [20]	Disagreement	?	☺	☺	☹
Schnierer et al. (2020) [18]	Agreement	☺	☺	☺	☹
Silva et al. (2019) [57]	Disagreement	☺	NA	☺	☺
Šinigoj et al. (2015) [50]	Disagreement	?	☺	☺	☺
Skeppholm et al. (2014) [51]	Disagreement	?	☺	NA	☺
Taune et al. (2017) [58]	Disagreement	?	☺	☺	☹
Testa et al. (2016) [11]	Disagreement	☹	☺	☺	☺
Tomita et al. (2016) [53]	Disagreement	☹	☺	NA	☹
**Edoxaban**					
<u>15 mg once daily</u>					
Koretsune et al. (2015) [59]	Agreement	☹	☺	☺	☹
Ruff et al. (2015) [60]	Agreement	☹	☺	NA	☹
<u>30 mg once daily</u>					
Koretsune et al. (2015) [59]	Agreement	☹	☺	☺	☹
Ruff et al. (2015) [60]	Agreement	☹	☺	NA	☹
Testa et al. (2019) [61]	Disagreement	☺	☺	☺	☺
<u>60 mg once daily</u>					
Koretsune et al. (2015) [59]	Agreement	☹	☺	☺	☹
Ruff et al. (2015) [60]	Agreement	☹	☺	NA	☹
Testa et al. (2019) [61]	Disagreement	☺	☺	☺	☺
**Rivaroxaban**					
<u>10 mg once daily</u>					

Study (year)*	Initial agreement or disagreement on all domains?	Risk of bias†			Indirectness‡ (applicability concerns)
			Test		
		Patient selection	Trough	Peak	Patient selection
Hirota et al. (2020) [62]	Disagreement	☹	NA	☺	☹
Horinaka et al. (2018) [63]	Agreement	☺	☺	NA	☹
Nakagawa et al. (2021) [19]	Disagreement	?	☺	☺	☹
Suwa et al. (2019) [34]	Agreement	☹	☺	☺	☹
Takatsuki et al. (2017) [36]	Disagreement	?	☹	☹	☹
Wongcharoen et al. (2020) [22]	Agreement	?	☺	☺	☹
<u>15 mg once daily</u>					
Al-Aieshy et al. (2016) [64]	Disagreement	☹	☺	☺	☺
Bánovčin Jr et al. (2017) [26]	Disagreement	?	☺	☺	☹
Bolek et al. (2019) [23]	Agreement	☹	☺	☺	☹
Hirota et al. (2020) [62]	Disagreement	☹	NA	☺	☹
Horinaka et al. (2018) [63]	Agreement	☺	☺	NA	☹
Miklič et al. (2019) [65]	Agreement	☺	☺	☺	☺
Nakagawa et al. (2021) [19]	Disagreement	?	☺	☺	☹
Samoš et al. (2018a) [24]	Disagreement	☹	☺	☺	☹
Samoš et al. (2018b) [21]	Disagreement	☺	☺	☺	☹
Shyamkumar et al. (2021) [66]	Agreement	☺	☺	☺	☺
Suwa et al. (2019) [34]	Agreement	☹	☺	☺	☹
Takatsuki et al. (2017) [36]	Disagreement	?	☹	☹	☹
Testa et al. (2016) [11]	Disagreement	☹	☺	☺	☺
Wongcharoen et al. (2020) [22]	Agreement	?	☺	☺	☹
<u>20 mg once daily</u>					
Al-Aieshy et al. (2016) [64]	Disagreement	☹	☺	☺	☺
Bolek et al. (2019) [23]	Agreement	☹	☺	☺	☹
Harenberg et al. (2016) [37]	Disagreement	?	NA	☺	?

Study (year)*	Initial agreement or disagreement on all domains?	Risk of bias†			Indirectness‡ (applicability concerns)
			Test		
		Patient selection	Trough	Peak	Patient selection
Miklič et al. (2019) [65]	Agreement	☺	☺	☺	☺
Nosáľ et al. (2022) [38]	Agreement	☹	☺	☺	☹
Shyamkumar et al. (2021) [66]	Agreement	☺	☺	☺	☺
Silva et al. (2017) [67]	Disagreement	?	NA	☺	?
Silva et al. (2019) [57]	Disagreement	☺	NA	☺	☺
Testa et al. (2016) [11]	Disagreement	☹	☺	☺	☺
Wongcharoen et al. (2020) [22]	Agreement	?	☺	☺	☹

This table shows the final judgements of the risk of bias and indirectness assessments. We judged each study using the QUADAS-2 tool, tailored to our review question (Section: "Development of the quality assessment tool to assess the risk of bias and risk of indirectness of included studies").[1] If a primary study reported on multiple direct oral anticoagulants, we answered all signalling questions for each anticoagulant type separately (but not by dosing regimen). Half of all included studies were reviewed independently by two assessors and the other half by two different assessors. Any disagreement among one pair was solved by discussion between the paired reviewers. We had planned to involve one of the reviewers from the other pair to reach a final decision if consensus among the initial pair could not be reached, but this was not necessary for any of the studies. The judgement to reduce the level of evidence by one grade per outcome of interest due to risk of bias are summarized in **Table S8** and that to reduce the level of evidence due to indirectness in **Table S9**.

NA not applicable.

* Assessments were performed on study level but for each direct oral anticoagulant type independently; † We considered a study as at high risk of bias if at least one signalling question was answered with 'No', indicating high risk of bias. Studies were labelled as at unclear risk of bias if none of signalling question indicated high risk of bias, but at least one signalling question indicated unclear risk of bias; ‡ We considered a study as at high concern of inapplicability to our review question if at least one signalling question was answered with 'No', indicating high risk of concern. Studies were labelled as at unclear risk of concern if none of signalling question indicated high concern of inapplicability, but at least one signalling question indicated unclear concern.

☺ Low risk/concern; ☹ High risk/concern; ? Unclear risk/concern

Table S8. Results of the risk of bias assessment per outcome of interest

Trough levels

DOAC dosing regimen	No. of studies	No. (sum of weights) of studies at high risk of bias	No. (sum of weights) of studies at high or unclear risk of bias	Judgement
**Median trough drug level**				
**Apixaban**				
2.5 mg twice daily	17	10 (55%)	15 (85%)	Rated down one level
5 mg twice daily	19	11 (56%)	17 (88%)	Rated down one level
**Dabigatran**				
75 mg twice daily	1	0 (0%)	1 (100%)	Rated down one level
110 mg twice daily	26	15 (66%)	19 (80%)	Rated down one level
150 mg twice daily	18	7 (46%)	11 (68%)	Rated down one level
**Edoxaban**				
15 mg once daily	2	2 (100%)	2 (100%)	Rated down one level
30 mg once daily	4	3 (78%)	3 (78%)	Rated down one level
60 mg once daily	3	2 (70%)	2 (70%)	Rated down one level
**Rivaroxaban**				
10 mg once daily	4	1 (42%)	3 (81%)	Rated down one level
15 mg once daily	15	7 (52%)	11 (76%)	Rated down one level
20 mg once daily	8	5 (60%)	6 (72%)	Rated down one level
**10th percentile trough drug level**				
**Apixaban**				

DOAC dosing regimen	No. of studies	No. (sum of weights) of studies at high risk of bias	No. (sum of weights) of studies at high or unclear risk of bias	Judgement
2.5 mg twice daily	17	10 (60%)	15 (85%)	Rated down one level
5 mg twice daily	19	11 (53%)	17 (88%)	Rated down one level
**Dabigatran**				
75 mg twice daily	1	0 (0%)	1 (100%)	Rated down one level
110 mg twice daily	26	15 (62%)	19 (77%)	Rated down one level
150 mg twice daily	18	7 (46%)	11 (68%)	Rated down one level
**Edoxaban**				
15 mg once daily	2	2 (100%)	2 (100%)	Rated down one level
30 mg once daily	4	3 (84%)	3 (84%)	Rated down one level
60 mg once daily	3	2 (89%)	2 (89%)	Rated down one level
**Rivaroxaban**				
10 mg once daily	4	1 (51%)	3 83%)	Rated down one level
15 mg once daily	15	7 (47%)	11 (74%)	Rated down one level
20 mg once daily	8	5 (55%)	6 (69%)	Rated down one level
**90th percentile trough drug level**				
**Apixaban**				
2.5 mg twice daily	17	10 (56%)	15 (86%)	Rated down one level
5 mg twice daily	19	11 (55%)	17 (86%)	Rated down one level
**Dabigatran**				
75 mg twice daily	1	0 (0%)	1 (100%)	Rated down one level
110 mg twice daily	26	15 (68%)	19 (81%)	Rated down one level
150 mg twice daily	18	7 (47%)	11 (72%)	Rated down one level
**Edoxaban**				

DOAC dosing regimen	No. of studies	No. (sum of weights) of studies at high risk of bias	No. (sum of weights) of studies at high or unclear risk of bias	Judgement
15 mg once daily	2	2 (100%)	2 (100%)	Rated down one level
30 mg once daily	4	3 (79%)	3 (79%)	Rated down one level
60 mg once daily	3	2 (69%)	2 (69%)	Rated down one level
**Rivaroxaban**				
10 mg once daily	4	1 (39%)	3 (80%)	Rated down one level
15 mg once daily	15	7 (53%)	11 (73%)	Rated down one level
20 mg once daily	8	5 (51%)	6 (65%)	Rated down one level

This table summarizes our results of the risk of bias assessment. The results of the risk of bias assessment per study are presented in **Table S7**. The criteria and considerations to rate down for risk of bias are presented in **Table S5**.

* If studies reported on multiple subgroups of participants on the same DOAC type and dosing regimen, those studies were considered unique studies.

Peak levels

DOAC dosing regimen	No. of studies	No. (sum of weights) of studies at high risk of bias	No. (sum of weights) of studies at high or unclear risk of bias	Judgement
**Median peak drug level**				
**Apixaban**				
2.5 mg twice daily	12	7 (55%)	10 (72%)	Rated down one level
5 mg twice daily	16	9 (61%)	14 (86%)	Rated down one level
**Dabigatran**				
75 mg twice daily	1	0 (0%)	1 (100%)	Rated down one level
110 mg twice daily	18	10 (64%)	14 (83%)	Rated down one level
150 mg twice daily	17	7 (45%)	12 (68%)	Rated down one level
**Edoxaban**				
15 mg once daily	1	1 (100%)	1 (100%)	Rated down one level
30 mg once daily	2	1 (49%)	1 (49%)	Rated down one level
60 mg once daily	2	1 (45%)	1 (45%)	Rated down one level
**Rivaroxaban**				
10 mg once daily	4	2 (82%)	4 (100%)	Rated down one level
15 mg once daily	14	7 (53%)	11 (80%)	Rated down one level
20 mg once daily	10	4 (43%)	7 (68%)	Rated down one level
**10th percentile peak drug level**				
**Apixaban**				
2.5 mg twice daily	12	7 (57%)	10 (78%)	Rated down one level
5 mg twice daily	16	9 (58%)	14 (87%)	Rated down one level

DOAC dosing regimen	No. of studies	No. (sum of weights) of studies at high risk of bias	No. (sum of weights) of studies at high or unclear risk of bias	Judgement
**Dabigatran**				
75 mg twice daily	1	0 (0%)	1 (100%)	Rated down one level
110 mg twice daily	18	10 (60%)	14 (81%)	Rated down one level
150 mg twice daily	17	7 (45%)	12 (70%)	Rated down one level
**Edoxaban**				
15 mg once daily	1	1 (100%)	1 (100%)	Rated down one level
30 mg once daily	2	1 (51%)	1 (51%)	Rated down one level
60 mg once daily	2	1 (49%)	1 (49%)	Rated down one level
**Rivaroxaban**				
10 mg once daily	4	2 (60%)	4 (100%)	Rated down one level
15 mg once daily	14	7 (51%)	11 (79%)	Rated down one level
20 mg once daily	10	4 (41%)	7 (65%)	Rated down one level
**90th percentile peak drug level**				
**Apixaban**				
2.5 mg twice daily	12	7 (55%)	10 (75%)	Rated down one level
5 mg twice daily	16	9 (67%)	14 (84%)	Rated down one level
**Dabigatran**				
75 mg twice daily	1	0 (0%)	1 (100%)	Rated down one level
110 mg twice daily	18	10 (64%)	14 (83%)	Rated down one level
150 mg twice daily	17	7 (44%)	12 (68%)	Rated down one level
**Edoxaban**				
15 mg once daily	1	1 (100%)	1 (100%)	Rated down one level
30 mg once daily	2	1 (51%)	1 (51%)	Rated down one level

DOAC dosing regimen	No. of studies	No. (sum of weights) of studies at high risk of bias	No. (sum of weights) of studies at high or unclear risk of bias	Judgement
60 mg once daily	2	1 (44%)	1 (44%)	Rated down one level
**Rivaroxaban**				
10 mg once daily	4	2 (79%)	4 (100%)	Rated down one level
15 mg once daily	14	7 (55%)	11 (79%)	Rated down one level
20 mg once daily	10	4 (41%)	7 (69%)	Rated down one level

This table summarizes our results of the risk of bias assessment. The results of the risk of bias assessment per study are presented in **Table S7**. The criteria and considerations to rate down for risk of bias are presented in **Table S5**.

* If studies reported on multiple subgroups of participants on the same DOAC type and dosing regimen, those studies were considered unique studies.

Table S9. Results of indirectness assessment per outcome of interest

Trough levels

**DOAC dosing regimen**	**No. of studies**	**No. (sum of weights) of studies at high concern of inapplicability**	**No. (sum of weights) of studies at high or unclear concern of inapplicability**	**Judgement**
**Median trough drug level**				
**Apixaban**				
2.5 mg twice daily	17	15 (87%)	16 (93%)	Not rated down†
5 mg twice daily	19	17 (89%)	17 (89%)	Not rated down†
**Dabigatran**				
75 mg twice daily	1	1 (100%)	1 (100%)	Not rated down†
110 mg twice daily	26	21 (83%)	22 (88%)	Not rated down†
150 mg twice daily	18	11 (54%)	12 (59%)	Not rated down†
**Edoxaban**				
15 mg once daily	2	2 (100%)	2 (100%)	Not rated down†
30 mg once daily	4	3 (78%)	3 (78%)	Not rated down†
60 mg once daily	3	2 (70%)	2 (70%)	Not rated down†
**Rivaroxaban**				
10 mg once daily	4	4 (100%)	4 (100%)	Not rated down†
15 mg once daily	15	11 (76%)	11 (76%)	Not rated down†
20 mg once daily	8	4 (30%)	4 (30%)	Not rated down†
**10th percentile trough drug level**				
**Apixaban**				

DOAC dosing regimen	No. of studies	No. (sum of weights) of studies at high concern of inapplicability	No. (sum of weights) of studies at high or unclear concern of inapplicability	Judgement
2.5 mg twice daily	17	15 (89%)	16 (93%)	Not rated down†
5 mg twice daily	19	17 (89%)	17 (89%)	Not rated down†
**Dabigatran**				
75 mg twice daily	1	1 (100%)	1 (100%)	Not rated down†
110 mg twice daily	26	21 (81%)	22 (86%)	Not rated down†
150 mg twice daily	18	11 (54%)	12 (59%)	Not rated down†
**Edoxaban**				
15 mg once daily	2	2 (100%)	2 (100%)	Not rated down†
30 mg once daily	4	3 (84%)	3 (84%)	Not rated down†
60 mg once daily	3	2 (89%)	2 (89%)	Not rated down†
**Rivaroxaban**				
10 mg once daily	4	4 (100%)	4 (100%)	Not rated down†
15 mg once daily	15	11 (74%)	11 (74%)	Not rated down†
20 mg once daily	8	4 (36%)	4 (36%)	Not rated down†
**90th percentile trough drug level**				
**Apixaban**				
2.5 mg twice daily	17	15 (86%)	16 (94%)	Not rated down†
5 mg twice daily	19	17 (88%)	17 (88%)	Not rated down†
**Dabigatran**				
75 mg twice daily	1	1 (100%)	1 (100%)	Not rated down†
110 mg twice daily	26	21 (85%)	22 (89%)	Not rated down†
150 mg twice daily	18	11 (54%)	12 (57%)	Not rated down†
**Edoxaban**				

DOAC dosing regimen	No. of studies	No. (sum of weights) of studies at high concern of inapplicability	No. (sum of weights) of studies at high or unclear concern of inapplicability	Judgement
15 mg once daily	2	2 (100%)	2 (100%)	Not rated down†
30 mg once daily	4	3 (79%)	3 (79%)	Not rated down†
60 mg once daily	3	2 (69%)	2 (69%)	Not rated down†
**Rivaroxaban**				
10 mg once daily	4	4 (100%)	4 (100%)	Not rated down†
15 mg once daily	15	11 (69%)	11 (69%)	Not rated down†
20 mg once daily	8	4 (25%)	4 (25%)	Not rated down†

This table summarizes our results of the risk of bias assessment. The results of the risk of bias assessment per study are presented in **Table S7**. The criteria and considerations to rate down for risk of bias are presented in **Table S5**.

* If studies reported on multiple subgroups of participants on the same DOAC type and dosing regimen, those studies were considered unique studies; † to avoid penalizing for the same issue twice, we post-hoc decided to not rate down by one grade for indirectness if the outcome of interest was already downgraded due to high risk of bias. The rationale for this decision is that most studies at high risk of bias were given this label due to inappropriate inclusion or exclusion of special populations that could skew the drug level distribution. All such studies were then also labelled as at high concern of indirectness because the population was not sufficiently representative of the typical population.

Peak levels

DOAC dosing regimen	No. of studies	No. (sum of weights) of studies at high concern of inapplicability	No. (sum of weights) of studies at high or unclear concern of inapplicability	Judgement
**Median peak drug level**				
**Apixaban**				
2.5 mg twice daily	12	11 (89%)	11 (89%)	Not rated down†
5 mg twice daily	16	13 (84%)	14 (86%)	Not rated down†
**Dabigatran**				
75 mg twice daily	1	1 (100%)	1 (100%)	Not rated down†
110 mg twice daily	18	15 (86%)	15 (86%)	Not rated down†
150 mg twice daily	17	10 (61%)	11 (62%)	Not rated down†
**Edoxaban**				
15 mg once daily	1	1 (100%)	1 (100%)	Not rated down†
30 mg once daily	2	1 (49%)	1 (49%)	Not rated down†
60 mg once daily	2	1 (45%)	1 (45%)	Not rated down†
**Rivaroxaban**				
10 mg once daily	4	4 (100%)	4 (100%)	Not rated down†
15 mg once daily	14	11 (79%)	11 (79%)	Not rated down†
20 mg once daily	10	4 (41%)	6 (57%)	Not rated down†
**10th percentile peak drug level**				
**Apixaban**				
2.5 mg twice daily	12	11 (92%)	11 (92%)	Not rated down†
5 mg twice daily	16	13 (83%)	14 (87%)	Not rated down†

DOAC dosing regimen	No. of studies	No. (sum of weights) of studies at high concern of inapplicability	No. (sum of weights) of studies at high or unclear concern of inapplicability	Judgement
**Dabigatran**				
75 mg twice daily	1	1 (100%)	1 (100%)	Not rated down†
110 mg twice daily	18	15 (84%)	15 (84%)	Not rated down†
150 mg twice daily	17	10 (60%)	11 (63%)	Not rated down†
**Edoxaban**				
15 mg once daily	1	1 (100%)	1 (100%)	Not rated down†
30 mg once daily	2	1 (51%)	1 (51%)	Not rated down†
60 mg once daily	2	1 (49%)	1 (49%)	Not rated down†
**Rivaroxaban**				
10 mg once daily	4	4 (100%)	4 (100%)	Not rated down†
15 mg once daily	14	11 (79%)	11 (79%)	Not rated down†
20 mg once daily	10	4 (37%)	6 (55%)	Not rated down†
**90th percentile peak drug level**				
**Apixaban**				
2.5 mg twice daily	12	11 (88%)	11 (88%)	Not rated down†
5 mg twice daily	16	13 (83%)	14 (86%)	Not rated down†
**Dabigatran**				
75 mg twice daily	1	1 (100%)	1 (100%)	Not rated down†
110 mg twice daily	18	15 (85%)	15 (85%)	Not rated down†
150 mg twice daily	17	10 (59%)	11 (61%)	Not rated down†
**Edoxaban**				
15 mg once daily	1	1 (100%)	1 (100%)	Not rated down†
30 mg once daily	2	1 (51%)	1 (51%)	Not rated down†

DOAC dosing regimen	No. of studies	No. (sum of weights) of studies at high concern of inapplicability	No. (sum of weights) of studies at high or unclear concern of inapplicability	Judgement
60 mg once daily	2	1 (44%)	1 (44%)	Not rated down†
**Rivaroxaban**				
10 mg once daily	4	4 (100%)	4 (100%)	Not rated down†
15 mg once daily	14	11 (72%)	11 (72%)	Not rated down†
20 mg once daily	10	4 (39%)	6 (58%)	Not rated down†

This table summarizes our results of the risk of bias assessment. The results of the risk of bias assessment per study are presented in **Table S7**. The criteria and considerations to rate down for risk of bias are presented in **Table S5**.

* If studies reported on multiple subgroups of participants on the same DOAC type and dosing regimen, those studies were considered unique studies; † to avoid penalizing for the same issue twice, we post-hoc decided to not rate down by one grade for indirectness if the outcome of interest was already downgraded due to high risk of bias. The rationale for this decision is that most studies at high risk of bias were given this label due to inappropriate inclusion or exclusion of special populations that could skew the drug level distribution. All such studies were then also labelled as at high concern of indirectness because the population was not sufficiently representative of the typical population.

Table S10. Results of the inconsistency assessment of each outcome of interest

A. Trough levels

DOAC dosing regimen	Visual assessment*	p-value of X^2-test	I^2 value	Discordance among the three assessments?†	Judgement†
**Median trough drug level**					
**Apixaban**					
2.5 mg twice daily	Serious	<0.0001	73.1%	No	Rated down one level
5 mg twice daily	Critical	<0.0001	90.3%	No	Rated down one level
**Dabigatran**					
75 mg twice daily‡	NA	NA	NA	NA	NA
110 mg twice daily	Serious	<0.0001	94.0%	No	Rated down one level
150 mg twice daily	Serious	<0.0001	79.2%	No	Rated down one level
**Edoxaban**					
15 mg once daily	Moderate	0.0659	70.4%	No	Rated down one level
30 mg once daily	Critical	<0.0001	99.2%	No	Rated down one level
60 mg once daily	Serious	<0.0001	96.2%	No	Rated down one level
**Rivaroxaban**					
10 mg once daily	Some	0.1082	43.7%	No	Not rated down
15 mg once daily	Serious	<0.0001	76.7%	No	Rated down one level
20 mg once daily	Some	0.3168	29.2%	No	Not rated down
**10th percentile trough drug level**					
**Apixaban**					
2.5 mg twice daily	Serious	<0.0001	76.6%	No	Rated down one level

DOAC dosing regimen	Visual assessment*	p-value of X^2-test	I^2 value	Discordance among the three assessments?†	Judgement†
5 mg twice daily	Critical	<0.0001	93.5%	No	Rated down one level
**Dabigatran**					
75 mg twice daily‡	NA	NA	NA	NA	NA
110 mg twice daily	Critical	<0.0001	96.2%	No	Rated down one level
150 mg twice daily	Serious	<0.0001	77.3%	No	Rated down one level
**Edoxaban**					
15 mg once daily	Serious	0.0832	66.7%	No	Rated down one level
30 mg once daily	Critical	<0.0001	96.3%	No	Rated down one level
60 mg once daily	Some	0.2126	26.0%	No	Not rated down
**Rivaroxaban**					
10 mg once daily	Some	0.0647	18.2%	Yes	Not rated down§
15 mg once daily	Serious	<0.0001	75.1%	No	Rated down one level
20 mg once daily	Serious	0.0184	55.7%	No	Rated down one level
**90th percentile trough drug level**					
**Apixaban**					
2.5 mg twice daily	Serious	<0.0001	80.1%	No	Rated down one level
5 mg twice daily	Critical	<0.0001	93.7%	No	Rated down one level
**Dabigatran**					
75 mg twice daily‡	NA	NA	NA	NA	NA
110 mg twice daily	Serious	<0.0001	95.6%	No	Rated down one level
150 mg twice daily	Serious	<0.0001	80.3%	No	Rated down one level
**Edoxaban**					
15 mg once daily	Some	0.2384	28.0%	No	Not rated down
30 mg once daily	Critical	<0.0001	99.4%	No	Rated down one level

DOAC dosing regimen	Visual assessment*	p-value of X^2-test	I^2 value	Discordance among the three assessments?†	Judgement†
60 mg once daily	Critical	<0.0001	99.6%	No	Rated down one level
**Rivaroxaban**					
10 mg once daily	Near none	0.8367	0.0%	No	Not rated down
15 mg once daily	Serious	<0.0001	85.4%	No	Rated down one level
20 mg once daily	Some	0.5278	14.4%	No	Not rated down

This table summarizes our results of the inconsistency assessment for all analyses on trough levels. The criteria and considerations to rate down for risk of bias are presented in **Table S5**.

* Visual assessment of the consistency of point estimates and overlap in the 95% confidence intervals per analysis was done by a single author (TdV); † If any of these assessments were in disagreement, the findings were discussed among two of the authors (TdV and NC) to arrive at a final judgement to rate down the level of evidence one grade for inconsistency; ‡ Not applicable because it concerns only a singular study; § We did not rate down for inconsistency because of (i) the inconsistency is small in absolute terms, and (ii) the low weight (i.e., 3.0%) of the study with the inconsistent results.

B. Peak levels

DOAC dosing regimen	Visual assessment*	p-value of X^2-test	I^2 value	Discordance among the three assessments?†	Judgement†
**Median peak drug level**					
**Apixaban**					
2.5 mg twice daily	Near none	0.5673	0.0%	No	Not rated down
5 mg twice daily	Serious	<0.0001	82.1%	No	Rated down one level
**Dabigatran**					
75 mg twice daily‡	NA	NA	NA	NA	NA
110 mg twice daily	Critical	<0.0001	98.2%	No	Rated down one level
150 mg twice daily	Serious	<0.0001	85.3%	No	Rated down one level
**Edoxaban**					
15 mg once daily‡	NA	NA	NA	NA	NA
30 mg once daily	Critical	0.0005	91.7%	No	Rated down one level
60 mg once daily	Serious	0.0118	84.2%	No	Rated down one level
**Rivaroxaban**					
10 mg once daily	Near none	0.3019	0.0%	No	Not rated down
15 mg once daily	Serious	<0.0001	90.5%	No	Rated down one level
20 mg once daily	Critical	<0.0001	99.6%	No	Rated down one level
**10th percentile peak drug level**					
**Apixaban**					
2.5 mg twice daily	Serious	0.0036	61.8%	No	Rated down one level
5 mg twice daily	Critical	<0.0001	88.4%	No	Rated down one level
**Dabigatran**					

DOAC dosing regimen	Visual assessment*	p-value of X^2-test	I^2 value	Discordance among the three assessments?†	Judgement†
75 mg twice daily‡	NA	NA	NA	NA	NA
110 mg twice daily	Critical	<0.0001	97.6%	No	Rated down one level
150 mg twice daily	Serious	<0.0001	85.1%	No	Rated down one level
**Edoxaban**					
15 mg once daily‡	NA	NA	NA	NA	NA
30 mg once daily	Serious	0.0008	91.0%	No	Rated down one level
60 mg once daily	Serious	0.0013	90.3%	No	Rated down one level
**Rivaroxaban**					
10 mg once daily	Moderate	0.0228	69.5%	No	Rated down one level
15 mg once daily	Critical	<0.0001	91.9%	No	Rated down one level
20 mg once daily	Serious	<0.0001	98.8%	No	Rated down one level
**90th percentile peak drug level**					
**Apixaban**					
2.5 mg twice daily	Some	0.6617	8.3%	No	Not rated down
5 mg twice daily	Moderate	0.0143	55.4%	No	Rated down one level
**Dabigatran**					
75 mg twice daily‡	NA	NA	NA	NA	NA
110 mg twice daily	Critical	<0.0001	98.6%	No	Rated down one level
150 mg twice daily	Serious	<0.0001	83.2%	No	Rated down one level
**Edoxaban**					
15 mg once daily‡	NA	NA	NA	NA	NA
30 mg once daily	Serious	0.0013	90.4%	No	Rated down one level
60 mg once daily	Moderate	0.0827	66.8%	No	Rated down one level
**Rivaroxaban**					

DOAC dosing regimen	Visual assessment[*]	p-value of X^2-test	I^2 value	Discordance among the three assessments?[†]	Judgement[†]
10 mg once daily	Near none	0.5154	13.0%	No	Not rated down
15 mg once daily	Serious	<0.0001	76.7%	No	Rated down one level
20 mg once daily	Critical	<0.0001	99.6%	No	Rated down one level

This table summarizes our results of the inconsistency assessment for all analyses on peak levels. The criteria and considerations to rate down for risk of bias are presented in **Table S5**.

[*] Visual assessment of the consistency of point estimates and overlap in the 95% confidence intervals per analysis was done by a single author (TdV); [†] If any of these assessments were in disagreement, the findings were discussed among two of the authors (TdV and NC) to arrive at a final judgement to rate down the level of evidence one grade for inconsistency; [‡] Not applicable because it concerns only a singular study.

Table S11. Results of the imprecision assessment per outcome of interest

DOAC dosing regimen	Number of patients	Judgement
**Trough levels (median, 10th percentile, and 90th percentile)***		
**Apixaban**		
2.5 mg twice daily	1,011	Rated up one level
5 mg twice daily	3,226	Rated up one level
**Dabigatran**		
75 mg twice daily†	60	Rated down one level
110 mg twice daily	5,303	Rated up one level
150 mg twice daily	4,693	Rated up one level
**Edoxaban**		
15 mg once daily	1,824	Rated up one level
30 mg once daily	7,107	Rated up one level
60 mg once daily	5,318	Rated up one level
**Rivaroxaban**		
10 mg once daily	83	Rated down one level
15 mg once daily	425	Not rated down
20 mg once daily	216	Rated down one level
**Peak levels (median, 10th percentile, and 90th percentile)***		
**Apixaban**		
2.5 mg twice daily	299	Not rated down
5 mg twice daily	446	Not rated down
**Dabigatran**		
75 mg twice daily†	60	Rated down one grade
110 mg twice daily	5,154	Rated up one level
150 mg twice daily	4,996	Rated up one level
**Edoxaban**		
15 mg once daily†	39	Rated down two grades
30 mg once daily	74	Rated down one grade
60 mg once daily	67	Rated down one grade
**Rivaroxaban**		
10 mg once daily	137	Rated down one grade
15 mg once daily	470	Not rated down
20 mg once daily	361	Not rated down

This table summarizes our results of the imprecision assessment per outcome of interest on trough levels. The criteria and considerations to rate down the level of evidence for imprecision are shown in **Table S5**.

* Identical judgements were given to each outcome of interest (i.e., median, 10th percentile, 90th percentile) per DOAC dosing regimen as the same number of patients were included in these models; † Concerns only a single study.

Level of evidence profiles using the GRADE criteria for all outcomes of interest

Table S12. GRADE evidence profile: median and 10th to 90th percentile interval of direct oral anticoagulant drug levels in patients with atrial fibrillation

A. Trough levels

	Quality assessments					
**Outcomes**	**Risk of bias**	**Inconsistency**	**Imprecision**	**Indirectness**	**Publication bias***	**Certainty of the evidence†**
**Median trough drug level**						
**Apixaban**						
2.5 mg twice daily	-1	-1	+1	0§	NA	Moderate
5 mg twice daily	-1	-1	+1	0§	NA	Moderate
**Dabigatran**						
75 mg twice daily‡	-1	NA	-1	0§	NA	Low
110 mg twice daily	-1	-1	+1	0§	NA	Moderate
150 mg twice daily	-1	-1	+1	0§	NA	Moderate
**Edoxaban**						
15 mg once daily	-1	-1	+1	0§	NA	Moderate
30 mg once daily	-1	-1	+1	0§	NA	Moderate
60 mg once daily	-1	-1	+1	0§	NA	Moderate
**Rivaroxaban**						
10 mg once daily	-1	0	-1	0§	NA	Low
15 mg once daily	-1	-1	0	0§	NA	Low
20 mg once daily	-1	0	-1	0§	NA	Low
**10th percentile trough drug level**						

	Quality assessments					
Outcomes	Risk of bias	Inconsistency	Imprecision	Indirectness	Publication bias*	Certainty of the evidence†
**Apixaban**						
2.5 mg twice daily	-1	-1	+1	0§	NA	Moderate
5 mg twice daily	-1	-1	+1	0§	NA	Moderate
**Dabigatran**						
75 mg twice daily‡	-1	NA	-1	0§	NA	Low
110 mg twice daily	-1	-1	+1	0§	NA	Moderate
150 mg twice daily	-1	-1	+1	0§	NA	Moderate
**Edoxaban**						
15 mg once daily	-1	-1	+1	0§	NA	Moderate
30 mg once daily	-1	-1	+1	0§	NA	Moderate
60 mg once daily	-1	0	+1	0§	NA	High
**Rivaroxaban**						
10 mg once daily	-1	0	-1	0§	NA	Low
15 mg once daily	-1	-1	0	0§	NA	Low
20 mg once daily	-1	-1	-1	0§	NA	Very low
**90th percentile trough drug level**						
**Apixaban**						
2.5 mg twice daily	-1	-1	+1	0§	NA	Moderate
5 mg twice daily	-1	-1	+1	0§	NA	Moderate
**Dabigatran**						
75 mg twice daily‡	-1	NA	-1	0§	NA	Low
110 mg twice daily	-1	-1	+1	0§	NA	Moderate
150 mg twice daily	-1	-1	+1	0§	NA	Moderate
**Edoxaban**						
15 mg once daily	-1	0	+1	0§	NA	High

	Quality assessments					
**Outcomes**	**Risk of bias**	**Inconsistency**	**Imprecision**	**Indirectness**	**Publication bias***	**Certainty of the evidence**†
30 mg once daily	-1	-1	+1	0§	NA	Moderate
60 mg once daily	-1	-1	+1	0§	NA	Moderate
**Rivaroxaban**						
10 mg once daily	-1	0	-1	0§	NA	Low
15 mg once daily	-1	-1	0	0§	NA	Low
20 mg once daily	-1	0	-1	0§	NA	Low

In this table the final judgements of all quality assessments (i.e., risk of bias, indirectness, imprecision, inconsistency, and publication bias) are presented together with the level of evidence GRADE for trough levels.[68] The considerations and criteria used to arrive at a decision to (not) rate down the level of evidence are presented in **Table S5**. The interpretation of the level of evidence ratings are presented in **Table S6**. The number indicate how many levels were graded down per quality assessment (e.g., 0 equals "not rated down", -1 equals "rated down by one grade").

NA not applicable.

* We did not perform assessment to detect potential publication bias (**Table S5**); † All outcomes of interest started at high quality of evidence; ‡ Concerns only a single study; § To avoid penalizing for the same issue twice, we post-hoc decided to not rate down by one grade for indirectness if the outcome of interest was already downgraded due to high risk of bias. The rationale for this decision is that most studies at high risk of bias were given this label due to inappropriate inclusion or exclusion of special populations that could skew the drug level distribution. All such studies were then also labelled as at high concern of indirectness because the population was not sufficiently representative of the typical population.

A. Peak levels

Outcomes	Quality assessments					Certainty of the evidence[†]
	Risk of bias	Inconsistency	Imprecision	Indirectness	Publication bias[*]	
**Median peak drug level**						
**Apixaban**						
2.5 mg twice daily	-1	0	0	0[§]	NA	Moderate
5 mg twice daily	-1	-1	0	0[§]	NA	Low
**Dabigatran**						
75 mg twice daily[‡]	-1	NA	-1	0[§]	NA	Low
110 mg twice daily	-1	-1	+1	0[§]	NA	Moderate
150 mg twice daily	-1	-1	+1	0[§]	NA	Moderate
**Edoxaban**						
15 mg once daily[‡]	-1	NA	-2	0[§]	NA	Very low
30 mg once daily	-1	-1	-1	0[§]	NA	Very low
60 mg once daily	-1	-1	-1	0[§]	NA	Very low
**Rivaroxaban**						
10 mg once daily	-1	0	-1	0[§]	NA	Low
15 mg once daily	-1	-1	0	0[§]	NA	Low
20 mg once daily	-1	-1	0	0[§]	NA	Low
**10th percentile peak drug level**						
**Apixaban**						
2.5 mg twice daily	-1	-1	0	0[§]	NA	Low
5 mg twice daily	-1	-1	0	0[§]	NA	Low
**Dabigatran**						
75 mg twice daily[‡]	-1	NA	-1	0[§]	NA	Low
110 mg twice daily	-1	-1	+1	0[§]	NA	Moderate

	Quality assessments					
Outcomes	Risk of bias	Inconsistency	Imprecision	Indirectness	Publication bias*	Certainty of the evidence†
150 mg twice daily	-1	-1	+1	0§	NA	Moderate
**Edoxaban**						
15 mg once daily‡	-1	NA	-2	0§	NA	Very low
30 mg once daily	-1	-1	-1	0§	NA	Very low
60 mg once daily	-1	-1	-1	0§	NA	Very low
**Rivaroxaban**						
10 mg once daily	-1	-1	-1	0§	NA	Very low
15 mg once daily	-1	-1	0	0§	NA	Low
20 mg once daily	-1	-1	0	0§	NA	Low
**90th percentile peak drug level**						
**Apixaban**						
2.5 mg twice daily	-1	0	0	0§	NA	Moderate
5 mg twice daily	-1	-1	0	0§	NA	Low
**Dabigatran**						
75 mg twice daily‡	-1	NA	-1	0§	NA	Low
110 mg twice daily	-1	-1	+1	0§	NA	Moderate
150 mg twice daily	-1	-1	+1	0§	NA	Moderate
**Edoxaban**						
15 mg once daily‡	-1	NA	-2	0§	NA	Very low
30 mg once daily	-1	-1	-1	0§	NA	Very low
60 mg once daily	-1	-1	-1	0§	NA	Very low
**Rivaroxaban**						
10 mg once daily	-1	0	-1	0§	NA	Low
15 mg once daily	-1	-1	0	0§	NA	Low
20 mg once daily	-1	-1	0	0§	NA	Low

In this table the final judgements of all quality assessments (i.e., risk of bias, indirectness, imprecision, inconsistency, and publication bias) are presented together with the level of evidence GRADE for peak levels.[68] The considerations and criteria used to arrive at a decision to (not) rate down the level of evidence are presented in **Table S5**. The interpretation of the level of evidence ratings are presented in **Table S6**. The number indicate how many levels were graded down per quality assessment (e.g., 0 equals “not rated down”, -1 equals “rated down by one grade”).

NA not applicable.

[*] We did not perform assessment to detect potential publication bias (**Table S5**); [†] All outcomes of interest started at high quality of evidence; [‡] Concerns only a single study; [§] To avoid penalizing for the same issue twice, we post-hoc decided to not rate down by one grade for indirectness if the outcome of interest was already downgraded due to high risk of bias. The rationale for this decision is that most studies at high risk of bias were given this label due to inappropriate inclusion or exclusion of special populations that could skew the drug level distribution. All such studies were then also labelled as at high concern of indirectness because the population was not sufficiently representative of the typical population.

References

1. Whiting PF, Rutjes AW, Westwood ME*, et al.* QUADAS-2: a revised tool for the quality assessment of diagnostic accuracy studies. *Ann Intern Med*. 2011;155(8):529-536
2. Sterne JA, Hernán MA, Reeves BC*, et al.* ROBINS-I: a tool for assessing risk of bias in non-randomised studies of interventions. *Bmj*. 2016;355:i4919
3. Desmaele S, Steurbaut S, Cornu P, Brouns R, Dupont AG. Clinical trials with direct oral anticoagulants for stroke prevention in atrial fibrillation: how representative are they for real life patients? *Eur J Clin Pharmacol*. 2016;72(9):1125-1134
4. Fanning L, Ilomäki J, Bell JS, Dārziņš P. The representativeness of direct oral anticoagulant clinical trials to hospitalized patients with atrial fibrillation. *Eur J Clin Pharmacol*. 2017;73(11):1427-1436
5. Hirsh Raccah B, Rottenstreich A, Zacks N*, et al.* Appropriateness of direct oral anticoagulant dosing and its relation to drug levels in atrial fibrillation patients. *J Thromb Thrombolysis*. 2019;47(4):550-557
6. Van Spall HG, Toren A, Kiss A, Fowler RA. Eligibility criteria of randomized controlled trials published in high-impact general medical journals: a systematic sampling review. *Jama*. 2007;297(11):1233-1240
7. Yoon CH, Park YK, Kim SJ*, et al.* Eligibility and preference of new oral anticoagulants in patients with atrial fibrillation: comparison between patients with versus without stroke. 2014;45(10):2983-2988
8. Rothwell PM. Factors That Can Affect the External Validity of Randomised Controlled Trials. *PLOS Clinical Trials*. 2006;1(1):e9
9. Chan NC, Coppens M, Hirsh J*, et al.* Real-world variability in dabigatran levels in patients with atrial fibrillation. *J Thromb Haemost*. 2015;13(3):353-359
10. de Vries TAC, Hirsh J, Bhagirath VC*, et al.* Can a Single Measurement of Apixaban Levels Identify Patients at Risk of Overexposure? A Prospective Cohort Study. *TH Open*. 2022;06(01):e10-e17
11. Testa S, Tripodi A, Legnani C*, et al.* Plasma levels of direct oral anticoagulants in real life patients with atrial fibrillation: Results observed in four anticoagulation clinics. *Thromb Res*. 2016;137:178-183
12. Bhaskaran K, Smeeth L. What is the difference between missing completely at random and missing at random? *Int J Epidemiol*. 2014;43(4):1336-1339
13. Steffel J, Collins R, Antz M*, et al.* 2021 European Heart Rhythm Association Practical Guide on the Use of Non-Vitamin K Antagonist Oral Anticoagulants in Patients with Atrial Fibrillation. *Europace*. 2021;23(10):1612-1676

14. Chan NC, Eikelboom JW, Weitz JI. Evolving Treatments for Arterial and Venous Thrombosis: Role of the Direct Oral Anticoagulants. *Circ Res*. 2016;118(9):1409-1424
15. Faul F, Erdfelder E, Lang A-G, Buchner A. G*Power 3: A flexible statistical power analysis program for the social, behavioral, and biomedical sciences. *Behavior Research Methods*. 2007;39(2):175-191
16. Schünemann HJ, Neumann I, Hultcrantz M*, et al.* GRADE guidance 35: update on rating imprecision for assessing contextualized certainty of evidence and making decisions. *J Clin Epidemiol*. 2022;150:225-242
17. Page MJ, Higgins JPT, Sterne JAC. Chapter 13: Assessing risk of bias due to missing results in a synthesis. In: Higgins JPT, Thomas J, Chandler J, Cumpston M, Li T, Page MJ, Welch VA (editors). *Cochrane Handbook for Systematic Reviews of Interventions version 63 (updated February 2022) Cochrane, 2022 Available from wwwtrainingcochraneorg/handbook*. 2022
18. Schnierer M, Samoš M, Bolek T*, et al.* The Effect of Proton Pump Inhibitor Withdrawal on Dabigatran Etexilate Plasma Levels in Patients With Atrial Fibrillation: A Washout Study. *J Cardiovasc Pharmacol*. 2020;75(4):333-335
19. Nakagawa J, Kinjo T, Iizuka M*, et al.* Impact of gene polymorphisms in drug-metabolizing enzymes and transporters on trough concentrations of rivaroxaban in patients with atrial fibrillation. *Basic Clin Pharmacol Toxicol*. 2021;128(2):297-304
20. Samoš M, Stančiaková L, Ivanková J*, et al.* Monitoring of dabigatran therapy using Hemoclot(®) Thrombin Inhibitor assay in patients with atrial fibrillation. *J Thromb Thrombolysis*. 2015;39(1):95-100
21. Samoš M, Bolek T, Stančiaková L*, et al.* Anti-Xa activity in oral factor Xa inhibitor-treated patients with atrial fibrillation and a higher risk of bleeding: a pilot study. *Blood Coagul Fibrinolysis*. 2018;29(4):369-373
22. Wongcharoen W, Pacharasupa P, Norasetthada L, Gunaparn S, Phrommintikul A. Anti-Factor Xa Activity of Standard and Japan-Specific Doses of Rivaroxaban in Thai Patients With Non-Valvular Atrial Fibrillation. *Circ J*. 2020;84(7):1075-1082
23. Bolek T, Samoš M, Škorňová I*, et al.* Does proton pump inhibition change the on-treatment anti-Xa activity in xabans-treated patients with atrial fibrillation? A pilot study. *J Thromb Thrombolysis*. 2019;47(1):140-145
24. Samoš M, Bolek T, Stančiaková L*, et al.* Does type 2 diabetes affect the on-treatment levels of direct oral anticoagulants in patients with atrial fibrillation? *Diabetes Res Clin Pract*. 2018;135:172-177
25. Nissan R, Spectre G, Hershkovitz A*, et al.* Apixaban Levels in Octogenarian Patients with Non-valvular Atrial Fibrillation. *Drugs Aging*. 2019;36(2):165-177

26. Bánovčin P, Jr., Škorňová I, Samoš M*, et al.* Platelet Aggregation in Direct Oral Factor Xa Inhibitors-treated Patients With Atrial Fibrillation: A Pilot Study. *J Cardiovasc Pharmacol*. 2017;70(4):263-266
27. Bhagirath VC, Eikelboom JW, Hirsh J*, et al.* Apixaban-Calibrated Anti-FXa Activity in Relation to Outcome Events and Clinical Characteristics in Patients with Atrial Fibrillation: Results from the AVERROES Trial. *TH Open*. 2017;1(2):e139-e145
28. Bhagirath VC, Chan N, Hirsh J*, et al.* Plasma Apixaban Levels in Patients Treated Off Label With the Lower Dose. *J Am Coll Cardiol*. 2020;76(24):2906-2907
29. Lin SY, Kuo CH, Yeh SJ*, et al.* Real-World Rivaroxaban and Apixaban Levels in Asian Patients With Atrial Fibrillation. *Clin Pharmacol Ther*. 2020;107(1):278-286
30. Mavri A, Vene N, Božič-Mijovski M*, et al.* Apixaban concentration variability and relation to clinical outcomes in real-life patients with atrial fibrillation. *Sci Rep*. 2021;11(1):13908
31. Mukai Y, Wada K, Miyamoto K*, et al.* The influence of residual apixaban on bleeding complications during and after catheter ablation of atrial fibrillation. *J Arrhythm*. 2017;33(5):434-439
32. Shin H, Cho MC, Kim RB*, et al.* Laboratory measurement of apixaban using anti-factor Xa assays in acute ischemic stroke patients with non-valvular atrial fibrillation. *J Thromb Thrombolysis*. 2018;45(2):250-256
33. Skeppholm M, Al-Aieshy F, Berndtsson M*, et al.* Clinical evaluation of laboratory methods to monitor apixaban treatment in patients with atrial fibrillation. *Thromb Res*. 2015;136(1):148-153
34. Suwa M, Morii I, Kino M. Rivaroxaban or Apixaban for Non-Valvular Atrial Fibrillation - Efficacy and Safety of Off-Label Under-Dosing According to Plasma Concentration. *Circ J*. 2019;83(5):991-999
35. Suzuki S, Yamashita T, Akao M, Okumura K. Clinical implications of assessment of apixaban levels in elderly atrial fibrillation patients: J-ELD AF registry sub-cohort analysis. *Eur J Clin Pharmacol*. 2020;76(8):1111-1124
36. Takatsuki S, Kimura T, Sugimoto K*, et al.* Real-world monitoring of direct oral anticoagulants in clinic and hospitalization settings. *SAGE Open Med*. 2017;5:2050312117734773
37. Harenberg J, Du S, Wehling M*, et al.* Measurement of dabigatran, rivaroxaban and apixaban in samples of plasma, serum and urine, under real life conditions. An international study. *Clin Chem Lab Med*. 2016;54(2):275-283
38. Nosáľ V, Petrovičová A, Škorňová I*, et al.* Plasma levels of direct oral anticoagulants in atrial fibrillation patients at the time of embolic stroke: a pilot prospective multicenter study. *Eur J Clin Pharmacol*. 2022;78(4):557-564

39. Roşian AN, Roşian Ş H, Kiss B*, et al.* Interindividual Variability of Apixaban Plasma Concentrations: Influence of Clinical and Genetic Factors in a Real-Life Cohort of Atrial Fibrillation Patients. *Genes (Basel)*. 2020;11(4)
40. Martin JL, Esmaeili H, Manuel RC*, et al.* Pharmacokinetics/Pharmacodynamics of Dabigatran 75 mg Twice Daily in Patients With Nonvalvular Atrial Fibrillation and Severely Impaired Renal Function. *J Cardiovasc Pharmacol Ther*. 2018;23(5):399-406
41. Bolek T, Samoš M, Škorňová I*, et al.* Dabigatran Levels in Elderly Patients with Atrial Fibrillation: First Post-Marketing Experiences. *Drugs Aging*. 2018;35(6):539-544
42. Bolek T, Samoš M, Stančiaková L*, et al.* The impact of atorvastatin on dabigatran plasma levels in patients with atrial fibrillation. *Blood Coagul Fibrinolysis*. 2021;32(1):69-71
43. Chang YT, Hu YF, Liao JN*, et al.* The assessment of anticoagulant activity to predict bleeding outcome in atrial fibrillation patients receiving dabigatran etexilate. *Blood Coagul Fibrinolysis*. 2016;27(4):389-395
44. Chaussade E, Hanon O, Boully C*, et al.* Real-Life Peak and Trough Dabigatran Plasma Measurements over Time in Hospitalized Geriatric Patients with Atrial Fibrillation. *J Nutr Health Aging*. 2018;22(1):165-173
45. Ji Q, Zhang C, Xu Q*, et al.* The impact of ABCB1 and CES1 polymorphisms on dabigatran pharmacokinetics and pharmacodynamics in patients with atrial fibrillation. *Br J Clin Pharmacol*. 2021;87(5):2247-2255
46. Lin SY, Tang SC, Kuo CH*, et al.* Factors affecting serum concentration of dabigatran in Asian patients with non-valvular atrial fibrillation. *J Formos Med Assoc*. 2019;118(7):1154-1160
47. Liu Z, Xie Q, Xiang Q*, et al.* Anti-FXa-IIa activity test in Asian and its potential role for drug adherence evaluation in patients with direct oral anticoagulants: a nationwide multi-center synchronization study. *Cardiovasc Diagn Ther*. 2020;10(5):1293-1302
48. Mochalina N, Juhlin T, Platonov PG, Svensson PJ, Wieloch M. Concomitant use of dronedarone with dabigatran in patients with atrial fibrillation in clinical practice. *Thromb Res*. 2015;135(6):1070-1074
49. Reilly PA, Lehr T, Haertter S*, et al.* The effect of dabigatran plasma concentrations and patient characteristics on the frequency of ischemic stroke and major bleeding in atrial fibrillation patients: the RE-LY Trial (Randomized Evaluation of Long-Term Anticoagulation Therapy). *J Am Coll Cardiol*. 2014;63(4):321-328
50. Šinigoj P, Malmström RE, Vene N*, et al.* Dabigatran Concentration: Variability and Potential Bleeding Prediction In "Real-Life" Patients With Atrial Fibrillation. *Basic Clin Pharmacol Toxicol*. 2015;117(5):323-329

51. Skeppholm M, Hjemdahl P, Antovic JP*, et al.* On the monitoring of dabigatran treatment in "real life" patients with atrial fibrillation. *Thromb Res*. 2014;134(4):783-789
52. Skripka A, Sychev D, Bochkov P*, et al.* Factors Affecting Trough Plasma Dabigatran Concentrations in Patients with Atrial Fibrillation and Chronic Kidney Disease. *High Blood Press Cardiovasc Prev*. 2020;27(2):151-156
53. Tomita H, Araki T, Kadokami T*, et al.* Factors influencing trough and 90-minute plasma dabigatran etexilate concentrations among patients with non-valvular atrial fibrillation. *Thromb Res*. 2016;145:100-106
54. Zhang C, Zhang P, Li H*, et al.* The effect of dabigatran on thrombin generation and coagulation assays in rabbit and human plasma. *Thromb Res*. 2018;165:38-43
55. Zhu Z, Shen Z, Shi A*, et al.* Dabigatran plasma concentration indicated the risk of patients with non-valvular atrial fibrillation. *Heart Vessels*. 2022;37(5):821-827
56. Boonen K, Schmitz E, Rozestraten F*, et al.* Real life dabigatran and metabolite concentrations, focused on inter-patient variability and assay differences in patients with atrial fibrillation. *Clin Chem Lab Med*. 2017;55(12):2002-2009
57. Silva VM, Scanavacca M, Darrieux F, Cavalheiro C, Strunz CC. Routine Coagulation Tests in Patients With Nonvalvular Atrial Fibrillation Under Dabigatran and Rivaroxaban Therapy: An Affordable and Reliable Strategy? *Clin Appl Thromb Hemost*. 2019;25:1076029619835053
58. Taune V, Wallén H, Ågren A*, et al.* Whole blood coagulation assays ROTEM and T-TAS to monitor dabigatran treatment. *Thromb Res*. 2017;153:76-82
59. Koretsune Y, Yamashita T, Kimura T*, et al.* Short-Term Safety and Plasma Concentrations of Edoxaban in Japanese Patients With Non-Valvular Atrial Fibrillation and Severe Renal Impairment. *Circ J*. 2015;79(7):1486-1495
60. Ruff CT, Giugliano RP, Braunwald E*, et al.* Association between edoxaban dose, concentration, anti-Factor Xa activity, and outcomes: an analysis of data from the randomised, double-blind ENGAGE AF-TIMI 48 trial. *Lancet*. 2015;385(9984):2288-2295
61. Testa S, Legnani C, Antonucci E*, et al.* Drug levels and bleeding complications in atrial fibrillation patients treated with direct oral anticoagulants. *J Thromb Haemost*. 2019;17(7):1064-1072
62. Hirota N, Suzuki S, Yamasaki M*, et al.* Analysis of bioMARKer Distribution and Individual Reproducibility Under Rivaroxaban Treatment in Japanese Patients with Non-Valvular Atrial Fibrillation (R-MARK Study, CVI ARO2). *Int Heart J*. 2020;61(4):695-704

63. Horinaka S, Sugawara R, Yonezawa Y, Ishimitsu T. Factor Xa inhibition by rivaroxaban in the trough steady state can significantly reduce thrombin generation. *Br J Clin Pharmacol*. 2018;84(1):79-87
64. Al-Aieshy F, Malmström RE, Antovic J*, et al.* Clinical evaluation of laboratory methods to monitor exposure of rivaroxaban at trough and peak in patients with atrial fibrillation. *Eur J Clin Pharmacol*. 2016;72(6):671-679
65. Miklič M, Mavri A, Vene N*, et al.* Intra- and inter- individual rivaroxaban concentrations and potential bleeding risk in patients with atrial fibrillation. *Eur J Clin Pharmacol*. 2019;75(8):1069-1075
66. Shyamkumar K, Hirsh J, Bhagirath VC*, et al.* Plasma Rivaroxaban Level to Identify Patients at Risk of Drug Overexposure: Is a Single Measurement of Drug Level Reliable? *TH Open*. 2021;5(1):e84-e88
67. Silva VM, Scanavacca M, Darrieux F, Cavalheiro-Filho C, Strunz CC. Effects of rivaroxaban on coagulation tests in patients with non-valvular atrial fibrillation under real-life conditions. *Thromb Res*. 2017;154:26-27
68. Guyatt GH, Oxman AD, Schünemann HJ, Tugwell P, Knottnerus A. GRADE guidelines: A new series of articles in the Journal of Clinical Epidemiology. *Journal of Clinical Epidemiology*. 2011;64(4):380-382
