# Supplementary material for: Usual On-therapy Ranges of Drug Concentrations in Patients with Atrial Fibrillation Treated with Direct Oral Anticoagulants: A Systematic Review and Meta-analysis
Source: Thromb Haemost. 2024 Nov 21;125(6):563–73. doi: 10.1055/a-2446-1348 (PMC12115550; doi:10.1055/a-2446-1348)
Supplement: Supplementary file 4 — Supporting Information File 4 [file 10-1055-a-2446-1348-s24030110-4.pdf]

Supporting Information File 4 — Study characteristics and main analyses to estimate on-therapy ranges

Usual On-therapy Ranges of Drug Concentrations in Patients With Atrial Fibrillation Treated With Direct Oral Anticoagulants: a Systematic Review and Meta-analysis

Last updated on: August 7, 2024

Table of contents

Table S1. Characteristics of included studies 7

Results on usual on-therapy ranges in more detail 24

Apixaban: 24

Trough levels of the 2.5 mg twice daily dose: 24

Peak levels of the 2.5 mg twice daily dose: 24

Trough levels of the 5 mg twice daily dose 25

Peak levels of 5 mg twice daily dose 26

Dabigatran 26

Trough levels of the 75 twice daily dose 26

Peak levels of the 75 mg twice daily dose 27

Trough levels of the 110 twice daily dose 27

Peak levels of the 110 mg twice daily dose 28

Trough levels of the 150 twice daily dose 28

Peak levels of the 150 mg twice daily dose 29

Edoxaban 30

Trough levels of the 15 once daily dose 30

Peak levels of the 15 once daily dose 30

Trough levels of the 30 once daily dose 31

Peak levels of the 30 once daily dose 31

Trough levels of the 60 once daily dose 32

Peak levels of the 60 once daily dose 33

Rivaroxaban 33

Trough levels of the 10 once daily dose 33

Peak levels of the 10 once daily dose 34

Trough levels of the 15 once daily dose 34

Peak levels of the 15 once daily dose 35

Trough levels of the 20 once daily dose 36

Peak levels of the 20 once daily dose 36

Risk of bias and concern of indirectness: 37

Inconsistency and imprecision: 38

Figure S1. Median (and 10th – 90th percentiles) of drug levels for apixaban 39

Figure S2. Median (and 10th – 90th percentiles) of drug levels for dabigatran 40

Figure S3. Median (and 10th – 90th percentiles) of drug levels for edoxaban 41

Figure S4. Median (and 10th – 90th percentiles) of drug levels for rivaroxaban 42

Figure S5. Estimating the pooled median trough level of each direct oral anticoagulant stratified by administered dose. 43

A. Apixaban 2.5 mg twice daily 44

B. Apixaban 5 mg twice daily 45

C. Dabigatran 75 mg twice daily 46

D. Dabigatran 110 mg twice daily 47

E. Dabigatran 150 mg twice daily 48

F. Edoxaban 15 mg once daily 49

G. Edoxaban 30 mg once daily 50

H. Edoxaban 60 mg once daily 51

I. Rivaroxaban 10 mg once daily 52

J. Rivaroxaban 15 mg once daily ... 53

K. Rivaroxaban 20 mg once daily ... 54

Figure S6. Estimating the pooled 10th percentile of trough levels of each direct oral anticoagulant stratified by administered dose and using the modified QE-method ... 55

A. Apixaban 2.5 mg twice daily ... 56

B. Apixaban 5 mg twice daily ... 57

C. Dabigatran 75 mg twice daily ... 58

D. Dabigatran 110 mg twice daily ... 59

E. Dabigatran 150 mg twice daily ... 60

F. Edoxaban 15 mg once daily ... 61

G. Edoxaban 30 mg once daily ... 62

H. Edoxaban 60 mg once daily ... 63

I. Rivaroxaban 10 mg once daily ... 64

J. Rivaroxaban 15 mg once daily ... 65

K. Rivaroxaban 20 mg once daily ... 66

Figure S7. Estimating the pooled 90th percentile of trough levels of each direct oral anticoagulant stratified by administered dose and using the modified QE-method ... 67

A. Apixaban 2.5 mg twice daily ... 68

B. Apixaban 5 mg twice daily ... 69

C. Dabigatran 75 mg twice daily ... 70

D. Dabigatran 110 mg twice daily ... 71

E. Dabigatran 150 mg twice daily ... 72

F. Edoxaban 15 mg once daily ... 73

G. Edoxaban 30 mg once daily ... 74

H. Edoxaban 60 mg once daily ... 75

I. Rivaroxaban 10 mg once daily ... 76

J. Rivaroxaban 15 mg once daily ... 77

K. Rivaroxaban 20 mg once daily ... 78

Figure S8. Estimating the pooled median peak level of each direct oral anticoagulant stratified by administered dose ... 79

A. Apixaban 2.5 mg twice daily ... 80

B. Apixaban 5 mg twice daily ... 81

C. Dabigatran 75 mg twice daily ... 82

D. Dabigatran 110 mg twice daily ... 83

E. Dabigatran 150 mg twice daily ... 84

F. Edoxaban 15 mg once daily ... 85

G. Edoxaban 30 mg once daily ... 86

H. Edoxaban 60 mg once daily ... 87

I. Rivaroxaban 10 mg once daily ... 88

J. Rivaroxaban 15 mg once daily ... 89

K. Rivaroxaban 20 mg once daily ... 90

Figure S9. Estimating the pooled 10th percentile of peak levels of each direct oral anticoagulant stratified by administered dose and using the modified QE-method ... 91

A. Apixaban 2.5 mg twice daily ... 92

B. Apixaban 5 mg twice daily ... 93

C. Dabigatran 75 mg twice daily ... 94

D. Dabigatran 110 mg twice daily 95

E. Dabigatran 150 mg twice daily 96

F. Edoxaban 15 mg once daily 97

G. Edoxaban 30 mg once daily 98

H. Edoxaban 60 mg once daily 99

I. Rivaroxaban 10 mg once daily 100

J. Rivaroxaban 15 mg once daily 101

K. Rivaroxaban 20 mg once daily 102

Figure S10. Main analysis: Estimating the pooled 90th percentile of peak levels of each direct oral anticoagulant stratified by administered dose and using the modified QE-method 103

A. Apixaban 2.5 mg twice daily 104

B. Apixaban 5 mg twice daily 105

C. Dabigatran 75 mg twice daily 106

D. Dabigatran 110 mg twice daily 107

E. Dabigatran 150 mg twice daily 108

F. Edoxaban 15 mg once daily 109

G. Edoxaban 30 mg once daily 110

H. Edoxaban 60 mg once daily 111

I. Rivaroxaban 10 mg once daily 112

J. Rivaroxaban 15 mg once daily 113

K. Rivaroxaban 20 mg once daily 114

References 115

Table S1. Characteristics of included studies

Study (year)	DOAC	Sample size (by dose)	Assay	Key population characteristics
Al-Aieshy et al. (2016)[1]	Rivaroxaban	71:<br>▪ 61 on 20 mg OD<br>▪ 10 on 15 mg OD	LC-MS/MS	Inclusion criteria: AF patients treated with rivaroxaban according to clinical routine care. All study patients reported full compliance.<br><br>Patients on the 15-mg OD dose (*n* = 10) had a significantly higher mean age, lower body weight, lower CrCl, were more likely to be of female gender, and had a non-significant tendency to have a higher $CHADS_2$ score than the 20-mg OD dosed group (*n* = 61).
Bánovčin Jr et al. (2017)[2]	Apixaban	12 on 2.5 mg BID	BIOPHEN Heparin kit & Rivaroxaban/Apixaban Plasma Calibrator	Inclusion criteria: hospitalized factor Xa inhibitors–treated patients with NVAF. No patient was taking antiplatelet agents or other medication known to substantially affect platelet function (peak samples tested for platelet aggregation).
	Rivaroxaban	9 on 15 mg OD		
Bhagirath et al. (2017)[3]<br><br>*AVERROES*	Apixaban	2,392:<br>▪ 2,247 on 5 mg BID<br>▪ 145 on 2.5 mg BID	Rotachrom Heparin	Inclusion criteria: AF and additional risk factor for stroke ($CHADS_2$ score of 1 or with documented peripheral arterial disease). All patients were considered unsuitable for treatment with a vitamin K antagonist.
Bhagirath et al. (2020)[4]	Apixaban	62<br>▪ 18 on 5 mg BID	STA-Liquid anti-Xa assay	Inclusion criteria: AF with at least 1 of the following criteria: age >85 years, body weight <50 kg, or CrCl <40 ml/min

Study (year)	DOAC	Sample size (by dose)	Assay	Key population characteristics
		▪ 44 (20 off-label) on 2.5 mg BID		
Bolek et al. (2018)[5]	Dabigatran	21:<br>▪ 16 on 150 mg BID<br>▪ 34 on 110 mg BID	Hemoclot Thrombin Inhibitor	Inclusion criteria: patients with NVAF treated with dabigatran.<br><br>Exclusion criteria: disabling or recent stroke, recent or pending surgery, recent or known bleeding disorders, uncontrolled hypertension, need for anticoagulation for disorders other than AF, planned ablation or surgery for AF, severe renal dysfunction (eGFR <30 mL/min/ 1.73 m^2), active liver disease, extreme body weight (body mass index <18 kg/m^2 and > 40 kg/m^2), and pregnancy.
Bolek et al. (2019)[6]	Apixaban	35:<br>▪ 16 on 5 mg BID<br>▪ 19 on 2.5 mg BID	BIOPHEN Heparin kit & Rivaroxaban/ Apixaban Plasma Calibrator	Inclusion criteria: patients with NVAF treated with either apixaban or rivaroxaban and 'need for long term anticoagulation'.<br><br>Exclusion criteria: disabling or recent stroke, recent or pending surgery, recent or known bleeding disorders, uncontrolled hypertension, the need for anticoagulation for disorders other than AF, planned ablation or surgery for AF, severe renal dysfunction (eGFR <30 mL/min/1.73 m^2), active liver disease, extreme body weight (body mass index <18 kg/m^2 and >40 kg/m^2), and pregnancy.
	Rivaroxaban	42<br>▪ 10 on 20 mg OD<br>▪ 32 on 15 mg OD		
Bolek et al. (2021)[7]	Dabigatran	65<br>▪ 30 on 150 mg BID<br>▪ 35 on 110 mg BID	Hemoclot Thrombin Inhibitor	Inclusion criteria: Dabigatran-treated patients with AF. Atorvastatin dosed 40 mg OD was co-administrated in 31 individuals.

Study (year)	DOAC	Sample size (by dose)	Assay	Key population characteristics
				A lower dose of dabigatran (110 mg BID) was used in 35 patients (19 in statin-treated group) who had reduced glomerular filtration (eGFR 30–50 ml/min/1.73 m^2), or higher age (75 years).
Boonen et al. (2017)[8]	Dabigatran	40 on 150 mg BID	UPLC-MS/MS	Inclusion criteria: Patients with AF treated with dabigatran for at least 1 week.<br><br>Exclusion criteria: age <18 years, BMI >30 kg/m^2, previous malignancy, concomitant use of platelet aggregation inhibitors or other anticoagulants, kidney failure (eGFR <30 mL/min/1.73 m^2).
Chan et al. (2015)[9]	Dabigatran	100:<br>▪ 60 on 150 mg BID<br>▪ 40 on 110 mg BID	Hemoclot Thrombin inhibitor	Inclusion criteria: adult patients with AF receiving long-term treatment with dabigatran visiting the hematology outpatient clinic.<br><br>Exclusion criteria: Those who were either geographically inaccessible for follow-up, or unwilling or unable to provide written informed consent were not eligible for inclusion.
Chang et al. (2016)[10]	Dabigatran	208<br>▪ 33 on 150 mg BID<br>▪ 175 on 110 mg BID	Hemoclot Thrombin Inhibitor	Inclusion criteria: NVAF patients who received dabigatran etexilate treatment with an approved indication for a DOAC according to American College of Cardiology/American Heart Association Guidelines.

Study (year)	DOAC	Sample size (by dose)	Assay	Key population characteristics
Chaussade et al. (2018)[11]	Dabigatran	68 on 110 mg BID	Hemoclot Thrombin Inhibitor	Inclusion criteria: Hemodynamically stable patients hospitalized at the geriatric department in a French university hospital. Patients were ≥75 years of age, had NVAF, and were novel to treatment with an oral anticoagulant.
de Vries et al. (2022)[12]	Apixaban	77<br>▪ 50 on 5 mg BID<br>▪ 27 on 2.5 mg BID	STA-Liquid anti-Xa assay	Inclusion criteria: Patients were eligible if they were treated with apixaban and had received at least 1 week of treatment.<br><br>Exclusion criteria: Those who were either geographically inaccessible for follow-up, or unwilling or unable to provide written informed consent were not eligible for inclusion.
Harenberg et al. (2016)[13]	Apixaban	6 on 5 mg BID	Technochrom assay; HaemosIL assay	Inclusion criteria: Patients received anticoagulation for prevention of systemic embolism in NVAF. All patients were at steady state.<br><br>Exclusion criteria: Patients on a lower dose DOAC.
	Dabigatran	6 on 150 mg BID	Direct Thrombin nhibitor Assas; S2238 chromogenic substrate assay	
	Rivaroxaban	6 on 20 mg OD	Technochrom assay; HaemosIL assay	
Hirota et al. (2020)[14]	Rivaroxaban	219<br>▪ 153 on 15 mg OD<br>▪ 66 on 10 mg OD	LC-MS/MS	Inclusion criteria: Japanese patients (Tokyo) with NVAF on rivaroxaban for the prevention of ischemic stroke.<br><br>Exclusion criteria: receiving dual antiplatelet therapy, inadequate dosage of rivaroxaban at enrolment, rivaroxaban hypersensitivity, liver dysfunction with clotting disorder, moderate or high liver dysfunction (Child-Pugh

Study (year)	DOAC	Sample size (by dose)	Assay	Key population characteristics
				classification B or C), renal dysfunction (CrCl < 30 mL/minute), pregnancy, use of HIV protease inhibitors (ritonavir, atazanavir, indinavir, etc.), use of azole antimycotic agents (itraconazole, voriconazole, ketoconazole, etc., excluding fluconazole), use of drugs containing cobicistat, acute bacterial endocarditis, lack of written informed consent to participation in this study, judged to be inappropriate by researchers (i.e., incapable of understanding the study protocol due to dementia, intellectual disturbance, and/or psychiatric/psychosomatic disorder).
Horinaka et al. (2018)[15]	Rivaroxaban	51<br>▪ 41 on 15 mg OD<br>▪ 10 on 10 mg OD	LC-MS/MS	Inclusion criteria: ≥20 years, newly diagnosed patients with NVAF, referred to Dokkyo Medical University Hospital to initiate oral anticoagulant treatment with rivaroxaban.<br><br>Exclusion criteria: <6 months after the onset of acute myocardial infarction, unstable angina or arteriosclerosis obliterans, within 6 months after surgery, having acute phase congestive heart failure (defined by National Institute for Health and Care Excellence guideline), taking dual-antiplatelet therapy, concomitant chronic kidney disease (CrCl<30 ml/min), known malignancy, rheumatic disease, uncontrollable hypertension or infection, considered ineligible to participate by the attending physician.

Study (year)	DOAC	Sample size (by dose)	Assay	Key population characteristics
Ji et al. (2020)[16]	Dabigatran	198 on 110 mg OD	BIOPHEN DTI	Inclusion criteria: Patients with NVAF treated with 110 mg OD dabigatran for at least 4 continuous weeks, and admitted for elective catheter ablation.<br><br>Exclusion criteria: (i) presence of a prosthetic heart valve or hemodynamically significant valvular disease; (ii) presence of thrombus in atrium or atrial appendage by transoesophageal echocardiography; (iii) severe liver impairment (Child–Pugh B/C and liver cirrhosis) or renal dysfunction (eGFR <30 mL/min/1.73m^2); (iv) comorbidities with significant risk for bleeding such as peptic ulcer and thrombocytopenia, or clinically active bleeding; (v) concomitant use of any anticoagulants, antiplatelets, or strong P-glycoprotein inhibitors (systemic ketoconazole, cyclosporine, and tacrolimus); (vi) unable to fulfil the follow-up visits and declined follow-up phone calls from the researchers; and (vii) incompliance with the prescribed use of dabigatran, such as change in dosage or dosing interval.
Koretsune et al. (2015)[17]	Edoxaban	79<br>▪ 19 on 60 mg OD<br>▪ 21 on 30 mg OD<br>▪ 39 on 15 mg OD	LC-MS/MS	Inclusion criteria: ≥20 years and history of NVAF (documented by electrical tracing within the past 12 months), anticoagulant therapy indicated, $CHADS_2$ score ≥1, and CrCl of either ≥15 to <30 ml/min or ≥50 ml/min.<br><br>Exclusion criteria: hemodialysis, bleeding risk, receiving other anticoagulant therapy (except warfarin, rivaroxaban, or dabigatran).

Study (year)	DOAC	Sample size (by dose)	Assay	Key population characteristics
Lin et al. (2019)[18]	Dabigatran	46<br>▪ 8 on 150 mg BID<br>▪ 38 (23 off-label) on 110 mg BID	UPLC-MS/MS	Inclusion criteria: ≥20 years, dabigatran use >7 days.<br><br>Exclusion criteria: pregnant, breastfeeding, failed to provide informed consent, unable to comply with at least one scheduled blood sample collection.
Lin et al. (2020)[19]	Apixaban	105<br>▪ 44 (3 off-label) on 5 mg BID<br>▪ 61 (60 off-label) on 2.5 mg BID	UPLC-MS/MS	Inclusion criteria: ≥20 years, AF, rivaroxaban or apixaban use for >7 days.<br><br>Exclusion criteria: pregnant, breastfeeding, refused to provide informed consent, failed to comply with at least one blood sample collection
	Rivaroxaban	73 (18 off-label)		Data not included in this review because of mixed doses.
Liu et al. (2020)[20]	Dabigatran	122 on 110 mg BID	BIOPHEN Rivaroxaban/Dabigatran Calibrator	Inclusion criteria: ≥18 years, dabigatran or rivaroxaban use for stroke prevention in NVAF patients, conscious and could understand and answer questions.
	Rivaroxaban	149		Data not included in this review because of mixed doses.
Martin et al. (2018)[21]	Dabigatran	60 on 75 mg BID	LC-MS/MS	Inclusion criteria: ≥18 years, CrCl 15-30 mL/min<br><br>Exclusion criteria: known contraindication for dabigatran, active bleeding, mechanical heart valve.
Mavri et al. (2021)[22]	Apixaban	62<br>▪ 32 on 5 mg<br>▪ 30 on 2.5 mg	LC-MS/MS	Inclusion criteria: AF treated with apixaban on average on average for 10 ± 7 months.

Study (year)	DOAC	Sample size (by dose)	Assay	Key population characteristics
Miklič et al. (2019)[23]	Rivaroxaban	60<br>▪ 30 on 20 mg OD<br>▪ 30 (18 off-label) on 15 mg OD	LC-MS/MS	Inclusion criteria: AF treated with rivaroxaban on average for 20 ± 12 months. Among them, there were 30 patients on rivaroxaban 20 mg daily and 30 patients on rivaroxaban 15 mg daily. The lower-dose rivaroxaban was prescribed to patients with moderate renal impairment (CrCl 30–50 mL/min), high bleeding risk, or previous major bleeding, at the discretion of the treating physician.
Mochalina et al. (2015)[24]	Dabigatran	33 on 110 mg BID	Hemoclot thrombin inhibitor	Inclusion criteria: concomitant use of dabigatran 110 mg bid and dronedarone 400 mg bid at discretion of patient's cardiologist at Skåne University Hospital.
Mukai et al. (2017)[25]	Apixaban	58<br>▪ 50 on 10 mg OD<br>▪ 8 (6 off-label) on 5 mg OD	LC-MS/MS	Inclusion criteria: AF patients treated with apixaban (either 5 mg/day or 10 mg/day for > 21 days) and catheter ablation (did not receive apixaban the morning of procedure) from April 2015-March 2016 at the National Cerebral and Cardio- vascular Center.
Nakagawa et al. (2021)[26]	Rivaroxaban	86<br>▪ 80 on 15 mg OD<br>▪ 6 on 10 mg OD	UPLC-MS/MS	Inclusion criteria: rivaroxaban use > 1 week prior to hospitalization.<br><br>Exclusion criteria: contraindication with rivaroxaban.

Study (year)	DOAC	Sample size (by dose)	Assay	Key population characteristics
Nissan et al. (2019)[27]	Apixaban	80<br>▪ 40 on 5 mg BID<br>▪ 40 (20 off-label) on 2.5 mg BID	Liquid anti-Xa & TECHNOVIEW apixaban Calibrator	Inclusion criteria: ≥ 80 years (octogenarians) or ≤70 years old with an established diagnosis of NVAF and CHA_2DS_2-VASc score of ≥ 1, stable renal function and complete adherence to apixaban for > 4 days.<br><br>Exclusion criteria: hemodynamic instability (cardiogenic shock or circulatory collapse), an expected interruption of apixaban in the subsequent 4–8 days (due to a planned invasive procedure), body weight > 120 kg, elevated liver enzymes [ALT/AST > 3 × upper limit of normal or total bilirubin ≥ 1.5 × ULN), CrCl <30 mL/min, a history of gastrointestinal disorders that may affect drug absorption, and substance or alcohol abuse or concomitant medication use that may influence apixaban levels.
Nosál et al. (2022)[28]	Apixaban	22 control patients on 5 mg<br><br>20 stroke patients<br>▪ 8 on 5 mg BID<br>▪ 12 on 2.5 mg BID	BIOPHEN Heparin (LRT) kit	Inclusion criteria: Patients with acute embolic stroke with trough and peak anti-Xa and anti-IIa levels of patients who tolerated long-term high-dose DOAC therapy without any adverse event (ischemia or bleeding).<br><br>Exclusion criteria: Patients were excluded if the time period from symptom onset to hospital admission was more than 12 hours, or had low life expectancy due to an untreatable concomitant disease (such as active cancer).
	Dabigatran	21 control patients on 150 mg BID<br><br>10 stroke patients<br>▪ 6 on 150 mg BID	Hemoclot Thrombin Inhibitor assay	

Study (year)	DOAC	Sample size (by dose)	Assay	Key population characteristics
		▪ 4 on 110 mg BID		
	Rivaroxaban	14 control patients on 20 mg OD<br><br>13 stroke patients<br>▪ 9 on 20 mg OD<br>▪ 4 on 15 mg OD	LC-MS/MS	
Reilly et al. (2014)[29]<br><br>*RELY*	Dabigatran	9,183<br>▪ 4,600 on 150 mg BID<br>▪ 4,583 on 110 mg BID	LC-MS/MS	Inclusion criteria: valid blood sample and all ischemic stroke/SEE or bleeding events that occurred on-treatment.<br><br>Exclusion criteria: off-treatment at the time of sampling or the time of event.
Roşian et al. (2020)[30]	Apixaban	53 on 5 mg BID	LC-MS/MS	Inclusion criteria: NVAF, willing to attend to the hospital for blood sampling at the specified visits and consented to provide at least two blood samples.<br><br>Exclusion criteria: age <18, inconsistent dosing.
Ruff et al. (2015)[31]<br><br>*ENGAGE-AF TIMI 48*	Edoxaban	14,069<br>▪ 5,251 on 60 mg OD<br>▪ 7,034 on 30 mg OD<br>▪ 1,785 on 15 mg OD	LC-MS/MS	Inclusion criteria: ≥21 years, AF (documented on an electrical tracing within 12 months), $CHADS_2$ ≥2, and anticoagulation planned for the trial duration.

Study (year)	DOAC	Sample size (by dose)	Assay	Key population characteristics
Samoš et al. (2018a)[32]	Apixaban	17 on 5 mg BID	Hemoclot Thrombin Inhibitor & BIOPHEN Heparin (LRT) kit	Inclusion criteria: Consecutive patients with NVAF admitted to Department of Internal Medicine. All patients had been taking DOACs prior to their hospitalization and continued with the DOAC therapy during hospitalization.
	Dabigatran	20 on 110 mg BID		
	Rivaroxaban	28 on 10 mg OD		
Samoš et al. (2015)[33]	Dabigatran	19<br>▪ 5 on 150 mg BID<br>▪ 15 on 110 mg BID	Hemoclot Thrombin Inhibitor	Inclusion criteria: NVAF treated with dabigatran after fulfilling standard European Medicines Agency criteria.<br><br>Exclusion criteria: disabling or recent stroke, recent or pending surgery, recent or known bleeding disorders, uncontrolled hypertension, need for anticoagulation for disorders other than AF, planned ablation or surgery for AF, severe renal dysfunction (CrCl <30 ml/min), active liver disease, and pregnancy.
Samoš et al. (2018b)[34]	Rivaroxaban	24 on 15 mg OD	BIOPHEN Heparin (LRT) kit	Inclusion criteria: Newly diagnosed NVAF, the need for long-term anticoagulation and a higher risk of bleeding.<br><br>Exclusion criteria: disabling or recent stroke, recent or pending surgery, recent or known bleeding disorders, uncontrolled hypertension, the need for anticoagulation for disorders other than atrial fibrillation, planned ablation or surgery for atrial fibrillation, severe renal dysfunction (CrCl <30 ml/min), and active liver disease.
	Apixaban	15		Data not included in this review because of mixed doses.

Study (year)	DOAC	Sample size (by dose)	Assay	Key population characteristics
Schnierer et al. (2020)[35]	Dabigatran	23<br>▪ 16 on 150 mg BID<br>▪ 7 on 110 mg BID	Hemoclot Thrombin Inhibitor	Inclusion criteria: long-term dabigatran and proton pump inhibitor therapy requiring hospitalization for uncontrolled tachycardia or symptomatic heart failure.
Shin et al. (2018)[36]	Apixaban	85<br>▪ 60 on 5 mg BID<br>▪ 25 on 2.5 mg BID	STA-Liquid Anti-Xa	Inclusion criteria: NVAF and CHA_2DS_2-VASC score >2.<br><br>Exclusion criteria: contraindication to anticoagulation treatment, severe hepatic impairment, or renal impairment (total bilirubin >3.0 mg/dL, serum creatinine level >2.5 mg/dL).
Shyamkumar et al. (2021)[37]	Rivaroxaban	100<br>▪ 91 on 20 mg OD<br>▪ 9 on 15 mg OD	STA-Liquid Anti-Xa	Inclusion criteria: Consecutive adult patients with AF (permanent, paroxysmal, or persistent) or venous thromboembolism (idiopathic or provoked) receiving long-term rivaroxaban therapy were enrolled from outpatient clinics.<br><br>Exclusion criteria: Eligible patients who were either geographically inaccessible for follow-up, or unwilling or unable to provide written informed consent, were excluded.
Silva et al. (2017)[38]	Rivaroxaban	100 on 20 mg OD	Biophen DiXal assay	Inclusion criteria: rivaroxaban concentration assessed by specific chromogenic test in plasma of patients receiving treatment for atrial fibrillation.
Silva et al. (2019)[39]	Dabigatran	30 on 150 mg BID	Hemoclot thrombin inhibitor & Biophen anti-	Inclusion criteria: Patients diagnosed with nonvalvular AF attended in our clinical anticoagulation ambulatory and

Study (year)	DOAC	Sample size (by dose)	Assay	Key population characteristics
			activated factor X Rivaroxaban kit	already receiving anticoagulation treatment (dabigatran or rivaroxaban) were invited to participate.<br><br>Exclusion criteria: abnormal coagulation tests, creatinine concentrations >2.0 mg/dL, and eGFR <30 mL/min/1.73 m^2.
	Rivaroxaban	100 on 20 mg OD		
Šinigoj et al. (2015)[40]	Dabigatran	44<br>▪ 23 on 150 mg BID<br>▪ 21 on 110 mg BID	LC-MS/MS	Inclusion criteria: 44 patients with atrial fibrillation who started treatment with dabigatran. A lower dose of dabigatran was prescribed to patients with moderately impaired renal function (creatinine clearance 30–50 mL/min), elderly patients (>75 years), those with a history of major bleeding, those on amiodarone, verapamil or antiplatelet drugs, and to frail patients.
Skeppholm et al. (2014)[41]	Dabigatran	90<br>▪ 73 on 150 mg BID<br>▪ 17 on 110 mg BID	LC-MS/MS & Hemoclot thrombin inhibitors	Inclusion criteria: Ninety patients treated with dabigatran due to NVAF were recruited from Danderyd's Hospital in Stockholm County where they were followed as outpatients.
Skeppholm et al. (2015)[42]	Apixaban	70<br>▪ 60 on 5 mg BID<br>▪ 10 on 2.5 mg BID	LC-MS/MS & STA® Liquid Anti-FXa	Inclusion criteria: Patients with AF treated with apixaban were recruited from the coagulation centre at Danderyd´s Hospital in the Stockholm County during the period 2013-10-01 and 2014-06-03. Oral and written informed consent was obtained from each participant. The patients were treated with apixaban according to clinical routine care.

Study (year)	DOAC	Sample size (by dose)	Assay	Key population characteristics
				Exclusion criteria: Patients were excluded from the study if they were in treatment with any possibly interacting drug according to the summary of product characteristics.<br><br>Note: All study patients reported full compliance during the last three days before trough plasma samples were collected in median 12.3 hours (9.8 – 18.8; min-max) after last intake of the drug.
Skripka et al. (2020)[43]	Dabigatran	60<br>▪ 24 on 150 mg BID<br>▪ 36 on 110 mg BID	LC-MS/MS	Inclusion criteria: chronic kidney disease 3A–3B stage (eGFR 30-59 ml/min/1.73 m^2); opted for dabigatran treatment.
Suwa et al. (2019)[44]	Apixaban	119<br>▪ 58 on 5 mg BID<br>▪ 61 (35 off-label) on 2.5 mg BID	Biophen® DiXaI	Inclusion criteria: Outpatients (168 men and 87 women; age range, 45–90 years) with NVAF, who were undergoing treatment with factor Xa inhibitors (either rivaroxaban or apixaban).
	Rivaroxaban	136<br>▪ 90 on 15 mg OD<br>▪ 46 (27 off-label) on 10 mg OD		
Suzuki et al. (2020)[45]	Apixaban	943<br>▪ 431 on 5 mg BID<br>▪ 512 on 2.5 mg BID	STA®-Liquid Anti-Xa	Inclusion criteria: Japanese patients with NVAF aged ≥75 years who visited the participating facilities after the start of the main study and had been taking or started taking apixaban

Study (year)	DOAC	Sample size (by dose)	Assay	Key population characteristics
				Exclusion criteria: a history of hypersensitivity to apixaban, active bleeding symptoms, liver disease with coagulation disorders.
Takatsuki et al. (2017)[46]	Apixaban	27<br>▪ 20 on 5 mg BID<br>▪ 7 on 2.5 mg BID	STA-Liquid Anti-Xa, STA-Rivaroxaban Calibrator, & STA-Apixaban Calibrator kit	Inclusion criteria: Patients who were taking DOACs for anticoagulation therapy for AF in a clinic setting.
	Rivaroxaban	27<br>▪ 23 on 15 mg OD<br>▪ 4 on 10 mg OD		
	Edoxaban	144 on 30 mg OD		Data not included in this review because patients on edoxaban were treated for prophylaxis of venous thrombosis after total hip or knee arthroplasty.
Taune et al. (2017)[47]	Dabigatran	30 on 150 mg BID	LC-MS/MS	Inclusion criteria: dabigatran treatment > 2 weeks
Testa et al. (2016)[48]	Apixaban	40<br>▪ 20 on 5 mg BID<br>▪ 20 on 2.5 mg BID	Hyphen Biomed & Calibrator Stago	Inclusion criteria: consecutive patients seen at the anticoagulation clinics were enrolled in the study, provided they had been treated with DOAC for at least one week and were available to attend the clinic for blood sampling at the specified time points.
	Dabigatran	40<br>▪ 20 on 150 mg BID<br>▪ 20 on 110 mg BID		

Study (year)	DOAC	Sample size (by dose)	Assay	Key population characteristics
	Rivaroxaban	40<br>▪ 20 on 20 mg OD<br>▪ 20 on 15 mg OD		
Testa et al. (2019)[49]	Edoxaban	101<br>▪ 48 on 60 mg OD<br>▪ 53 on 30 mg OD	STA- liquid anti-Xa, STA-edoxaban calibration	Inclusion Criteria: This is a prospective, observational study in patients with NVAF treated with edoxaban, performed in a specialized anticoagulation clinic.
Tomita et al. (2016)[50]	Dabigatran	98<br>▪ 40 on 300 mg OD<br>▪ 58 on 220 mg OD	LC-MS/MS	Inclusion criteria: CHA_2DS_2-VaSc scores ≥2, only lansoprazole as proton pump inhibitor.<br><br>Exclusion criteria: cancer, systemic artery or venous diseases.
Wongcharoen et al. (2020)[51]	Rivaroxaban	60<br>▪ 35 (1 off-label) on 20 mg OD<br>▪ 56 on 15 mg OD<br>▪ 18 (3 off-label) on 10 mg OD	BIOPHEN DiXal	Inclusion criteria: ≥18 years with NVAF receiving rivaroxaban.<br><br>Exclusion criteria: severe renal impairment (CrCl <15 mL/min) or poor compliance to medications.
Zhang et al. (2018)[52]	Dabigatran	46 on 110 mg BID	LC-MS/MS	Inclusion criteria: Patients with AF admitting to the cardiology department of the First Affiliated Hospital of Soochow University were recruited.
Zhu et al. (2021)[53]	Dabigatran	86 on 110 mg	UPLC-MS/MS	Inclusion criteria: Diagnosed with NVAF, received anticoagulant therapy with oral dabigatran for ≥18 months,

Study (year)	DOAC	Sample size (by dose)	Assay	Key population characteristics
				provided at least one blood sample for determining dabigatran plasma concentration.<br><br>Exclusion criteria: pregnancy, <18 years old, severe vascular damage, severe liver damage, cancer, prior kidney transplant, long term dialysis.

This table summarized the study characteristics of included primary studies.

AF atrial fibrillation; *BMI* body mass index; *BID* twice daily; *CrCl* creatinine clearance; *DOAC* direct-acting oral anticoagulant; *eGFR* estimated glomerular filtration rate; *LC-MS/MS* liquid chromatography–mass spectrometry/mass spectrometry; *NVAF* non-valvular atrial fibrillation; *OD* once daily; *UPLC-MS/MS* ultra-high performance liquid chromatography–mass spectrometry/mass spectrometry.

Results on usual on-therapy ranges in more detail

Apixaban:

Fig S1 illustrates the usual on-therapy ranges of both trough and peak concentrations for the two approved dosing regimens of apixaban.

Trough levels of the 2.5 mg twice daily dose:

For the 2.5 mg twice daily dose of apixaban, the best estimate for the usual on-therapy range (10th to 90th percentile range) of trough levels is 38 to 155 ng/ml, with lower and upper bounds of the 95% CIs for 10th and 90th percentiles of 31 and 181 ng/ml, respectively.

To determine these ranges, we included seventeen studies (of which 1 is a substudy of a randomized trial) that reported trough levels of a total of 1,011 patients. The pooled median, 10th percentile, and 90th percentile trough level and measures of heterogeneity were 75 ng/ml (95% CI 66-85 ng/ml, Q = 58.30, p <0.0001, I^2 = 73.1%), 38 ng/ml (95% CI 31-45 ng/ml, Q = 71.29, p <0.0001, I^2 = 76.6%), and 155 ng/ml (95% CI 129-181 ng/ml, Q = 80.34, p <0.0001, I^2 = 80.1%), respectively (**Fig S5A, S6A, and S7A)**.

We rated 10 of the 17 studies as at high risk of bias and 5 as at unclear risk of bias; and 15 studies were rated as at high concern of indirectness to our research question and one study as at unclear concern of indirectness. Based on our quality assessments, we rated all three outcomes of interest at moderate quality of evidence (**Table S7-S12 of Supporting Information File 3**).

Peak levels of the 2.5 mg twice daily dose:

For peak levels of the 2.5 mg twice daily dose of apixaban, the best estimate for the usual on-therapy range (10th to 90th percentile range) is 96 to 251 ng/ml, with lower and upper bounds of the 95% CIs for 10th and 90th percentiles of 84 and 271 ng/ml, respectively.

To determine these ranges, we included 12 studies (none are substudies of a randomized trial) reporting on peak levels of 299 patients. The pooled median, 10th percentile, and 90th

percentile peak level of these patients was 155 ng/ml (95% CI 145-165 ng/ml, Q = 9.59, p = 0.5673, I^2 = 0.0%), 96 ng/ml (95% CI 84-108 ng/ml, Q = 27.73, p = 0.0036, I^2 = 61.8%), and 251 ng/ml (95% CI 232-271 ng/ml, Q = 8.57, p = 0.6617, I^2 = 8.3%), respectively (**Fig S8A, S9A, and S10A)**.

We rated 7 of the 12 studies as at high risk of bias and 3 as at unclear risk of bias; and 11 as high concern of indirectness to our research question and none of the studies as at unclear concern. Based on our quality assessments, we rated all three outcomes of interest at moderate quality of evidence (**Table S7-S12 of Supporting Information File 3**).

Trough levels of the 5 mg twice daily dose

For the 5 mg twice daily dose of apixaban, the best estimate for the usual on-therapy range (10th to 90th percentile range) of trough levels is 58 to 206 ng/ml, with lower and upper bounds of the 95% CI for 10th and 90th percentiles, respectively of 46 and 236 ng/ml.

To determine these ranges, we included 19 studies (1 is a substudy of a randomized trial) providing trough levels of 3,226 patients. The pooled median, 10th percentile, and 90th percentile trough level of these patients was 109 ng/ml (95% CI 97-121 ng/ml, Q = 152.69, p <0.001, I^2 = 90.2%), 58 ng/ml (95% CI 46-69 ng/ml, Q = 660.24, p <0.001, I^2 = 93.5%), and 206 ng/ml (95% CI 176-236 ng/ml, Q = 446.59, p <0.001, I^2 = 93.7%), respectively (**Fig S5B, S6B, and S7B)**.

We rated 11 of the 19 studies at high risk of bias and 6 as at unclear risk of bias; and 17 studies as at high concern of indirectness to our research question and none as at unclear concern of indirectness. We rated all three outcomes of interest at moderate quality evidence (**Table S7-S12 of Supporting Information File 3**).

Peak levels of 5 mg twice daily dose

For the 5 mg twice daily dose of apixaban, the best estimate for the usual on-therapy range (10th to 90th percentile range) of peak levels is 132 to 343 ng/ml, with lower and upper bounds of the 95% CI for 10th and 90th percentiles, respectively of 109 and 371 ng/ml.

To determine these ranges, we included 16 studies (none is a substudy of a randomized trial) providing peak levels of 466 patients. The pooled median, 10th percentile, and 90th percentile peak level of these patients was 216 ng/ml (95% CI 192-240 ng/ml, Q = 101.97, p <0.0001, I^2 = 82.1%), 132 ng/ml (95% CI 109-155 ng/ml, Q = 266.46, p <0.0001, I^2 = 88.4%), and 343 ng/ml (95% CI 315-371 ng/ml, Q = 29.40, p = 0.0143, I^2 = 55.4%), respectively (**Fig S8B, S9B, and S10B)**.

We rated 9 of the 16 studies at high risk of bias and 5 as at unclear risk of bias; and 13 studies as at high concern of indirectness to our research question and one as at unclear concern of indirectness. We rated all three outcomes of interest as low quality evidence (**Table S7-S12 of Supporting Information File 3**).

Dabigatran

Fig S2 illustrates the usual on-therapy ranges of both trough and peak concentrations for all three approved dosing regimens of dabigatran.

Trough levels of the 75 twice daily dose

For the 75 mg twice daily dose of dabigatran, the best estimate for the usual on-therapy range (10th to 90th percentile range) of trough levels is 66 to 352 ng/ml, with lower and upper bounds of the 95% CI for 10th and 90th percentiles, respectively of 44 and 438 ng/ml.

This range is based on the results of a single study (a substudy of a randomized trial), providing trough levels of 60 patients. The median, 10th percentile, and 90th percentile trough level of these patients was 161 ng/ml (95% CI 128-194 ng/ml, Q = not applicable [NA], p =

NA, I^2 = NA), 66 ng/ml (95% CI 44-89 ng/ml, Q = NA, *p* = NA, I^2 = NA), and 352 ng/ml (95% CI 267-438 ng/ml, Q = NA, *p* = NA, I^2 = NA), respectively (**Fig S6C, S6C, and S7C)**.

We rated this single study as at unclear risk of bias and at high concern of indirectness to our research question. We rated all three outcomes of interest as low quality evidence (**Table S7-S12 of Supporting Information File 3**).

Peak levels of the 75 mg twice daily dose

For the 75 mg twice daily dose of dabigatran, the best estimate for the usual on-therapy range (10th to 90th percentile range) of peak levels 82 to 444 ng/ml, with lower and upper bounds of the 95% CI for 10th and 90th percentiles, respectively of 57 and 583 ng/ml.

This range is based on the peak levels of the 60 patients from the same study that also provided trough levels. The median, 10th percentile, and 90th percentile peak level of these patients was 195 ng/ml (95% CI 159-231 ng/ml, Q = NA, *p* = NA, I^2 = NA), 82 ng/ml (95% CI 57-107 ng/ml, Q = NA, *p* = NA, I^2 = NA), and 444 ng/ml (95% CI 305-583 ng/ml, Q = NA, *p* = NA, I^2 = NA), respectively (**Fig S8C, S9C, and S10C)**.

We rated this study as at unclear risk of bias and at high concern of indirectness to our research question. We rated all three outcomes of interest as low quality evidence (**Table S7-S12 of Supporting Information File 3**).

Trough levels of the 110 twice daily dose

For the 110 mg twice daily dose of dabigatran, the best estimate for the usual on-therapy range (10th to 90th percentile range) of trough levels 35 to 138 ng/ml, with lower and upper bounds of the 95% CI for 10th and 90th percentiles, respectively of 29 and 157 ng/ml.

To determine these ranges, we included 26 studies (2 are substudies of a randomized trial), which together provided trough levels for 5,303 patients. The pooled median, 10th percentile, and 90th percentile trough level of these patients was 66 ng/ml (95% CI 60-72 ng/ml, Q = 118.08, *p* <0.0001, I^2 = 94.0%), 35 ng/ml (95% CI 29-40 ng/ml, Q = 1,908.07, *p* <0.0001, I^2 =

96.2%), and 138 ng/ml (95% CI 119-157 ng/ml, Q = 861.68, p <0.0001, I^2 = 95.6%), respectively (**Fig S5D, S6D, and S7D)**.

We rated 15 of the 26 studies as at high risk of bias and 4 as at unclear risk of bias; and 21 studies as at high concern of indirectness to our research question and 1 as at unclear concern of indirectness. We rated all three outcomes of interest as moderate quality evidence (**Table S7-S12 of Supporting Information File 3**).

Peak levels of the 110 mg twice daily dose

For the 110 mg twice daily dose of dabigatran, the best estimate for the usual on-therapy range (10th to 90th percentile range) of peak levels 65 to 223 ng/ml, with lower and upper bounds of the 95% CI for 10th and 90th percentiles, respectively of 51 and 265 ng/ml.

To determine these ranges, we included 18 studies (2 are substudies of a randomized trial) reporting peak levels of 5,154 patients. The pooled median, 10th percentile, and 90th percentile trough level of these patients was 118 ng/ml (95% CI 102-134 ng/ml, Q = 1,416.98, p <0.0001, I^2 = 98.2%), 65 ng/ml (95% CI 51-79 ng/ml, Q = 2,315.38, p <0.0001, I^2 = 97.6%), and 223 ng/ml (95% CI 181-265 ng/ml, Q = 1,087.65, p <0.0001, I^2 = 98.6%), respectively (**Fig S8D, S9D, and S10D)**.

We rated 10 of the 18 studies as at high risk of bias and 4 as at unclear risk of bias; and 15 studies as at high concern of indirectness to our research question and none as at unclear concern of indirectness. We rated all three outcomes of interest as moderate quality evidence (**Table S7-S12 of Supporting Information File 3**).

Trough levels of the 150 twice daily dose

For the 150 mg twice daily dose of dabigatran, the best estimate for the usual on-therapy range (10th to 90th percentile range) of trough levels 33 to 151 ng/ml, with lower and upper bounds of the 95% CI for 10th and 90th percentiles, respectively of 27 and 172 ng/ml.

To determine these ranges, we included 18 studies (3 are substudies of a randomized trial) reporting trough levels of 4,693 patients. The pooled median, 10th percentile, and 90th percentile trough level of these patients was 72 ng/ml (95% CI 64-80 ng/ml, Q = 138.52, p <0.0001, I^2 = 79.2%), 33 ng/ml (95% CI 27-38 ng/ml, Q = 69.79, p <0.0001, I^2 = 77.3%), and 151 ng/ml (95% CI 131-172 ng/ml, Q = 1148.04, p <0.0001, I^2 = 80.3%), respectively (**Fig S5E, S6E, and S7E)**.

We rated 7 of the 18 studies as at high risk of bias and 4 as at unclear risk of bias; and 11 studies as at high concern of indirectness to our research question and 1 as at unclear concern of indirectness. We rated all three outcomes of interest as moderate quality evidence (**Table S7-S12 of Supporting Information File 3**).

Peak levels of the 150 mg twice daily dose

For the 150 mg twice daily dose of dabigatran, the best estimate for the usual on-therapy range (10th to 90th percentile range) of peak levels 76 to 285 ng/ml, with lower and upper bounds of the 95% CI for 10th and 90th percentiles, respectively of 64 and 327 ng/ml.

To determine these ranges, we included 17 studies (3 are substudies of a randomized trial) reporting peak levels of 4,996 patients. The pooled median, 10th percentile, and 90th percentile peak level of these patients was 148 ng/ml (95% CI 130-166 ng/ml, Q = 160.89, p <0.0001, I^2 = 85.3%), 76 ng/ml (95% CI 64-89 ng/ml, Q = 78.16, p <0.0001, I^2 = 85.1%), and 285 ng/ml (95% CI 243-327 ng/ml, Q = 172.61, p <0.0001, I^2 = 83.2%), respectively (**Fig S8E, S9E, and S10E)**.

We rated 7 of the 17 studies as at high risk of bias and 5 as at unclear risk of bias; and 10 studies as at high concern of indirectness to our research question and 1 as at unclear concern of indirectness. We rated all three outcomes of interest at moderate quality evidence (**Table S7-S12 of Supporting Information File 3**).

Edoxaban

Fig S3 illustrates the usual on-therapy ranges of both trough and peak concentrations for all three approved dosing regimens of edoxaban.

Trough levels of the 15 once daily dose

For the 15 mg once daily dose of edoxaban, the best estimate for the usual on-therapy range (10th to 90th percentile range) of trough levels 5 to 34 ng/ml, with lower and upper bounds of the 95% CI for 10th and 90th percentiles, respectively of 3 and 41 ng/ml.

To determine these ranges, we included 2 studies (both are substudies of a randomized trial) reporting peak levels of 1,824 patients. The pooled median, 10th percentile, and 90th percentile trough level of these patients was 14 ng/ml (95% CI 9-19 ng/ml, Q = 3.38, p = 0.0659, I^2 = 70.4%), 5 ng/ml (95% CI 3-7 ng/ml, Q = 3.00, p = 0.0832, I^2 = 66.7%), and 34 ng/ml (95% CI 28-41 ng/ml, Q = 1.39, p = 0.2384, I^2 = 28.0%), respectively (**Fig S5F, S6F, and S7F)**.

We rated both studies as at high risk of bias as well as at high concern of indirectness to our research question. We rated the median and 10th percentile value at moderate quality evidence and the 90th percentile at high quality evidence (**Table S7-S12 of Supporting Information File 3**).

Peak levels of the 15 once daily dose

For the 15 mg once daily dose of edoxaban, the best estimate for the usual on-therapy range (10th to 90th percentile range) of peak levels 37 to 144 ng/ml, with lower and upper bounds of the 95% CI for 10th and 90th percentiles, respectively of 25 and 176 ng/ml.

The ranges were based on a single study (which is a substudy of a randomized trial) reporting peak levels of 39 patients. The median, 10th percentile, and 90th percentile peak level of these patients was 74 ng/ml (95% CI 55-94 ng/ml, Q = NA, p = NA, I^2 = NA), 37

ng/ml (95% CI 25-48 ng/ml, Q = NA, p = NA, I^2 = NA), and 144 ng/ml (95% CI 111-176 ng/ml, Q = NA, p = NA, I^2 = NA), respectively (**Fig S8F, S9F, and S10F)**.

We rated the included study as at high risk of bias and at high concern of indirectness to our review question. We rated all three outcomes of interest as very low quality evidence (**Table S7-S12 of Supporting Information File 3**).

Trough levels of the 30 once daily dose

For the 30 mg once daily dose of edoxaban, the best estimate for the usual on-therapy range (10th to 90th percentile range) of trough levels 8 to 54 ng/ml, with lower and upper bounds of the 95% CI for 10th and 90th percentiles, respectively of 6 and 82 ng/ml.

To determine these ranges, we included 4 studies (3 are substudies of a randomized trial) reporting trough levels of 7,107 patients. The pooled median, 10th percentile, and 90th percentile trough level of these patients was 21 ng/ml (95% CI 12-29 ng/ml, Q = 205.70, p <0.0001, I^2 = 99.2%), 8 ng/ml (95% CI 6-10 ng/ml, Q = 47.22, p <0.0001, I^2 = 96.3%), and 54 ng/ml (95% CI 27-82 ng/ml, Q = 385.63, p <0.0001, I^2 = 99.4%), respectively (**Fig S5G, S5G, and S6G)**.

We rated 3 of the 4 studies as at high risk of bias and none as at unclear risk of bias; and 3 studies as at high concern of indirectness to our research question and none as at unclear concern of indirectness. We rated all three outcomes of interest as moderate evidence (**Table S7-S12 of Supporting Information File 3**).

Peak levels of the 30 once daily dose

For the 30 mg once daily dose of edoxaban, the best estimate for the usual on-therapy range (10th to 90th percentile range) of peak levels 57 to 219 ng/ml, with lower and upper bounds of the 95% CI for 10th and 90th percentiles, respectively of 15 and 349 ng/ml.

To determine these ranges, we included 2 studies (1 is a substudy of a randomized trial) reporting peak levels of 74 patients. The pooled median, 10th percentile, and 90th percentile peak level of these patients was 115 ng/ml (95% CI 39-192 ng/ml, Q = 12.09, *p* = 0.0005, I^2 = 91.7%), 57 ng/ml (95% CI 15-99 ng/ml, Q = 11.16, *p* = 0.0008, I^2 = 91.0%), and 219 ng/ml (95% CI 88-349 ng/ml, Q = 10.41, *p* = 0.0013, I^2 = 90.4%), respectively (**Fig S8G, S9G, and S10G)**.

We rated 1 of the 2 studies as at high risk of bias and none as at unclear risk of bias; and 1 study as at high concern of indirectness to our research question and none as at unclear concern of indirectness. We rated all three outcomes of interest as very low quality evidence (**Table S7-S12 of Supporting Information File 3**).

Trough levels of the 60 once daily dose

For the 60 mg once daily dose of edoxaban, the best estimate for the usual on-therapy range (10th to 90th percentile range) of trough levels 13 to 66 ng/ml, with lower and upper bounds of the 95% CI for 10th and 90th percentiles, respectively of 11 and 110 ng/ml.

To determine these ranges, we included 3 studies (2 are substudies of a randomized trial) reporting trough levels of 5,318 patients. The pooled median, 10th percentile, and 90th percentile trough level of these patients was 29 ng/ml (95% CI 18-40 ng/ml, Q = 74.62, *p* <0.0001, I^2 = 96.2%), 13 ng/ml (95% CI 11-14 ng/ml, Q = 3.10, *p* = 0.2126, I^2 = 26.0%), and 66 ng/ml (95% CI 22-110 ng/ml, Q = 879.48, *p* <0.0001, I^2 = 99.6%), respectively (**Fig S5H, S6H, and S7H)**.

We rated 2 of the 3 studies as at high risk of bias as well as at high concern of indirectness to our research question. None of the included studies were at unclear risk of bias or unclear concern of indirectness. We rated the median as moderate quality evidence, and the 10th and 90th percentile values as high quality evidence (**Table S7-S12 of Supporting Information File 3**).

Peak levels of the 60 once daily dose

For the 60 mg once daily dose of edoxaban, the best estimate for the usual on-therapy range (10th to 90th percentile range) of peak levels 127 to 407 ng/ml, with lower and upper bounds of the 95% CI for 10th and 90th percentiles, respectively of 39 and 519 ng/ml.

To determine these ranges, we included 2 studies (1 is a substudy of a randomized trial) reporting peak levels of 67 patients. The pooled median, 10th percentile, and 90th percentile peak level of these patients was 234 ng/ml (95% CI 120-349 ng/ml, Q = 6.35, p = 0.0118, I^2 = 84.2%), 127 ng/ml (95% CI 39-215 ng/ml, Q = 10.30, p = 0.0013, I^2 = 90.3%), and 407 ng/ml (95% CI 294-519 ng/ml, Q = 3.01, p = 0.0827, I^2 = 66.8%), respectively (**Fig S8H, S9H, and S10H)**.

We rated 1 of the 2 studies as at high risk of bias as well as at high concern of indirectness to our research question. None of the studies were at unclear risk of bias or unclear concern of indirectness. We rated all three outcomes of interest as very low quality evidence (**Table S7-S12 of Supporting Information File 3**).

Rivaroxaban

Fig S4 illustrates the usual on-therapy ranges of both trough and peak concentrations for all three approved dosing regimens of rivaroxaban.

Trough levels of the 10 once daily dose

For the 10 mg once daily dose of rivaroxaban, the best estimate for the usual on-therapy range (10th to 90th percentile range) of trough levels 9 to 71 ng/ml, with lower and upper bounds of the 95% CI for 10th and 90th percentiles, respectively of is 5 and 87 ng/ml.

To determine these ranges, we included 4 studies (none are substudies of a randomized trial) reporting peak levels of 83 patients. The pooled median, 10th percentile, and 90th percentile trough level of these patients was 28 ng/ml (95% CI 20-37 ng/ml, Q = 6.07, p = 0.1082, I^2 = 43.7%), 9 ng/ml (95% CI 5-12 ng/ml, Q = 7.24, p = 0.0647, I^2 = 18.2%), and 71

ng/ml (95% CI 54-87 ng/ml, Q = 0.85, p = 0.8367, I^2 = 0.0%), respectively (**Fig S6I, S6I, and S7I)**.

We rated 1 of the 4 studies as at high risk of bias and 2 as at unclear risk of bias; and all studies as at high concern of indirectness to our research question. We rated all three outcomes of interest as low quality evidence (**Table S7-S12 of Supporting Information File 3**).

Peak levels of the 10 once daily dose

For the 10 mg once daily dose of rivaroxaban, the best estimate for the usual on-therapy range (10th to 90th percentile range) of peak levels 136 to 418 ng/ml, with lower and upper bounds of the 95% CI for 10th and 90th percentiles, respectively of is 96 and 462 ng/ml.

To determine these ranges, we included 4 studies (1 is a substudy of a randomized trial) reporting peak levels of 137 patients. The pooled median, 10th percentile, and 90th percentile peak level of these patients was 266 ng/ml (95% CI 244-288 ng/ml, Q = 3.65, p = 0.3019, I^2 = 0.0%), 136 ng/ml (95% CI 96-176 ng/ml, Q = 9.55, p = 0.0228, I^2 = 69.5%), and 418 ng/ml (95% CI 375-462 ng/ml, Q = 2.29, p = 0.5154, I^2 = 13.0%), respectively (**Fig S8I, S9I, and S10I)**.

We rated 2 of the 4 studies as at high risk of bias and the other two as at unclear risk of bias; and all studies as at high concern of indirectness to our research question. We rated the median and the 90th percentile value at low quality evidence, and the 10th percentile value at very low quality evidence (**Table S7-S12 of Supporting Information File 3**).

Trough levels of the 15 once daily dose

For the 15 mg once daily dose of rivaroxaban, the best estimate for the usual on-therapy range (10th to 90th percentile range) of trough levels 16 to 74 ng/ml, with lower and upper bounds of the 95% CI for 10th and 90th percentiles, respectively of is 13 and 90 ng/ml.

To determine these ranges, we included 15 studies (none are substudies of a randomized trial) reporting trough levels of 425 patients. The pooled median, 10th percentile, and 90th percentile trough level of these patients was 32 ng/ml (95% CI 27-37 ng/ml, Q = 45.47, *p* <0.0001, I^2 = 76.7%), 16 ng/ml (95% CI 13-19 ng/ml, Q = 82.72, *p* <0.0001, I^2 = 75.1%), and 74 ng/ml (95% CI 58-90 ng/ml, Q = 98.47, *p* <0.0001, I^2 = 85.4%), respectively (**Fig S5J, S6J, and S7J)**.

We rated 7 of the 15 studies as at high risk of bias and 4 as at unclear risk of bias; and 11 studies as at high concern of indirectness to our research question, and none at unclear concern of indirectness. We rated all three outcomes of interest as low quality evidence (**Table S7-S12 of Supporting Information File 3**).

Peak levels of the 15 once daily dose

For the 15 mg once daily dose of rivaroxaban, the best estimate for the usual on-therapy range (10th to 90th percentile range) of peak levels 131 to 384 ng/ml, with lower and upper bounds of the 95% CI for 10th and 90th percentiles, respectively of is 98 and 433 ng/ml.

To determine these ranges, we included 14 studies (1 is a substudy of a randomized trial) reporting peak levels of 470 patients. The pooled median, 10th percentile, and 90th percentile peak level of these patients was 223 ng/ml (95% CI 184-262 ng/ml, Q = 162.23, *p* <0.0001, I^2 = 90.5%), 131 ng/ml (95% CI 98-163 ng/ml, Q = 179.06, *p* <0.0001, I^2 = 91.9%), and 384 ng/ml (95% CI 335-433 ng/ml, Q = 63.79, *p* <0.0001, I^2 = 79.7%), respectively (**Fig S8J, S9J, and S10J)**.

We rated 7 of the 14 studies as at high risk of bias and 4 as at unclear risk of bias; and 11 studies as at high concern of indirectness to our research question, and none at unclear concern of indirectness. We rated all three outcomes of interest as low quality evidence (**Table S7-S12 of Supporting Information File 3**).

Trough levels of the 20 once daily dose

For the 20 mg once daily dose of rivaroxaban, the best estimate for the usual on-therapy range (10th to 90th percentile range) of trough levels 19 to 72 ng/ml, with lower and upper bounds of the 95% CI for 10th and 90th percentiles, respectively of is 15 and 81 ng/ml.

To determine these ranges, we included 8 studies (none are substudies of a randomized trial) reporting peak levels of 216 patients. The pooled median, 10th percentile, and 90th percentile trough level of these patients was 39 ng/ml (95% CI 34-43 ng/ml, Q = 8.18, p = 00.3168, I^2 = 29.2%), 19 ng/ml (95% CI 15-23 ng/ml, Q = 16.84, p = 0.0184, I^2 = 55.7%), and 72 ng/ml (95% CI 64-81 ng/ml, Q = 6.10, p = 0.5278, I^2 = 14.4%), respectively (**Fig S5K, S6K, and S7K)**.

We rated 5 of the 8 studies as at high risk of bias and 1 as at unclear risk of bias; and 4 studies as at high concern of indirectness to our research question, and none at unclear concern of indirectness. We rated the median and 90th percentile value at low quality evidence, and the 10th percentile value at very low quality evidence (**Table S7-S12 of Supporting Information File 3**).

Peak levels of the 20 once daily dose

For the 20 mg once daily dose of rivaroxaban, the best estimate for the usual on-therapy range (10th to 90th percentile range) of peak levels 169 to 313 ng/ml, with lower and upper bounds of the 95% CI for 10th and 90th percentiles, respectively of is 146 and 391 ng/ml.

To determine these ranges, we included 10 studies (none are a substudy of a randomized trial) reporting peak levels of 361 patients. The pooled median, 10th percentile, and 90th percentile peak level of these patients was 225 ng/ml (95% CI 187-263 ng/ml, Q = 233.16, p <0.0001, I^2 = 99.6%), 169 ng/ml (95% CI 146-192 ng/ml, Q = 242.73, p <0.0001, I^2 = 98.8%), and 313 ng/ml (95% CI 236-391 ng/ml, Q = 122.81, p <0.0001, I^2 = 99.6%), respectively (**Fig S8K, S9K, and S10K)**.

We rated 4 of the 10 studies as at high risk of bias and 3 as at unclear risk of bias; and 4 studies as at high concern of indirectness to our research question, and 2 as at unclear concern of indirectness. We rated all three outcomes of interest as low quality evidence (**Table S7-S12 of Supporting Information File 3**).

Sensitivity analyses

Performing the sensitivity analysis was impossible (because only a single study was available, or all studies fell into the same category) or noninformative because fewer than ten studies were available in 30 (68%) of analyses on risk of bias and concern of inapplicability, 22 (50%) on method of data extraction, and 22 (50%) on the laboratory methods used to determine levels.

For the DOAC regimens for which these analyses were feasible, rather than including all eligible studies, selecting only the studies at low risk of bias and concern of inapplicability studies (**Table S1 and Fig S1 through S4 of Supporting Information File 5**), those studies that provided the 10th and 90th percentile values in their report (**Table S2 and Fig S5 through S8 of Supporting Information File 5**), or studies that used liquid chromatography-mass spectrometry/mass spectrometry to determine levels (**Table S3 and Fig S9 through S12 of Supporting Information File 5**) did not result in consistently higher or lower 10th or 90th percentile values.

Risk of bias and concern of indirectness:

The level of evidence of all outcomes of interest was downgraded due to risk of bias. In nearly all cases, studies were at high risk of bias due to patient selection and not due to the laboratory test or timing of measurement (**Table S7-S8 of Supporting Information File 3**).

To avoid penalizing for the same issue twice, we post-hoc decided to not rate down by one grade for indirectness if the outcome of interest was already downgraded due to high risk of bias (rationale mentioned in **Table S5 of Supporting Information File 3**). Accordingly, none of the outcomes of interest were downgraded due to indirectness (**Table S7 and S9 of Supporting Information File 3**)

Inconsistency and imprecision:

For nearly three-quarters of the outcomes of interest, the level of evidence was downgraded due to inconsistency (i.e., heterogeneity) in the results of the primary studies (**Table S10 of Supporting Information File 3**). Almost all outcomes not downgraded due to inconsistency, were downgraded due to imprecision as the number of patients was below predetermined thresholds based on the optimal information size (**Table S10 and S11 of Supporting Information File 3**). We upgraded the level of evidence due to high precision in about two-thirds (64%) of outcomes of interests for trough levels, and in about one-fifth (18%) of outcomes on peak levels (**Table S11 and S12 of Supporting Information File 3**).

Apixaban	No. of patients	No. of studies[*]	Median[†] [P10, P90][†]	[95% CI of usual range][†, ‡]	Level of evidence[§]
**2.5 mg twice daily**					
Trough	1,011	17	75 [38, 155]	[31, 181]	Moderate
Peak	299	12	155 [96, 251]	[84, 271]	Low to moderate
**5 mg twice daily**					
Trough	3,226	19	109 [58, 206]	[46, 236]	Moderate
Peak	446	16	216 [132, 343]	[109, 371]	Low

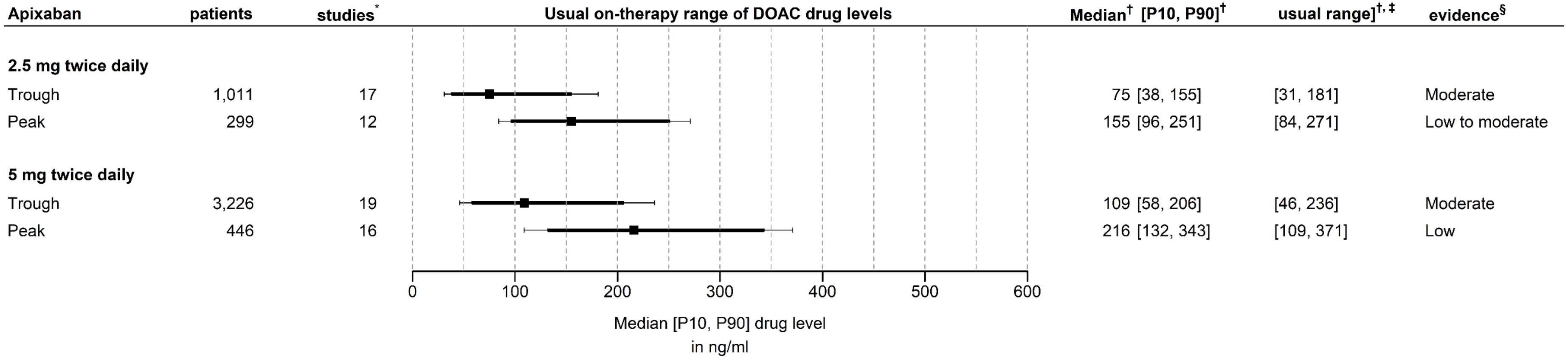

Figure S1. Median (and 10th – 90th percentiles) of drug levels for apixaban

This figure illustrates the usual on-therapy ranges of trough and peak levels of all dosing regimens of apixaban approved for stroke prevention in patients with atrial fibrillation. The squares represent the pooled median values, the solid bold lines the pooled estimates for the 10th to 90th percentile range, and the whiskers the interval from the lower bound of the 95% CI of the pooled 10th percentile (left side) to the upper bound of the 95% CI of the pooled 90th percentile value (right side).

P10 10th percentile; *P90* 90th percentile; *CI* confidence interval; *DOAC* direct oral anticoagulant; *No.* number.

[*] some studies reported on multiple subgroups of patients. Each subgroup was then considered a unique study; [†] estimated with random effects models using the (modified) QE-method;[54,55] [‡] the interval from the lower bound of the 95% CI of the pooled 10th percentile to the upper bound of the 95% CI of the pooled 90th percentile value; [§] Level of evidence following the GRADE-framework and determined for each outcome of interest (i.e., median, 10th percentile, and 90th percentile).[56]

Dabigatran	No. of patients	No. of studies*	Median† [P10, P90]†	[95% CI of usual range]†, ‡	Level of evidence§
**75 mg twice daily**					
Trough	60	1	161 [66, 352]	[44, 438]	Low
Peak	60	1	195 [82, 444]	[57, 583]	Low
**110 mg twice daily**					
Trough	5,303	26	66 [35, 138]	[29, 157]	Moderate
Peak	5,154	18	118 [65, 223]	[51, 265]	Moderate
**150 mg twice daily**					
Trough	4,693	18	72 [33, 151]	[27, 172]	Moderate
Peak	4,996	17	148 [76, 285]	[64, 327]	Moderate

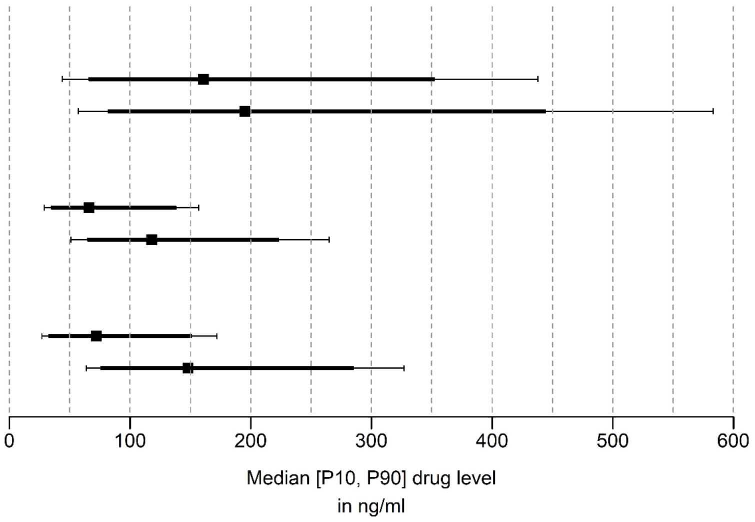

Figure S2. Median (and 10th – 90th percentiles) of drug levels for dabigatran

This figure illustrates the usual on-therapy ranges of trough and peak levels of all dosing regimens of dabigatran approved for stroke prevention in patients with atrial fibrillation. The squares represent the pooled median values, the solid bold lines the pooled estimates for the 10th to 90th percentile range, and the whiskers the interval from the lower bound of the 95% CI of the pooled 10th percentile (left side) to the upper bound of the 95% CI of the pooled 90th percentile value (right side).

P10 10th percentile; *P90* 90th percentile; *CI* confidence interval; *DOAC* direct oral anticoagulant; *No.* number.

[*] some studies reported on multiple subgroups of patients. Each subgroup was then considered a unique study; [†] estimated with random effects models using the (modified) QE-method;[54,55] [‡] the interval from the lower bound of the 95% CI of the pooled 10th percentile to the upper bound of the 95% CI of the pooled 90th percentile value; [§] Level of evidence following the GRADE-framework and determined for each outcome of interest (i.e., median, 10th percentile, and 90th percentile)[56]

Edoxaban	No. of patients	No. of studies*	Usual on-therapy range of DOAC drug levels	Median† [P10, P90]†	[95% CI of usual range]†, ‡	Level of evidence§
**15 mg once daily**						
Trough	1,824	2		14 [5, 34]	[3, 41]	Moderate to high
Peak	39	1		74 [37, 144]	[25, 176]	Very low
**30 mg once daily**						
Trough	7,107	4		21 [8, 54]	[6, 82]	Moderate
Peak	74	2		115 [57, 219]	[15, 349]	Very low
**60 mg once daily**						
Trough	5,318	3		29 [13, 66]	[11, 110]	Moderate to high
Peak	67	2		234 [127, 407]	[39, 519]	Very low

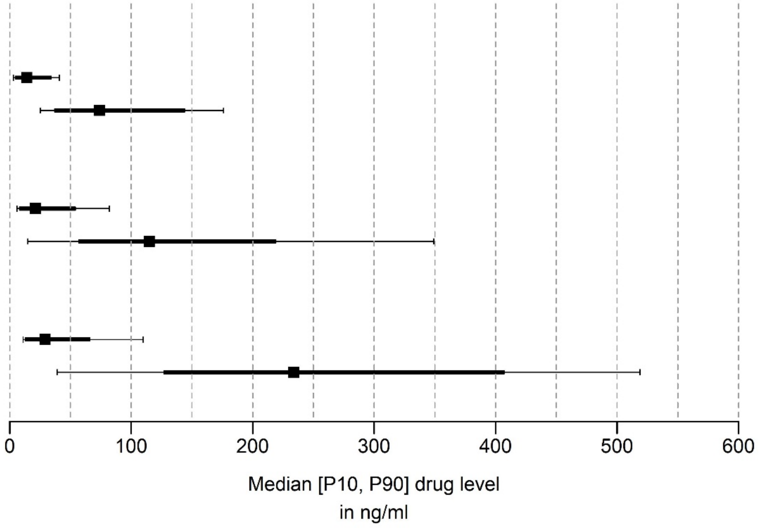

Figure S3. Median (and 10th – 90th percentiles) of drug levels for edoxaban

This figure illustrates the usual on-therapy ranges of trough and peak levels of all dosing regimens of edoxaban approved for stroke prevention in patients with atrial fibrillation. The squares represent the pooled median values, the solid bold lines the pooled estimates for the 10th to 90th percentile range, and the whiskers the interval from the lower bound of the 95% CI of the pooled 10th percentile (left side) to the upper bound of the 95% CI of the pooled 90th percentile value (right side).

P10 10th percentile; *P90* 90th percentile; *CI* confidence interval; *DOAC* direct oral anticoagulant; *No.* number.

* some studies reported on multiple subgroups of patients. Each subgroup was then considered a unique study; † estimated with random effects models using the (modified) QE-method;[54,55] ‡ the interval from the lower bound of the 95% CI of the pooled 10th percentile to the upper bound of the 95% CI of the pooled 90th percentile value; § Level of evidence following the GRADE-framework and determined for each outcome of interest (i.e., median, 10th percentile, and 90th percentile).[56]

Rivaroxaban	No. of patients	No. of studies*	Usual on-therapy range of DOAC drug levels	Median†	[P10, P90]†	[95% CI of usual range]†, ‡	Level of evidence§
**10 mg once daily**							
Trough	83	4		28	[9, 71]	[5, 87]	Low
Peak	137	4		266	[136, 418]	[96, 462]	Low to very low
**15 mg once daily**							
Trough	425	15		32	[16, 74]	[13, 90]	Low
Peak	470	14		223	[131, 384]	[98, 433]	Low
**20 mg once daily**							
Trough	216	8		39	[19, 72]	[15, 81]	Very low to low
Peak	361	10		225	[169, 313]	[146, 391]	Low

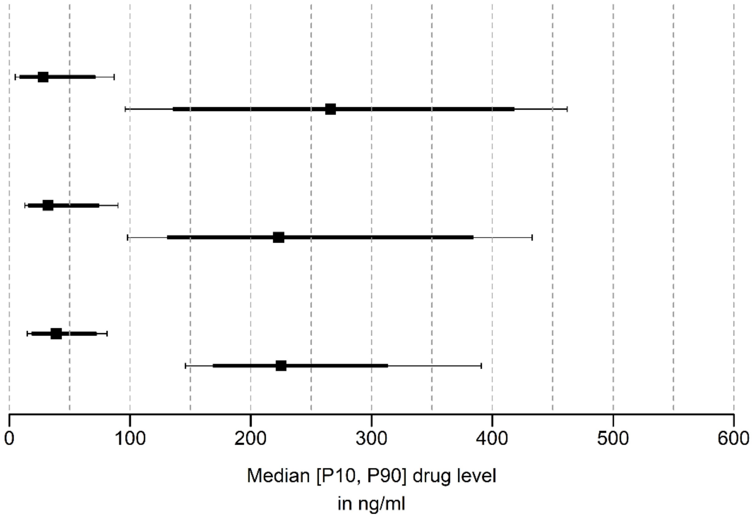

Figure S4. Median (and 10th – 90th percentiles) of drug levels for rivaroxaban

This figure illustrates the usual on-therapy ranges of trough and peak levels of all dosing regimens of rivaroxaban approved for stroke prevention in patients with atrial fibrillation. The squares represent the pooled median values, the solid bold lines the pooled estimates for the 10th to 90th percentile range, and the whiskers the interval from the lower bound of the 95% CI of the pooled 10th percentile (left side) to the upper bound of the 95% CI of the pooled 90th percentile value (right side).

P10 10th percentile; *P90* 90th percentile; *CI* confidence interval; *DOAC* direct oral anticoagulant; *No.* number.

* some studies reported on multiple subgroups of patients. Each subgroup was then considered a unique study; † estimated with random effects models using the (modified) QE-method;[54,55] ‡ the interval from the lower bound of the 95% CI of the pooled 10th percentile to the upper bound of the 95% CI of the pooled 90th percentile value; § Level of evidence following the GRADE-framework and determined for each outcome of interest (i.e., median, 10th percentile, and 90th percentile).[56]

Figure S5. Estimating the pooled median trough level of each direct oral anticoagulant stratified by administered dose.

The forest plots below illustrate the results of our analyses to estimate the median trough level of each DOAC type, stratified by dosing regimen. The squares represent the median values, the solid bold lines the 25th to 75th percentile range, and the whiskers either the minimum to maximum value interval (left side of the plot) or the 95% of the confidence interval of the median values (right side of the plot).

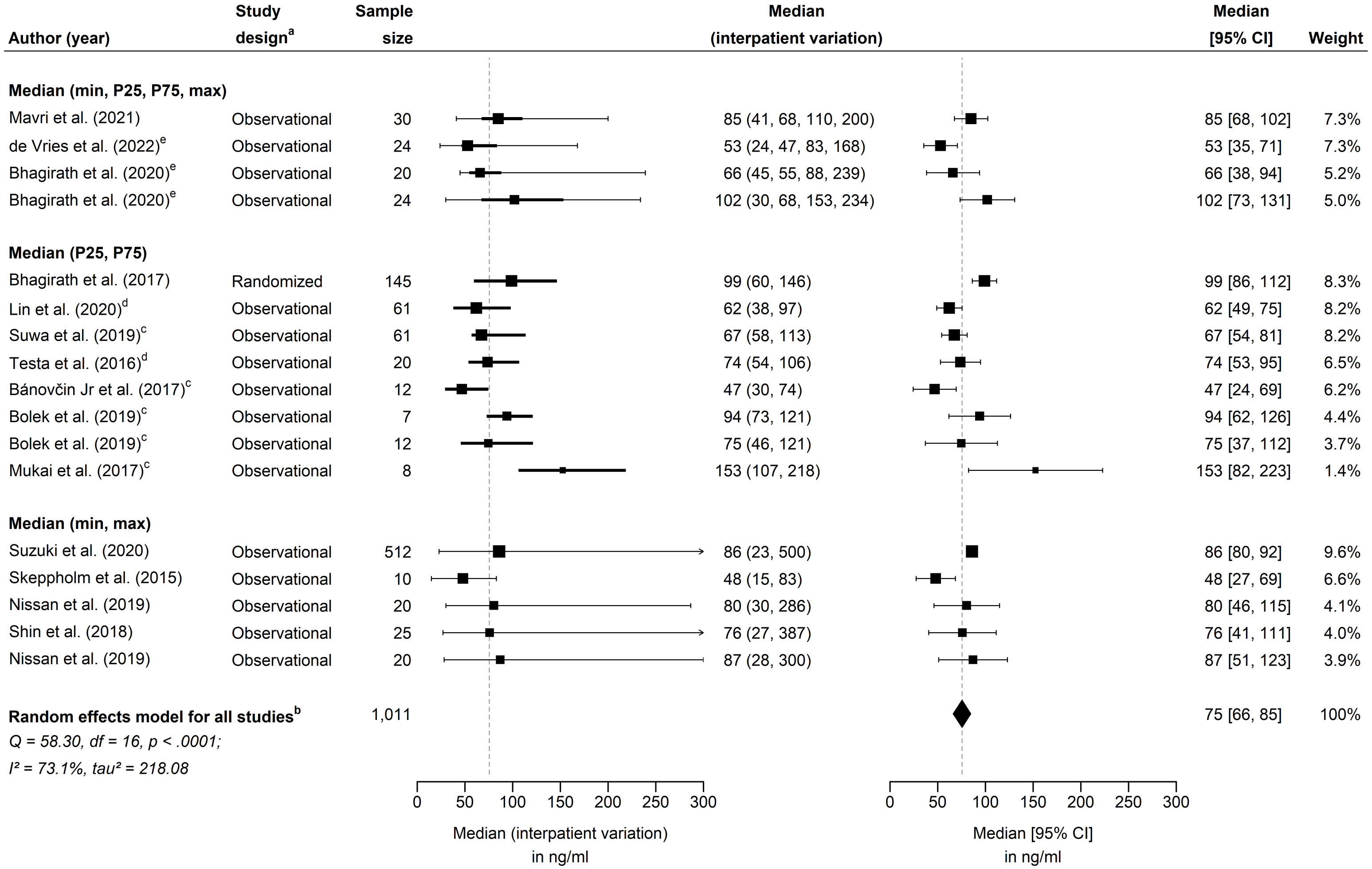

A. Apixaban 2.5 mg twice daily

[a] All analyses of interest were cross-sectional; [b] Random effects model using the quantile-estimation method;[54,57-59] [c] Simulated values were used because only the mean and standard deviation were available; [d] Simulated values were used because available parameters could not readily be included in the QE-method; [e] Percentiles were calculated directly from the original dataset if they were published by the authors of the current review.[4,12,37]

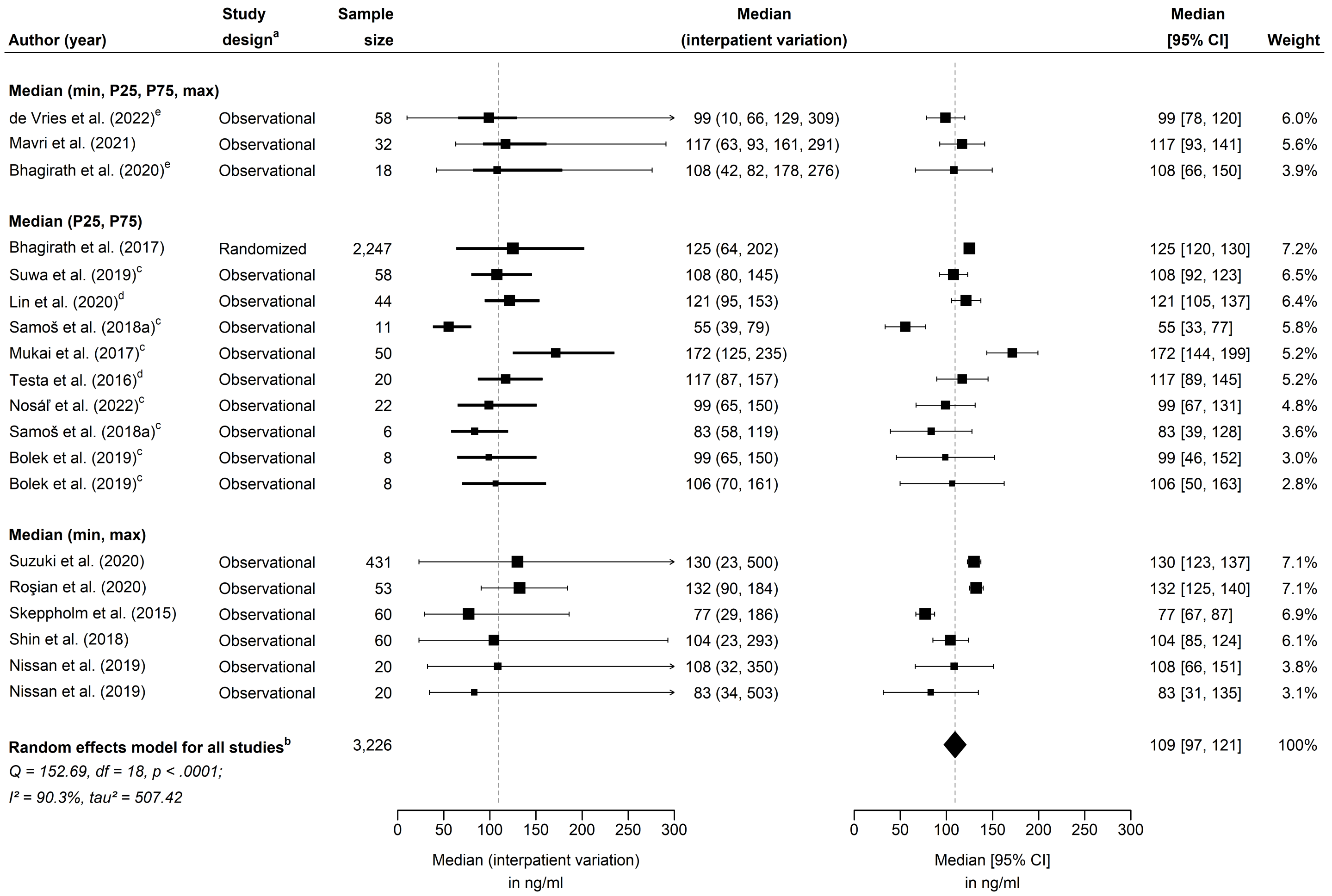

B. Apixaban 5 mg twice daily

[a] All analyses of interest were cross-sectional; [b] Random effects model using the quantile-estimation method;[54,57-59] [c] Simulated values were used because only the mean and standard deviation were available; [d] Simulated values were used because available parameters could not readily be included in the QE-method; [e] Percentiles were calculated directly from the original dataset if they were published by the authors of the current review.[4,12,37]

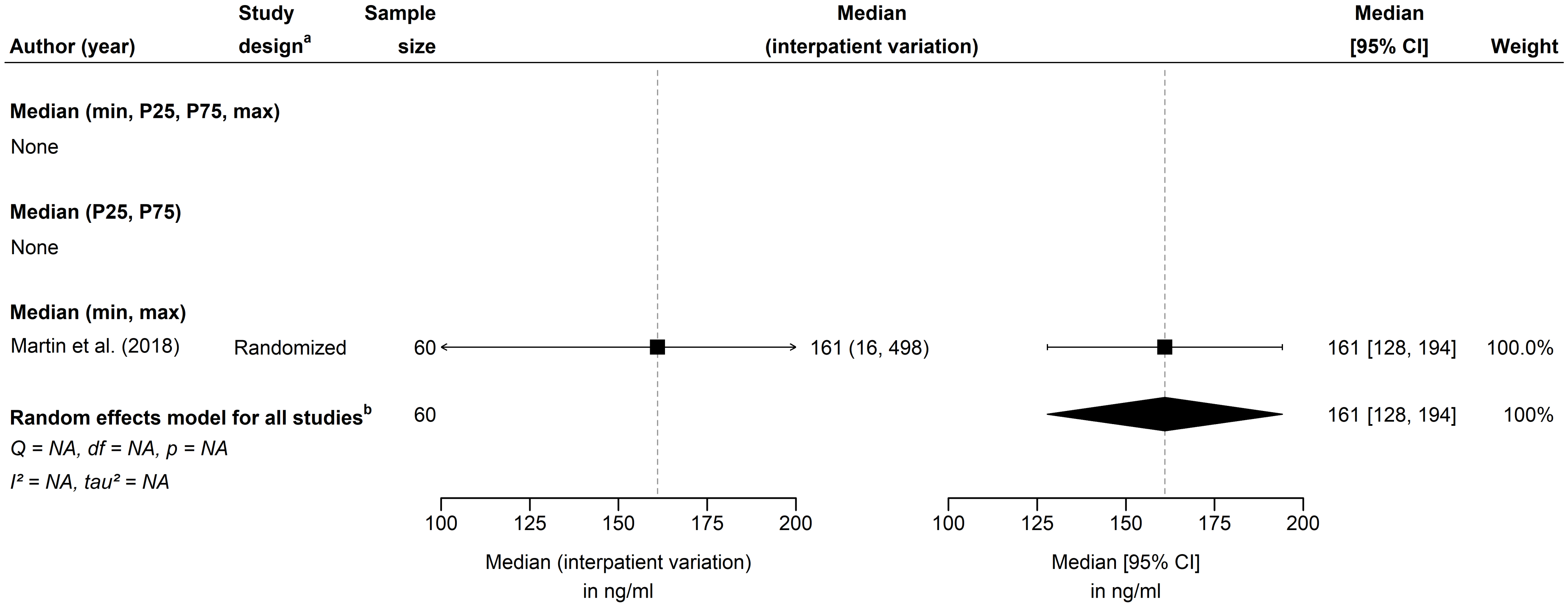

C. Dabigatran 75 mg twice daily

[a] All analyses of interest were cross-sectional; [b] Random effects model using the quantile-estimation method;[54,57-59] [c] Simulated values were used because only the mean and standard deviation were available; [d] Simulated values were used because available parameters could not readily be included in the QE-method; [e] Percentiles were calculated directly from the original dataset if they were published by the authors of the current review.[4,12,37]

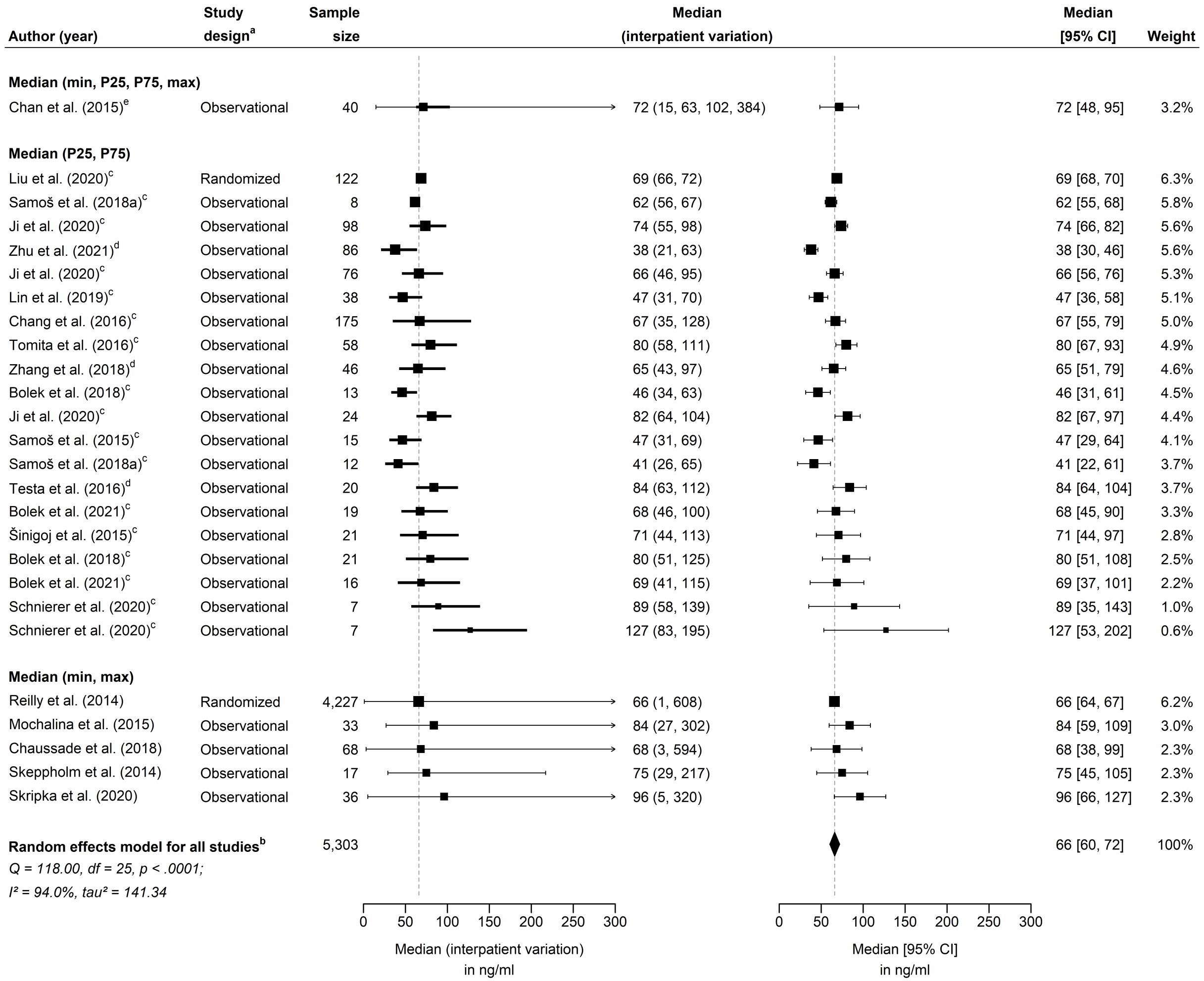

D. Dabigatran 110 mg twice daily

[a] All analyses of interest were cross-sectional; [b] Random effects model using the quantile-estimation method;[54,57-59] [c] Simulated values were used because only the mean and standard deviation were available; [d] Simulated values were used because available parameters could not readily be included in the QE-method; [e] Percentiles were calculated directly from the original dataset if they were published by the authors of the current review.[4,12,37]

Author (year)	Study design[a]	Sample size	Median (interpatient variation)	Median [95% CI]	Weight
**Median (min, P25, P75, max)**					
Chan et al. (2015)[e]	Observational	60	70 (15, 36, 122, 216)	70 [56, 85]	7.1%
**Median (P25, P75)**					
Testa et al. (2016)[d]	Observational	20	49 (35, 67)	49 [36, 61]	7.4%
Šinigoj et al. (2015)[c]	Observational	23	60 (42, 86)	60 [43, 76]	6.7%
Tomita et al. (2016)[c]	Observational	40	96 (70, 132)	96 [79, 114]	6.4%
Boonen et al. (2017)[d]	Randomized	40	60 (34, 99)	60 [42, 78]	6.3%
Boonen et al. (2017)[d]	Randomized	40	68 (41, 108)	68 [49, 86]	6.2%
Bolek et al. (2021)[c]	Observational	12	55 (37, 80)	55 [32, 77]	5.5%
Nosáľ et al. (2022)[c]	Observational	21	71 (47, 107)	71 [48, 94]	5.3%
Bolek et al. (2018)[c]	Observational	16	60 (38, 94)	60 [36, 85]	5.0%
Schnierer et al. (2020)[c]	Observational	16	78 (55, 111)	78 [53, 103]	4.9%
Chang et al. (2016)[c]	Observational	33	90 (55, 152)	90 [61, 120]	4.2%
Bolek et al. (2021)[c]	Observational	18	70 (42, 117)	70 [39, 101]	4.0%
Lin et al. (2019)[c]	Observational	8	51 (32, 83)	51 [20, 83]	3.9%
Schnierer et al. (2020)[c]	Observational	16	142 (96, 211)	142 [91, 193]	2.0%
Samoš et al. (2015)[c]	Observational	5	86 (54, 137)	86 [21, 151]	1.4%
**Median (min, max)**					
Reilly et al. (2014)	Randomized	4,222	93 (1, 809)	93 [91, 95]	9.0%
Skeppholm et al. (2014)	Observational	73	62 (12, 237)	62 [51, 73]	7.8%
Taune et al. (2017)	Observational	30	86 (29, 150)	86 [71, 101]	7.0%
**Random effects model for all studies[b]**		4,693		72 [64, 80]	100%

Q = 138.52, df = 17, p < .0001;
I² = 79.2%, tau² = 195.56

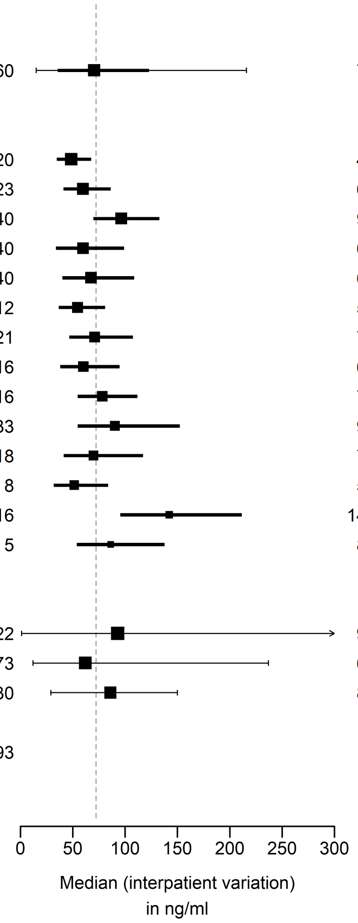

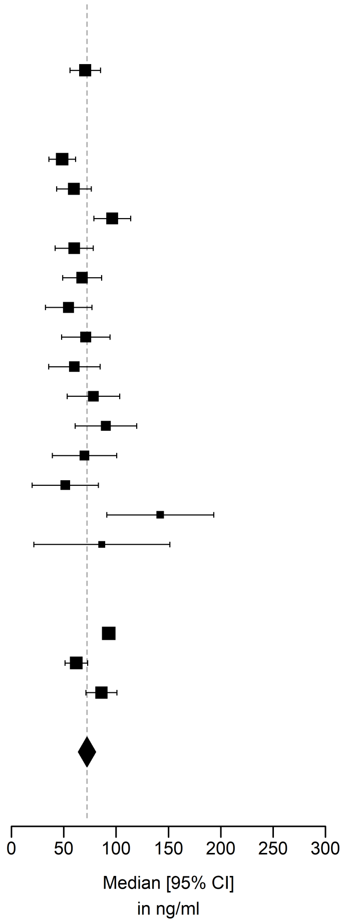

E. Dabigatran 150 mg twice daily

[a] All analyses of interest were cross-sectional; [b] Random effects model using the quantile-estimation method [54,57-59]; [c] Simulated values were used because only the mean and standard deviation were available; [d] Simulated values were used because available parameters could not readily be included in the QE-method; [e] Percentiles were calculated directly from the original dataset if they were published by the authors of the current review [4,12,37].

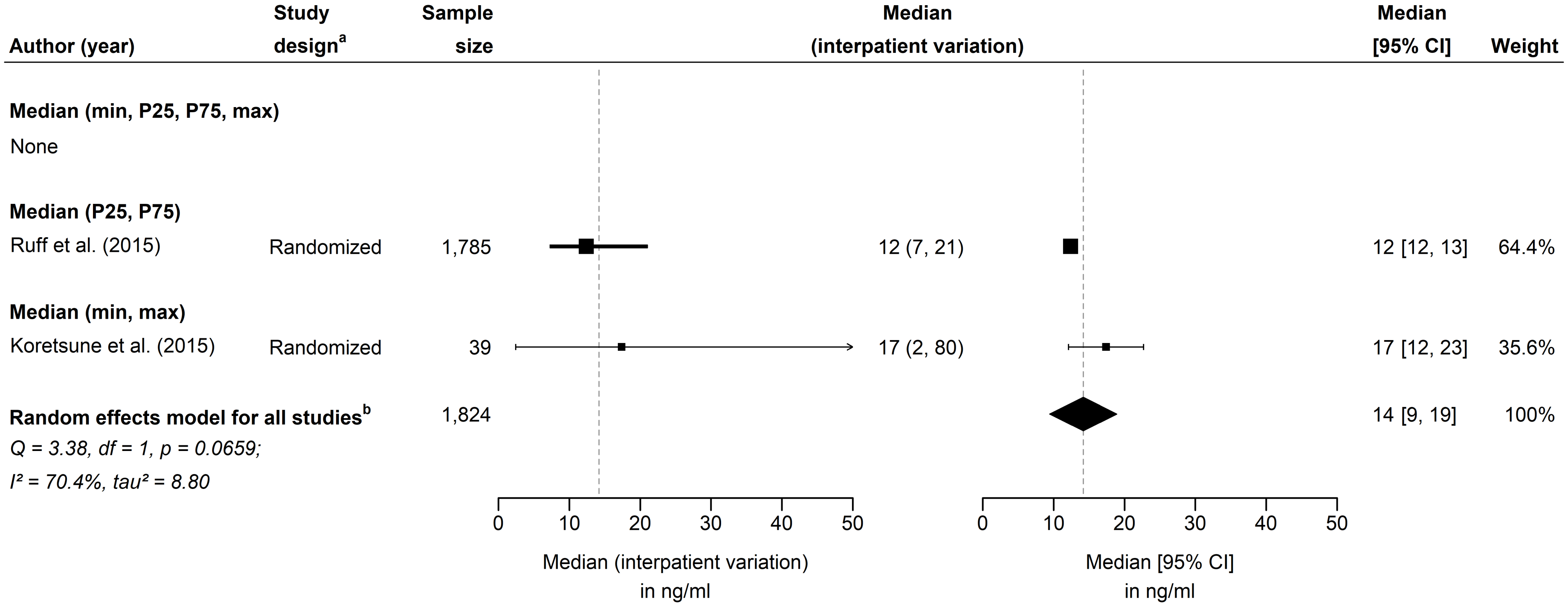

F. Edoxaban 15 mg once daily

[a] All analyses of interest were cross-sectional; [b] Random effects model using the quantile-estimation method;[54,57-59] [c] Simulated values were used because only the mean and standard deviation were available; [d] Simulated values were used because available parameters could not readily be included in the QE-method; [e] Percentiles were calculated directly from the original dataset if they were published by the authors of the current review.[4,12,37]

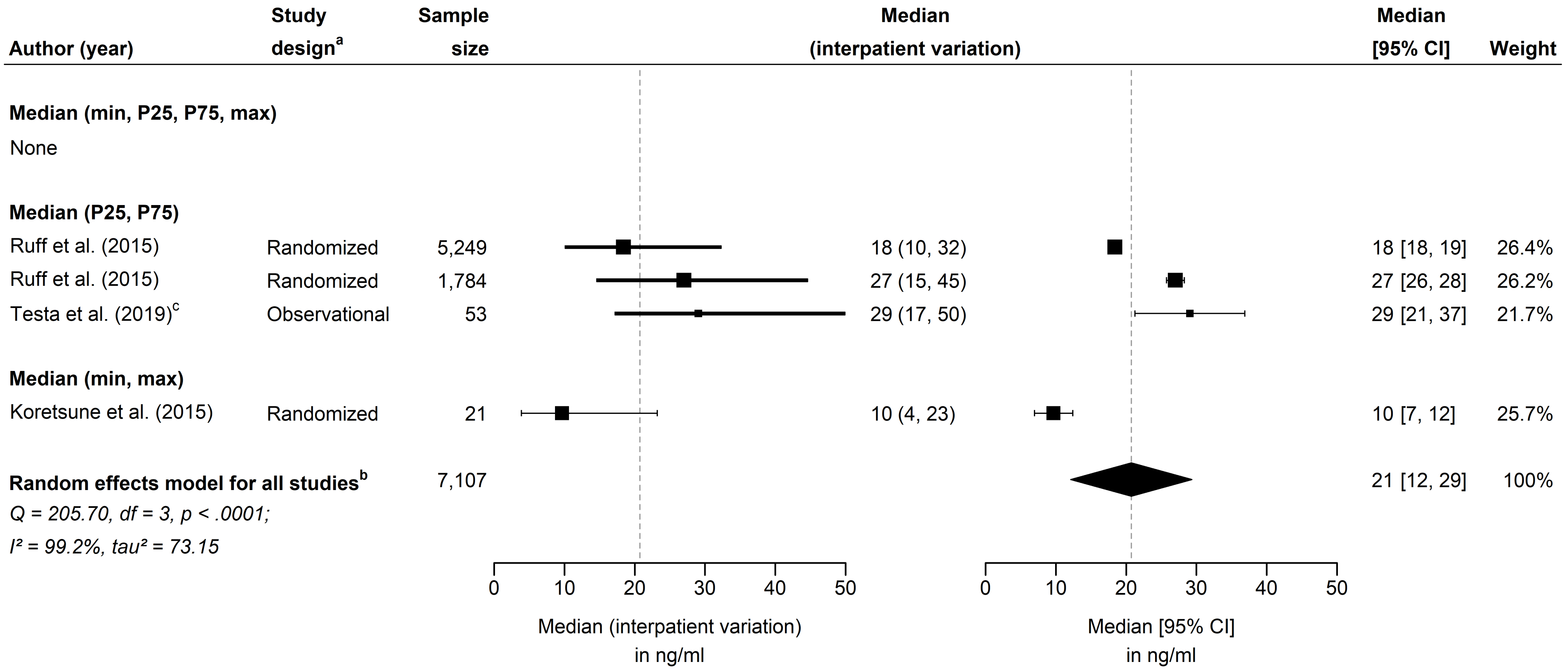

G. Edoxaban 30 mg once daily

[a] All analyses of interest were cross-sectional; [b] Random effects model using the quantile-estimation method;[54,57-59] [c] Simulated values were used because only the mean and standard deviation were available; [d] Simulated values were used because available parameters could not readily be included in the QE-method; [e] Percentiles were calculated directly from the original dataset if they were published by the authors of the current review.[4,12,37]

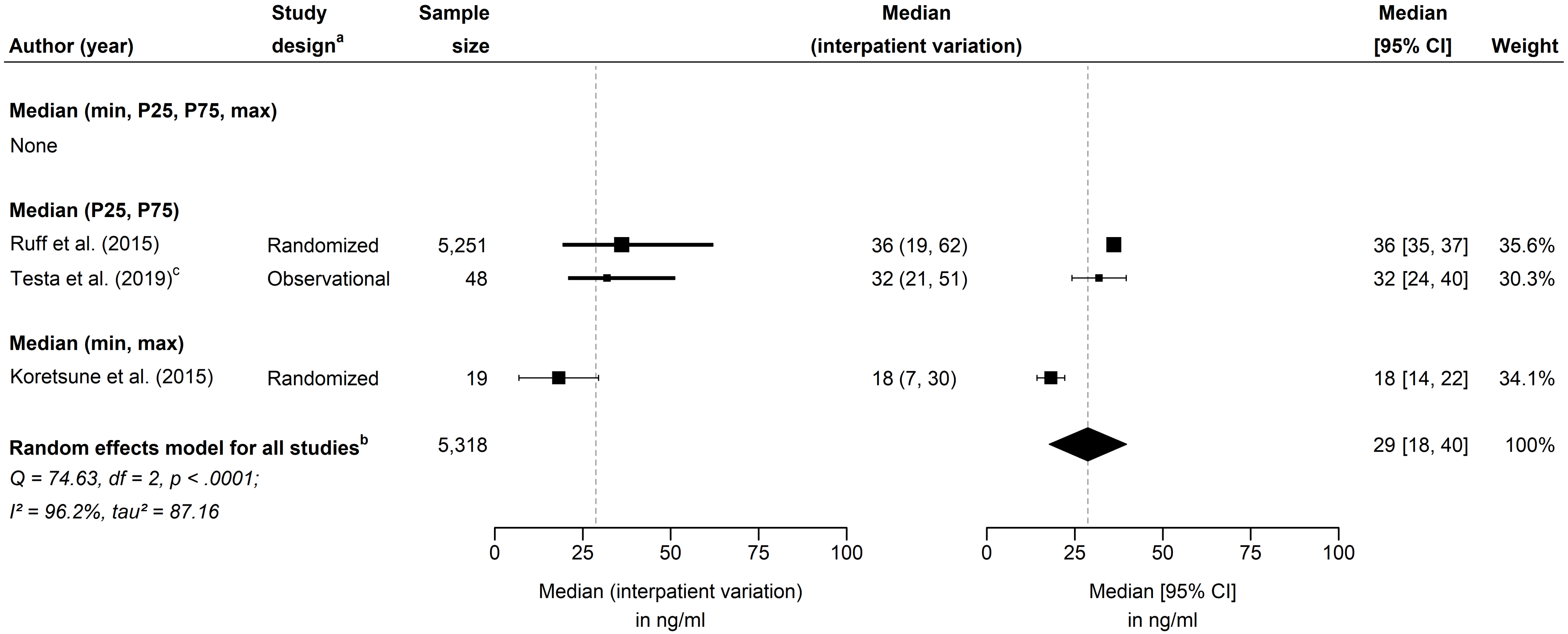

H. Edoxaban 60 mg once daily

[a] All analyses of interest were cross-sectional; [b] Random effects model using the quantile-estimation method;[54,57-59] [c] Simulated values were used because only the mean and standard deviation were available; [d] Simulated values were used because available parameters could not readily be included in the QE-method; [e] Percentiles were calculated directly from the original dataset if they were published by the authors of the current review.[4,12,37]

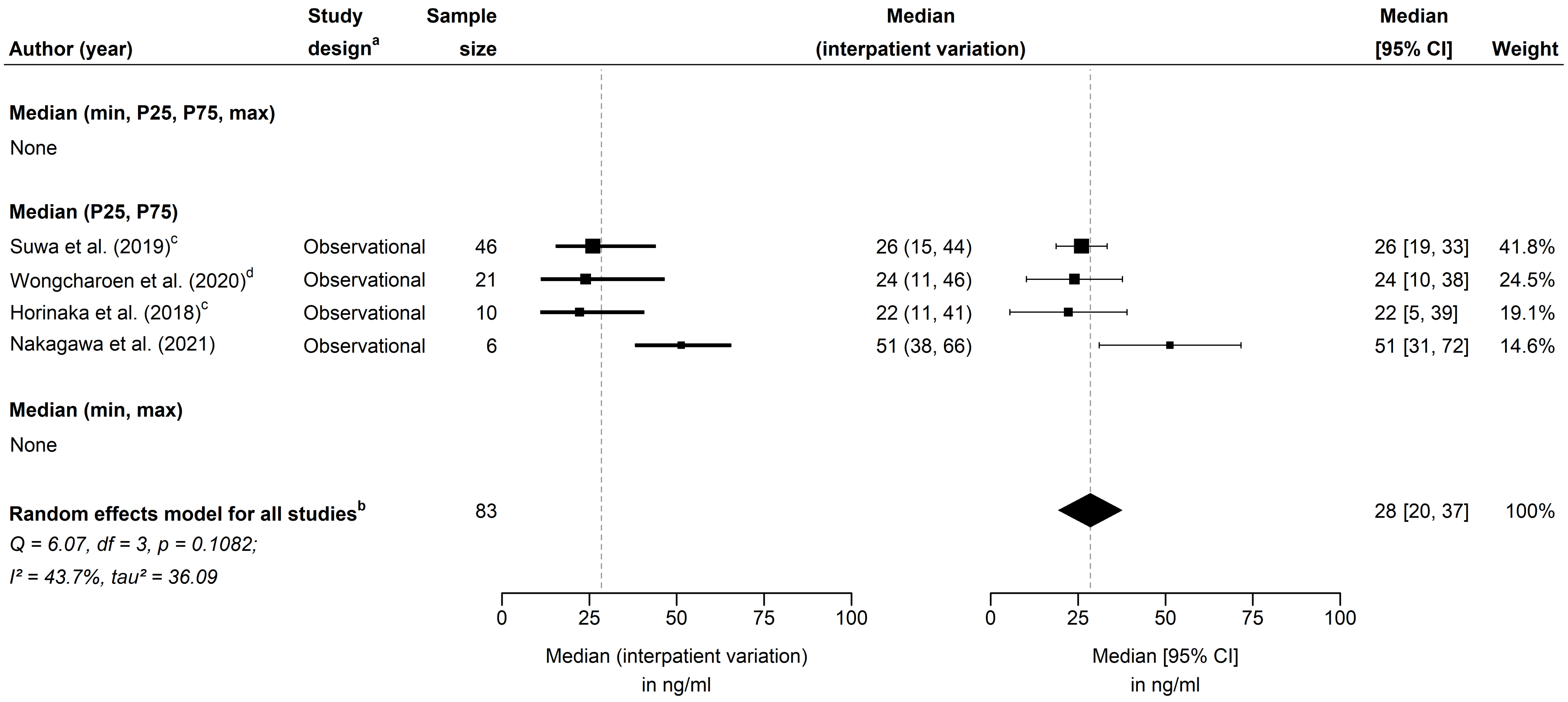

I. Rivaroxaban 10 mg once daily

[a] All analyses of interest were cross-sectional; [b] Random effects model using the quantile-estimation method;[54,57-59] [c] Simulated values were used because only the mean and standard deviation were available; [d] Simulated values were used because available parameters could not readily be included in the QE-method; [e] Percentiles were calculated directly from the original dataset if they were published by the authors of the current review.[4,12,37]

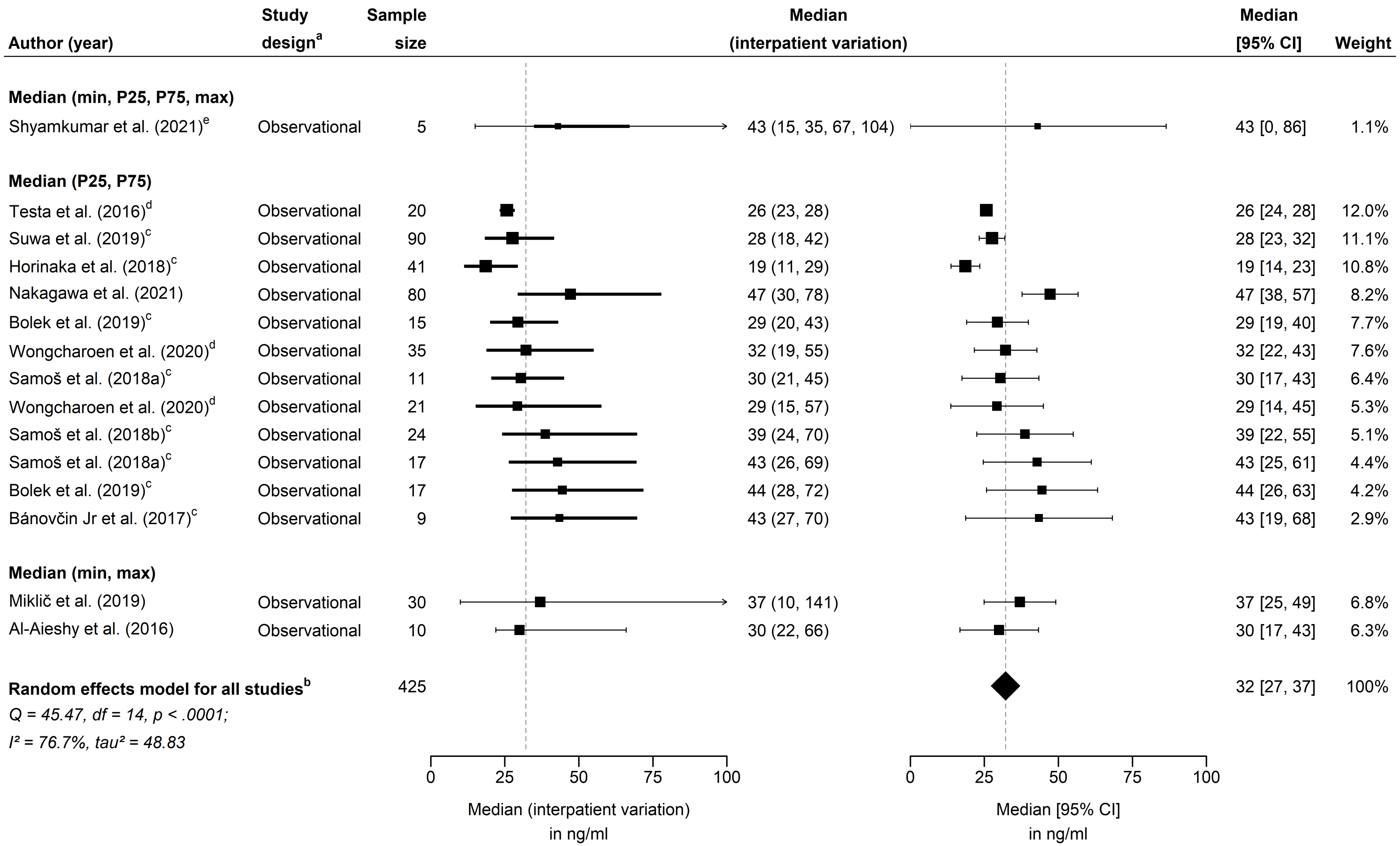

J. Rivaroxaban 15 mg once daily

[a] All analyses of interest were cross-sectional; [b] Random effects model using the quantile-estimation method;[54,57-59] [c] Simulated values were used because only the mean and standard deviation were available; [d] Simulated values were used because available parameters could not readily be included in the QE-method; [e] Percentiles were calculated directly from the original dataset if they were published by the authors of the current review.[4,12,37]

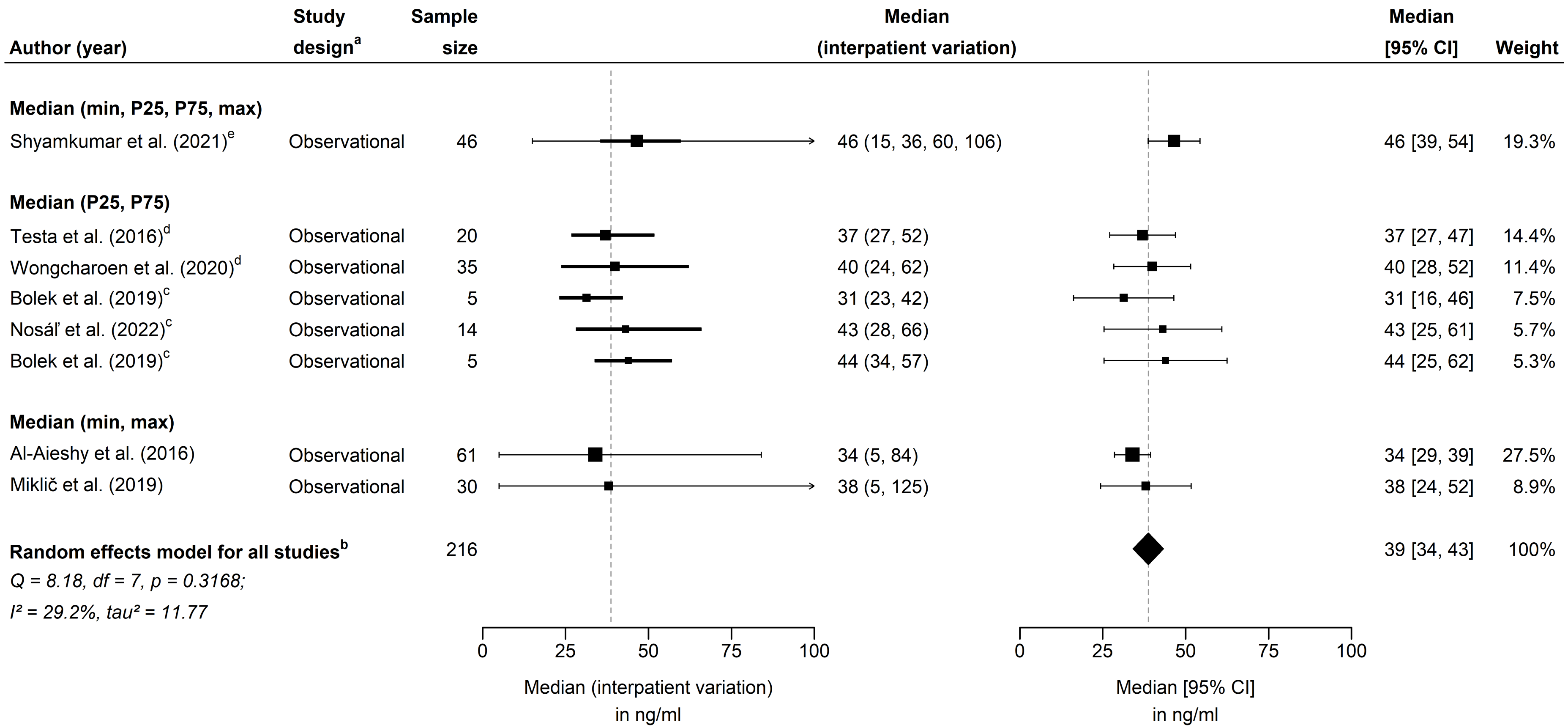

K. Rivaroxaban 20 mg once daily

[a] All analyses of interest were cross-sectional; [b] Random effects model using the quantile-estimation method;[54,57-59] [c] Simulated values were used because only the mean and standard deviation were available; [d] Simulated values were used because available parameters could not readily be included in the QE-method; [e] Percentiles were calculated directly from the original dataset if they were published by the authors of the current review.[4,12,37]

Figure S6. Estimating the pooled 10th percentile of trough levels of each direct oral anticoagulant stratified by administered dose and using the modified QE-method

The forest plots below illustrate the results of our analyses to estimate the 10th percentile of trough levels of each DOAC type, stratified by dosing regimen. The squares represent the 10th percentile values, the circles the median values, the solid bold lines the 25th to 75th percentile range, and the whiskers either the minimum to maximum value interval (left side of the plot) or the 95% of the confidence interval of the 10th percentile values (right side of the plot).

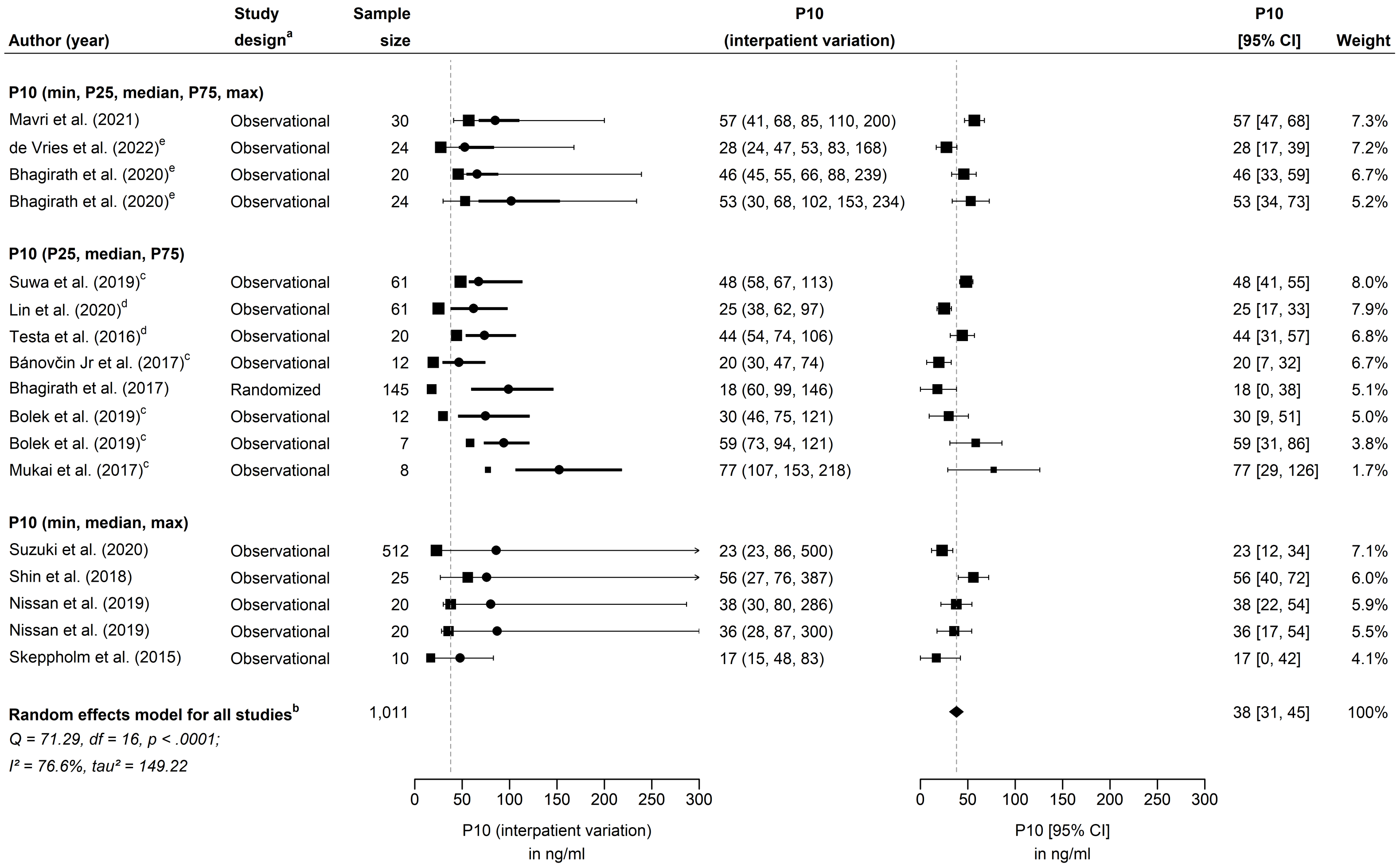

A. Apixaban 2.5 mg twice daily

[a] All analyses of interest were cross-sectional; [b] Random effects model using the quantile-estimation method;[54,57-59] [c] Simulated values were used because only the mean and standard deviation were available; [d] Simulated values were used because available parameters could not readily be included in the QE-method; [e] Percentiles were calculated directly from the original dataset if they were published by the authors of the current review.[4,12,37]

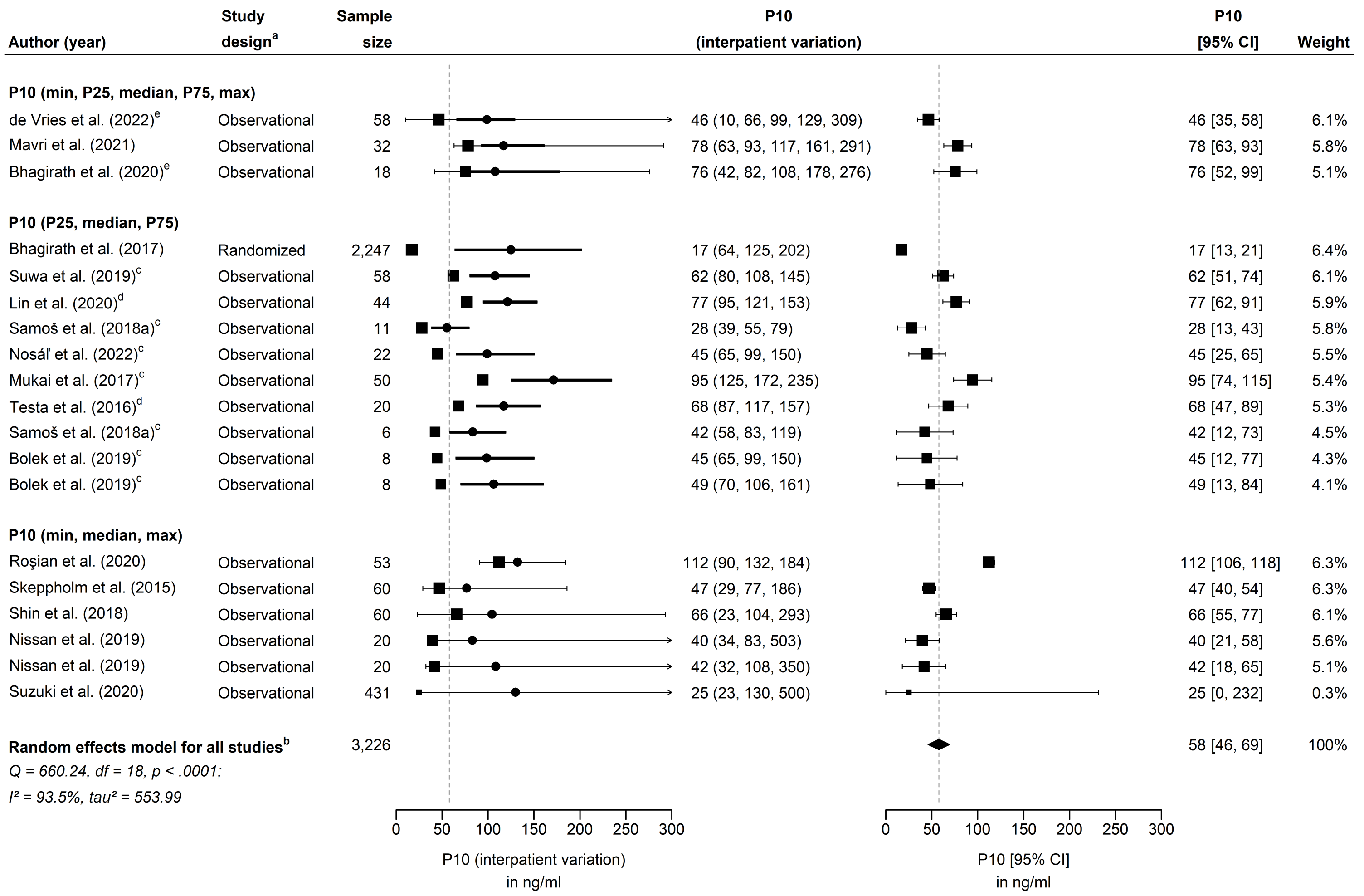

B. Apixaban 5 mg twice daily

[a] All analyses of interest were cross-sectional; [b] Random effects model using the quantile-estimation method;[54,57-59] [c] Simulated values were used because only the mean and standard deviation were available; [d] Simulated values were used because available parameters could not readily be included in the QE-method; [e] Percentiles were calculated directly from the original dataset if they were published by the authors of the current review.[4,12,37]

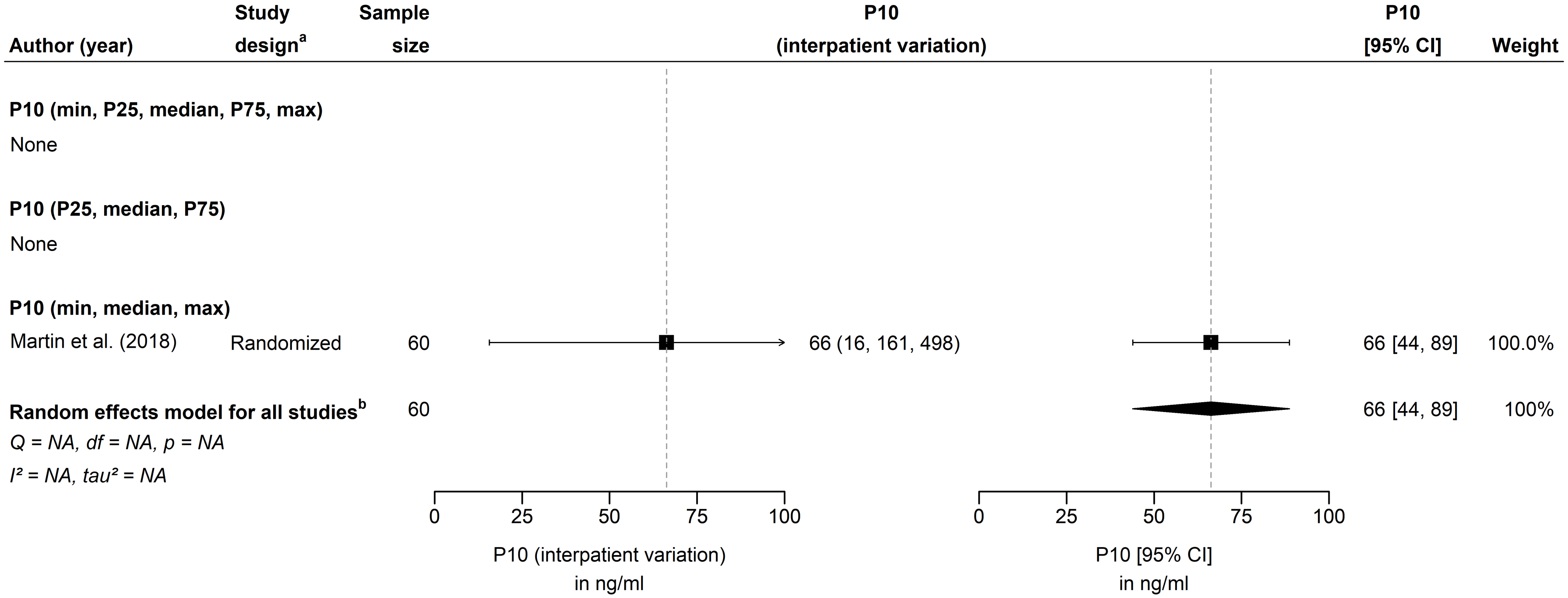

<u>C. Dabigatran 75 mg twice daily</u>

[a] All analyses of interest were cross-sectional; [b] Random effects model using the quantile-estimation method;[54,57-59] [c] Simulated values were used because only the mean and standard deviation were available; [d] Simulated values were used because available parameters could not readily be included in the QE-method; [e] Percentiles were calculated directly from the original dataset if they were published by the authors of the current review.[4,12,37]

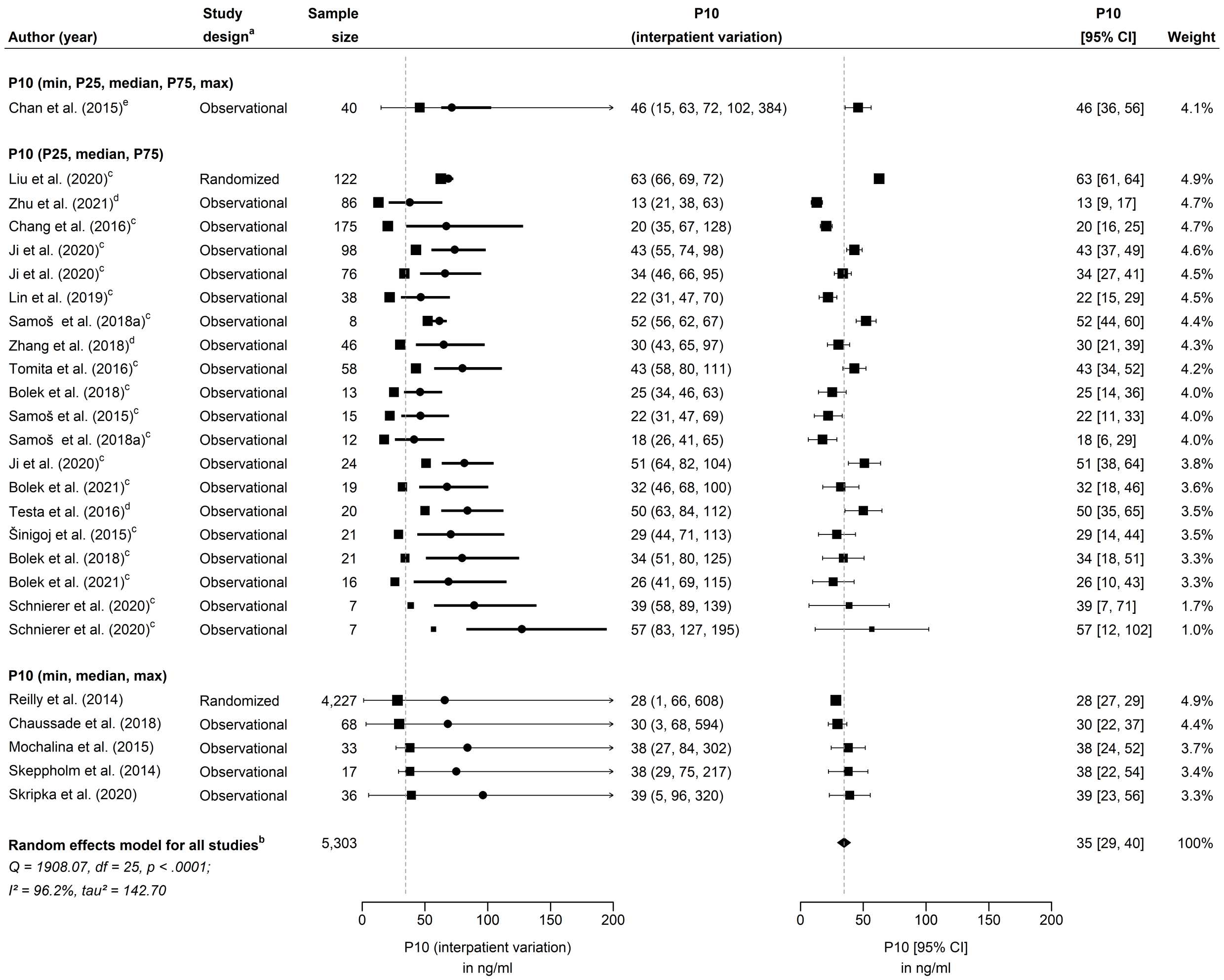

D. Dabigatran 110 mg twice daily

[a] All analyses of interest were cross-sectional; [b] Random effects model using the quantile-estimation method;[54,57-59] [c] Simulated values were used because only the mean and standard deviation were available; [d] Simulated values were used because available parameters could not readily be included in the QE-method; [e] Percentiles were calculated directly from the original dataset if they were published by the authors of the current review.[4,12,37]

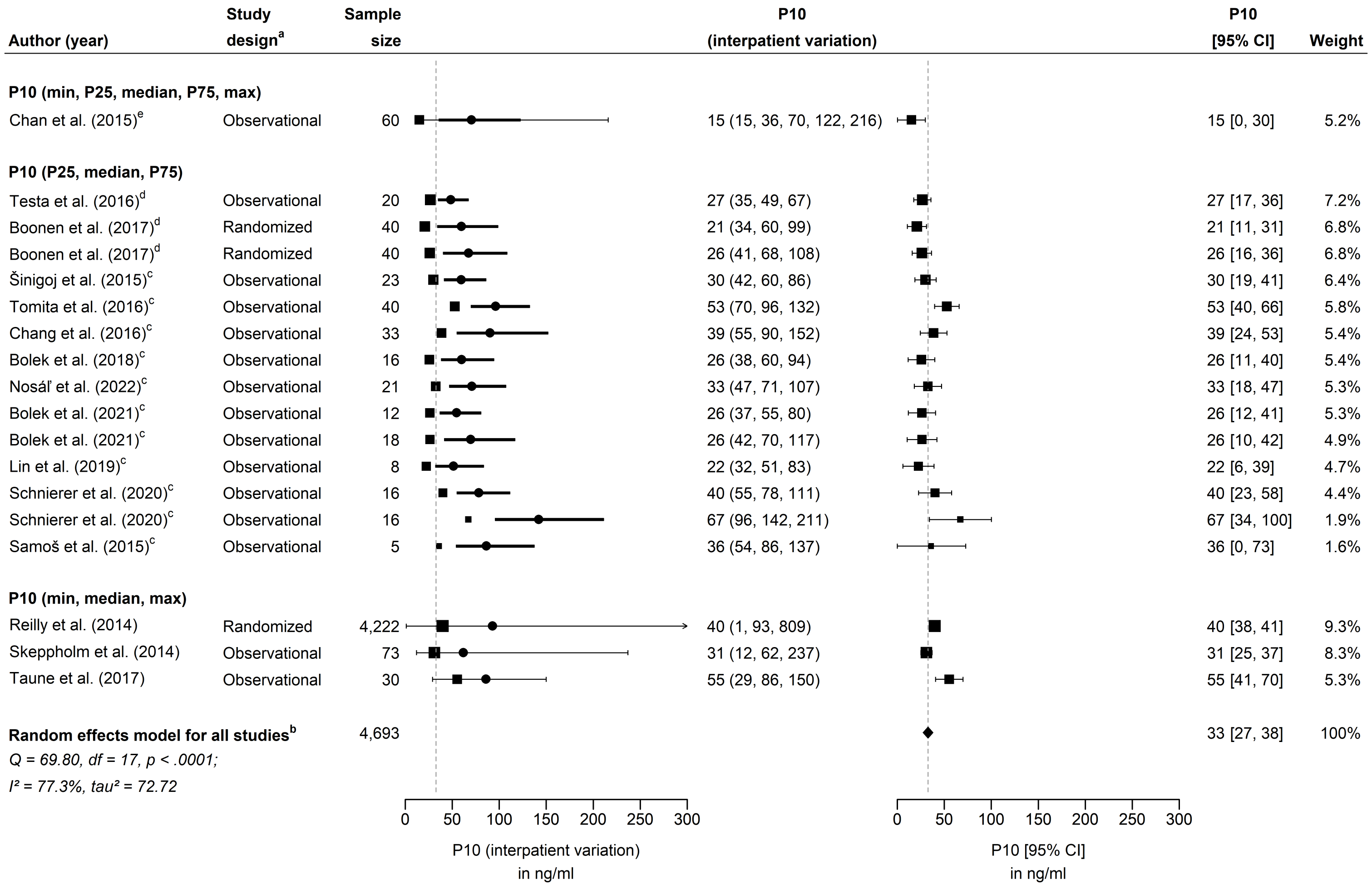

E. Dabigatran 150 mg twice daily

[a] All analyses of interest were cross-sectional; [b] Random effects model using the quantile-estimation method;[54,57-59] [c] Simulated values were used because only the mean and standard deviation were available; [d] Simulated values were used because available parameters could not readily be included in the QE-method; [e] Percentiles were calculated directly from the original dataset if they were published by the authors of the current review.[4,12,37]

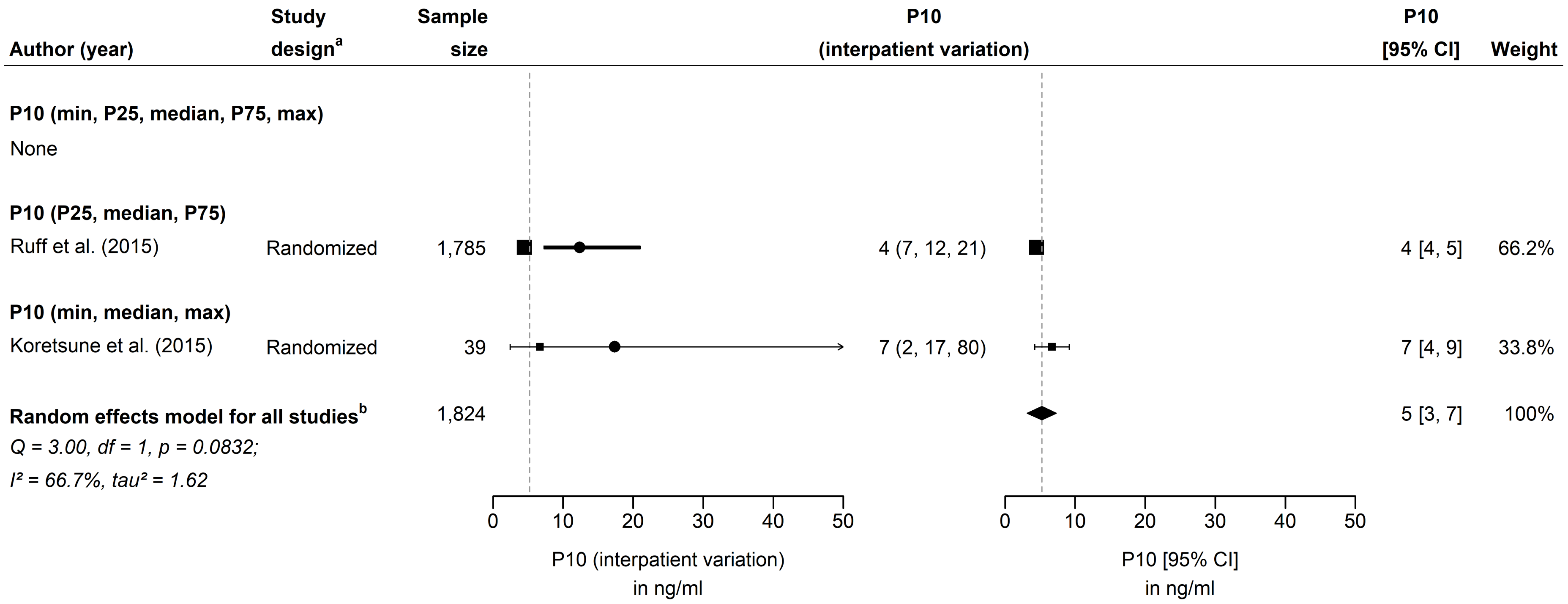

F. Edoxaban 15 mg once daily

[a] All analyses of interest were cross-sectional; [b] Random effects model using the quantile-estimation method;[54,57-59] [c] Simulated values were used because only the mean and standard deviation were available; [d] Simulated values were used because available parameters could not readily be included in the QE-method; [e] Percentiles were calculated directly from the original dataset if they were published by the authors of the current review.[4,12,37]

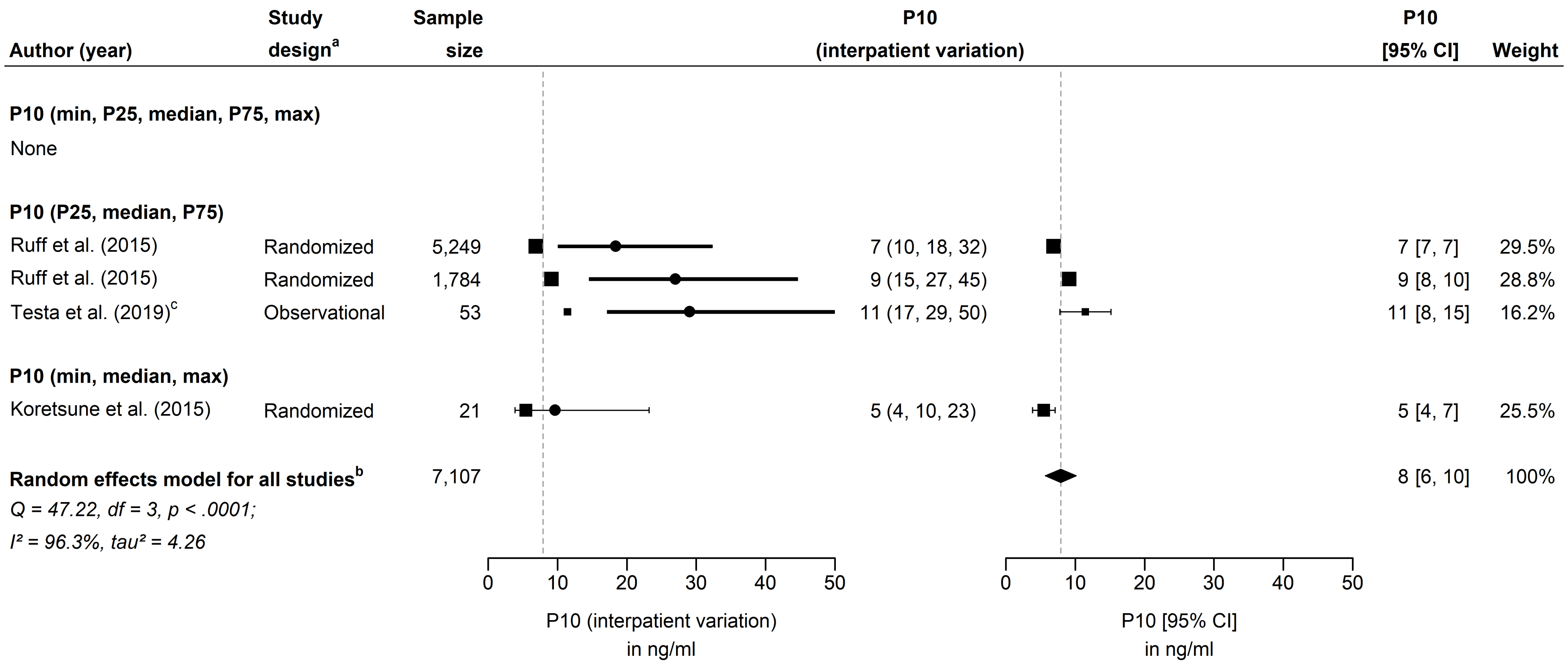

G. Edoxaban 30 mg once daily

[a] All analyses of interest were cross-sectional; [b] Random effects model using the quantile-estimation method;[54,57-59] [c] Simulated values were used because only the mean and standard deviation were available; [d] Simulated values were used because available parameters could not readily be included in the QE-method; [e] Percentiles were calculated directly from the original dataset if they were published by the authors of the current review.[4,12,37]

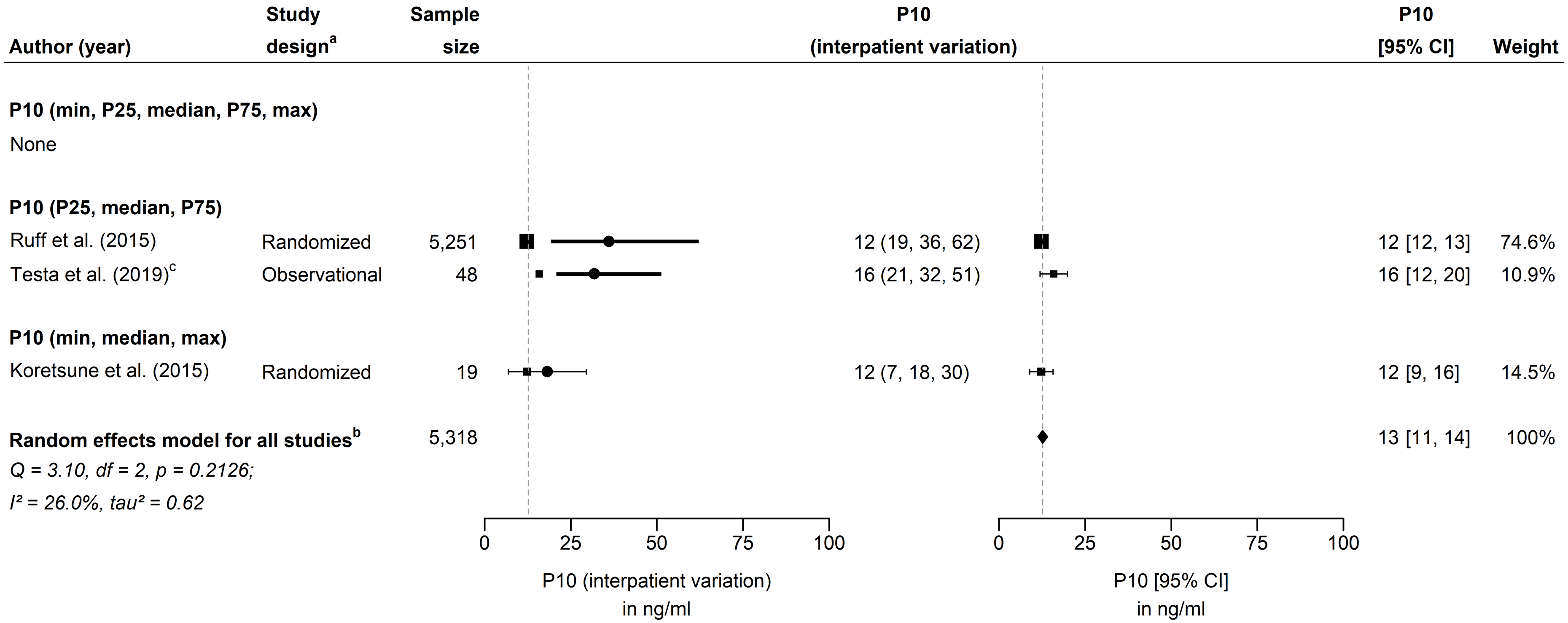

H. Edoxaban 60 mg once daily

[a] All analyses of interest were cross-sectional; [b] Random effects model using the quantile-estimation method;[54,57-59] [c] Simulated values were used because only the mean and standard deviation were available; [d] Simulated values were used because available parameters could not readily be included in the QE-method; [e] Percentiles were calculated directly from the original dataset if they were published by the authors of the current review.[4,12,37]

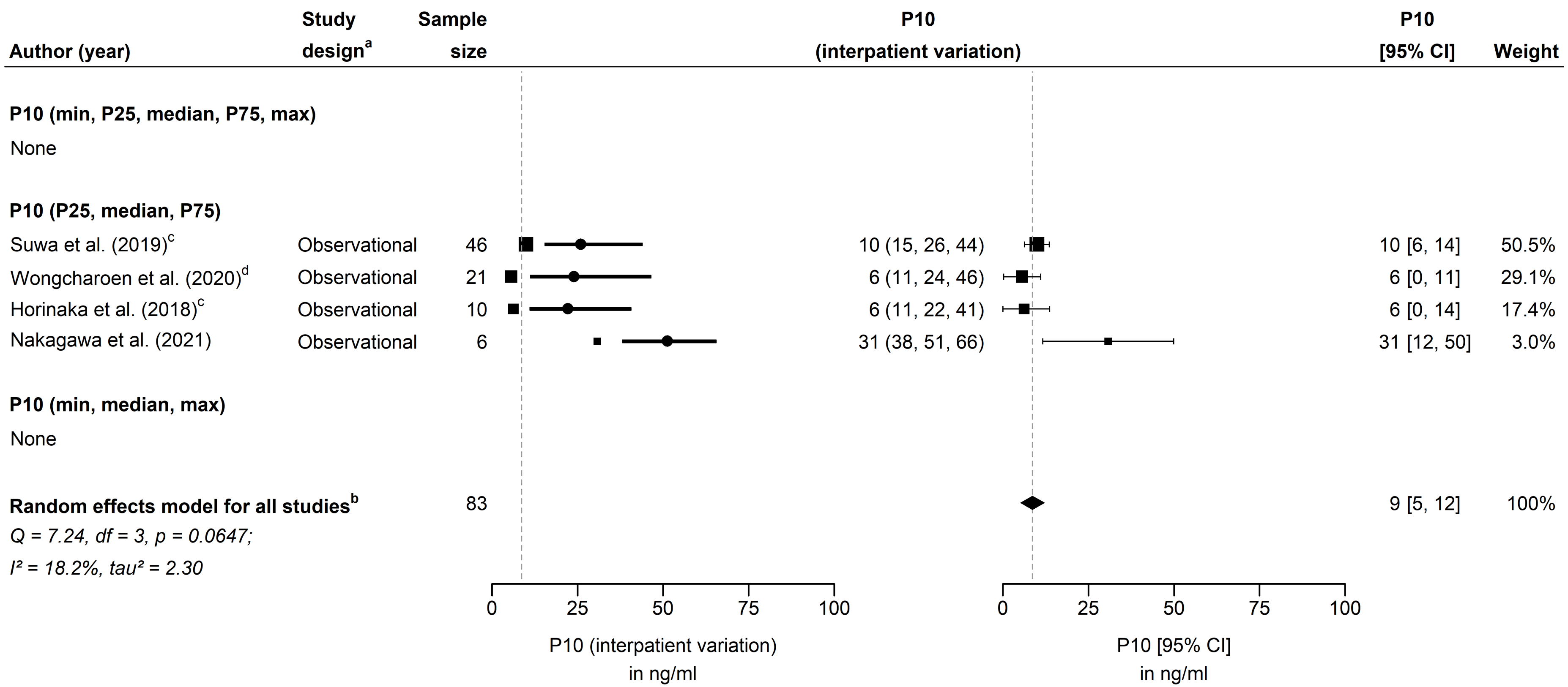

I. Rivaroxaban 10 mg once daily

[a] All analyses of interest were cross-sectional; [b] Random effects model using the quantile-estimation method;[54,57-59] [c] Simulated values were used because only the mean and standard deviation were available; [d] Simulated values were used because available parameters could not readily be included in the QE-method; [e] Percentiles were calculated directly from the original dataset if they were published by the authors of the current review.[4,12,37]

Author (year)	Study design[a]	Sample size	P10 (interpatient variation)	P10 [95% CI]	Weight
**P10 (min, P25, median, P75, max)**					
Shyamkumar et al. (2021)[e]	Observational	5	23 (15, 35, 43, 67, 104)	23 [5, 41]	2.0%
**P10 (P25, median, P75)**					
Testa et al. (2016)[d]	Observational	20	22 (23, 26, 28)	22 [20, 24]	10.4%
Suwa et al. (2019)[c]	Observational	90	13 (18, 28, 42)	13 [10, 16]	10.1%
Horinaka et al. (2018)[c]	Observational	41	8 (11, 19, 29)	8 [5, 10]	10.0%
Nakagawa et al. (2021)	Observational	80	20 (30, 47, 78)	20 [16, 25]	8.3%
Wongcharoen et al. (2020)[d]	Observational	35	12 (19, 32, 55)	12 [7, 17]	8.0%
Wongcharoen et al. (2020)[d]	Observational	21	9 (15, 29, 57)	9 [3, 15]	7.4%
Bolek et al. (2019)[c]	Observational	15	14 (20, 29, 43)	14 [7, 21]	6.5%
Samoš et al. (2018b)[c]	Observational	24	19 (24, 39, 70)	19 [12, 26]	6.5%
Samoš et al. (2018a)[c]	Observational	11	15 (21, 30, 45)	15 [6, 23]	5.5%
Samoš et al. (2018a)[c]	Observational	17	17 (26, 43, 69)	17 [7, 27]	4.6%
Bolek et al. (2019)[c]	Observational	17	18 (28, 44, 72)	18 [8, 28]	4.4%
Bánovčin Jr et al. (2017)[c]	Observational	9	18 (27, 43, 70)	18 [4, 32]	2.9%
**P10 (min, median, max)**					
Miklič et al. (2019)	Observational	30	17 (10, 37, 141)	17 [11, 23]	7.3%
Al-Aieshy et al. (2016)	Observational	10	22 (22, 30, 66)	22 [14, 30]	6.1%
**Random effects model for all studies[b]**		425		16 [13, 19]	100%

Q = 82.72, df = 14, p < .0001;
I² = 75.1%, tau² = 18.09

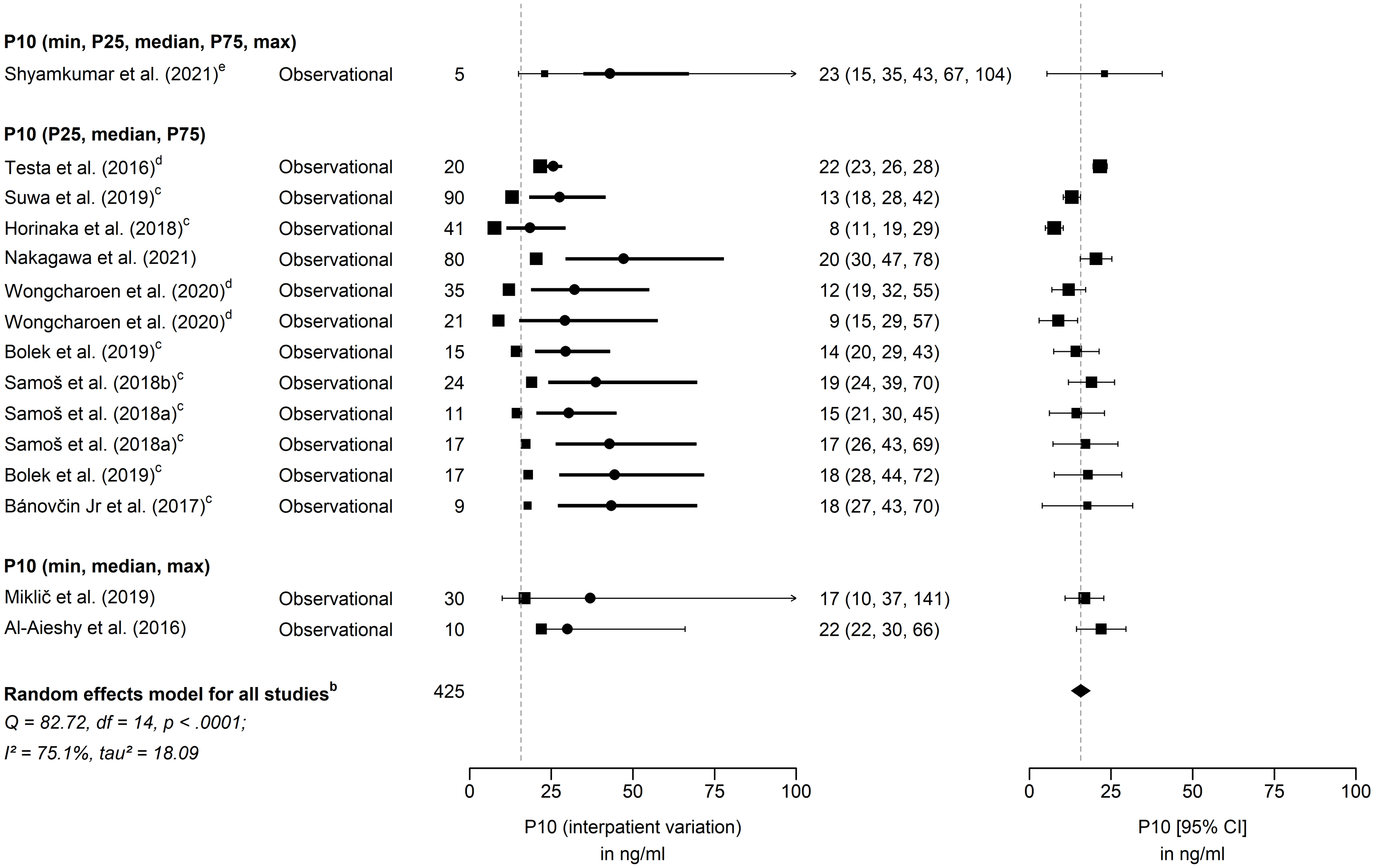

J. Rivaroxaban 15 mg once daily

[a] All analyses of interest were cross-sectional; [b] Random effects model using the quantile-estimation method;[54,57-59] [c] Simulated values were used because only the mean and standard deviation were available; [d] Simulated values were used because available parameters could not readily be included in the QE-method; [e] Percentiles were calculated directly from the original dataset if they were published by the authors of the current review.[4,12,37]

Author (year)	Study design[a]	Sample size	P10 (interpatient variation)	P10 [95% CI]	Weight
**P10 (min, P25, median, P75, max)**					
Shyamkumar et al. (2021)[e]	Observational	46	27 (15, 36, 46, 60, 106)	27 [22, 33]	16.6%
**P10 (P25, median, P75)**					
Testa et al. (2016)[d]	Observational	20	21 (27, 37, 52)	21 [15, 28]	14.8%
Wongcharoen et al. (2020)[d]	Observational	35	14 (24, 40, 62)	14 [7, 22]	13.6%
Nosáľ et al. (2022)[c]	Observational	14	19 (28, 43, 66)	19 [8, 30]	9.0%
Bolek et al. (2019)[c]	Observational	5	18 (23, 31, 42)	18 [6, 30]	8.1%
Bolek et al. (2019)[c]	Observational	5	27 (34, 44, 57)	27 [11, 42]	5.5%
**P10 (min, median, max)**					
Al-Aieshy et al. (2016)	Observational	61	13 (5, 34, 84)	13 [8, 18]	17.9%
Miklič et al. (2019)	Observational	30	18 (5, 38, 125)	18 [11, 25]	14.4%
**Random effects model for all studies[b]**		216		19 [15, 23]	100%

Q = 16.84, df = 7, p = 0.0184;

I² = 55.7%, tau² = 18.03

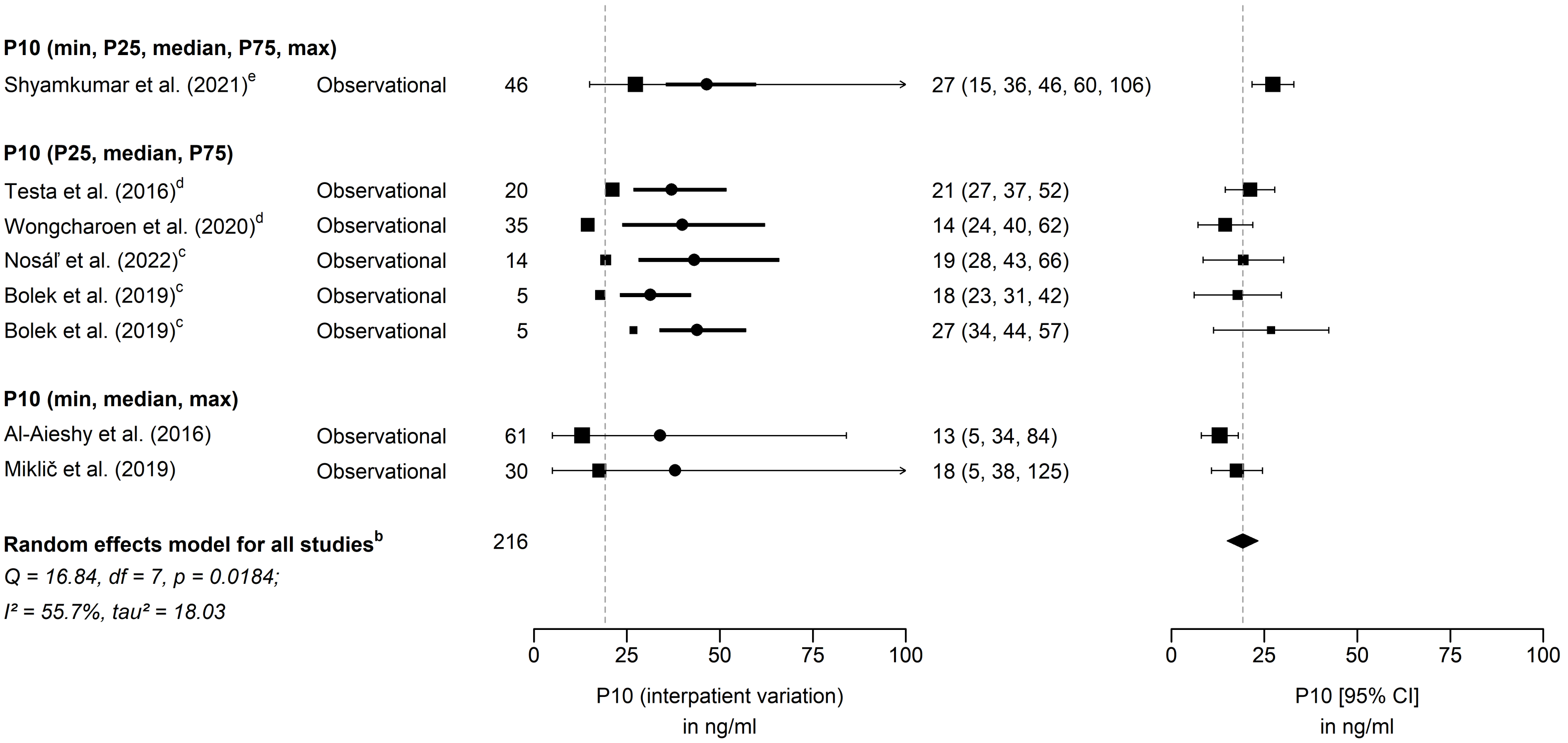

K. Rivaroxaban 20 mg once daily

[a] All analyses of interest were cross-sectional; [b] Random effects model using the quantile-estimation method;[54,57-59] [c] Simulated values were used because only the mean and standard deviation were available; [d] Simulated values were used because available parameters could not readily be included in the QE-method; [e] Percentiles were calculated directly from the original dataset if they were published by the authors of the current review.[4,12,37]

Figure S7. Estimating the pooled 90th percentile of trough levels of each direct oral anticoagulant stratified by administered dose and using the modified QE-method

The forest plots below illustrate the results of our analyses to estimate the 90th percentile of trough levels of each DOAC type, stratified by dosing regimen. The squares represent the 90th percentile values, the circles the median values, the solid bold lines the 25th to 75th percentile range, and the whiskers either the minimum to maximum value interval (left side of the plot) or the 95% of the confidence interval of the 90th percentile values (right side of the plot).

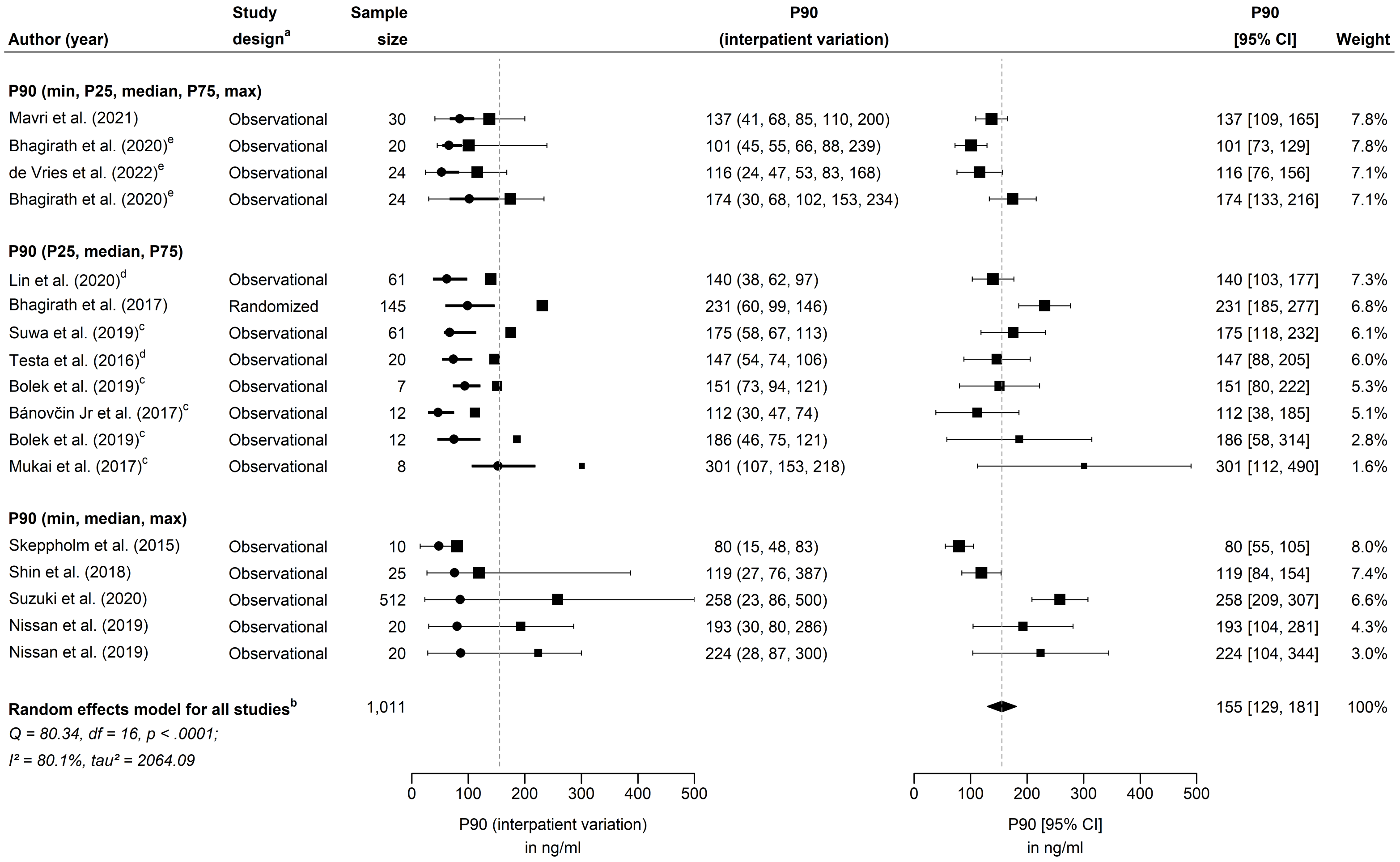

A. Apixaban 2.5 mg twice daily

[a] All analyses of interest were cross-sectional; [b] Random effects model using the quantile-estimation method;[54,57-59] [c] Simulated values were used because only the mean and standard deviation were available; [d] Simulated values were used because available parameters could not readily be included in the QE-method; [e] Percentiles were calculated directly from the original dataset if they were published by the authors of the current review.[4,12,37]

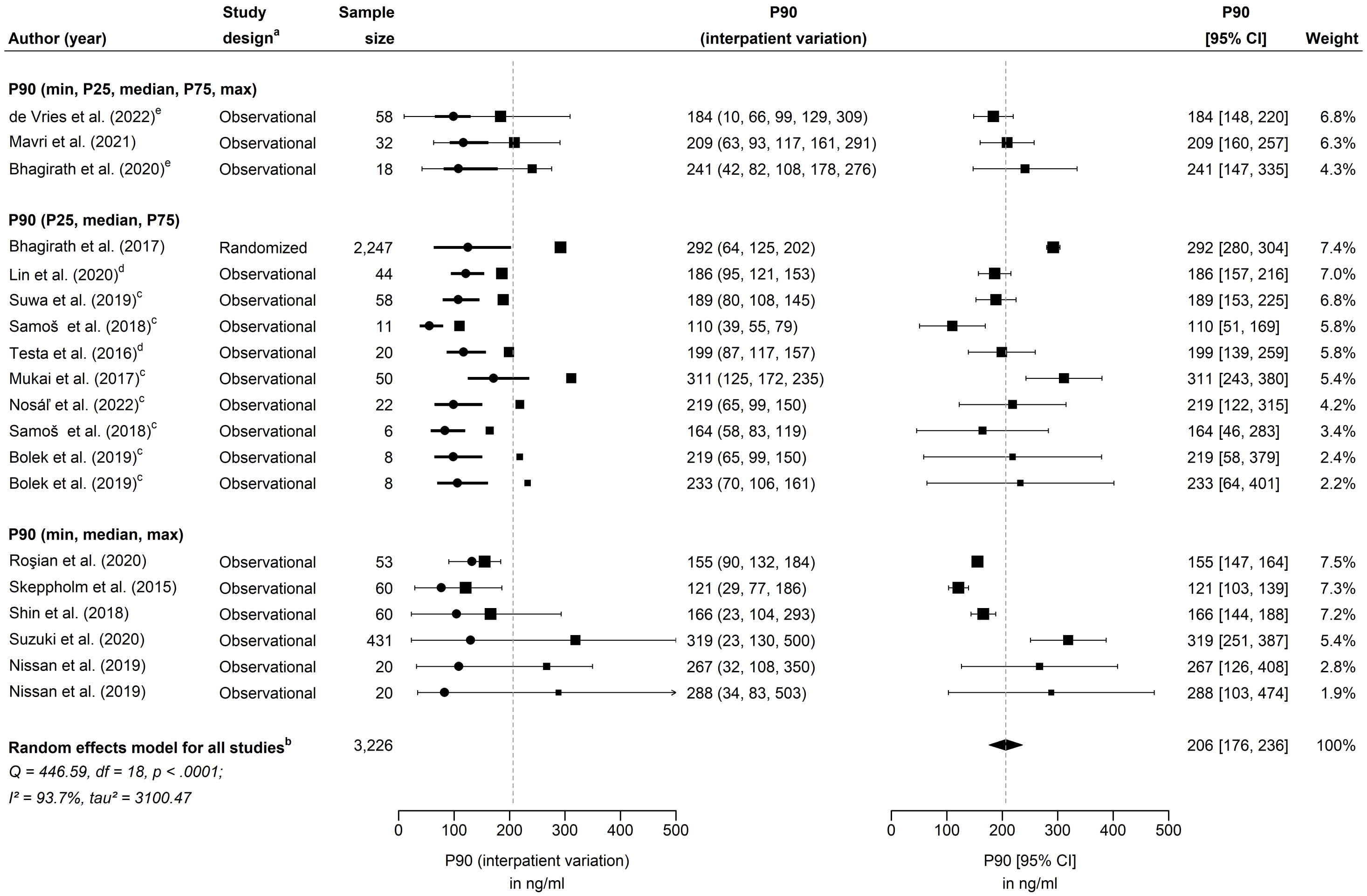

B. Apixaban 5 mg twice daily

[a] All analyses of interest were cross-sectional; [b] Random effects model using the quantile-estimation method;[54,57-59] [c] Simulated values were used because only the mean and standard deviation were available; [d] Simulated values were used because available parameters could not readily be included in the QE-method; [e] Percentiles were calculated directly from the original dataset if they were published by the authors of the current review.[4,12,37]

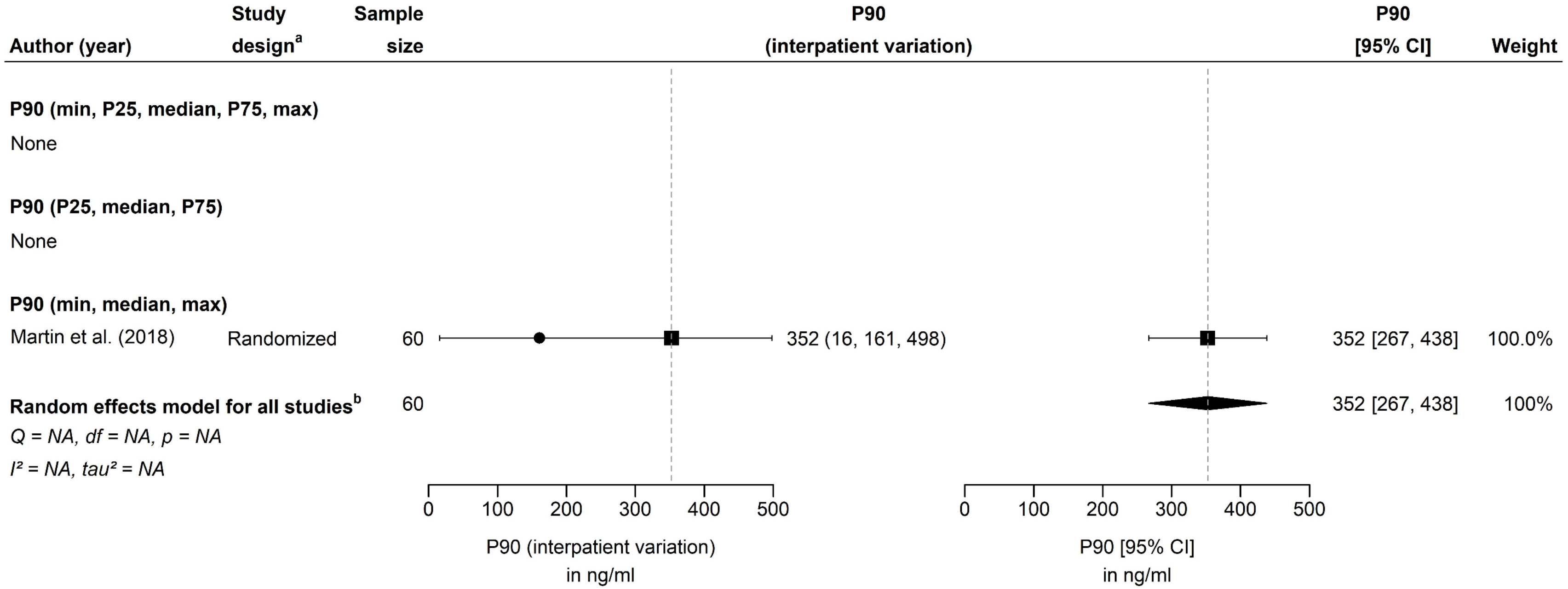

C. Dabigatran 75 mg twice daily

[a] All analyses of interest were cross-sectional; [b] Random effects model using the quantile-estimation method;[54,57-59] [c] Simulated values were used because only the mean and standard deviation were available; [d] Simulated values were used because available parameters could not readily be included in the QE-method; [e] Percentiles were calculated directly from the original dataset if they were published by the authors of the current review.[4,12,37]

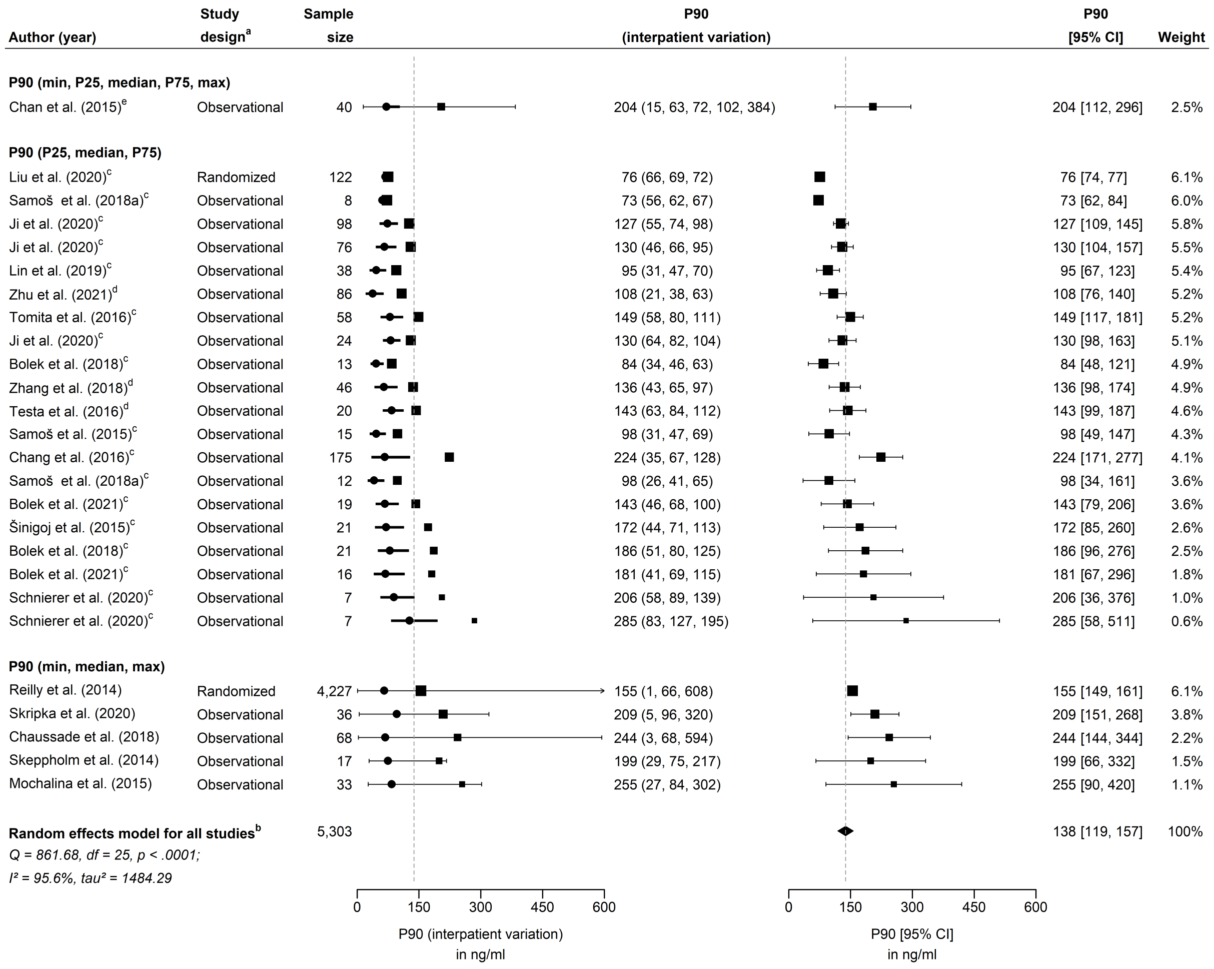

D. Dabigatran 110 mg twice daily

[a] All analyses of interest were cross-sectional; [b] Random effects model using the quantile-estimation method;[54,57-59] [c] Simulated values were used because only the mean and standard deviation were available; [d] Simulated values were used because available parameters could not readily be included in the QE-method; [e] Percentiles were calculated directly from the original dataset if they were published by the authors of the current review.[4,12,37]

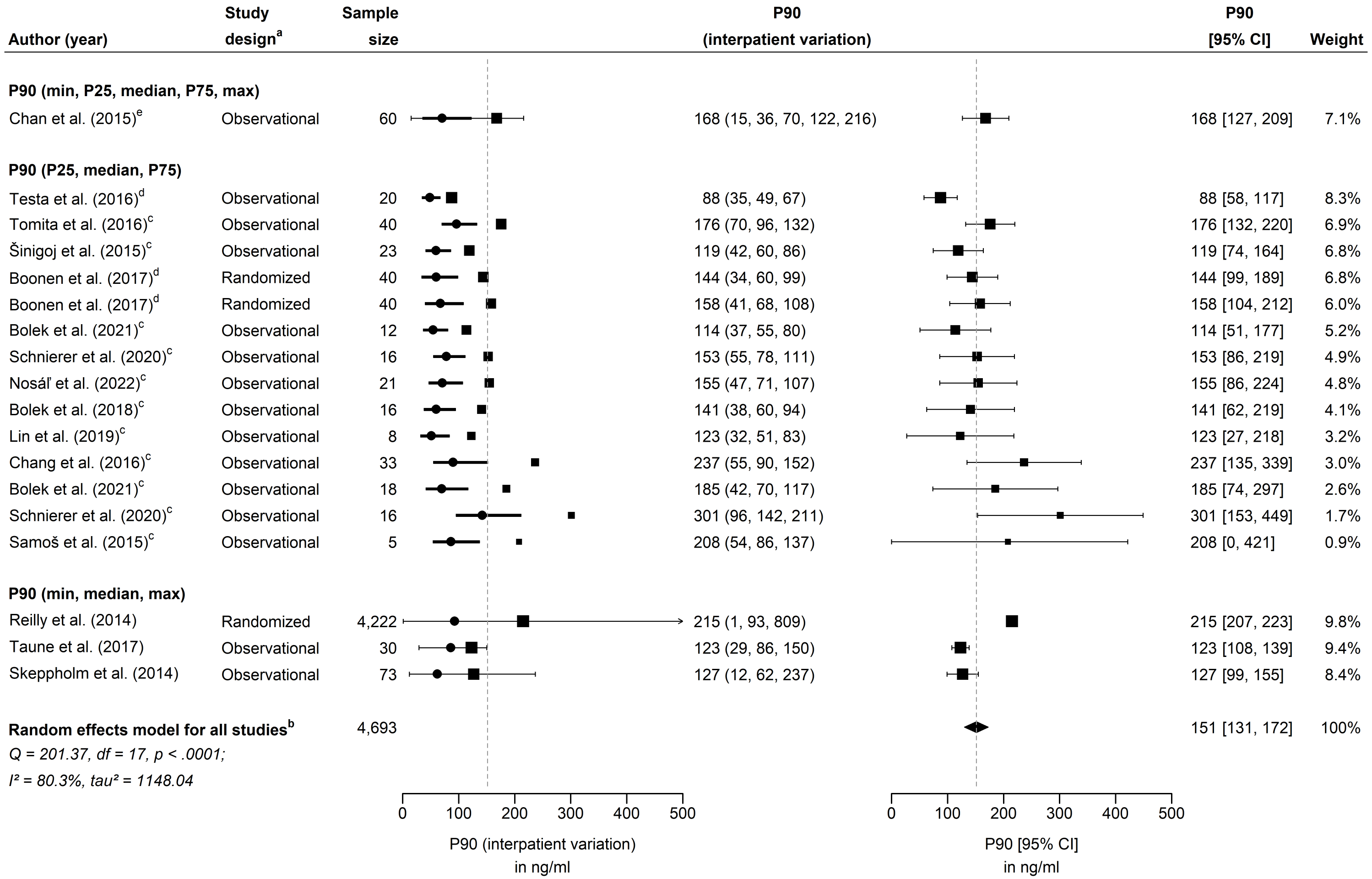

E. Dabigatran 150 mg twice daily

[a] All analyses of interest were cross-sectional; [b] Random effects model using the quantile-estimation method;[54,57-59] [c] Simulated values were used because only the mean and standard deviation were available; [d] Simulated values were used because available parameters could not readily be included in the QE-method; [e] Percentiles were calculated directly from the original dataset if they were published by the authors of the current review.[4,12,37]

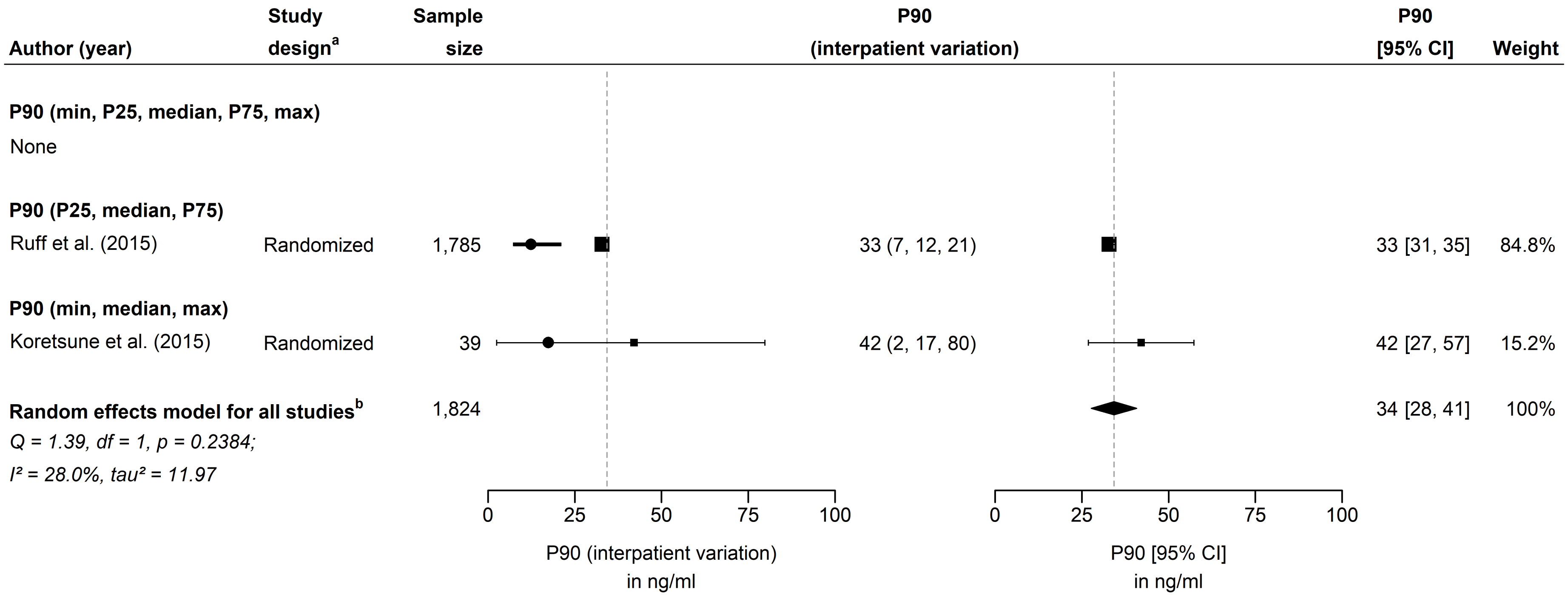

F. Edoxaban 15 mg once daily

[a] All analyses of interest were cross-sectional; [b] Random effects model using the quantile-estimation method;[54,57-59] [c] Simulated values were used because only the mean and standard deviation were available; [d] Simulated values were used because available parameters could not readily be included in the QE-method; [e] Percentiles were calculated directly from the original dataset if they were published by the authors of the current review.[4,12,37]

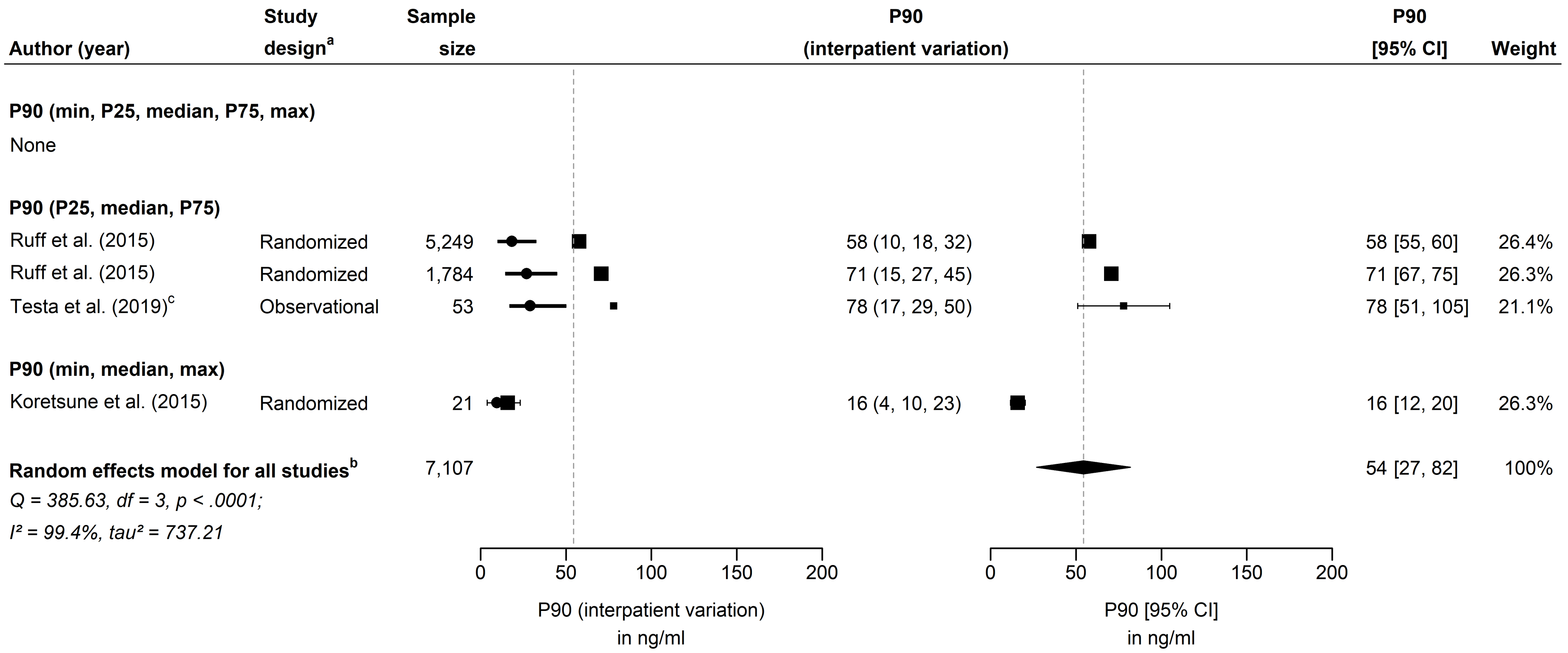

G. Edoxaban 30 mg once daily

[a] All analyses of interest were cross-sectional; [b] Random effects model using the quantile-estimation method;[54,57-59] [c] Simulated values were used because only the mean and standard deviation were available; [d] Simulated values were used because available parameters could not readily be included in the QE-method; [e] Percentiles were calculated directly from the original dataset if they were published by the authors of the current review.[4,12,37]

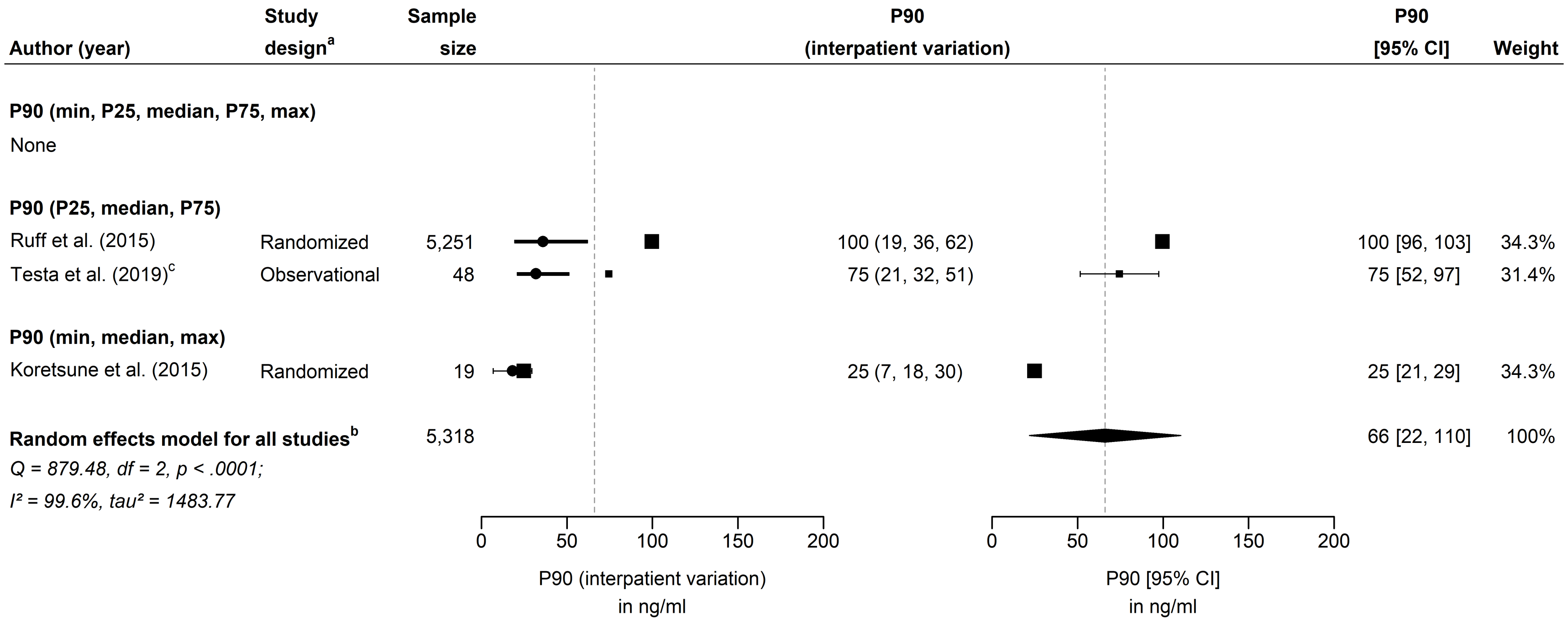

H. Edoxaban 60 mg once daily

[a] All analyses of interest were cross-sectional; [b] Random effects model using the quantile-estimation method;[54,57-59] [c] Simulated values were used because only the mean and standard deviation were available; [d] Simulated values were used because available parameters could not readily be included in the QE-method; [e] Percentiles were calculated directly from the original dataset if they were published by the authors of the current review.[4,12,37]

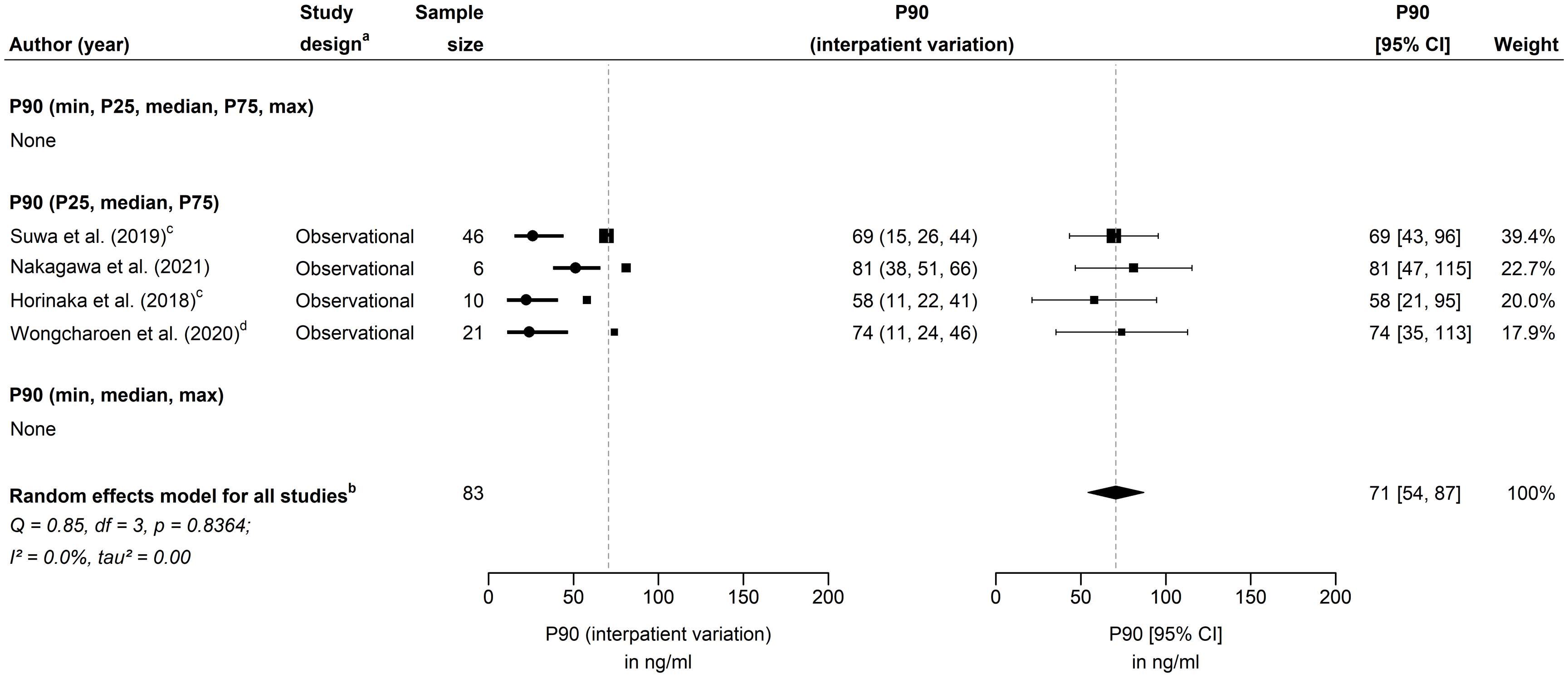

<u>I. Rivaroxaban 10 mg once daily</u>

[a] All analyses of interest were cross-sectional; [b] Random effects model using the quantile-estimation method;[54,57-59] [c] Simulated values were used because only the mean and standard deviation were available; [d] Simulated values were used because available parameters could not readily be included in the QE-method; [e] Percentiles were calculated directly from the original dataset if they were published by the authors of the current review.[4,12,37]

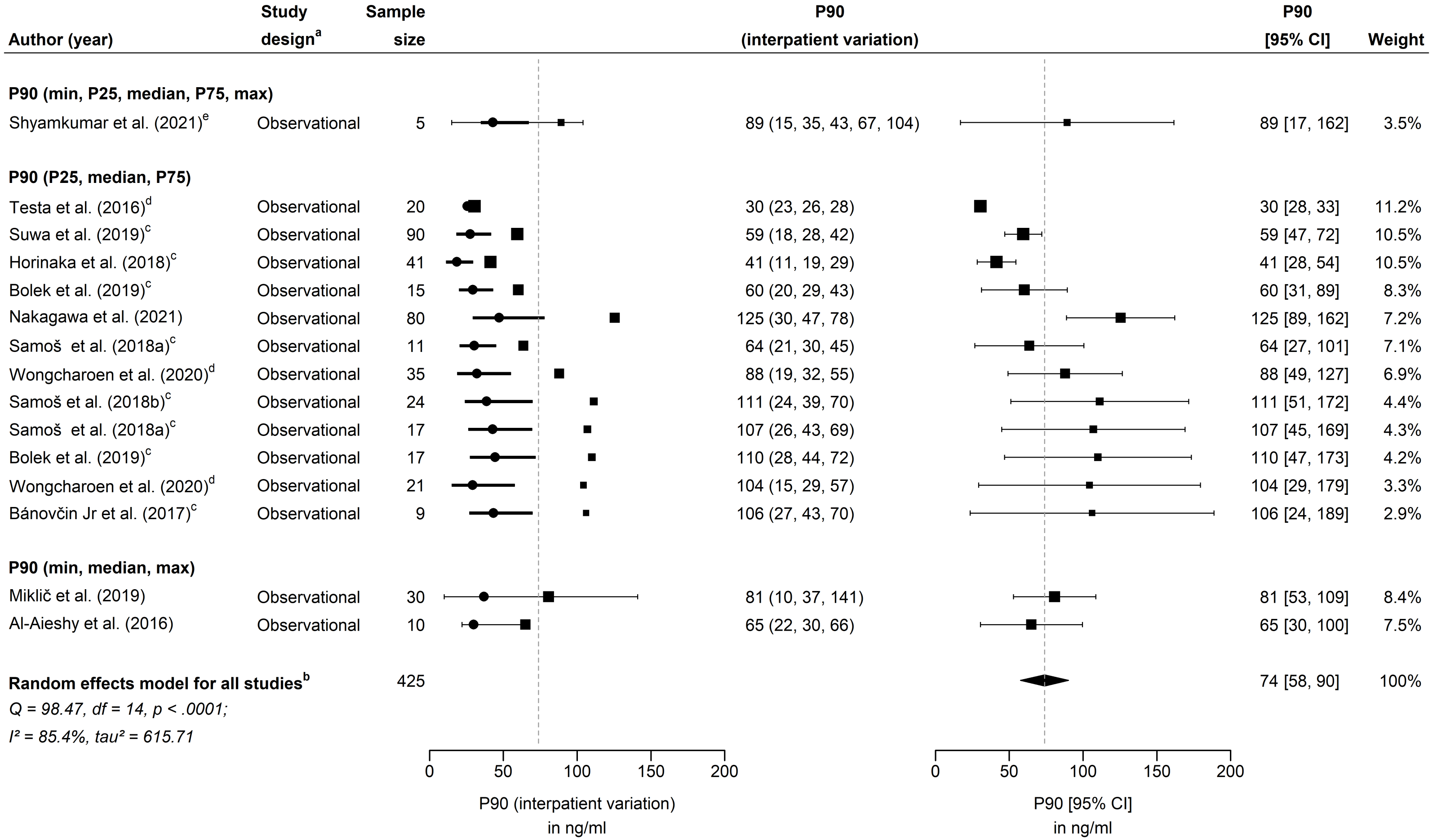

J. Rivaroxaban 15 mg once daily

[a] All analyses of interest were cross-sectional; [b] Random effects model using the quantile-estimation method;[54,57-59] [c] Simulated values were used because only the mean and standard deviation were available; [d] Simulated values were used because available parameters could not readily be included in the QE-method; [e] Percentiles were calculated directly from the original dataset if they were published by the authors of the current review.[4,12,37]

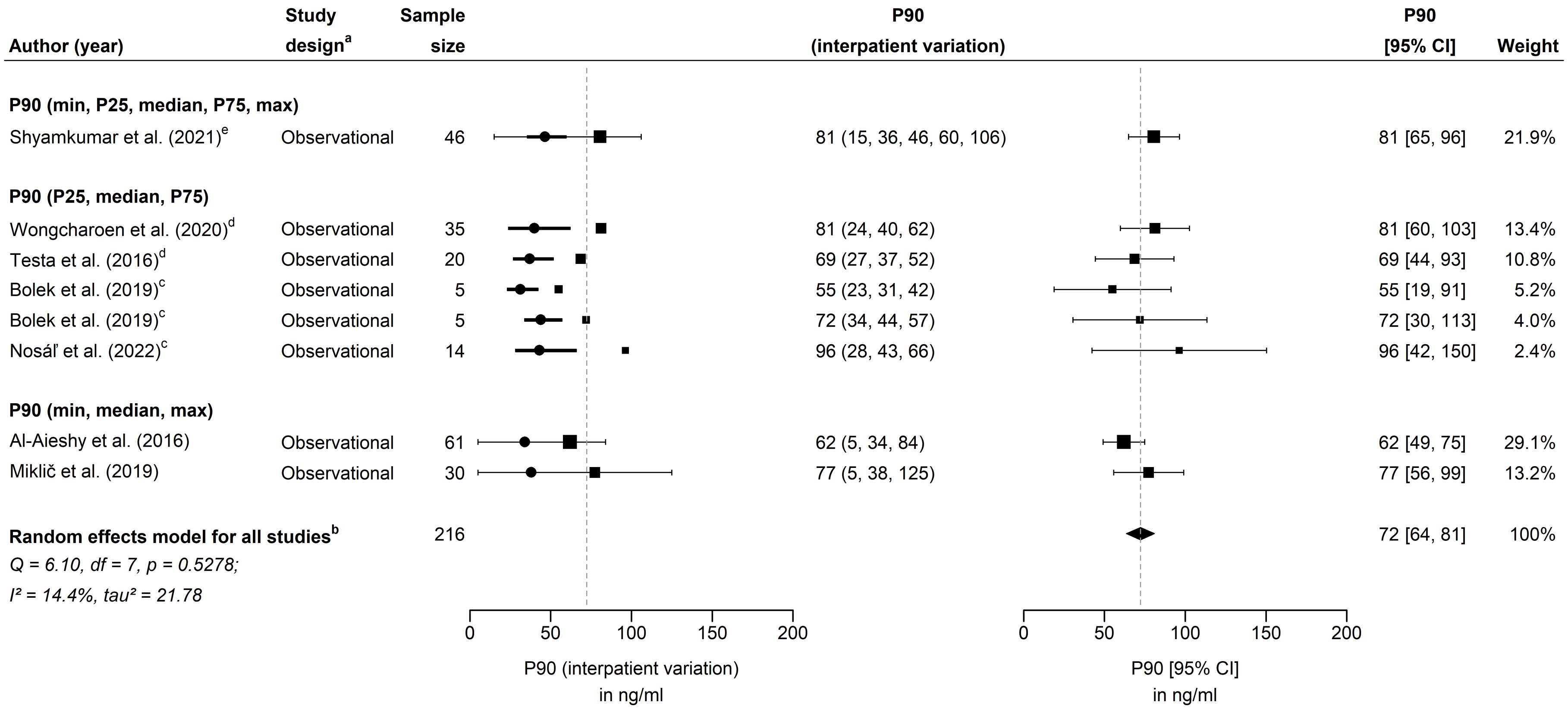

K. Rivaroxaban 20 mg once daily

[a] All analyses of interest were cross-sectional; [b] Random effects model using the quantile-estimation method;[54,57-59] [c] Simulated values were used because only the mean and standard deviation were available; [d] Simulated values were used because available parameters could not readily be included in the QE-method; [e] Percentiles were calculated directly from the original dataset if they were published by the authors of the current review.[4,12,37]

Figure S8. Estimating the pooled median peak level of each direct oral anticoagulant stratified by administered dose

The forest plots below illustrate the results of our analyses to estimate the median peak level of each DOAC type, stratified by dosing regimen. The squares represent the median values, the solid bold lines the 25th to 75th percentile range, and the whiskers either the minimum to maximum value interval (left side of the plot) or the 95% of the confidence interval of the median values (right side of the plot).

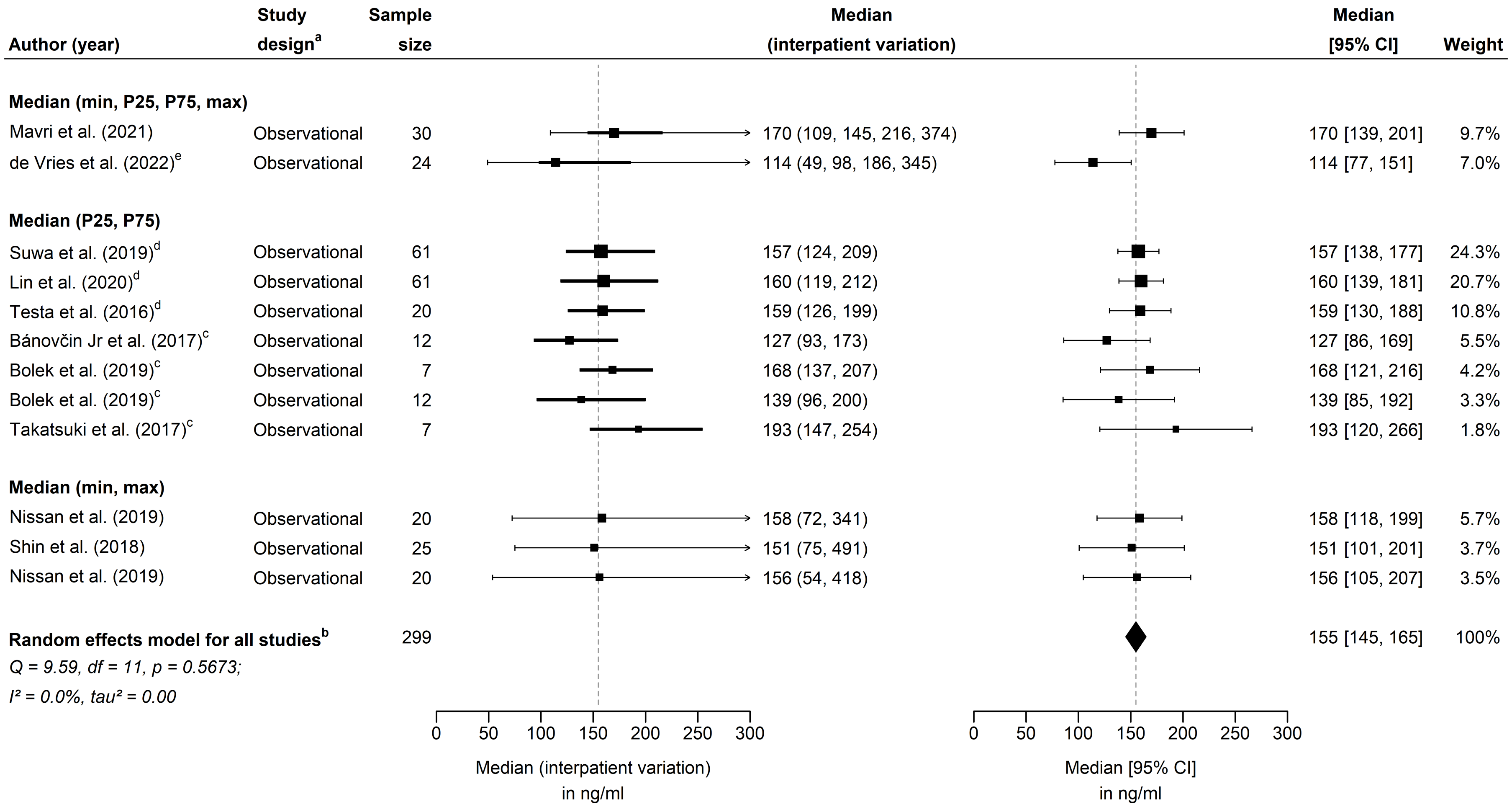

A. Apixaban 2.5 mg twice daily

[a] All analyses of interest were cross-sectional; [b] Random effects model using the quantile-estimation method;[54,57-59] [c] Simulated values were used because only the mean and standard deviation were available; [d] Simulated values were used because available parameters could not readily be included in the QE-method; [e] Percentiles were calculated directly from the original dataset if they were published by the authors of the current review.[4,12,37]

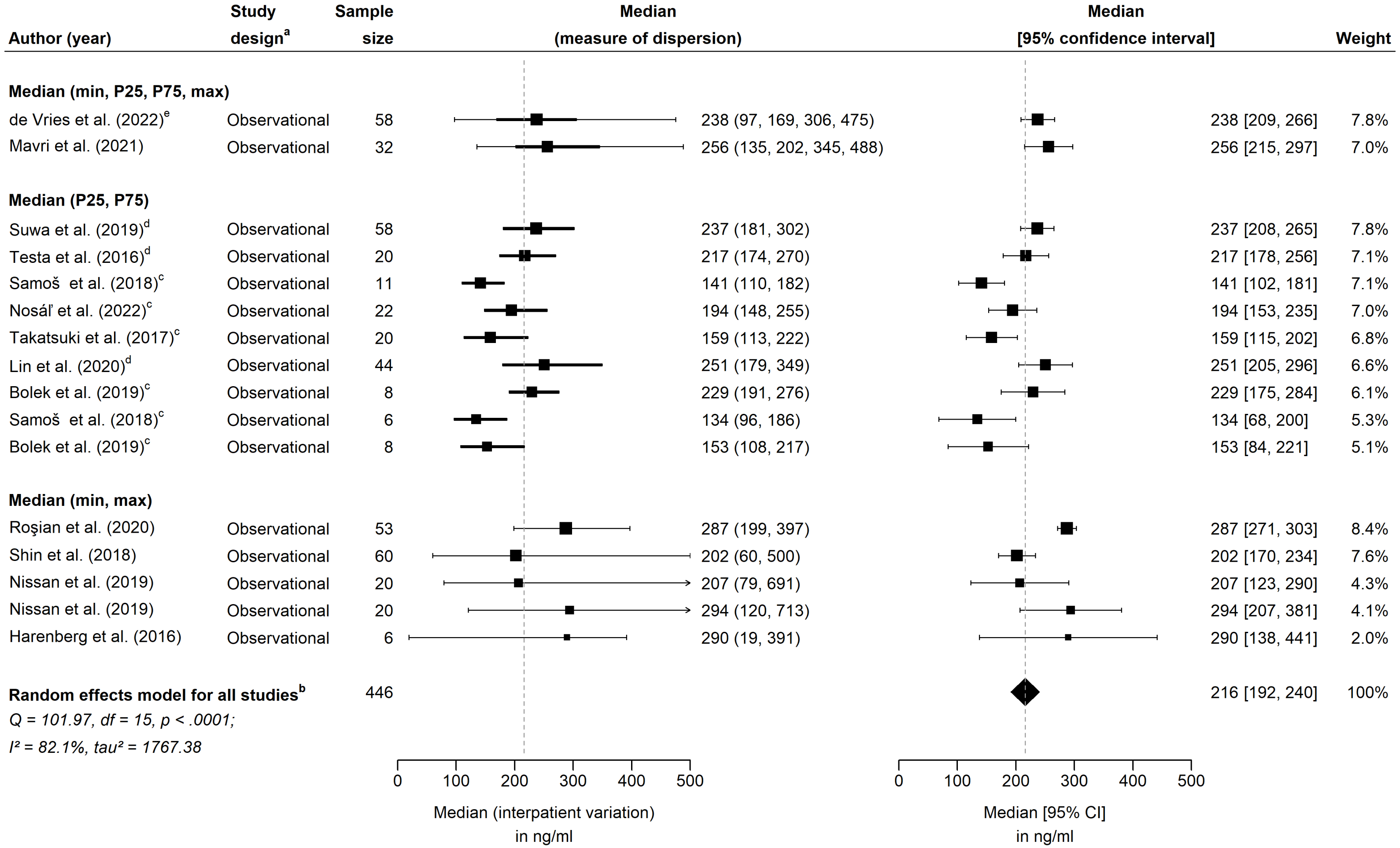

B. Apixaban 5 mg twice daily

[a] All analyses of interest were cross-sectional; [b] Random effects model using the quantile-estimation method;[54,57-59] [c] Simulated values were used because only the mean and standard deviation were available; [d] Simulated values were used because available parameters could not readily be included in the QE-method; [e] Percentiles were calculated directly from the original dataset if they were published by the authors of the current review.[4,12,37]

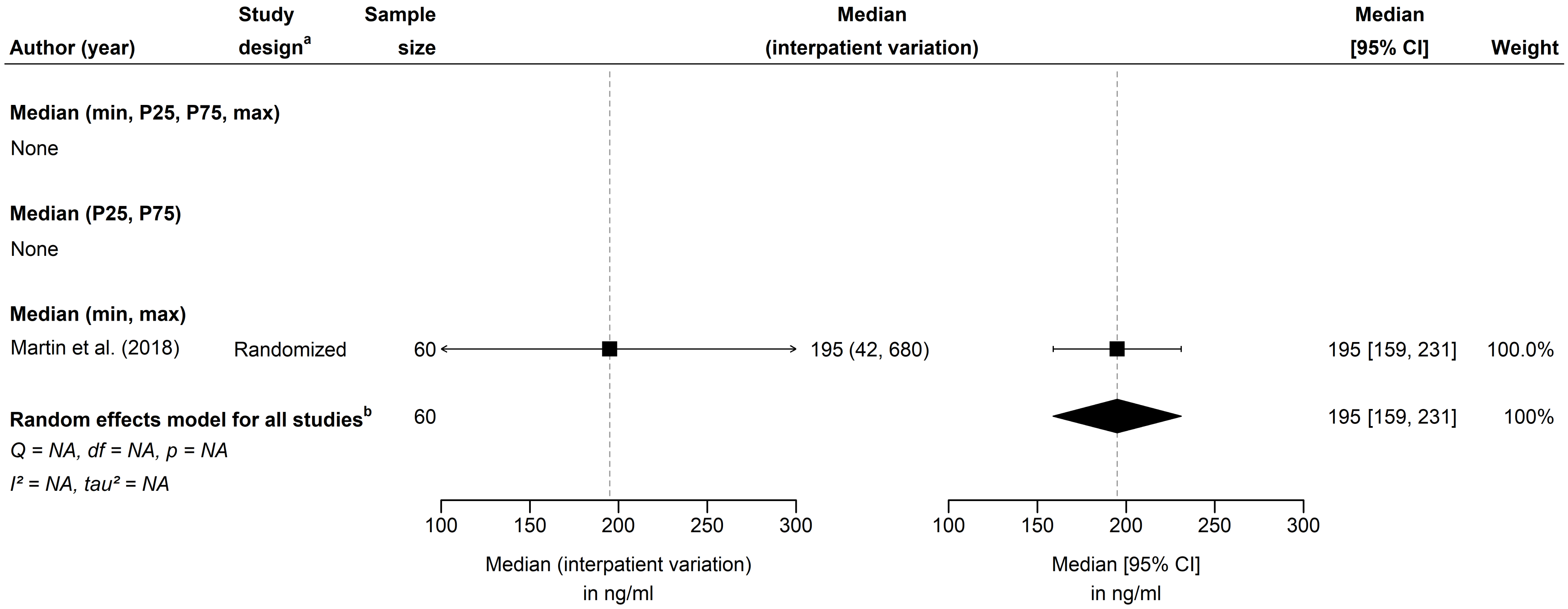

C. Dabigatran 75 mg twice daily

[a] All analyses of interest were cross-sectional; [b] Random effects model using the quantile-estimation method;[54,57-59] [c] Simulated values were used because only the mean and standard deviation were available; [d] Simulated values were used because available parameters could not readily be included in the QE-method; [e] Percentiles were calculated directly from the original dataset if they were published by the authors of the current review.[4,12,37]

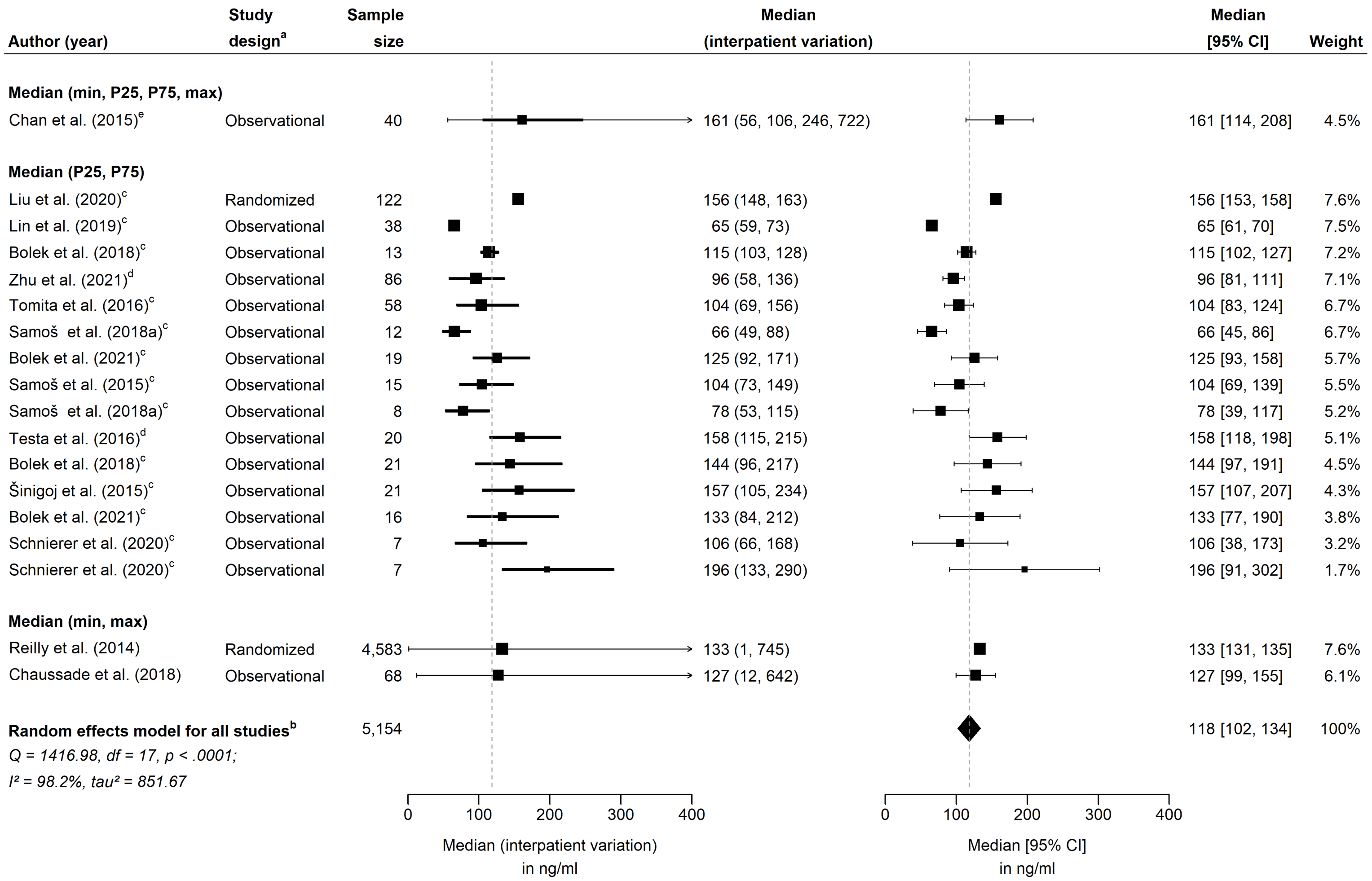

D. Dabigatran 110 mg twice daily

[a] All analyses of interest were cross-sectional; [b] Random effects model using the quantile-estimation method;[54,57-59] [c] Simulated values were used because only the mean and standard deviation were available; [d] Simulated values were used because available parameters could not readily be included in the QE-method; [e] Percentiles were calculated directly from the original dataset if they were published by the authors of the current review.[4,12,37]

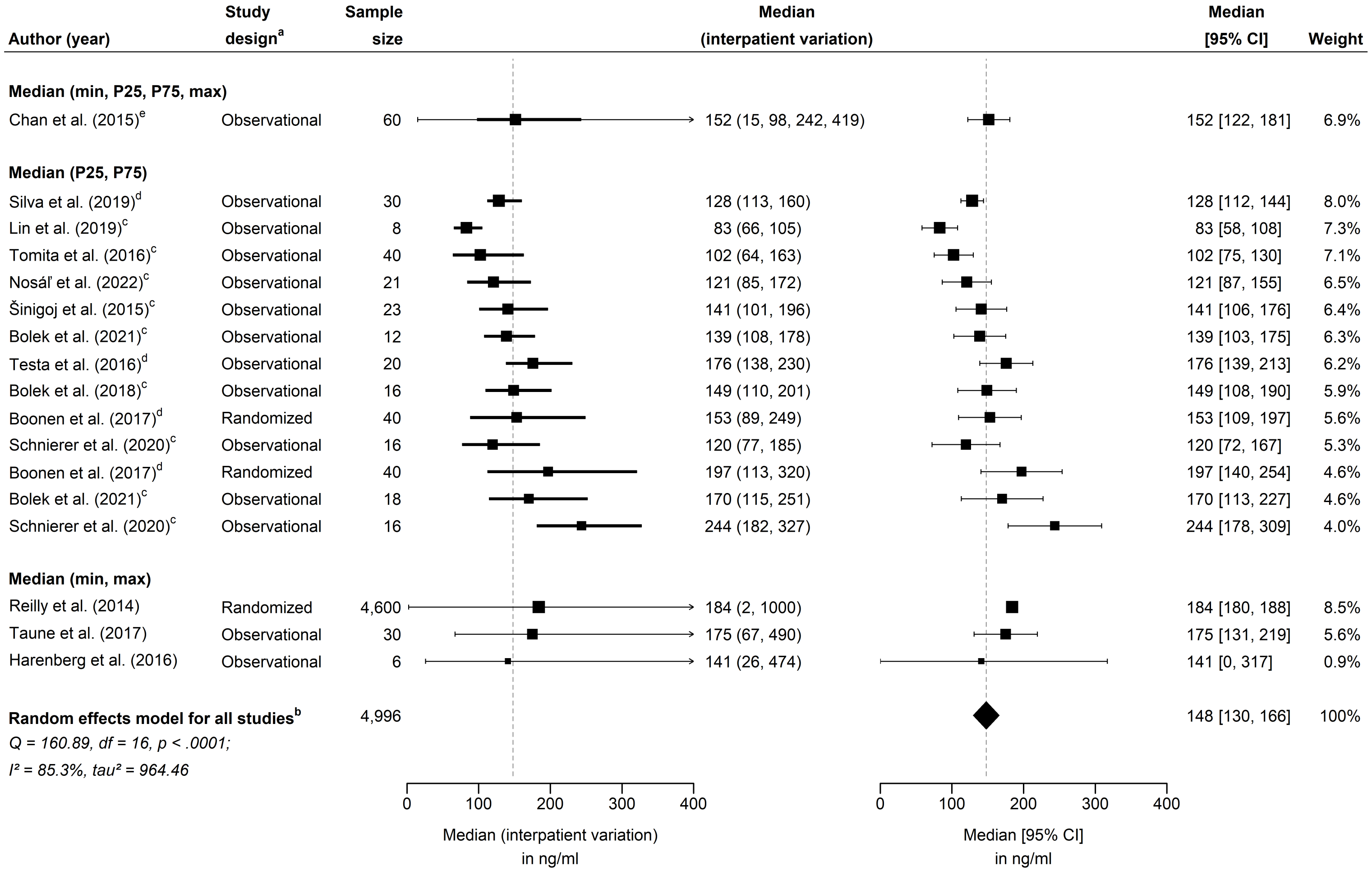

E. Dabigatran 150 mg twice daily

[a] All analyses of interest were cross-sectional; [b] Random effects model using the quantile-estimation method;[54,57-59] [c] Simulated values were used because only the mean and standard deviation were available; [d] Simulated values were used because available parameters could not readily be included in the QE-method; [e] Percentiles were calculated directly from the original dataset if they were published by the authors of the current review.[4,12,37]

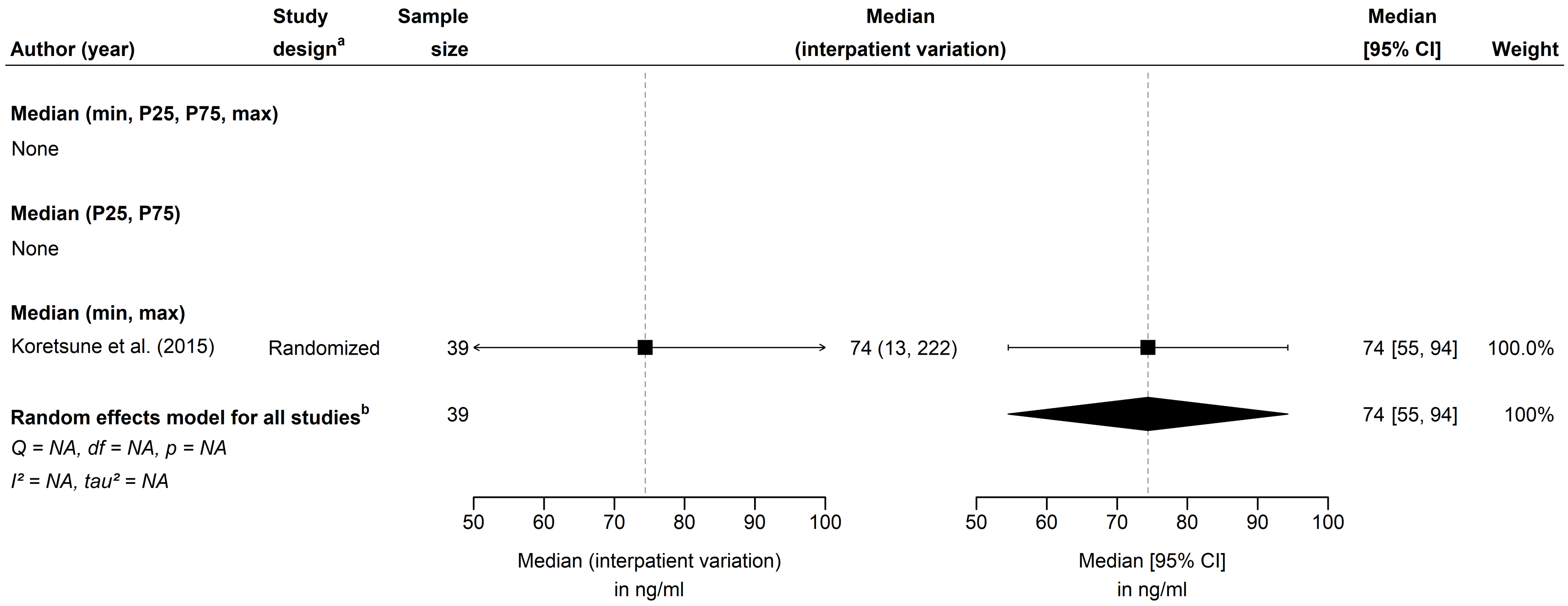

F. Edoxaban 15 mg once daily

[a] All analyses of interest were cross-sectional; [b] Random effects model using the quantile-estimation method;[54,57-59] [c] Simulated values were used because only the mean and standard deviation were available; [d] Simulated values were used because available parameters could not readily be included in the QE-method; [e] Percentiles were calculated directly from the original dataset if they were published by the authors of the current review.[4,12,37]

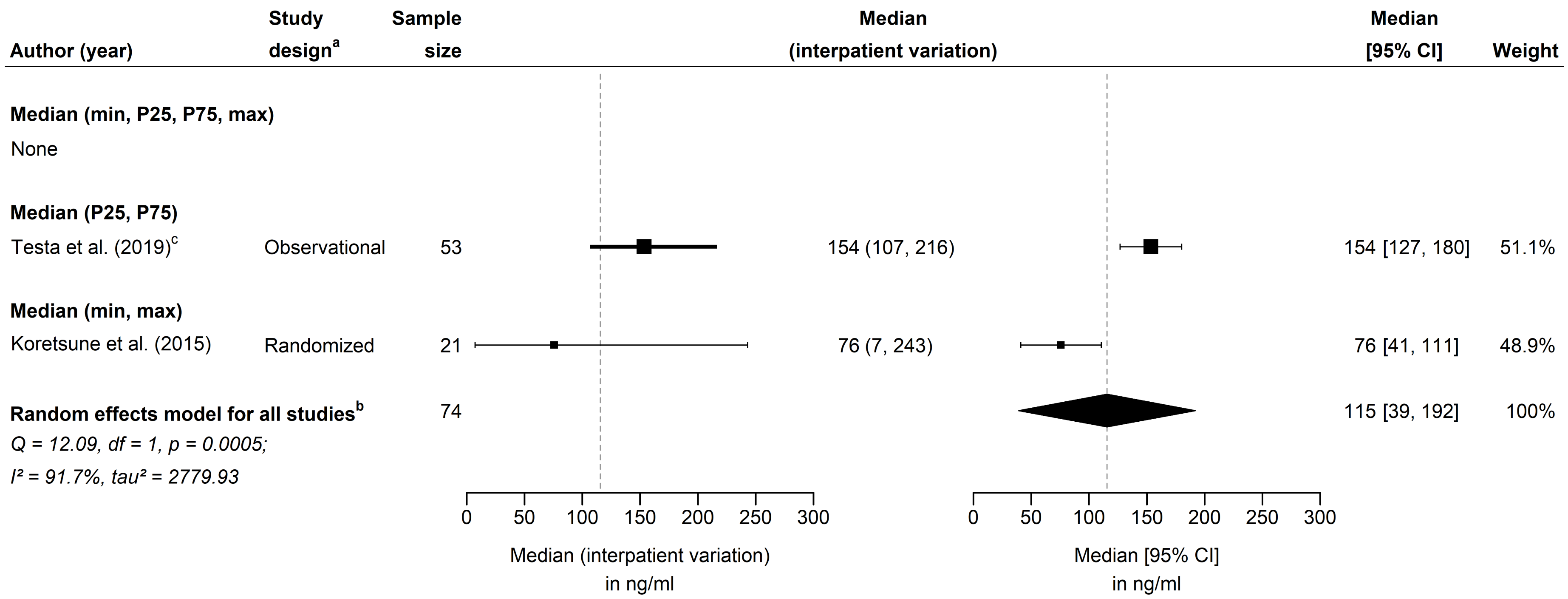

<u>G. Edoxaban 30 mg once daily</u>

[a] All analyses of interest were cross-sectional; [b] Random effects model using the quantile-estimation method;[54,57-59] [c] Simulated values were used because only the mean and standard deviation were available; [d] Simulated values were used because available parameters could not readily be included in the QE-method; [e] Percentiles were calculated directly from the original dataset if they were published by the authors of the current review.[4,12,37]

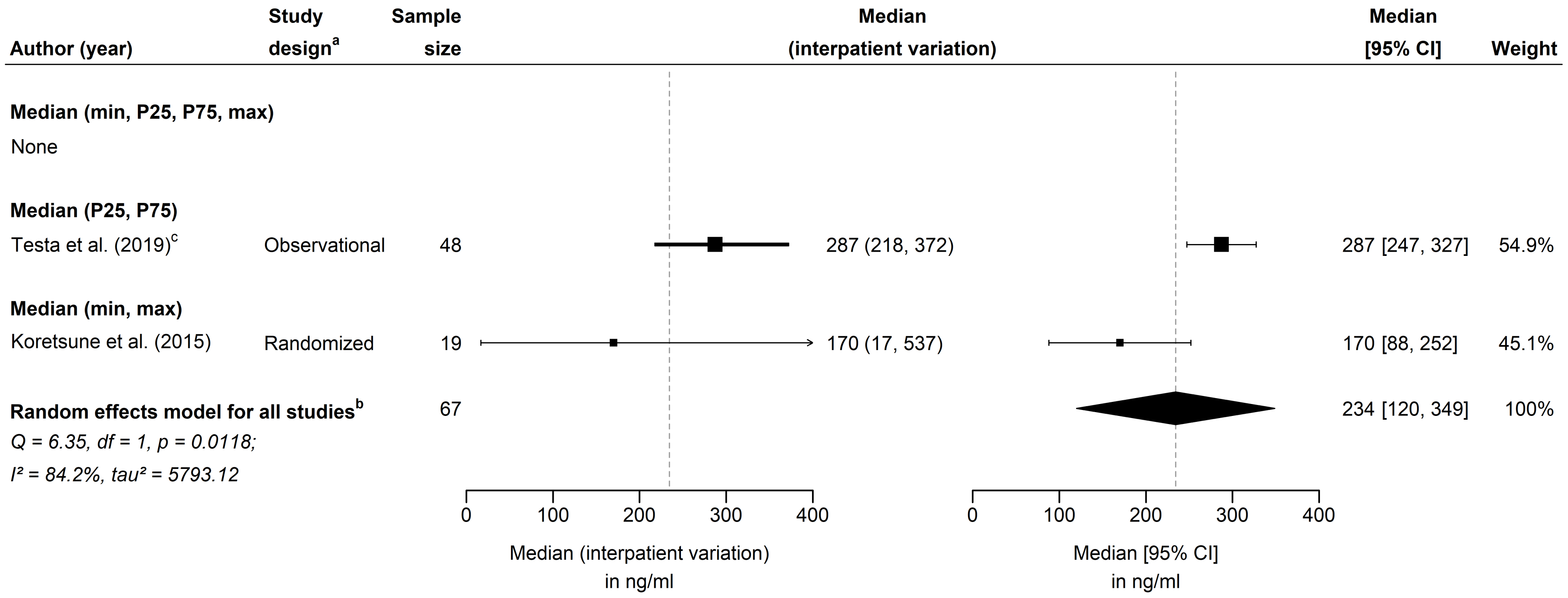

H. Edoxaban 60 mg once daily

[a] All analyses of interest were cross-sectional; [b] Random effects model using the quantile-estimation method;[54,57-59] [c] Simulated values were used because only the mean and standard deviation were available; [d] Simulated values were used because available parameters could not readily be included in the QE-method; [e] Percentiles were calculated directly from the original dataset if they were published by the authors of the current review.[4,12,37]

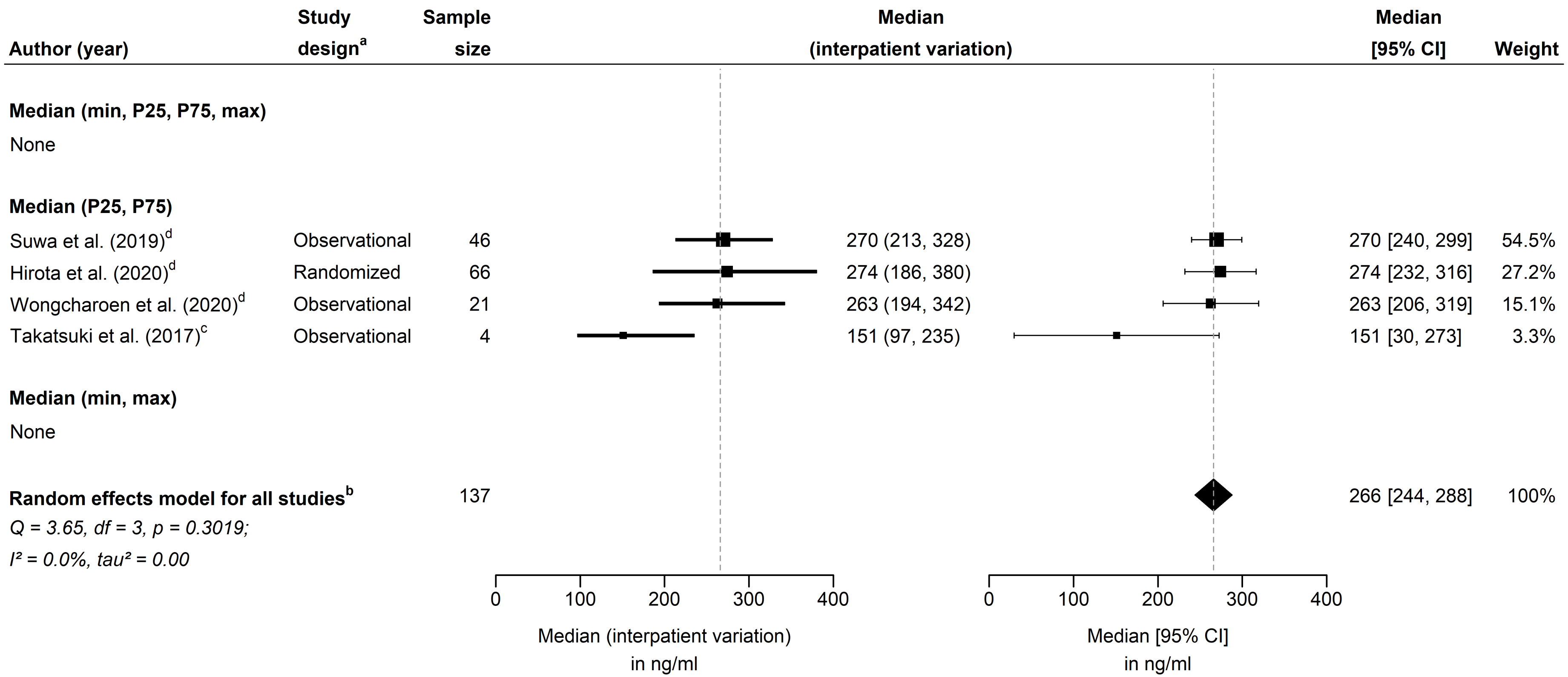

<u>I. Rivaroxaban 10 mg once daily</u>

[a] All analyses of interest were cross-sectional; [b] Random effects model using the quantile-estimation method;[54,57-59] [c] Simulated values were used because only the mean and standard deviation were available; [d] Simulated values were used because available parameters could not readily be included in the QE-method; [e] Percentiles were calculated directly from the original dataset if they were published by the authors of the current review.[4,12,37]

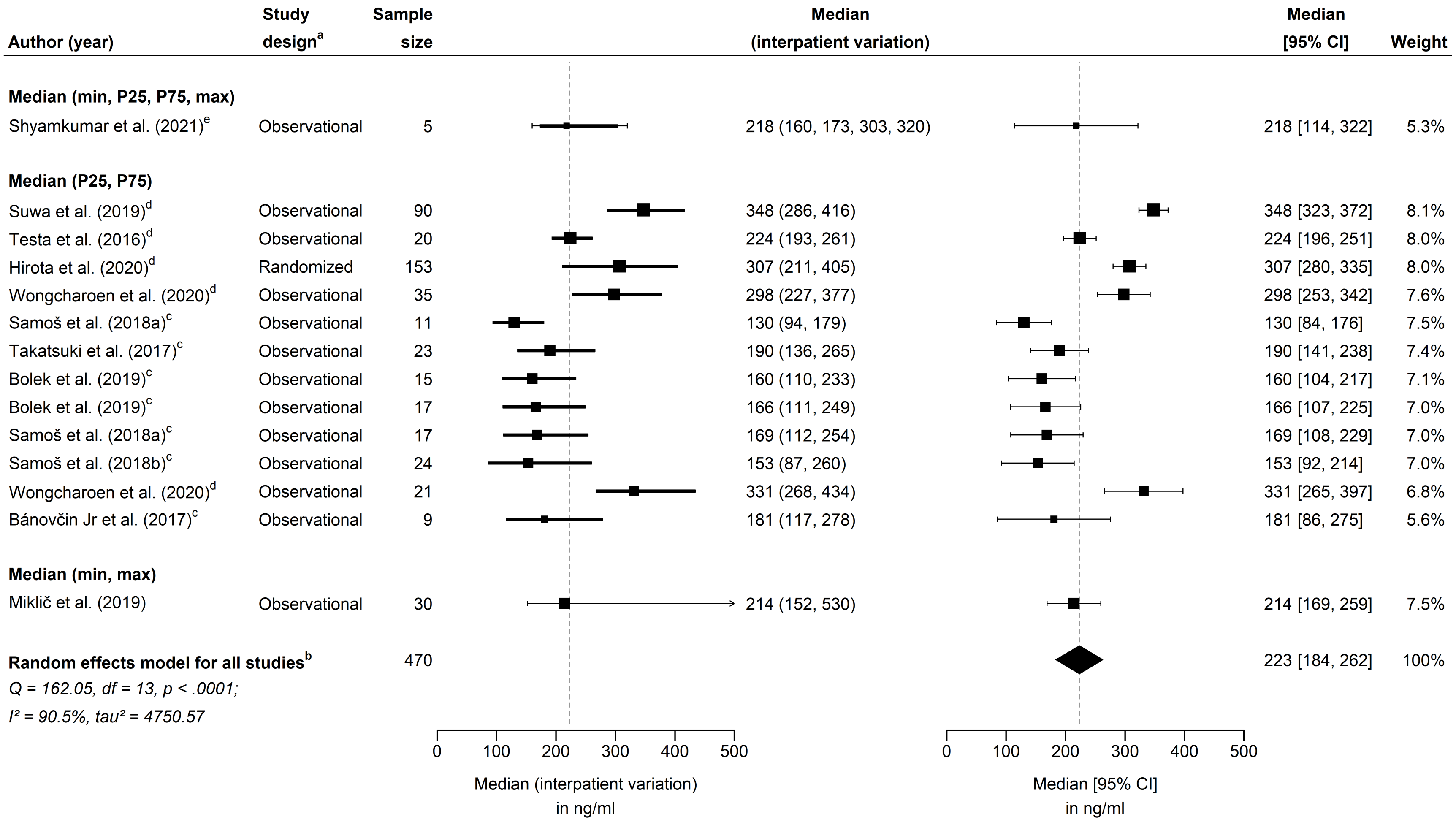

J. Rivaroxaban 15 mg once daily

[a] All analyses of interest were cross-sectional; [b] Random effects model using the quantile-estimation method;[54,57-59] [c] Simulated values were used because only the mean and standard deviation were available; [d] Simulated values were used because available parameters could not readily be included in the QE-method; [e] Percentiles were calculated directly from the original dataset if they were published by the authors of the current review.[4,12,37]

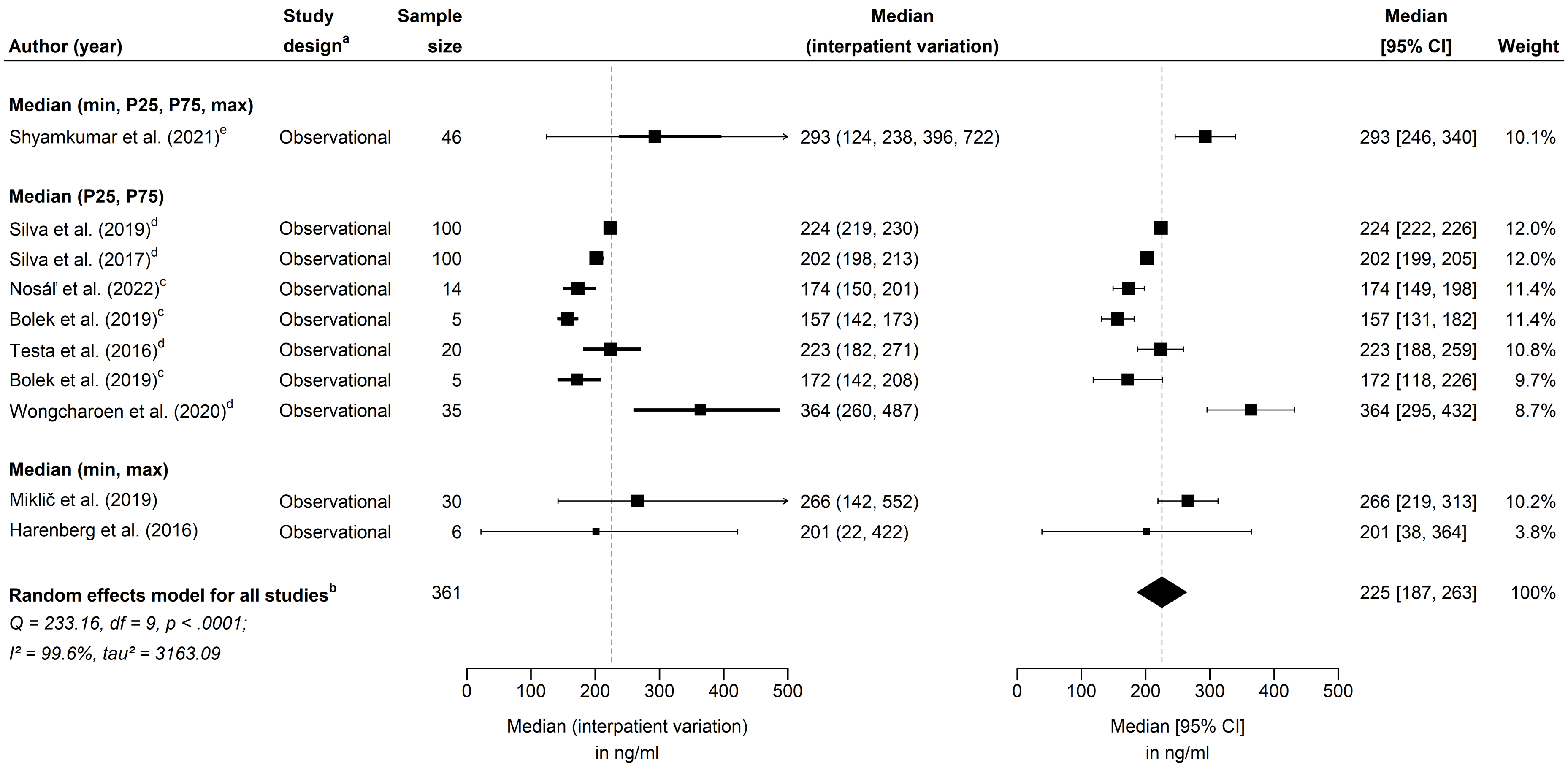

K. Rivaroxaban 20 mg once daily

[a] All analyses of interest were cross-sectional; [b] Random effects model using the quantile-estimation method;[54,57-59] [c] Simulated values were used because only the mean and standard deviation were available; [d] Simulated values were used because available parameters could not readily be included in the QE-method; [e] Percentiles were calculated directly from the original dataset if they were published by the authors of the current review.[4,12,37]

Figure S9. Estimating the pooled 10th percentile of peak levels of each direct oral anticoagulant stratified by administered dose and using the modified QE-method

The forest plots below illustrate the results of our analyses to estimate the 10th percentile of peak levels of each DOAC type, stratified by dosing regimen. The squares represent the 10th percentile values, the circles the median values, the solid bold lines the 25th to 75th percentile range, and the whiskers either the minimum to maximum value interval (left side of the plot) or the 95% of the confidence interval of the 10th percentile values (right side of the plot).

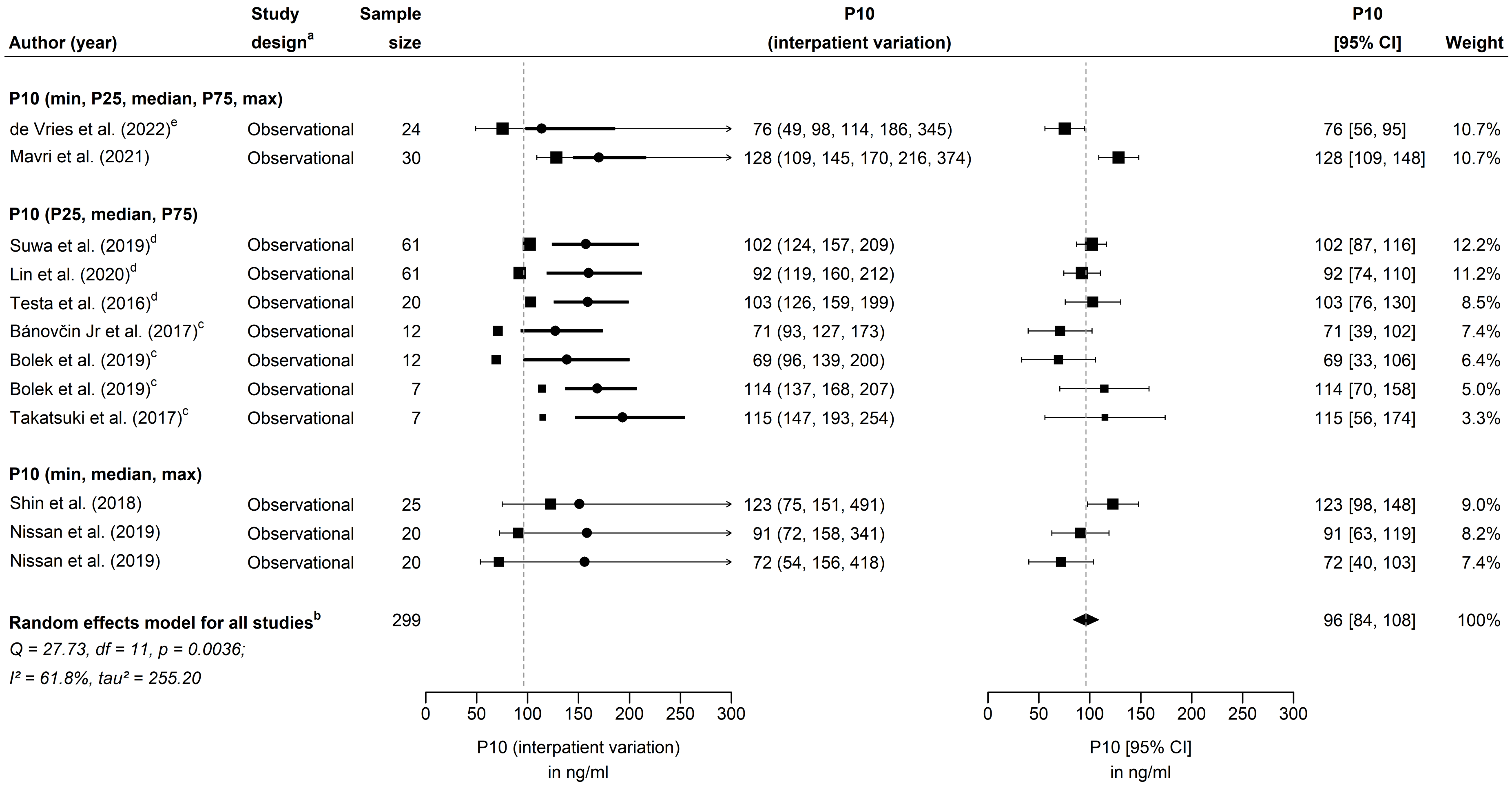

<u>A. Apixaban 2.5 mg twice daily</u>

[a] All analyses of interest were cross-sectional; [b] Random effects model using the quantile-estimation method;[54,57-59] [c] Simulated values were used because only the mean and standard deviation were available; [d] Simulated values were used because available parameters could not readily be included in the QE-method; [e] Percentiles were calculated directly from the original dataset if they were published by the authors of the current review.[4,12,37]

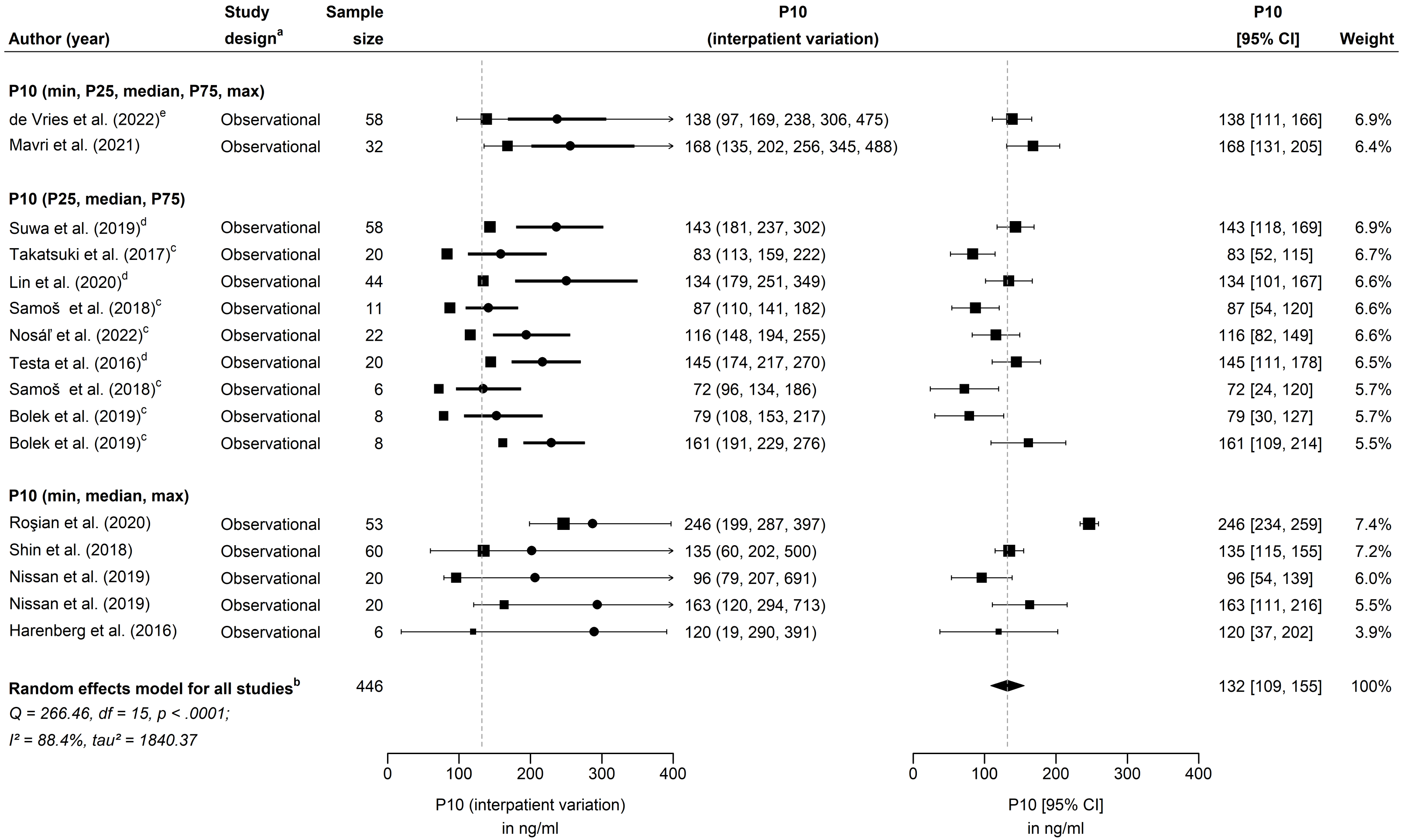

B. Apixaban 5 mg twice daily

[a] All analyses of interest were cross-sectional; [b] Random effects model using the quantile-estimation method;[54,57-59] [c] Simulated values were used because only the mean and standard deviation were available; [d] Simulated values were used because available parameters could not readily be included in the QE-method; [e] Percentiles were calculated directly from the original dataset if they were published by the authors of the current review.[4,12,37]

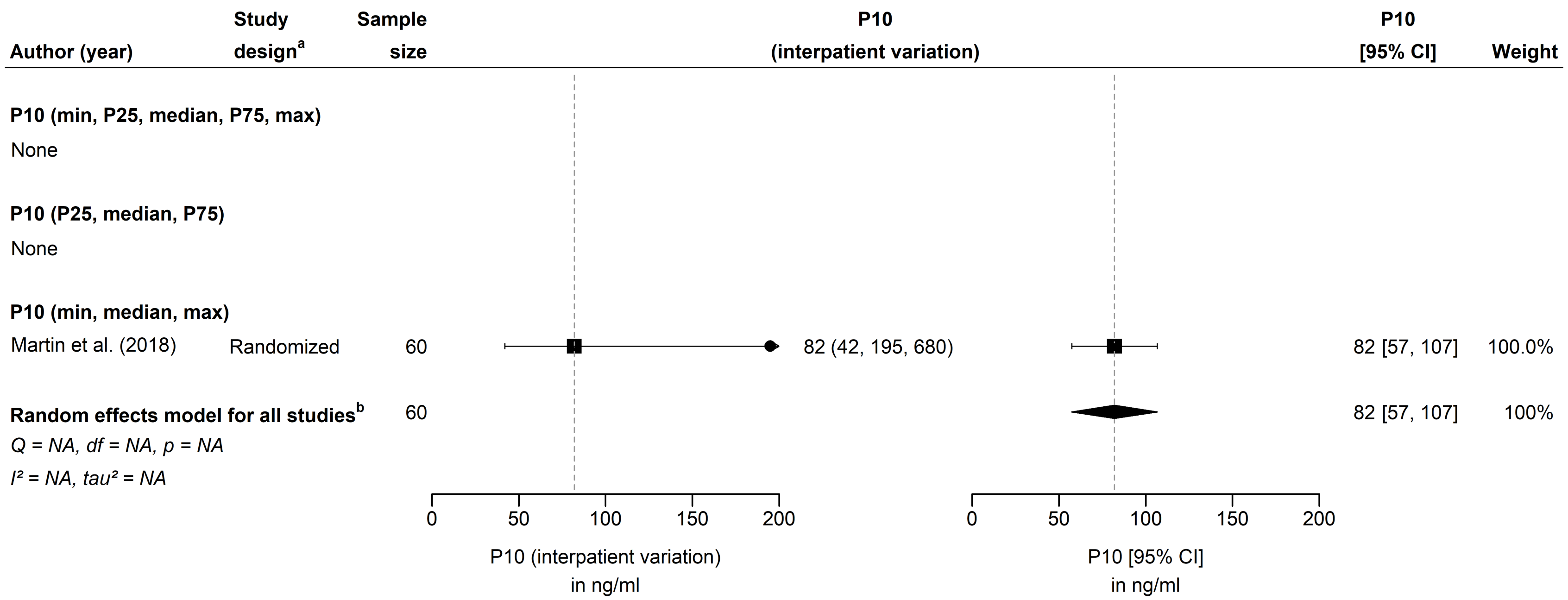

C. Dabigatran 75 mg twice daily

[a] All analyses of interest were cross-sectional; [b] Random effects model using the quantile-estimation method;[54,57-59] [c] Simulated values were used because only the mean and standard deviation were available; [d] Simulated values were used because available parameters could not readily be included in the QE-method; [e] Percentiles were calculated directly from the original dataset if they were published by the authors of the current review.[4,12,37]

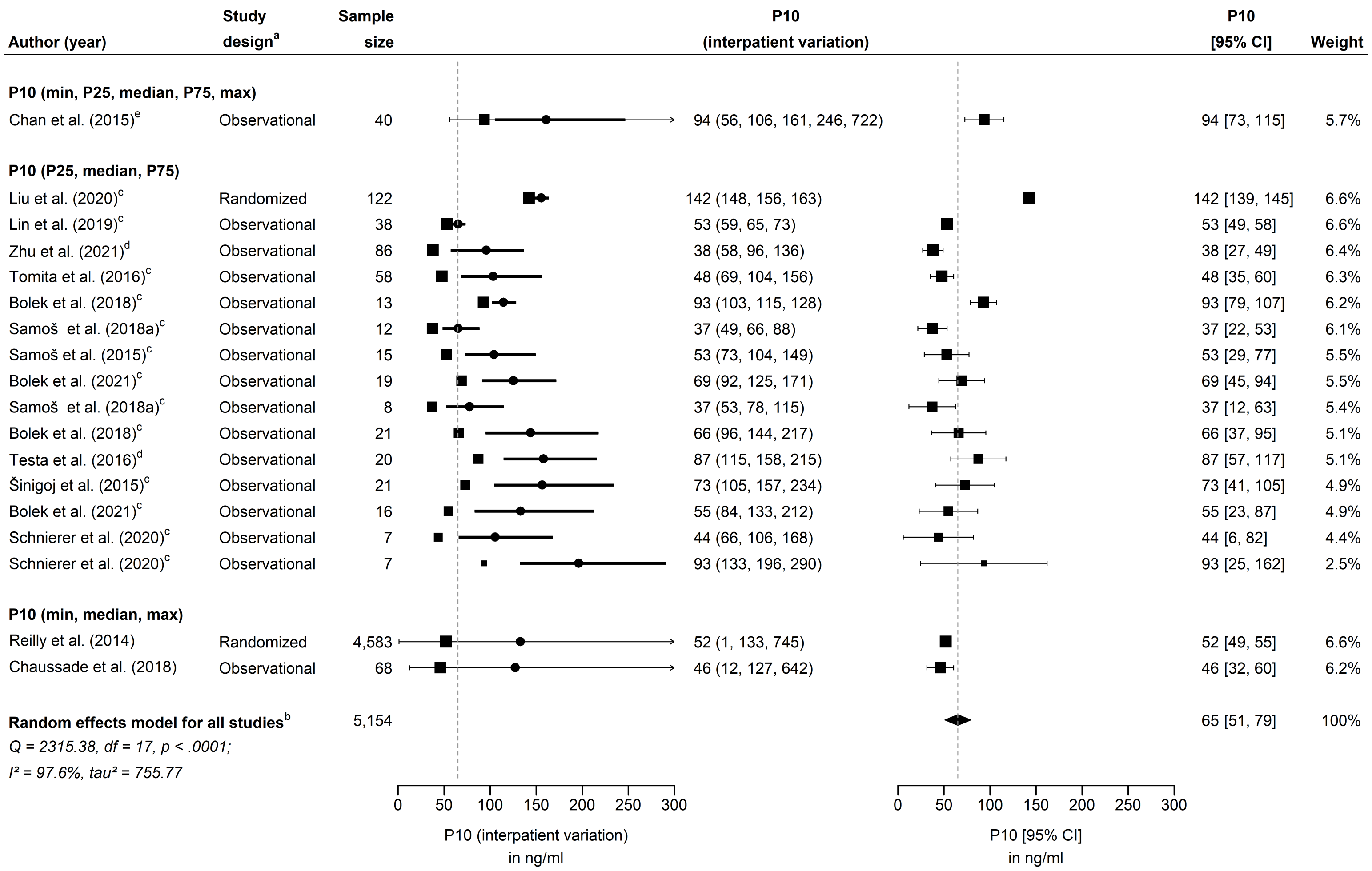

D. Dabigatran 110 mg twice daily

[a] All analyses of interest were cross-sectional; [b] Random effects model using the quantile-estimation method;[54,57-59] [c] Simulated values were used because only the mean and standard deviation were available; [d] Simulated values were used because available parameters could not readily be included in the QE-method; [e] Percentiles were calculated directly from the original dataset if they were published by the authors of the current review.[4,12,37]

Author (year)	Study design[a]	Sample size	P10 (interpatient variation)	P10 [95% CI]	Weight
**P10 (min, P25, median, P75, max)**					
Chan et al. (2015)[e]	Observational	60	48 (15, 98, 152, 242, 419)	48 [22, 74]	6.1%
**P10 (P25, median, P75)**					
Silva et al. (2019)[d]	Observational	30	101 (113, 128, 160)	101 [88, 113]	7.7%
Tomita et al. (2016)[c]	Observational	40	43 (64, 102, 163)	43 [27, 58]	7.4%
Lin et al. (2019)[c]	Observational	8	54 (66, 83, 105)	54 [32, 75]	6.6%
Nosáľ et al. (2022)[c]	Observational	21	61 (85, 121, 172)	61 [38, 85]	6.4%
Šinigoj et al. (2015)[c]	Observational	23	75 (101, 141, 196)	75 [49, 101]	6.1%
Boonen et al. (2017)[d]	Randomized	40	55 (89, 153, 249)	55 [29, 81]	6.1%
Testa et al. (2016)[d]	Observational	20	118 (138, 176, 230)	118 [92, 144]	6.1%
Schnierer et al. (2020)[c]	Observational	16	52 (77, 120, 185)	52 [24, 81]	5.8%
Bolek et al. (2021)[c]	Observational	12	87 (108, 139, 178)	87 [56, 117]	5.5%
Bolek et al. (2018)[c]	Observational	16	84 (110, 149, 201)	84 [53, 115]	5.4%
Boonen et al. (2017)[d]	Randomized	40	66 (113, 197, 320)	66 [32, 100]	5.1%
Bolek et al. (2021)[c]	Observational	18	81 (115, 170, 251)	81 [44, 118]	4.8%
Schnierer et al. (2020)[c]	Observational	16	139 (182, 244, 327)	139 [89, 190]	3.5%
**P10 (min, median, max)**					
Reilly et al. (2014)	Randomized	4,600	74 (2, 184, 1000)	74 [71, 78]	8.2%
Taune et al. (2017)	Observational	30	116 (67, 175, 490)	116 [93, 139]	6.5%
Harenberg et al. (2016)	Observational	6	70 (26, 141, 474)	70 [7, 132]	2.7%
**Random effects model for all studies[b]**		4,996		76 [64, 89]	100%

Q = 78.16, df = 16, p < .0001;
I² = 85.1%, tau² = 494.43

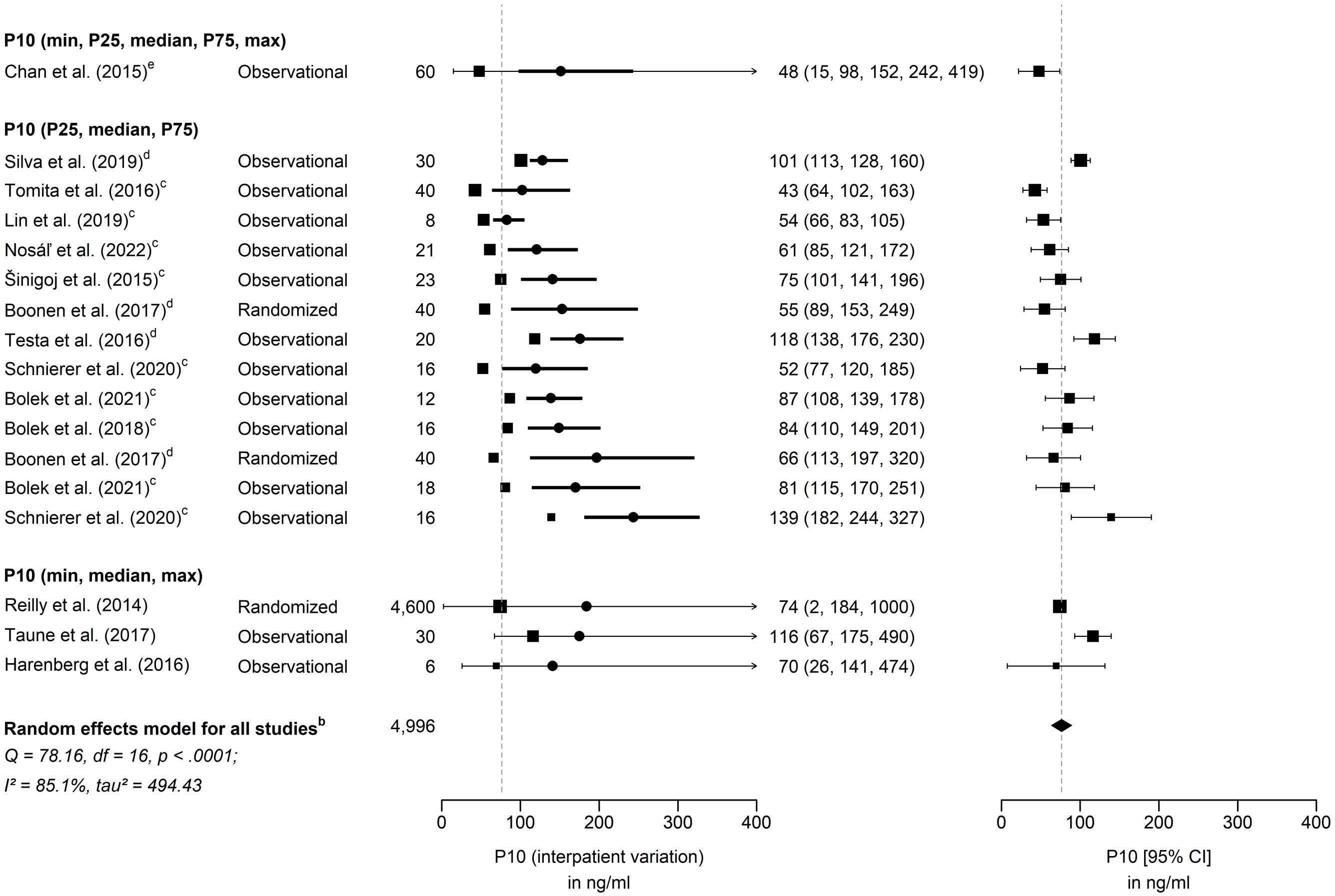

E. Dabigatran 150 mg twice daily

[a] All analyses of interest were cross-sectional; [b] Random effects model using the quantile-estimation method;[54,57-59] [c] Simulated values were used because only the mean and standard deviation were available; [d] Simulated values were used because available parameters could not readily be included in the QE-method; [e] Percentiles were calculated directly from the original dataset if they were published by the authors of the current review.[4,12,37]

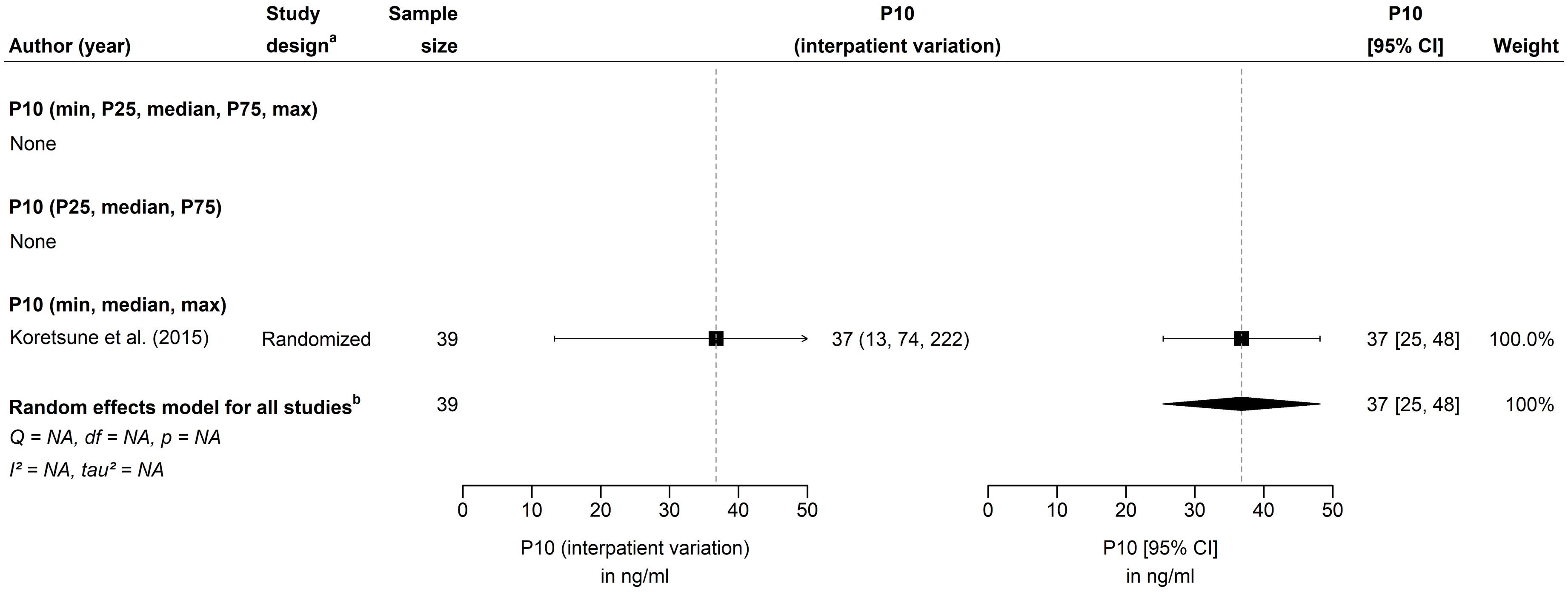

F. Edoxaban 15 mg once daily

[a] All analyses of interest were cross-sectional; [b] Random effects model using the quantile-estimation method;[54,57-59] [c] Simulated values were used because only the mean and standard deviation were available; [d] Simulated values were used because available parameters could not readily be included in the QE-method; [e] Percentiles were calculated directly from the original dataset if they were published by the authors of the current review.[4,12,37]

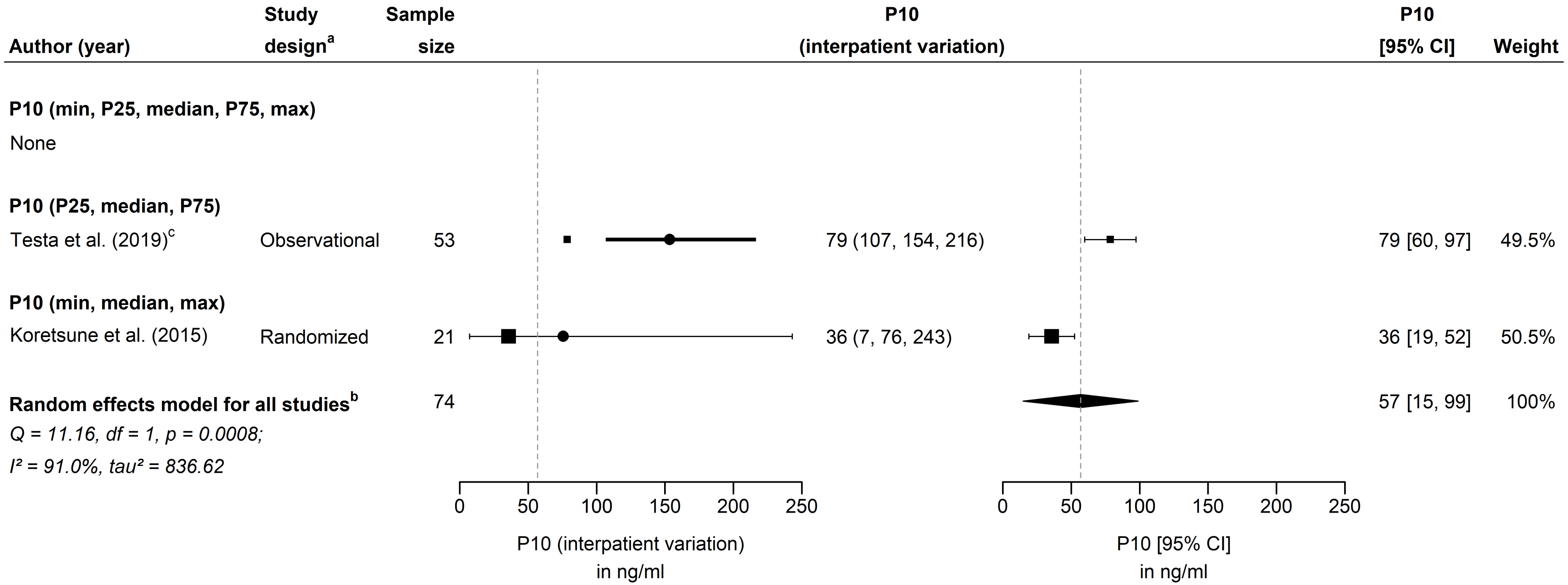

G. Edoxaban 30 mg once daily

[a] All analyses of interest were cross-sectional; [b] Random effects model using the quantile-estimation method;[54,57-59] [c] Simulated values were used because only the mean and standard deviation were available; [d] Simulated values were used because available parameters could not readily be included in the QE-method; [e] Percentiles were calculated directly from the original dataset if they were published by the authors of the current review.[4,12,37]

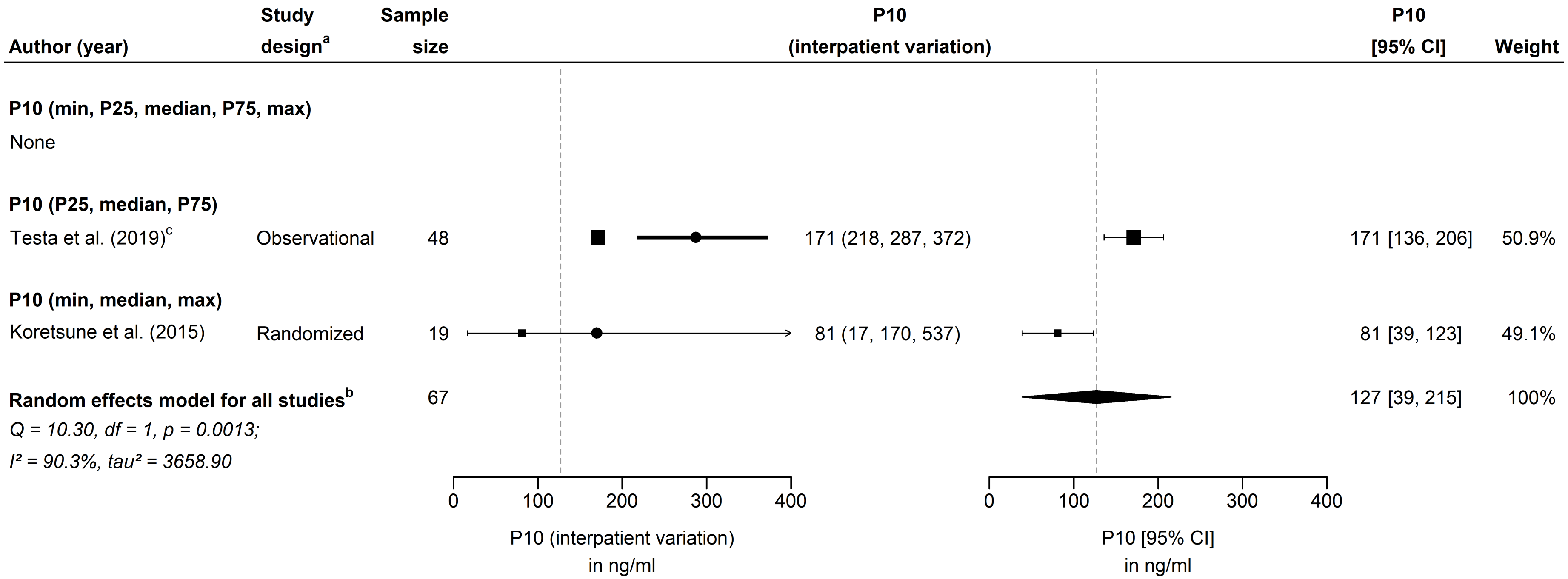

H. Edoxaban 60 mg once daily

[a] All analyses of interest were cross-sectional; [b] Random effects model using the quantile-estimation method;[54,57-59] [c] Simulated values were used because only the mean and standard deviation were available; [d] Simulated values were used because available parameters could not readily be included in the QE-method; [e] Percentiles were calculated directly from the original dataset if they were published by the authors of the current review.[4,12,37]

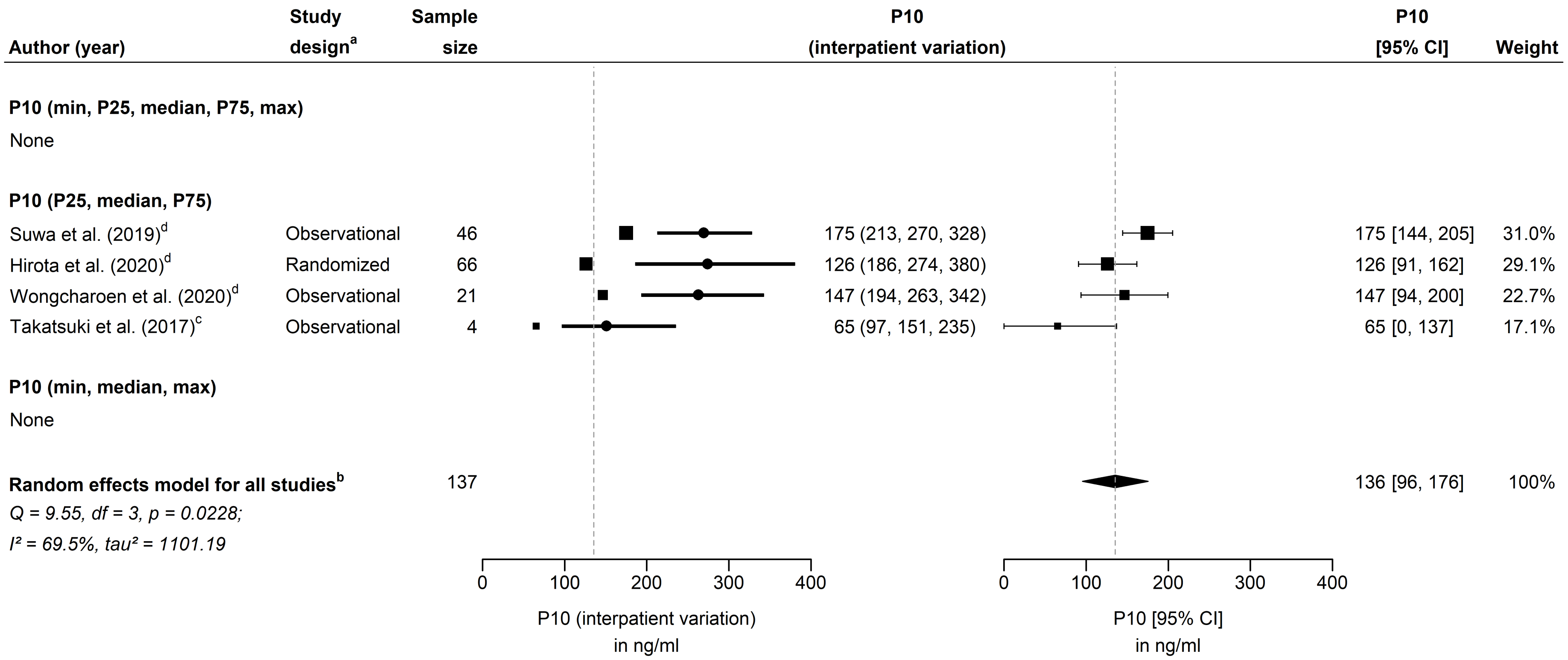

I. Rivaroxaban 10 mg once daily

[a] All analyses of interest were cross-sectional; [b] Random effects model using the quantile-estimation method;[54,57-59] [c] Simulated values were used because only the mean and standard deviation were available; [d] Simulated values were used because available parameters could not readily be included in the QE-method; [e] Percentiles were calculated directly from the original dataset if they were published by the authors of the current review.[4,12,37]

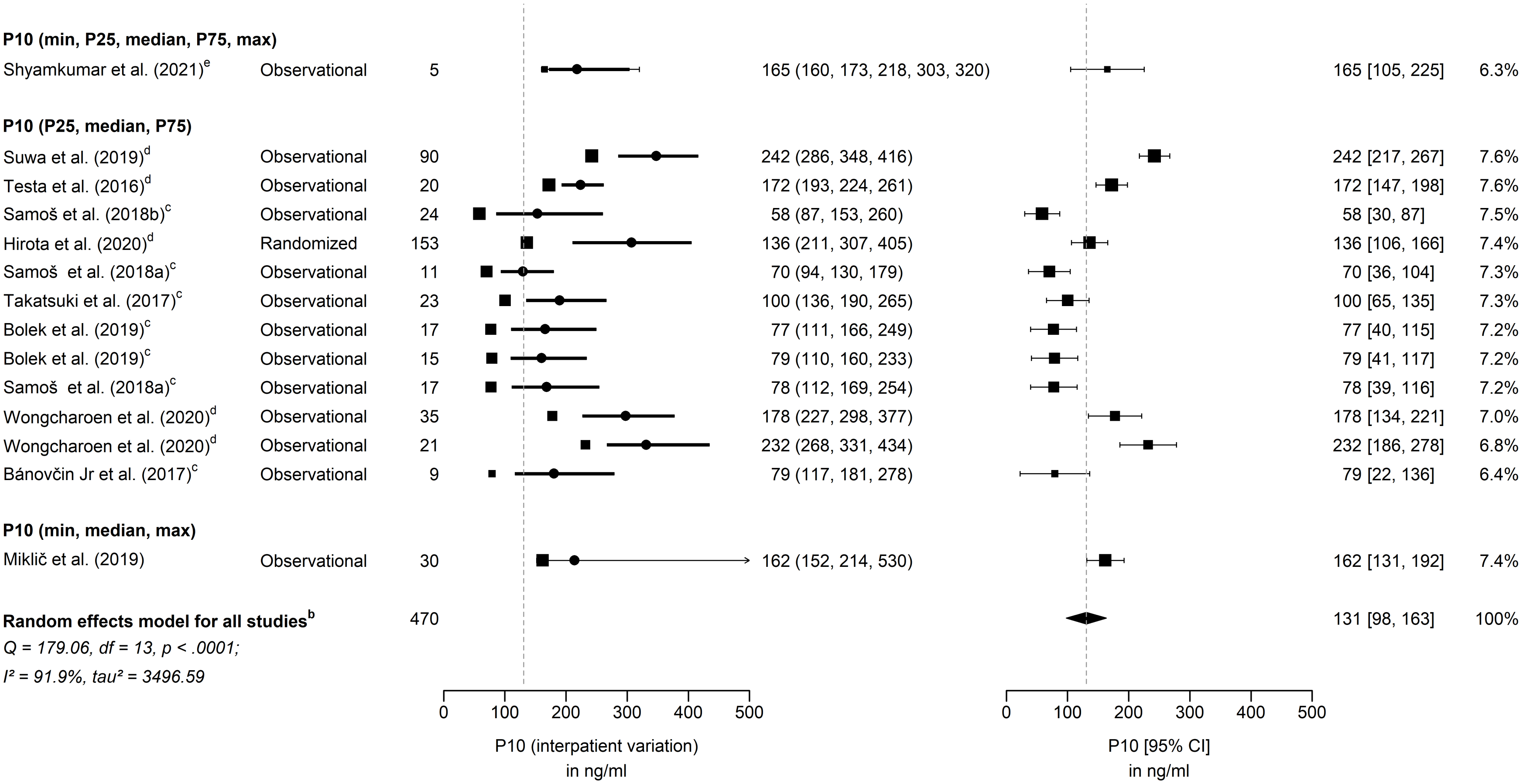

Author (year)	Study design[a]	Sample size	P10 (interpatient variation)	P10 [95% CI]	Weight
**P10 (min, P25, median, P75, max)**					
Shyamkumar et al. (2021)[e]	Observational	5	165 (160, 173, 218, 303, 320)	165 [105, 225]	6.3%
**P10 (P25, median, P75)**					
Suwa et al. (2019)[d]	Observational	90	242 (286, 348, 416)	242 [217, 267]	7.6%
Testa et al. (2016)[d]	Observational	20	172 (193, 224, 261)	172 [147, 198]	7.6%
Samoš et al. (2018b)[c]	Observational	24	58 (87, 153, 260)	58 [30, 87]	7.5%
Hirota et al. (2020)[d]	Randomized	153	136 (211, 307, 405)	136 [106, 166]	7.4%
Samoš et al. (2018a)[c]	Observational	11	70 (94, 130, 179)	70 [36, 104]	7.3%
Takatsuki et al. (2017)[c]	Observational	23	100 (136, 190, 265)	100 [65, 135]	7.3%
Bolek et al. (2019)[c]	Observational	17	77 (111, 166, 249)	77 [40, 115]	7.2%
Bolek et al. (2019)[c]	Observational	15	79 (110, 160, 233)	79 [41, 117]	7.2%
Samoš et al. (2018a)[c]	Observational	17	78 (112, 169, 254)	78 [39, 116]	7.2%
Wongcharoen et al. (2020)[d]	Observational	35	178 (227, 298, 377)	178 [134, 221]	7.0%
Wongcharoen et al. (2020)[d]	Observational	21	232 (268, 331, 434)	232 [186, 278]	6.8%
Bánovčin Jr et al. (2017)[c]	Observational	9	79 (117, 181, 278)	79 [22, 136]	6.4%
**P10 (min, median, max)**					
Miklič et al. (2019)	Observational	30	162 (152, 214, 530)	162 [131, 192]	7.4%
**Random effects model for all studies[b]**		470		131 [98, 163]	100%

Q = 179.06, df = 13, p < .0001;
I² = 91.9%, tau² = 3496.59

J. Rivaroxaban 15 mg once daily

[a] All analyses of interest were cross-sectional; [b] Random effects model using the quantile-estimation method;[54,57-59] [c] Simulated values were used because only the mean and standard deviation were available; [d] Simulated values were used because available parameters could not readily be included in the QE-method; [e] Percentiles were calculated directly from the original dataset if they were published by the authors of the current review.[4,12,37]

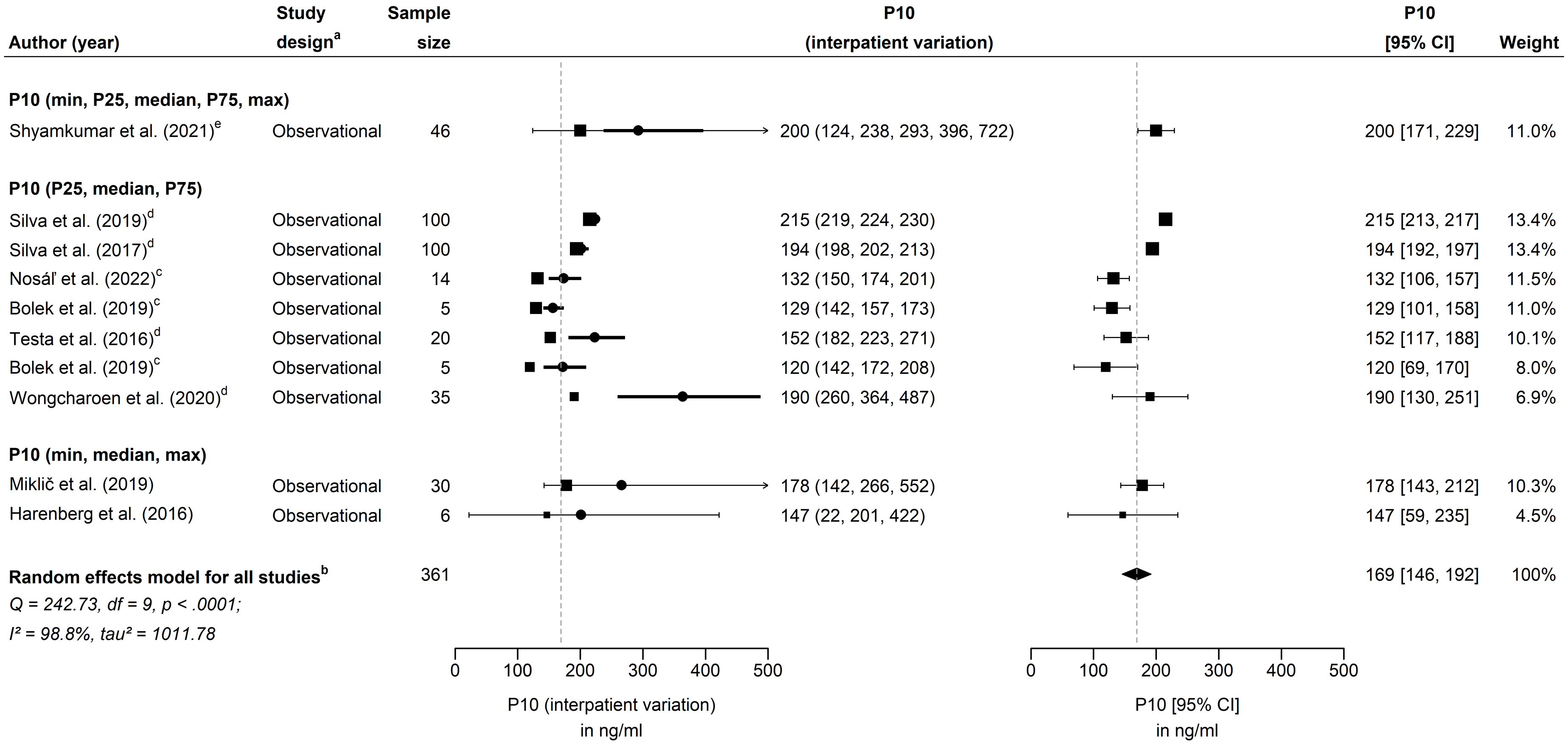

K. Rivaroxaban 20 mg once daily

[a] All analyses of interest were cross-sectional; [b] Random effects model using the quantile-estimation method;[54,57-59] [c] Simulated values were used because only the mean and standard deviation were available; [d] Simulated values were used because available parameters could not readily be included in the QE-method; [e] Percentiles were calculated directly from the original dataset if they were published by the authors of the current review.[4,12,37]

Figure S10. Main analysis: Estimating the pooled 90th percentile of peak levels of each direct oral anticoagulant stratified by administered dose and using the modified QE-method

The forest plots below illustrate the results of our analyses to estimate the 90th percentile of peak levels of each DOAC type, stratified by dosing regimen. The squares represent the 90th percentile values, the circles the median values, the solid bold lines the 25th to 75th percentile range, and the whiskers either the minimum to maximum value interval (left side of the plot) or the 95% of the confidence interval of the 90th percentile values (right side of the plot).

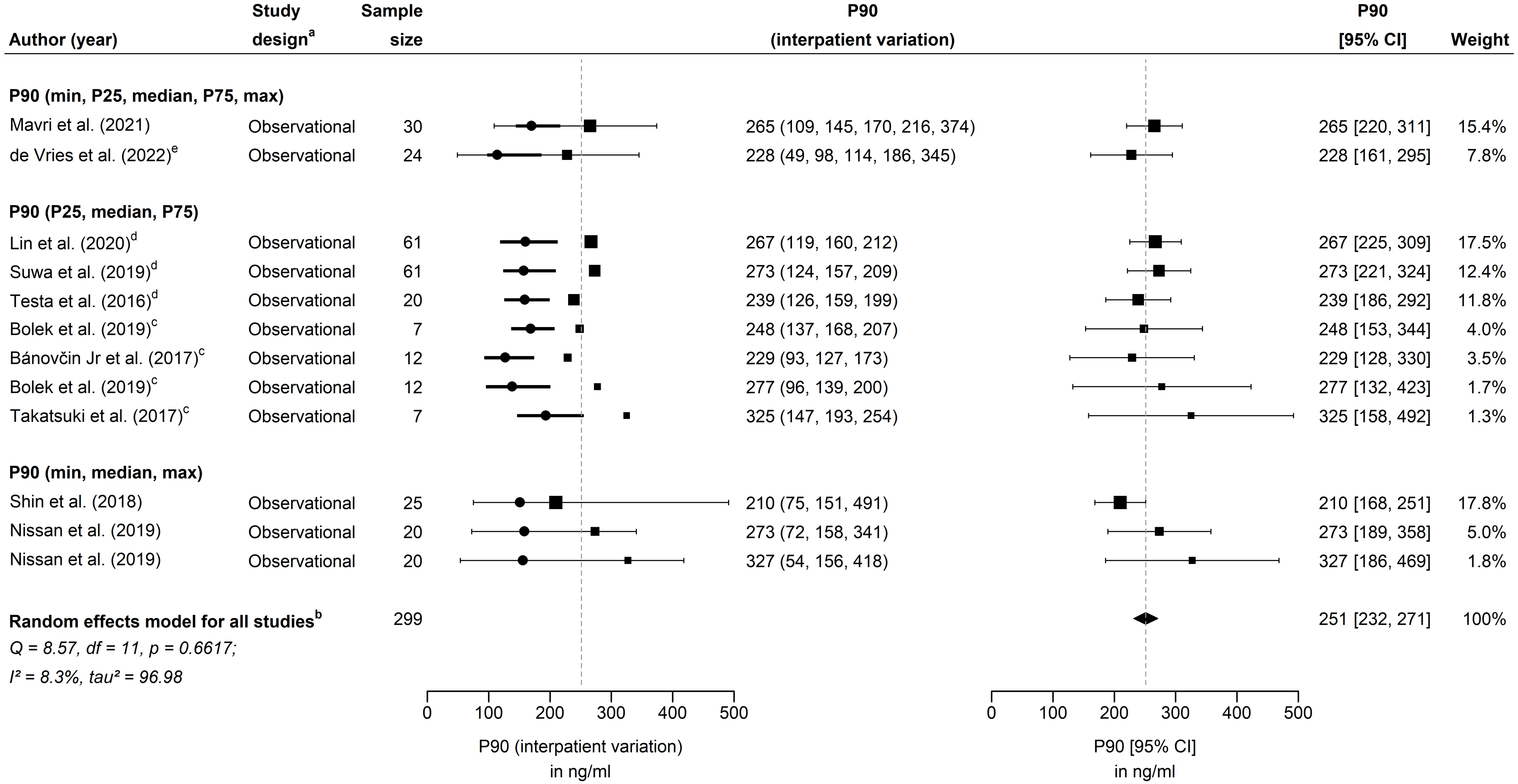

A. Apixaban 2.5 mg twice daily

[a] All analyses of interest were cross-sectional; [b] Random effects model using the quantile-estimation method;[54,57-59] [c] Simulated values were used because only the mean and standard deviation were available; [d] Simulated values were used because available parameters could not readily be included in the QE-method; [e] Percentiles were calculated directly from the original dataset if they were published by the authors of the current review.[4,12,37]

Author (year)	Study design[a]	Sample size	P90 (interpatient variation)	P90 [95% CI]	Weight
**P90 (min, P25, median, P75, max)**					
de Vries et al. (2022)[e]	Observational	58	358 (97, 169, 238, 306, 475)	358 [312, 405]	11.3%
Mavri et al. (2021)	Observational	32	419 (135, 202, 256, 345, 488)	419 [335, 503]	6.7%
**P90 (P25, median, P75)**					
Suwa et al. (2019)[d]	Observational	58	379 (181, 237, 302)	379 [320, 438]	9.5%
Testa et al. (2016)[d]	Observational	20	323 (174, 217, 270)	323 [250, 396]	7.8%
Samoš et al. (2018a)[c]	Observational	11	229 (110, 141, 182)	229 [142, 316]	6.4%
Nosáľ et al. (2022)[c]	Observational	22	326 (148, 194, 255)	326 [232, 420]	5.8%
Bolek et al. (2019)[c]	Observational	8	326 (191, 229, 276)	326 [220, 431]	4.9%
Lin et al. (2020)[d]	Observational	44	462 (179, 251, 349)	462 [353, 571]	4.7%
Takatsuki et al. (2017)[c]	Observational	20	301 (113, 159, 222)	301 [188, 415]	4.5%
Samoš et al. (2018a)[c]	Observational	6	251 (96, 134, 186)	251 [83, 418]	2.4%
Bolek et al. (2019)[c]	Observational	8	297 (108, 153, 217)	297 [115, 479]	2.1%
**P90 (min, median, max)**					
Roşian et al. (2020)	Observational	53	334 (199, 287, 397)	334 [318, 351]	15.4%
Shin et al. (2018)	Observational	60	304 (60, 202, 500)	304 [269, 339]	13.0%
Harenberg et al. (2016)	Observational	6	363 (19, 290, 391)	363 [189, 537]	2.3%
Nissan et al. (2019)	Observational	20	533 (120, 294, 713)	533 [358, 707]	2.2%
Nissan et al. (2019)	Observational	20	517 (79, 207, 691)	517 [256, 777]	1.1%
**Random effects model for all studies[b]**		446		343 [315, 371]	100%

Q = 29.40, df = 15, p = 0.0143;
I² = 55.4%, tau² = 1265.63

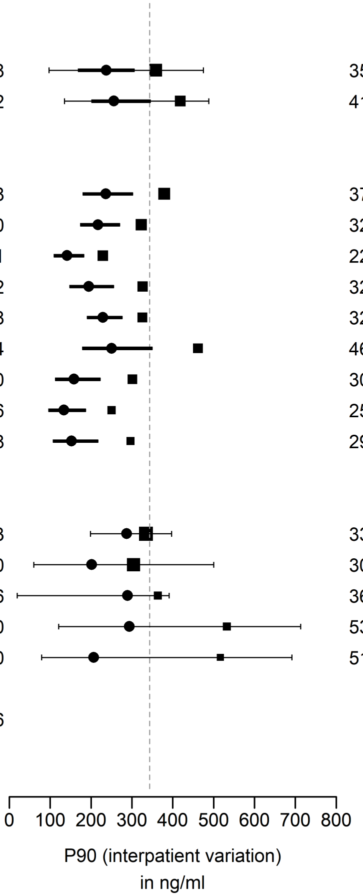

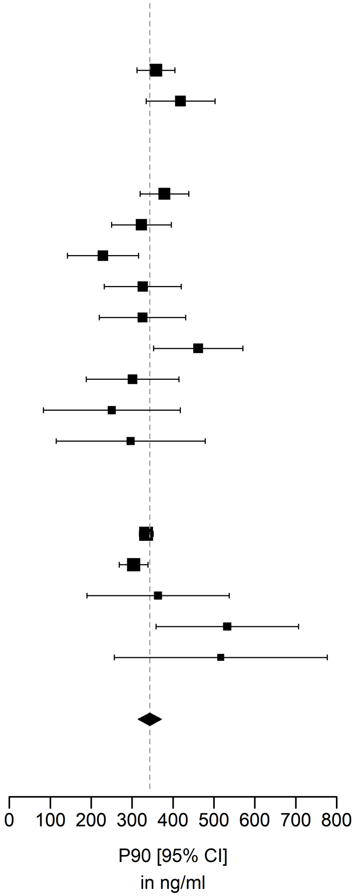

B. Apixaban 5 mg twice daily

[a] All analyses of interest were cross-sectional; [b] Random effects model using the quantile-estimation method;[54,57-59] [c] Simulated values were used because only the mean and standard deviation were available; [d] Simulated values were used because available parameters could not readily be included in the QE-method; [e] Percentiles were calculated directly from the original dataset if they were published by the authors of the current review.[4,12,37]

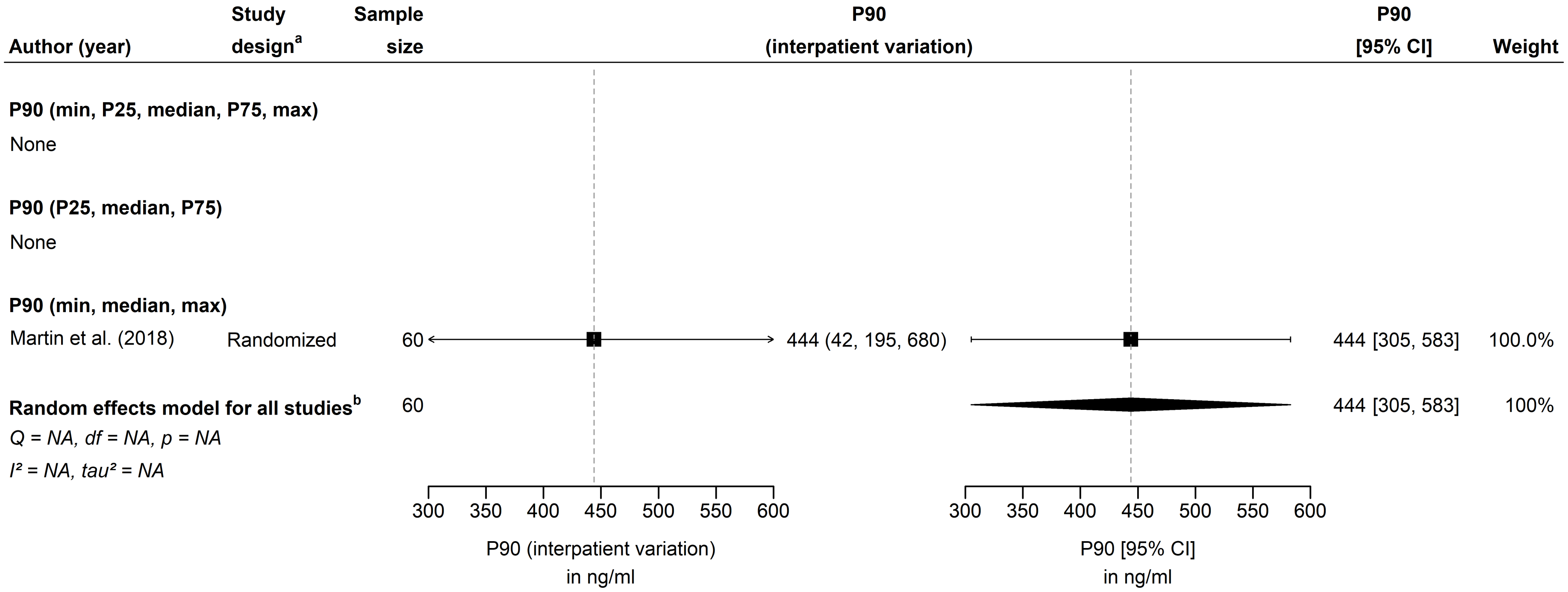

C. Dabigatran 75 mg twice daily

[a] All analyses of interest were cross-sectional; [b] Random effects model using the quantile-estimation method;[54,57-59] [c] Simulated values were used because only the mean and standard deviation were available; [d] Simulated values were used because available parameters could not readily be included in the QE-method; [e] Percentiles were calculated directly from the original dataset if they were published by the authors of the current review.[4,12,37]

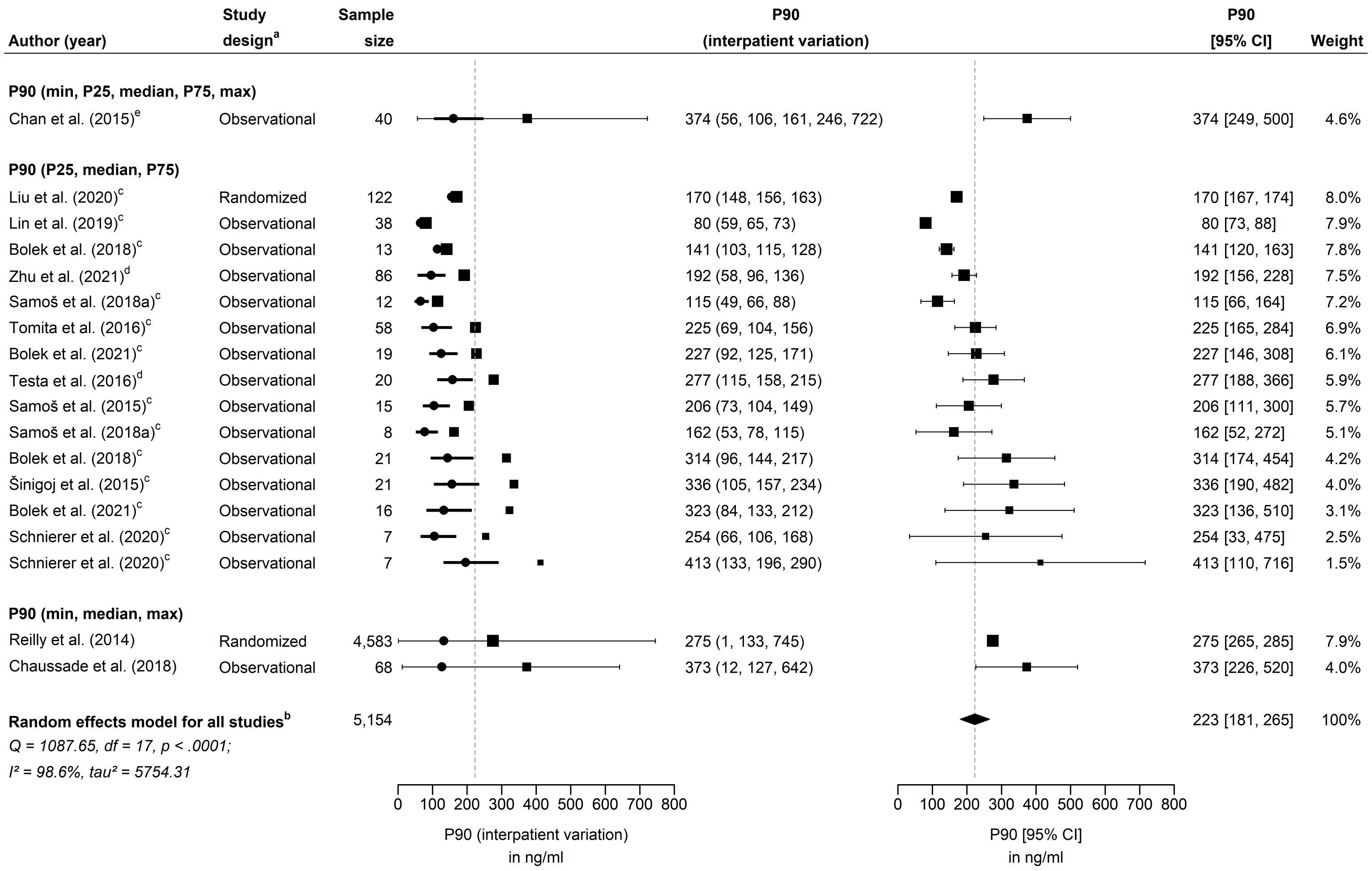

D. Dabigatran 110 mg twice daily

[a] All analyses of interest were cross-sectional; [b] Random effects model using the quantile-estimation method;[54,57-59] [c] Simulated values were used because only the mean and standard deviation were available; [d] Simulated values were used because available parameters could not readily be included in the QE-method; [e] Percentiles were calculated directly from the original dataset if they were published by the authors of the current review.[4,12,37]

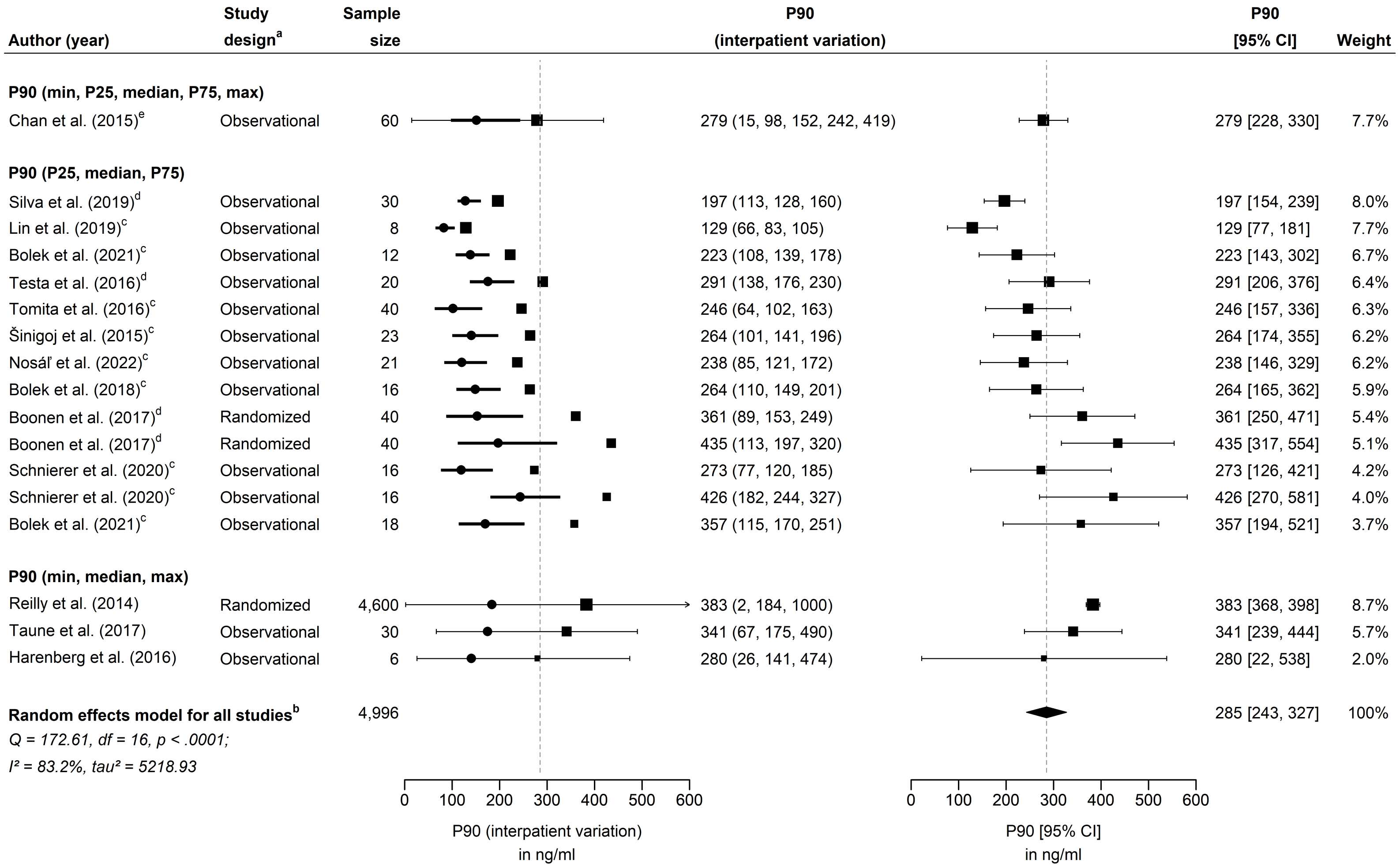

E. Dabigatran 150 mg twice daily

[a] All analyses of interest were cross-sectional; [b] Random effects model using the quantile-estimation method;[54,57-59] [c] Simulated values were used because only the mean and standard deviation were available; [d] Simulated values were used because available parameters could not readily be included in the QE-method; [e] Percentiles were calculated directly from the original dataset if they were published by the authors of the current review.[4,12,37]

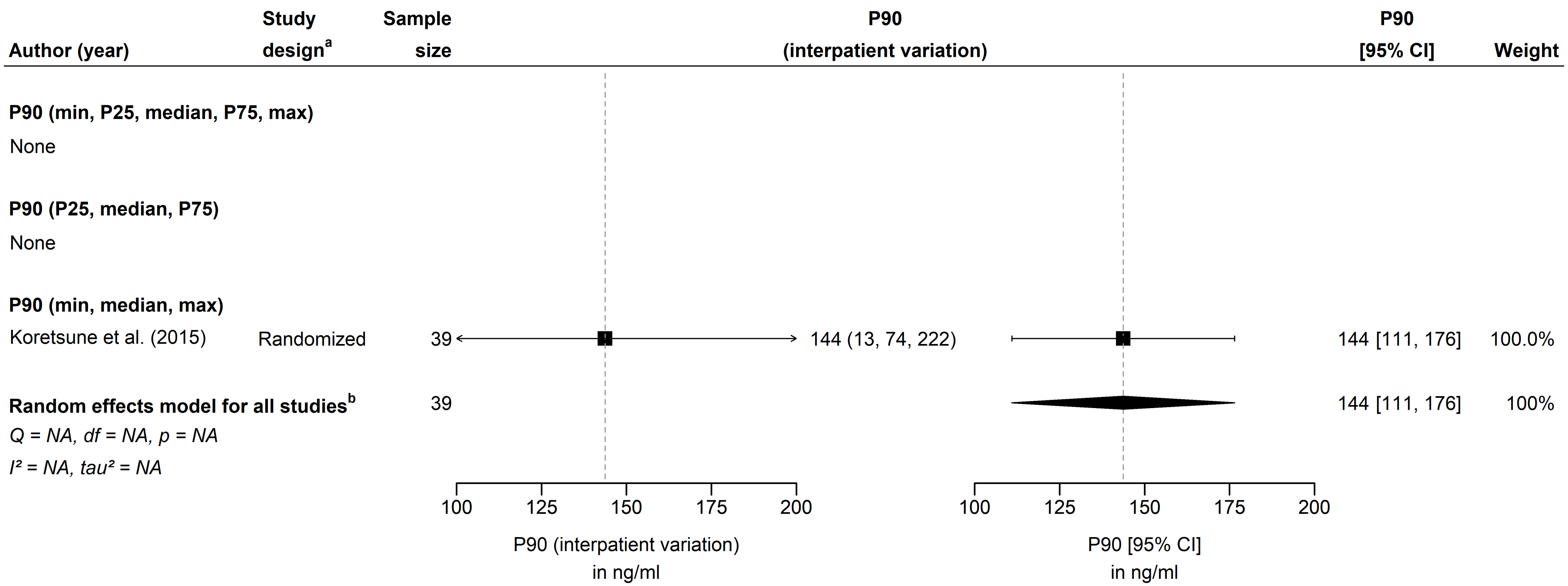

F. Edoxaban 15 mg once daily

[a] All analyses of interest were cross-sectional; [b] Random effects model using the quantile-estimation method;[54,57-59] [c] Simulated values were used because only the mean and standard deviation were available; [d] Simulated values were used because available parameters could not readily be included in the QE-method; [e] Percentiles were calculated directly from the original dataset if they were published by the authors of the current review.[4,12,37]

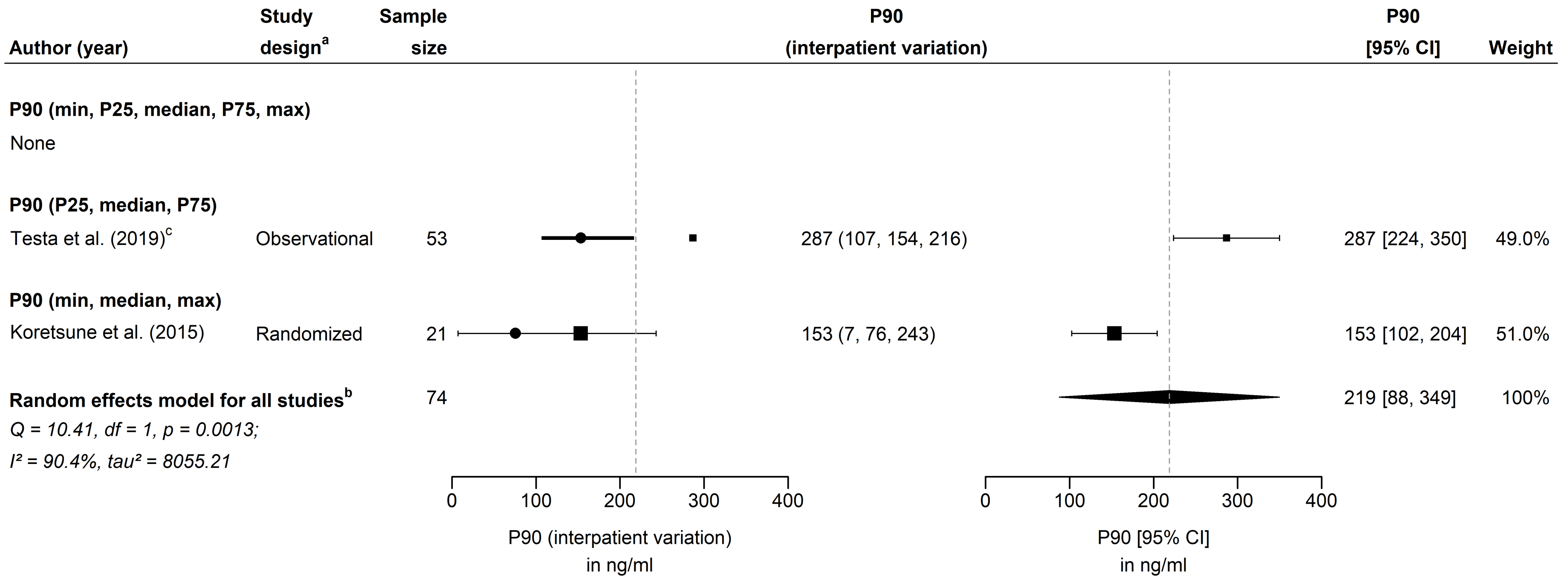

G. Edoxaban 30 mg once daily

[a] All analyses of interest were cross-sectional; [b] Random effects model using the quantile-estimation method;[54,57-59] [c] Simulated values were used because only the mean and standard deviation were available; [d] Simulated values were used because available parameters could not readily be included in the QE-method; [e] Percentiles were calculated directly from the original dataset if they were published by the authors of the current review.[4,12,37]

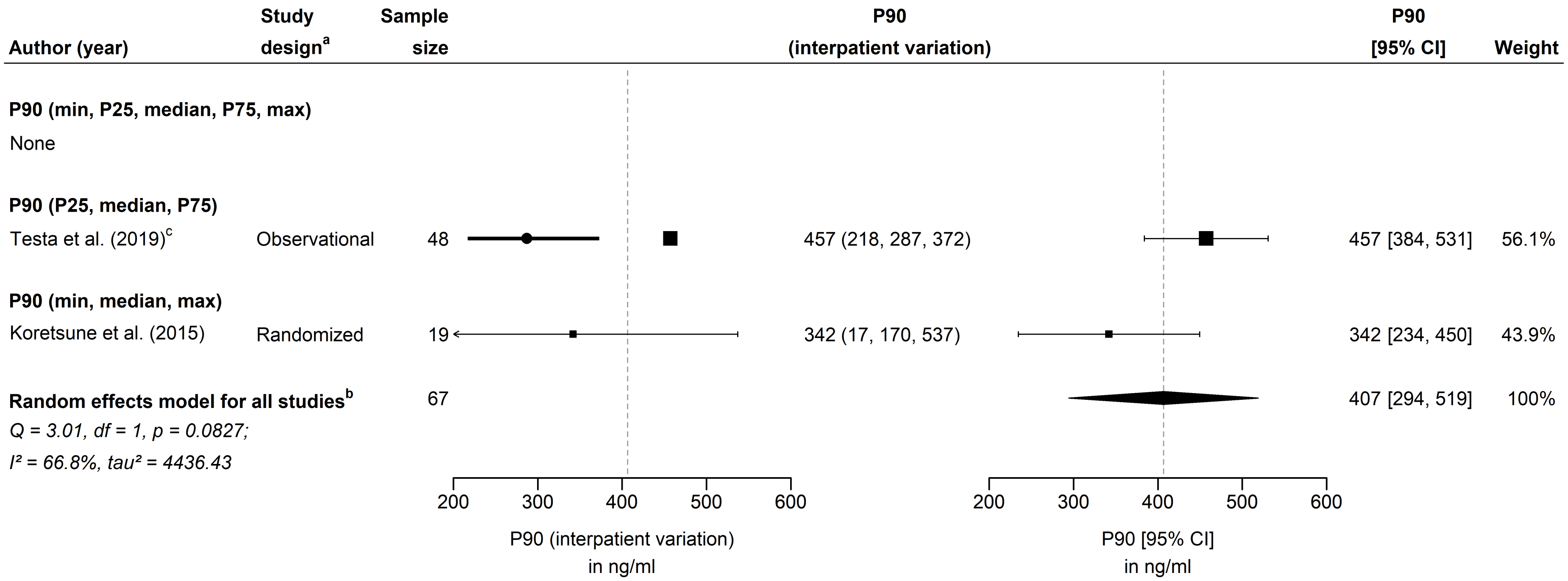

H. Edoxaban 60 mg once daily

[a] All analyses of interest were cross-sectional; [b] Random effects model using the quantile-estimation method;[54,57-59] [c] Simulated values were used because only the mean and standard deviation were available; [d] Simulated values were used because available parameters could not readily be included in the QE-method; [e] Percentiles were calculated directly from the original dataset if they were published by the authors of the current review.[4,12,37]

Author (year)	Study design[a]	Sample size	P90 (interpatient variation)	P90 [95% CI]	Weight
**P90 (min, P25, median, P75, max)**					
None					
**P90 (P25, median, P75)**					
Suwa et al. (2019)[d]	Observational	46	394 (213, 270, 328)	394 [338, 450]	44.5%
Hirota et al. (2020)[d]	Randomized	66	458 (186, 274, 380)	458 [392, 523]	34.9%
Wongcharoen et al. (2020)[d]	Observational	21	409 (194, 263, 342)	409 [317, 502]	19.4%
Takatsuki et al. (2017)[c]	Observational	4	350 (97, 151, 235)	350 [0, 733]	1.3%
**P90 (min, median, max)**					
None					
**Random effects model for all studies[b]**		137		418 [375, 462]	100%
Q = 2.29, df = 3, p = 0.5154;					
I² = 13.0%, tau² = 273.02					

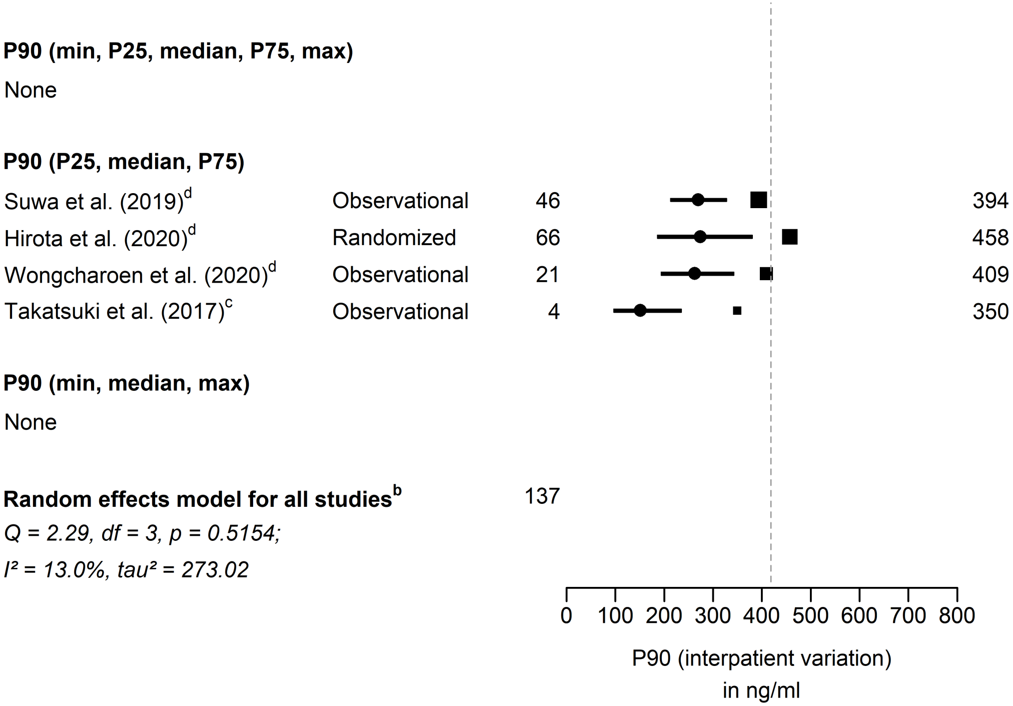

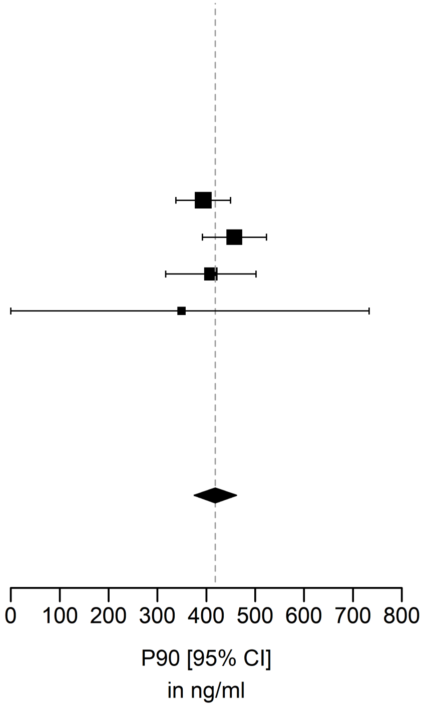

<u>I. Rivaroxaban 10 mg once daily</u>

[a] All analyses of interest were cross-sectional; [b] Random effects model using the quantile-estimation method;[54,57-59] [c] Simulated values were used because only the mean and standard deviation were available; [d] Simulated values were used because available parameters could not readily be included in the QE-method; [e] Percentiles were calculated directly from the original dataset if they were published by the authors of the current review.[4,12,37]

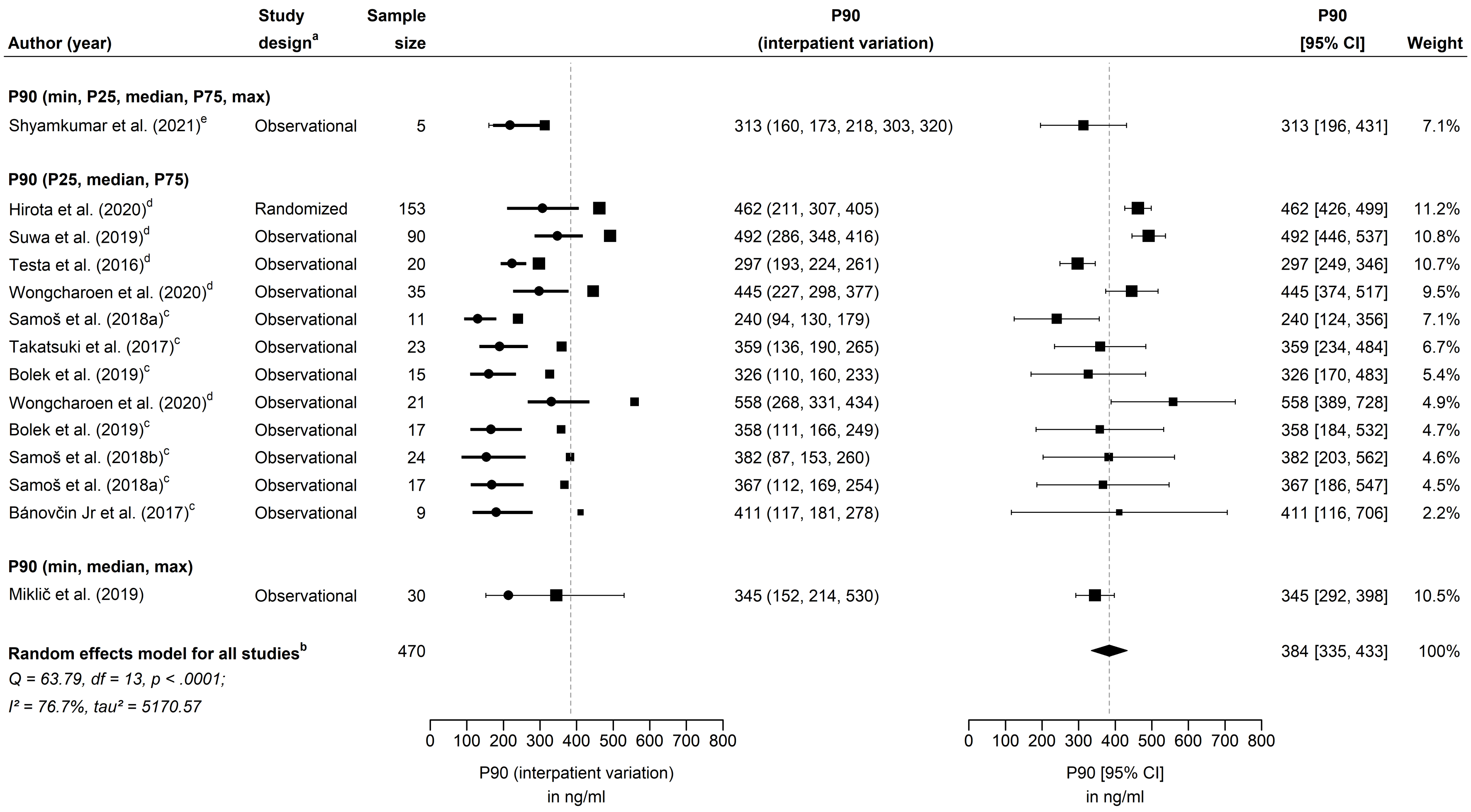

J. Rivaroxaban 15 mg once daily

[a] All analyses of interest were cross-sectional; [b] Random effects model using the quantile-estimation method;[54,57-59] [c] Simulated values were used because only the mean and standard deviation were available; [d] Simulated values were used because available parameters could not readily be included in the QE-method; [e] Percentiles were calculated directly from the original dataset if they were published by the authors of the current review.[4,12,37]

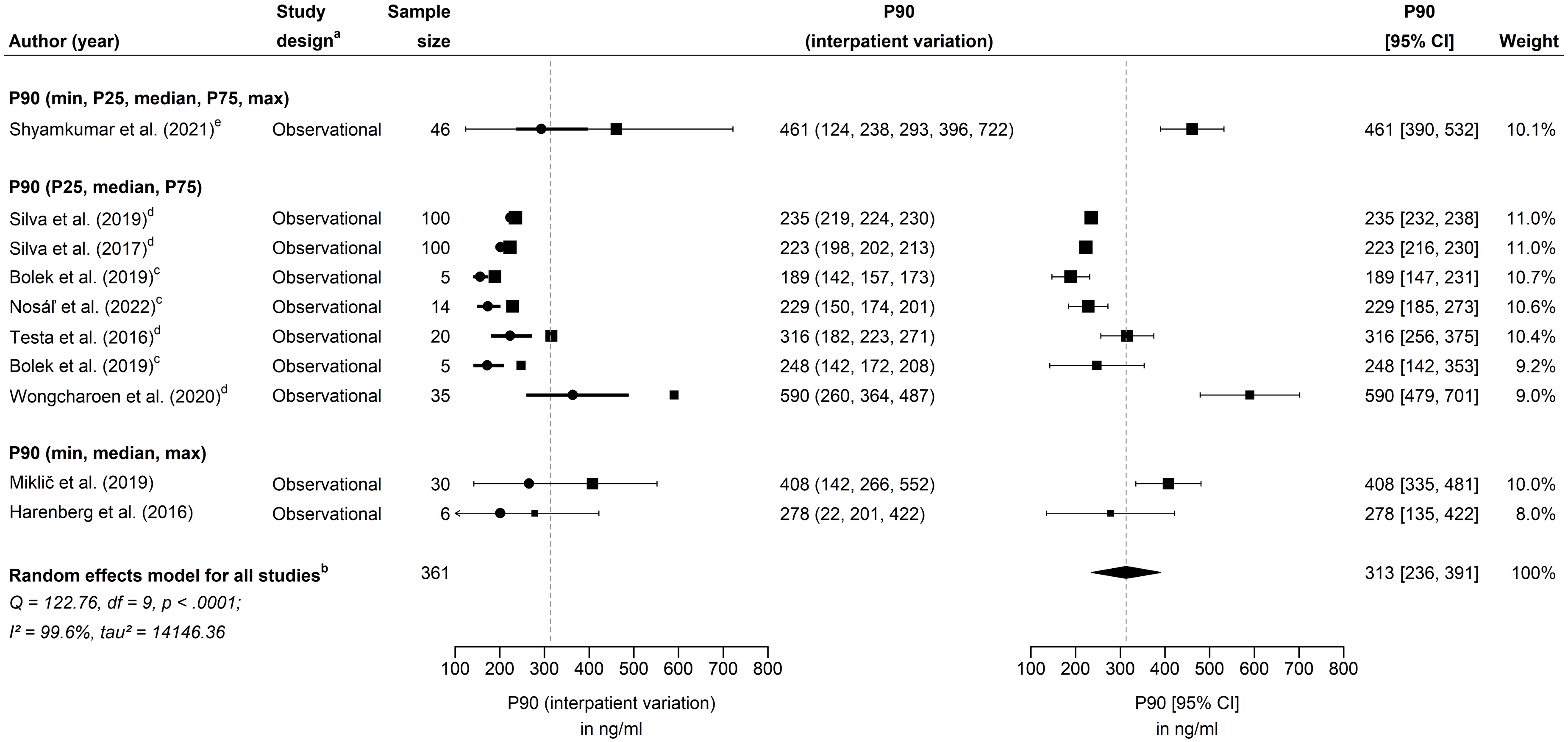

K. Rivaroxaban 20 mg once daily

[a] All analyses of interest were cross-sectional; [b] Random effects model using the quantile-estimation method;[54,57-59] [c] Simulated values were used because only the mean and standard deviation were available; [d] Simulated values were used because available parameters could not readily be included in the QE-method; [e] Percentiles were calculated directly from the original dataset if they were published by the authors of the current review.[4,12,37]

References

1. Al-Aieshy F, Malmström RE, Antovic J*, et al.* Clinical evaluation of laboratory methods to monitor exposure of rivaroxaban at trough and peak in patients with atrial fibrillation. *Eur J Clin Pharmacol*. 2016;72(6):671-679
2. Bánovčin P, Jr., Škorňová I, Samoš M*, et al.* Platelet Aggregation in Direct Oral Factor Xa Inhibitors-treated Patients With Atrial Fibrillation: A Pilot Study. *J Cardiovasc Pharmacol*. 2017;70(4):263-266
3. Bhagirath VC, Eikelboom JW, Hirsh J*, et al.* Apixaban-Calibrated Anti-FXa Activity in Relation to Outcome Events and Clinical Characteristics in Patients with Atrial Fibrillation: Results from the AVERROES Trial. *TH Open*. 2017;1(2):e139-e145
4. Bhagirath VC, Chan N, Hirsh J*, et al.* Plasma Apixaban Levels in Patients Treated Off Label With the Lower Dose. *J Am Coll Cardiol*. 2020;76(24):2906-2907
5. Bolek T, Samoš M, Škorňová I*, et al.* Dabigatran Levels in Elderly Patients with Atrial Fibrillation: First Post-Marketing Experiences. *Drugs Aging*. 2018;35(6):539-544
6. Bolek T, Samoš M, Škorňová I*, et al.* Does proton pump inhibition change the on-treatment anti-Xa activity in xabans-treated patients with atrial fibrillation? A pilot study. *J Thromb Thrombolysis*. 2019;47(1):140-145
7. Bolek T, Samoš M, Stančiaková L*, et al.* The impact of atorvastatin on dabigatran plasma levels in patients with atrial fibrillation. *Blood Coagul Fibrinolysis*. 2021;32(1):69-71
8. Boonen K, Schmitz E, Rozestraten F*, et al.* Real life dabigatran and metabolite concentrations, focused on inter-patient variability and assay differences in patients with atrial fibrillation. *Clin Chem Lab Med*. 2017;55(12):2002-2009
9. Chan NC, Coppens M, Hirsh J*, et al.* Real-world variability in dabigatran levels in patients with atrial fibrillation. *J Thromb Haemost*. 2015;13(3):353-359
10. Chang YT, Hu YF, Liao JN*, et al.* The assessment of anticoagulant activity to predict bleeding outcome in atrial fibrillation patients receiving dabigatran etexilate. *Blood Coagul Fibrinolysis*. 2016;27(4):389-395
11. Chaussade E, Hanon O, Boully C*, et al.* Real-Life Peak and Trough Dabigatran Plasma Measurements over Time in Hospitalized Geriatric Patients with Atrial Fibrillation. *J Nutr Health Aging*. 2018;22(1):165-173
12. de Vries TAC, Hirsh J, Bhagirath VC*, et al.* Can a Single Measurement of Apixaban Levels Identify Patients at Risk of Overexposure? A Prospective Cohort Study. *TH Open*. 2022;06(01):e10-e17

13. Harenberg J, Du S, Wehling M*, et al.* Measurement of dabigatran, rivaroxaban and apixaban in samples of plasma, serum and urine, under real life conditions. An international study. *Clin Chem Lab Med*. 2016;54(2):275-283
14. Hirota N, Suzuki S, Yamasaki M*, et al.* Analysis of bioMARKer Distribution and Individual Reproducibility Under Rivaroxaban Treatment in Japanese Patients with Non-Valvular Atrial Fibrillation (R-MARK Study, CVI ARO2). *Int Heart J*. 2020;61(4):695-704
15. Horinaka S, Sugawara R, Yonezawa Y, Ishimitsu T. Factor Xa inhibition by rivaroxaban in the trough steady state can significantly reduce thrombin generation. *Br J Clin Pharmacol*. 2018;84(1):79-87
16. Ji Q, Zhang C, Xu Q*, et al.* The impact of ABCB1 and CES1 polymorphisms on dabigatran pharmacokinetics and pharmacodynamics in patients with atrial fibrillation. *Br J Clin Pharmacol*. 2021;87(5):2247-2255
17. Koretsune Y, Yamashita T, Kimura T*, et al.* Short-Term Safety and Plasma Concentrations of Edoxaban in Japanese Patients With Non-Valvular Atrial Fibrillation and Severe Renal Impairment. *Circ J*. 2015;79(7):1486-1495
18. Lin SY, Tang SC, Kuo CH*, et al.* Factors affecting serum concentration of dabigatran in Asian patients with non-valvular atrial fibrillation. *J Formos Med Assoc*. 2019;118(7):1154-1160
19. Lin SY, Kuo CH, Yeh SJ*, et al.* Real-World Rivaroxaban and Apixaban Levels in Asian Patients With Atrial Fibrillation. *Clin Pharmacol Ther*. 2020;107(1):278-286
20. Liu Z, Xie Q, Xiang Q*, et al.* Anti-FXa-IIa activity test in Asian and its potential role for drug adherence evaluation in patients with direct oral anticoagulants: a nationwide multi-center synchronization study. *Cardiovasc Diagn Ther*. 2020;10(5):1293-1302
21. Martin JL, Esmaeili H, Manuel RC*, et al.* Pharmacokinetics/Pharmacodynamics of Dabigatran 75 mg Twice Daily in Patients With Nonvalvular Atrial Fibrillation and Severely Impaired Renal Function. *J Cardiovasc Pharmacol Ther*. 2018;23(5):399-406
22. Mavri A, Vene N, Božič-Mijovski M*, et al.* Apixaban concentration variability and relation to clinical outcomes in real-life patients with atrial fibrillation. *Sci Rep*. 2021;11(1):13908
23. Miklič M, Mavri A, Vene N*, et al.* Intra- and inter- individual rivaroxaban concentrations and potential bleeding risk in patients with atrial fibrillation. *Eur J Clin Pharmacol*. 2019;75(8):1069-1075
24. Mochalina N, Juhlin T, Platonov PG, Svensson PJ, Wieloch M. Concomitant use of dronedarone with dabigatran in patients with atrial fibrillation in clinical practice. *Thromb Res*. 2015;135(6):1070-1074

25. Mukai Y, Wada K, Miyamoto K, *et al.* The influence of residual apixaban on bleeding complications during and after catheter ablation of atrial fibrillation. *J Arrhythm*. 2017;33(5):434-439
26. Nakagawa J, Kinjo T, Iizuka M, *et al.* Impact of gene polymorphisms in drug-metabolizing enzymes and transporters on trough concentrations of rivaroxaban in patients with atrial fibrillation. *Basic Clin Pharmacol Toxicol*. 2021;128(2):297-304
27. Nissan R, Spectre G, Hershkovitz A, *et al.* Apixaban Levels in Octogenarian Patients with Non-valvular Atrial Fibrillation. *Drugs Aging*. 2019;36(2):165-177
28. Nosáľ V, Petrovičová A, Škorňová I, *et al.* Plasma levels of direct oral anticoagulants in atrial fibrillation patients at the time of embolic stroke: a pilot prospective multicenter study. *Eur J Clin Pharmacol*. 2022;78(4):557-564
29. Reilly PA, Lehr T, Haertter S, *et al.* The effect of dabigatran plasma concentrations and patient characteristics on the frequency of ischemic stroke and major bleeding in atrial fibrillation patients: the RE-LY Trial (Randomized Evaluation of Long-Term Anticoagulation Therapy). *J Am Coll Cardiol*. 2014;63(4):321-328
30. Roşian AN, Roşian Ş H, Kiss B, *et al.* Interindividual Variability of Apixaban Plasma Concentrations: Influence of Clinical and Genetic Factors in a Real-Life Cohort of Atrial Fibrillation Patients. *Genes (Basel)*. 2020;11(4)
31. Ruff CT, Giugliano RP, Braunwald E, *et al.* Association between edoxaban dose, concentration, anti-Factor Xa activity, and outcomes: an analysis of data from the randomised, double-blind ENGAGE AF-TIMI 48 trial. *Lancet*. 2015;385(9984):2288-2295
32. Samoš M, Bolek T, Stančiaková L, *et al.* Does type 2 diabetes affect the on-treatment levels of direct oral anticoagulants in patients with atrial fibrillation? *Diabetes Res Clin Pract*. 2018;135:172-177
33. Samoš M, Stančiaková L, Ivanková J, *et al.* Monitoring of dabigatran therapy using Hemoclot(®) Thrombin Inhibitor assay in patients with atrial fibrillation. *J Thromb Thrombolysis*. 2015;39(1):95-100
34. Samoš M, Bolek T, Stančiaková L, *et al.* Anti-Xa activity in oral factor Xa inhibitor-treated patients with atrial fibrillation and a higher risk of bleeding: a pilot study. *Blood Coagul Fibrinolysis*. 2018;29(4):369-373
35. Schnierer M, Samoš M, Bolek T, *et al.* The Effect of Proton Pump Inhibitor Withdrawal on Dabigatran Etexilate Plasma Levels in Patients With Atrial Fibrillation: A Washout Study. *J Cardiovasc Pharmacol*. 2020;75(4):333-335
36. Shin H, Cho MC, Kim RB, *et al.* Laboratory measurement of apixaban using anti-factor Xa assays in acute ischemic stroke patients with non-valvular atrial fibrillation. *J Thromb Thrombolysis*. 2018;45(2):250-256

37. Shyamkumar K, Hirsh J, Bhagirath VC*, et al.* Plasma Rivaroxaban Level to Identify Patients at Risk of Drug Overexposure: Is a Single Measurement of Drug Level Reliable? *TH Open*. 2021;5(1):e84-e88
38. Silva VM, Scanavacca M, Darrieux F, Cavalheiro-Filho C, Strunz CC. Effects of rivaroxaban on coagulation tests in patients with non-valvular atrial fibrillation under real-life conditions. *Thromb Res*. 2017;154:26-27
39. Silva VM, Scanavacca M, Darrieux F, Cavalheiro C, Strunz CC. Routine Coagulation Tests in Patients With Nonvalvular Atrial Fibrillation Under Dabigatran and Rivaroxaban Therapy: An Affordable and Reliable Strategy? *Clin Appl Thromb Hemost*. 2019;25:1076029619835053
40. Šinigoj P, Malmström RE, Vene N*, et al.* Dabigatran Concentration: Variability and Potential Bleeding Prediction In "Real-Life" Patients With Atrial Fibrillation. *Basic Clin Pharmacol Toxicol*. 2015;117(5):323-329
41. Skeppholm M, Hjemdahl P, Antovic JP*, et al.* On the monitoring of dabigatran treatment in "real life" patients with atrial fibrillation. *Thromb Res*. 2014;134(4):783-789
42. Skeppholm M, Al-Aieshy F, Berndtsson M*, et al.* Clinical evaluation of laboratory methods to monitor apixaban treatment in patients with atrial fibrillation. *Thromb Res*. 2015;136(1):148-153
43. Skripka A, Sychev D, Bochkov P*, et al.* Factors Affecting Trough Plasma Dabigatran Concentrations in Patients with Atrial Fibrillation and Chronic Kidney Disease. *High Blood Press Cardiovasc Prev*. 2020;27(2):151-156
44. Suwa M, Morii I, Kino M. Rivaroxaban or Apixaban for Non-Valvular Atrial Fibrillation - Efficacy and Safety of Off-Label Under-Dosing According to Plasma Concentration. *Circ J*. 2019;83(5):991-999
45. Suzuki S, Yamashita T, Akao M, Okumura K. Clinical implications of assessment of apixaban levels in elderly atrial fibrillation patients: J-ELD AF registry sub-cohort analysis. *Eur J Clin Pharmacol*. 2020;76(8):1111-1124
46. Takatsuki S, Kimura T, Sugimoto K*, et al.* Real-world monitoring of direct oral anticoagulants in clinic and hospitalization settings. *SAGE Open Med*. 2017;5:2050312117734773
47. Taune V, Wallén H, Ågren A*, et al.* Whole blood coagulation assays ROTEM and T-TAS to monitor dabigatran treatment. *Thromb Res*. 2017;153:76-82
48. Testa S, Tripodi A, Legnani C*, et al.* Plasma levels of direct oral anticoagulants in real life patients with atrial fibrillation: Results observed in four anticoagulation clinics. *Thromb Res*. 2016;137:178-183

49. Testa S, Legnani C, Antonucci E*, et al.* Drug levels and bleeding complications in atrial fibrillation patients treated with direct oral anticoagulants. *J Thromb Haemost*. 2019;17(7):1064-1072
50. Tomita H, Araki T, Kadokami T*, et al.* Factors influencing trough and 90-minute plasma dabigatran etexilate concentrations among patients with non-valvular atrial fibrillation. *Thromb Res*. 2016;145:100-106
51. Wongcharoen W, Pacharasupa P, Norasetthada L, Gunaparn S, Phrommintikul A. Anti-Factor Xa Activity of Standard and Japan-Specific Doses of Rivaroxaban in Thai Patients With Non-Valvular Atrial Fibrillation. *Circ J*. 2020;84(7):1075-1082
52. Zhang C, Zhang P, Li H*, et al.* The effect of dabigatran on thrombin generation and coagulation assays in rabbit and human plasma. *Thromb Res*. 2018;165:38-43
53. Zhu Z, Shen Z, Shi A*, et al.* Dabigatran plasma concentration indicated the risk of patients with non-valvular atrial fibrillation. *Heart Vessels*. 2022;37(5):821-827
54. McGrath S, Sohn H, Steele R, Benedetti A. Meta-analysis of the difference of medians. *Biom J*. 2020;62(1):69-98
55. McGrath S, Zhao X, Ozturk O*, et al.* metamedian: An R package for meta-analyzing studies reporting medians. *Res Synth Methods, in press*. 2023
56. Guyatt GH, Oxman AD, Schünemann HJ, Tugwell P, Knottnerus A. GRADE guidelines: A new series of articles in the Journal of Clinical Epidemiology. *Journal of Clinical Epidemiology*. 2011;64(4):380-382
57. McGrath S, Zhao X, Qin ZZ, Steele R, Benedetti A. One-sample aggregate data meta-analysis of medians. *Stat Med*. 2019;38(6):969-984
58. McGrath S, Zhao X, Steele R, Thombs BD, Benedetti A. Estimating the sample mean and standard deviation from commonly reported quantiles in meta-analysis. *Stat Methods Med Res*. 2020;29(9):2520-2537
59. Borenstein M, Hedges LV, Higgins JP, Rothstein HR. A basic introduction to fixed-effect and random-effects models for meta-analysis. *Res Synth Methods*. 2010;1(2):97-111
