# Supplementary material for: Usual On-therapy Ranges of Drug Concentrations in Patients with Atrial Fibrillation Treated with Direct Oral Anticoagulants: A Systematic Review and Meta-analysis
Source: Thromb Haemost. 2024 Nov 21;125(6):563–73. doi: 10.1055/a-2446-1348 (PMC12115550; doi:10.1055/a-2446-1348)
Supplement: Supplementary file 5 — Supporting Information File 5 [file 10-1055-a-2446-1348-s24030110-5.pdf]

Supporting Information File 5 — Sensitivity analyses

Usual On-therapy Ranges of Drug Concentrations in Patients With Atrial Fibrillation Treated With Direct Oral Anticoagulants: a Systematic Review and Meta-analysis

Last updated on: August 7, 2024

Table of contents

Sensitivity analysis 1: Risk of bias and concern of inapplicability 10

Table S1. Summary of sensitivity analysis 1: risk of bias and concern of inapplicability....10

Figure S1. Sensitivity analysis 1 (risk of bias and concern of inapplicability): Estimating the pooled 10th percentile of trough levels of each direct oral anticoagulant stratified by administered dose using the modified QE-method 12

A. Apixaban 2.5 mg twice daily..13

B. Apixaban 5 mg twice daily...13

C. Dabigatran 75 mg twice daily..13

D. Dabigatran 110 mg twice daily..14

E. Dabigatran 150 mg twice daily 15

F. Edoxaban 15 mg once daily 16

G. Edoxaban 30 mg once daily...16

H. Edoxaban 60 mg once daily...16

I. Rivaroxaban 10 mg once daily 16

J. Rivaroxaban 15 mg once daily..17

K. Rivaroxaban 20 mg once daily 18

Figure S2. Sensitivity analysis 1 (risk of bias and concern of inapplicability): Estimating the pooled 90th percentile of trough levels of each direct oral anticoagulant stratified by administered dose using the modified QE-method 19

A. Apixaban 2.5 mg twice daily..20

B. Apixaban 5 mg twice daily...20

C. Dabigatran 75 mg twice daily..20

D. Dabigatran 110 mg twice daily..21

E. Dabigatran 150 mg twice daily 22
F. Edoxaban 15 mg once daily 23
G. Edoxaban 30 mg once daily....................23
H. Edoxaban 60 mg once daily....................23
I. Rivaroxaban 10 mg once daily 23
J. Rivaroxaban 15 mg once daily....................24
K. Rivaroxaban 20 mg once daily 25

Figure S3. Sensitivity analysis 1 (risk of bias and concern of inapplicability): Estimating the pooled 10th percentile of peak levels of each direct oral anticoagulant stratified by administered dose using the modified QE-method 26

A. Apixaban 2.5 mg twice daily....................27
B. Apixaban 5 mg twice daily....................27
C. Dabigatran 75 mg twice daily....................27
D. Dabigatran 110 mg twice daily....................28
E. Dabigatran 150 mg twice daily 29
F. Edoxaban 15 mg once daily 30
G. Edoxaban 30 mg once daily....................30
H. Edoxaban 60 mg once daily....................30
I. Rivaroxaban 10 mg once daily 30
J. Rivaroxaban 15 mg once daily....................31
K. Rivaroxaban 20 mg once daily 32

Figure S4. Sensitivity analysis 1 (risk of bias and concern of inapplicability): Estimating the pooled 90th percentile of peak levels of each direct oral anticoagulant stratified by administered dose using the modified QE-method 33

A. Apixaban 2.5 mg twice daily....34
B. Apixaban 5 mg twice daily....34
C. Dabigatran 75 mg twice daily....34
D. Dabigatran 110 mg twice daily....35
E. Dabigatran 150 mg twice daily....36
F. Edoxaban 15 mg once daily....37
G. Edoxaban 30 mg once daily....37
H. Edoxaban 60 mg once daily....37
I. Rivaroxaban 10 mg once daily....37
J. Rivaroxaban 15 mg once daily....38
K. Rivaroxaban 20 mg once daily....39
Sensitivity analysis 2: Data extraction method....40
Table S2. Summary of sensitivity analysis 2: Data extraction method....40
Figure S5. Sensitivity analysis 2 (by data extraction method): Estimating the pooled 10th percentile of trough levels of each direct oral anticoagulant stratified by administered dose using the modified QE-method....42
A. Apixaban 2.5 mg twice daily....43
B. Apixaban 5 mg twice daily....44
C. Dabigatran 75 mg twice daily....45
D. Dabigatran 110 mg twice daily....46
E. Dabigatran 150 mg twice daily....47
F. Edoxaban 15 mg once daily....48
G. Edoxaban 30 mg once daily....48

H. Edoxaban 60 mg once daily..48

I. Rivaroxaban 10 mg once daily 48

J. Rivaroxaban 15 mg once daily...49

K. Rivaroxaban 20 mg once daily 50

Figure S6. Sensitivity analysis 2 (by data extraction method): Estimating the pooled 90th percentile of trough levels of each direct oral anticoagulant stratified by administered dose using the modified QE-method...51

A. Apixaban 2.5 mg twice daily..52

B. Apixaban 5 mg twice daily...53

C. Dabigatran 75 mg twice daily 54

D. Dabigatran 110 mg twice daily 55

E. Dabigatran 150 mg twice daily 56

F. Edoxaban 15 mg once daily 57

G. Edoxaban 30 mg once daily..57

H. Edoxaban 60 mg once daily..57

I. Rivaroxaban 10 mg once daily 57

J. Rivaroxaban 15 mg once daily...58

K. Rivaroxaban 20 mg once daily 59

Figure S7. Sensitivity analysis 2 (by data extraction method): Estimating the pooled 10th percentile of peak levels of each direct oral anticoagulant stratified by administered dose using the modified QE-method...60

A. Apixaban 2.5 mg twice daily..61

B. Apixaban 5 mg twice daily...62

C. Dabigatran 75 mg twice daily 63

D. Dabigatran 110 mg twice daily 64
E. Dabigatran 150 mg twice daily 65
F. Edoxaban 15 mg once daily 66
G. Edoxaban 30 mg once daily..66
H. Edoxaban 60 mg once daily..66
I. Rivaroxaban 10 mg once daily 66
J. Rivaroxaban 15 mg once daily..67
K. Rivaroxaban 20 mg once daily 68
Figure S8. Sensitivity analysis 2 (by data extraction method): Estimating the pooled 90th percentile of peak levels of each direct oral anticoagulant stratified by administered dose using the modified QE-method..69
A. Apixaban 2.5 mg twice daily..70
B. Apixaban 5 mg twice daily..71
C. Dabigatran 75 mg twice daily..72
D. Dabigatran 110 mg twice daily..73
E. Dabigatran 150 mg twice daily 74
F. Edoxaban 15 mg once daily 75
G. Edoxaban 30 mg once daily..75
H. Edoxaban 60 mg once daily..75
I. Rivaroxaban 10 mg once daily 75
J. Rivaroxaban 15 mg once daily..76
K. Rivaroxaban 20 mg once daily 77
Sensitivity analysis 3: Laboratory method..78

Table S3. Summary of sensitivity analysis 3: Laboratory method (post hoc defined)........78

Figure S9. Sensitivity analysis 3 (by laboratory method): Estimating the pooled 10th percentile of trough levels of each direct oral anticoagulant stratified by administered dose using the modified QE-method..80

A. Apixaban 2.5 mg twice daily..81

B. Apixaban 5 mg twice daily...82

C. Dabigatran 75 mg twice daily...83

D. Dabigatran 110 mg twice daily...84

E. Dabigatran 150 mg twice daily...85

F. Edoxaban 15 mg once daily...86

G. Edoxaban 30 mg once daily...86

H. Edoxaban 60 mg once daily...86

I. Rivaroxaban 10 mg once daily...86

J. Rivaroxaban 15 mg once daily...87

K. Rivaroxaban 20 mg once daily..88

Figure S10. Sensitivity analysis 3 (by laboratory method): Estimating the pooled 90th percentile of trough levels of each direct oral anticoagulant stratified by administered dose using the modified QE-method..89

A. Apixaban 2.5 mg twice daily..90

B. Apixaban 5 mg twice daily...91

C. Dabigatran 75 mg twice daily...92

D. Dabigatran 110 mg twice daily...93

E. Dabigatran 150 mg twice daily...94

F. Edoxaban 15 mg once daily...95

G. Edoxaban 30 mg once daily 95

H. Edoxaban 60 mg once daily 95

I. Rivaroxaban 10 mg once daily 95

J. Rivaroxaban 15 mg once daily 96

K. Rivaroxaban 20 mg once daily 97

Figure S11. Sensitivity analysis 3 (by laboratory method): Estimating the pooled 10th percentile of peak levels of each direct oral anticoagulant stratified by administered dose using the modified QE-method 98

A. Apixaban 2.5 mg twice daily 99

B. Apixaban 5 mg twice daily 100

C. Dabigatran 75 mg twice daily 101

D. Dabigatran 110 mg twice daily 102

E. Dabigatran 150 mg twice daily 103

F. Edoxaban 15 mg once daily 104

G. Edoxaban 30 mg once daily 104

H. Edoxaban 60 mg once daily 104

I. Rivaroxaban 10 mg once daily 104

J. Rivaroxaban 15 mg once daily 105

K. Rivaroxaban 20 mg once daily 106

Figure S12. Sensitivity analysis 3 (by laboratory method): Estimating the pooled 90th percentile of peak levels of each direct oral anticoagulant stratified by administered dose using the modified QE-method 107

A. Apixaban 2.5 mg twice daily 108

B. Apixaban 5 mg twice daily 109

C. Dabigatran 75 mg twice daily 110

D. Dabigatran 110 mg twice daily 111

E. Dabigatran 150 mg twice daily 112

F. Edoxaban 15 mg once daily 113

G. Edoxaban 30 mg once daily 113

H. Edoxaban 60 mg once daily 113

I. Rivaroxaban 10 mg once daily 113

J. Rivaroxaban 15 mg once daily 114

K. Rivaroxaban 20 mg once daily 115

References 116

Sensitivity analysis 1: Risk of bias and concern of inapplicability

Table S1. Summary of sensitivity analysis 1: risk of bias and concern of inapplicability

	**Pooled 10th percentile [95% CI]**				**Pooled 90th percentile [95% CI]**			
**DOAC dosing regimen**	**Low risk and concern[a]**	**Other[a]**	**Total**	***p*-value[b]**	**Low risk and concern[a]**	**Other[a]**	**Total**	***p*-value[b]**
**Trough**								
Apixaban								
2.5 mg twice daily	NA	NA	NA	NA	NA	NA	NA	NA
5 mg twice daily	NA	NA	NA	NA	NA	NA	NA	NA
Dabigatran								
75 mg twice daily	NA	NA	NA	NA	NA	NA	NA	NA
110 mg twice daily	46 [20, 72]	34 [29, 39]	34 [29, 39]	0.3816	204 [86, 322]	136 [117, 155]	136 [117, 155]	0.2633
150 mg twice daily	15 [0, 36]	34 [29, 39]	34 [29, 39]	0.0983	168 [88, 248]	150 [128, 173]	150 [128, 173]	0.6781
Edoxaban								
15 mg once daily	NA	NA	NA	NA	NA	NA	NA	NA
30 mg once daily	NA	NA	NA	NA	NA	NA	NA	NA
60 mg once daily	NA	NA	NA	NA	NA	NA	NA	NA
Rivaroxaban								
10 mg once daily	NA	NA	NA	NA	NA	NA	NA	NA
15 mg once daily	18 [9, 27]	16 [13, 18]	16 [13, 18]	0.5850	83 [35, 131]	73 [55, 91]	73 [55, 91]	0.6931
20 mg once daily	NA	NA	NA	NA	NA	NA	NA	NA
**Peak**								
Apixaban								
2.5 mg twice daily	NA	NA	NA	NA	NA	NA	NA	NA
5 mg twice daily	NA	NA	NA	NA	NA	NA	NA	NA
Dabigatran								

	Pooled 10th percentile [95% CI]				Pooled 90th percentile [95% CI]			
**DOAC dosing regimen**	**Low risk and concern[a]**	**Other[a]**	**Total**	***p*-value[b]**	**Low risk and concern[a]**	**Other[a]**	**Total**	***p*-value[b]**
75 mg twice daily	NA	NA	NA	NA	NA	NA	NA	NA
110 mg twice daily	94 [36, 152]	63 [49, 78]	63 [49, 78]	0.3173	374 [188, 561]	214 [173, 254]	214 [173, 254]	0.0990
150 mg twice daily	77 [42, 112]	76 [62, 90]	76 [62, 90]	0.9691	237 [131, 343]	294 [248, 340]	294 [248, 340]	0.3338
Edoxaban								
15 mg once daily	NA	NA	NA	NA	NA	NA	NA	NA
30 mg once daily	NA	NA	NA	NA	NA	NA	NA	NA
60 mg once daily	NA	NA	NA	NA	NA	NA	NA	NA
Rivaroxaban								
10 mg once daily	NA	NA	NA	NA	NA	NA	NA	NA
15 mg once daily	163 [74, 253]	125 [90, 161]	125 [90, 161]	0.4410	332 [216, 448]	395 [341, 449]	395 [341, 449]	0.3365
20 mg once daily	200 [169, 231]	154 [130, 177]	154 [130, 177]	**0.0208**	364 [223, 505]	290 [196, 385]	290 [196, 385]	0.3948

This table summarizes the results of the sensitivity analyses on the risk of bias and concern of inapplicability (i.e., low risk of bias and low concern of inapplicability vs. other studies), to assess the robustness of our findings on the 10th and 90th percentiles. We defined a significant difference as a *p*-value of <0.05 of the test of moderators (**bold** in table), though we acknowledge this has major limitations. We urge readers to appraise the individual forest plots (**Fig S1 through S4**).

NA not applicable.

[a] We kept Tau constant if there were five or fewer studies in either category;[1] [b] P-value of the test of moderators (two-tailed Wald-test).

Figure S1. Sensitivity analysis 1 (risk of bias and concern of inapplicability): Estimating the pooled 10th percentile of trough levels of each direct oral anticoagulant stratified by administered dose using the modified QE-method

The forest plots below illustrate the results of our analyses to estimate the 10th percentile of trough levels of each DOAC type, stratified by dosing regimen and by risk of bias and concern of inapplicability classification (i.e., low risk of bias and low concern of inapplicability vs. other classifications). The squares represent the 10th percentile values, the circles the median values, the solid bold lines the 25th to 75th percentile range, and the whiskers either the minimum to maximum value interval (left side of the plot) or the 95% of the confidence interval of the percentile value of interest (right side of the plot).

A. Apixaban 2.5 mg twice daily

Performing a sensitivity analysis was not possible because none of the 17 studies were at both low risk of bias and low concern of inapplicability.

B. Apixaban 5 mg twice daily

Performing a sensitivity analysis was not possible because none of the 19 studies were at both low risk of bias and low concern of inapplicability.

C. Dabigatran 75 mg twice daily

Performing a sensitivity analysis was not possible because only a single study was available.

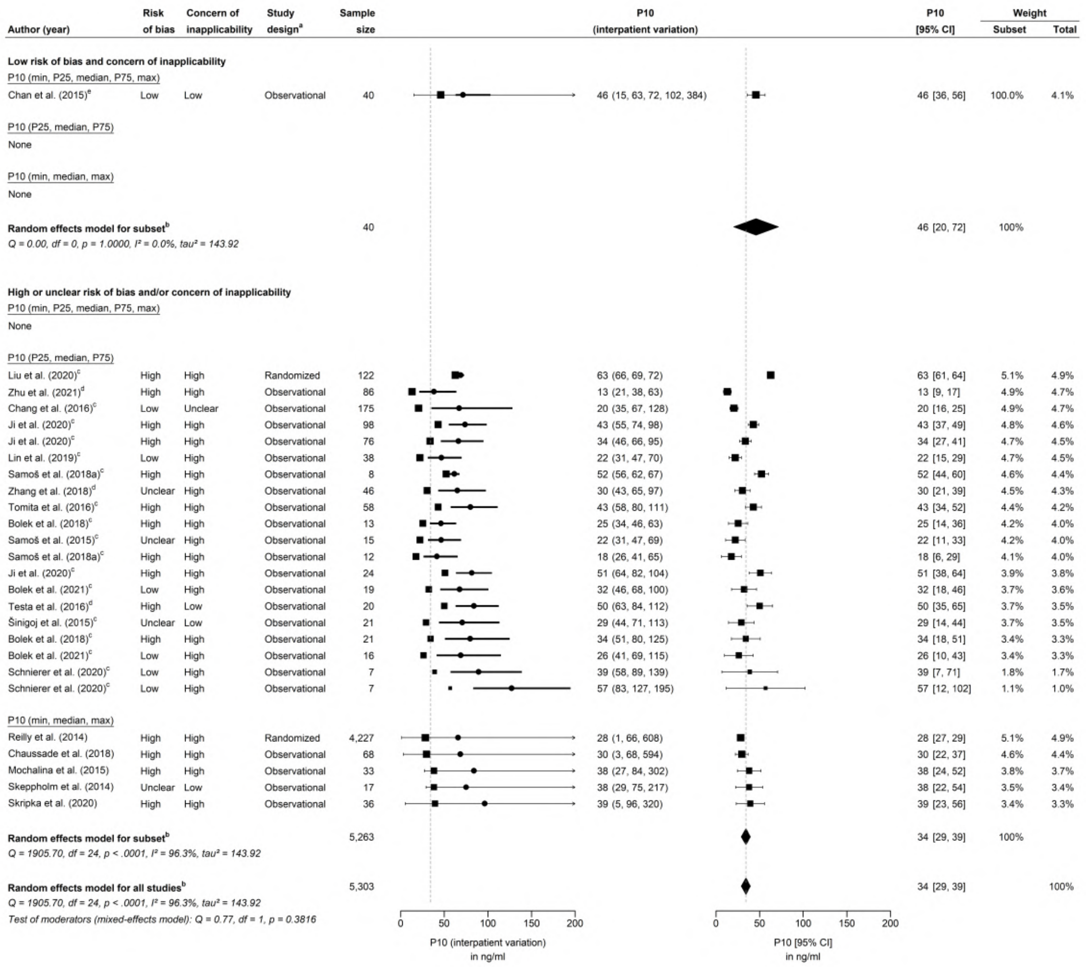

D. Dabigatran 110 mg twice daily

[a] All analyses of interest were cross-sectional; [b] Random effects model using the quantile-estimation method;[2-5] [c] Simulated values were used because only the mean and standard deviation were available; [d] Simulated values were used because available parameters could not readily be included in the QE-method; [e] Percentiles were calculated directly from the original dataset if they were published by the authors of the current review.[6-8]

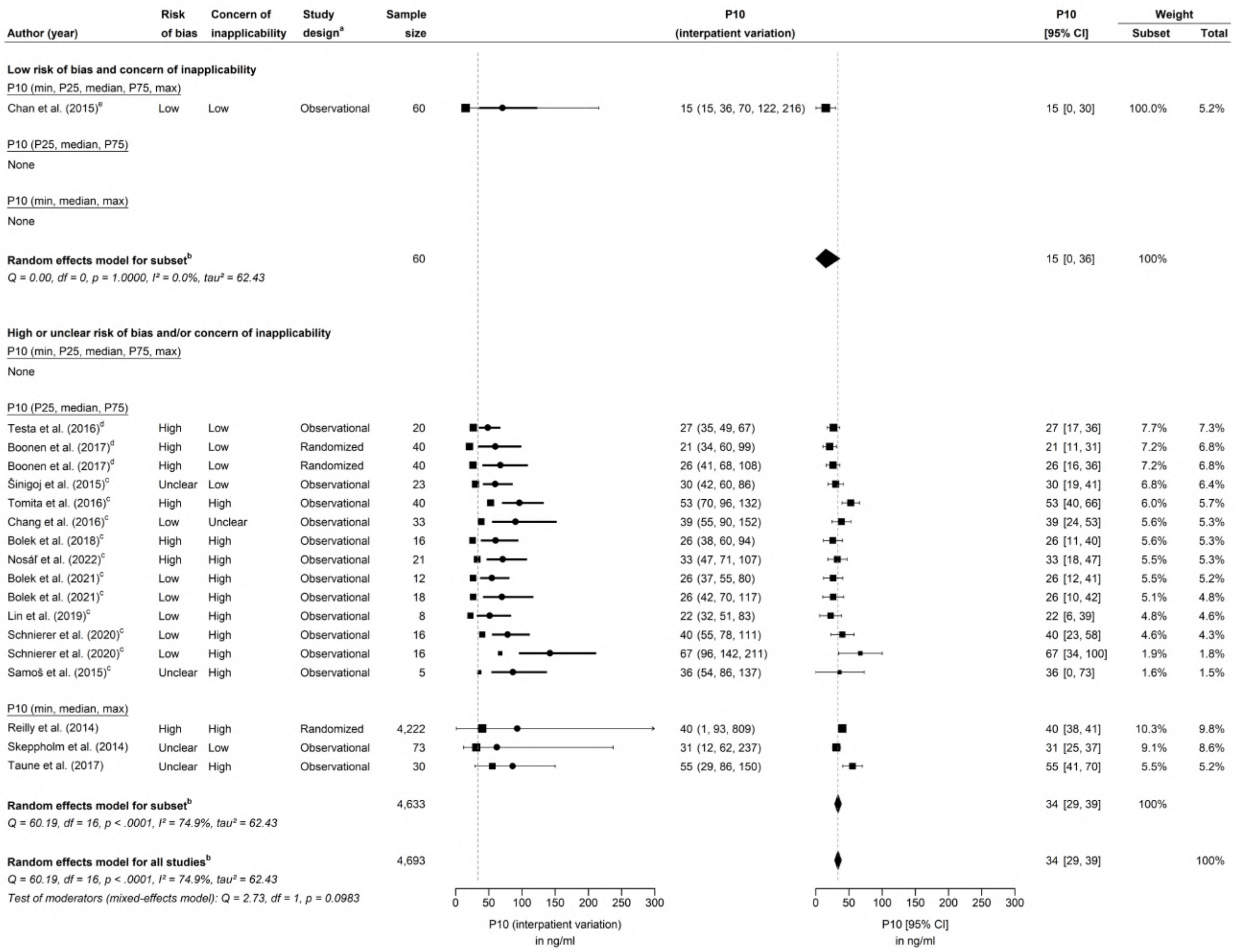

E. Dabigatran 150 mg twice daily

[a] All analyses of interest were cross-sectional; [b] Random effects model using the quantile-estimation method;[2-5] [c] Simulated values were used because only the mean and standard deviation were available; [d] Simulated values were used because available parameters could not readily be included in the QE-method; [e] Percentiles were calculated directly from the original dataset if they were published by the authors of the current review.[6-8]

F. Edoxaban 15 mg once daily

Performing a sensitivity analysis was not possible because only two studies were available, and both were not at low risk of bias and low concern of inapplicability.

G. Edoxaban 30 mg once daily

We did not perform a sensitivity analysis because fewer than ten studies were available (see **Supporting Information File 2**).[1] One study was at low risk of bias and low concern of inapplicability and three received other classifications.

H. Edoxaban 60 mg once daily

We did not perform a sensitivity analysis because fewer than ten studies were available (see **Supporting Information File 2**).[1] One study was at low risk of bias and low concern of inapplicability and two received other classifications.

I. Rivaroxaban 10 mg once daily

Performing a sensitivity analysis was not possible because only four studies were available, and all were not at low risk of bias and low concern of inapplicability.

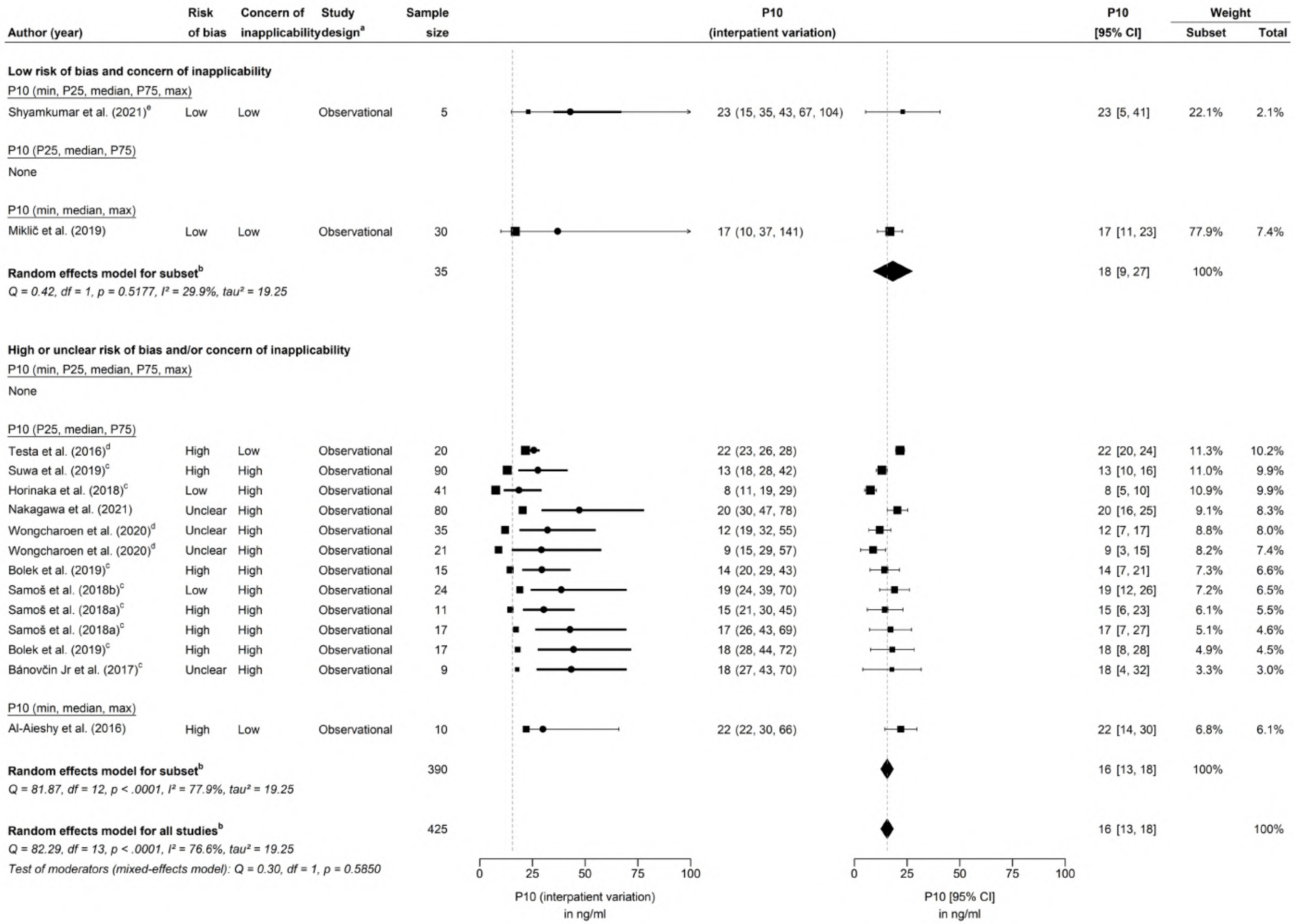

J. Rivaroxaban 15 mg once daily

[a] All analyses of interest were cross-sectional; [b] Random effects model using the quantile-estimation method;[2-5] [c] Simulated values were used because only the mean and standard deviation were available; [d] Simulated values were used because available parameters could not readily be included in the QE-method; [e] Percentiles were calculated directly from the original dataset if they were published by the authors of the current review.[6-8]

K. Rivaroxaban 20 mg once daily

We did not perform a sensitivity analysis because fewer than ten studies were available (see **Supporting Information File 2**).[1] Two studies were at low risk of bias and low concern of inapplicability and six received other classifications.

Figure S2. Sensitivity analysis 1 (risk of bias and concern of inapplicability): Estimating the pooled 90th percentile of trough levels of each direct oral anticoagulant stratified by administered dose using the modified QE-method

The forest plots below illustrate the results of our analyses to estimate the 90th percentile of trough levels of each DOAC type, stratified by dosing regimen and by risk of bias and concern of inapplicability classification (i.e., low risk of bias and low concern of inapplicability vs. other classifications). The squares represent the 90th percentile values, the circles the median values, the solid bold lines the 25th to 75th percentile range, and the whiskers either the minimum to maximum value interval (left side of the plot) or the 95% of the confidence interval of the percentile value of interest (right side of the plot).

A. Apixaban 2.5 mg twice daily

Performing a sensitivity analysis was not possible because none of the 17 studies were at both low risk of bias and low concern of inapplicability.

B. Apixaban 5 mg twice daily

Performing a sensitivity analysis was not possible because none of the 19 studies were at both low risk of bias and low concern of inapplicability.

C. Dabigatran 75 mg twice daily

Performing a sensitivity analysis was not possible because only a single study was available.

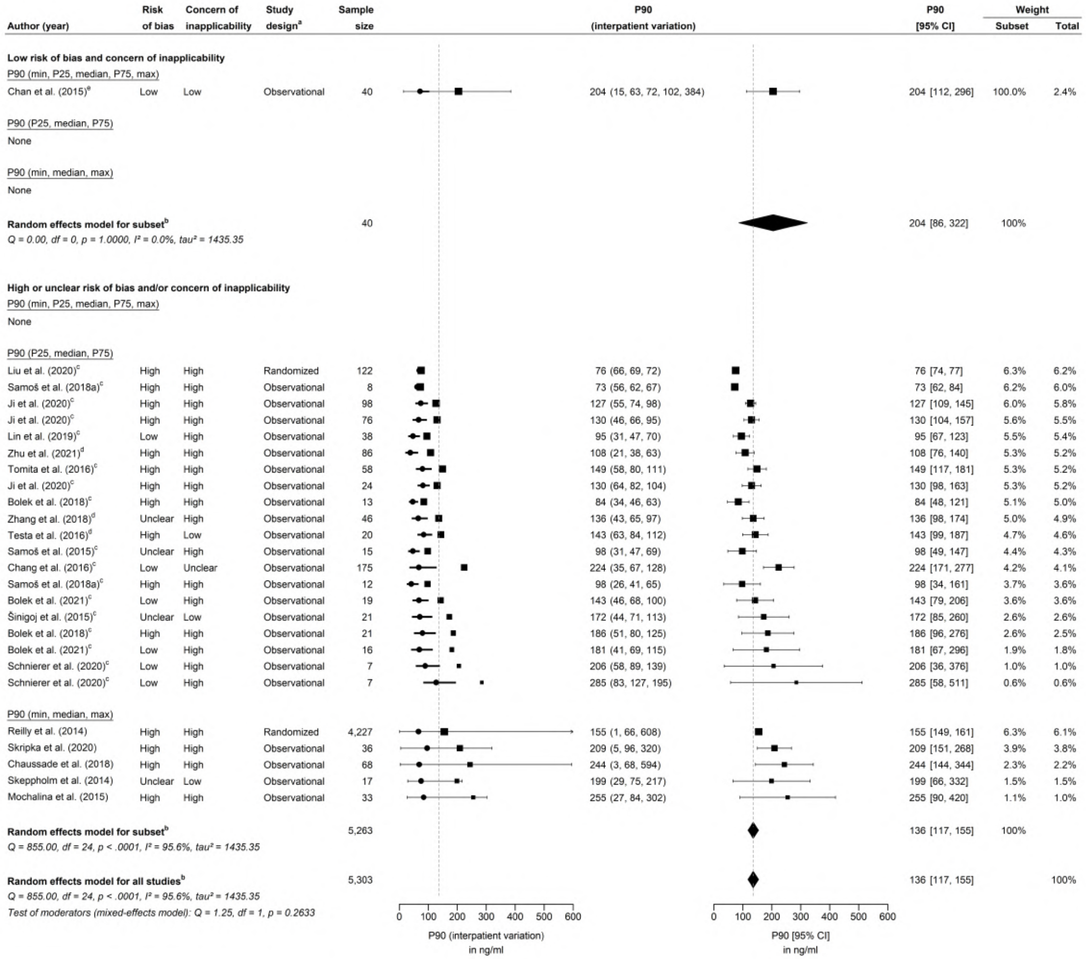

D. Dabigatran 110 mg twice daily

[a] All analyses of interest were cross-sectional; [b] Random effects model using the quantile-estimation method;[2-5] [c] Simulated values were used because only the mean and standard deviation were available; [d] Simulated values were used because available parameters could not readily be included in the QE-method; [e] Percentiles were calculated directly from the original dataset if they were published by the authors of the current review.[6-8]

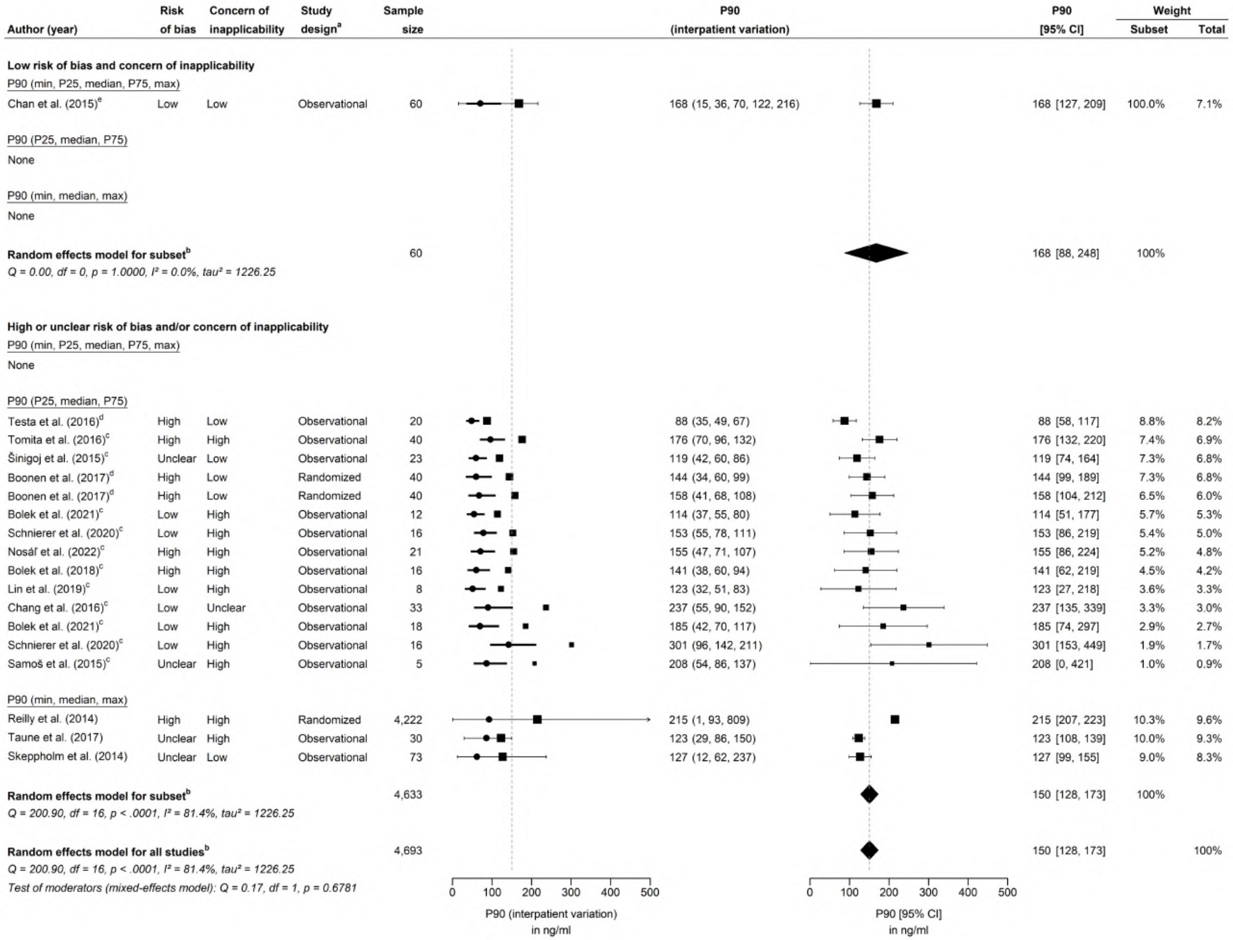

E. Dabigatran 150 mg twice daily

[a] All analyses of interest were cross-sectional; [b] Random effects model using the quantile-estimation method;[2-5] [c] Simulated values were used because only the mean and standard deviation were available; [d] Simulated values were used because available parameters could not readily be included in the QE-method; [e] Percentiles were calculated directly from the original dataset if they were published by the authors of the current review.[6-8]

F. Edoxaban 15 mg once daily

Performing a sensitivity analysis was not possible because only two studies were available, and both were not at low risk of bias and low concern of inapplicability.

G. Edoxaban 30 mg once daily

We did not perform a sensitivity analysis because fewer than ten studies were available (see **Supporting Information File 2**).[1] One study was at low risk of bias and low concern of inapplicability and three received other classifications.

H. Edoxaban 60 mg once daily

We did not perform a sensitivity analysis because fewer than ten studies were available (see **Supporting Information File 2**).[1] One study was at low risk of bias and low concern of inapplicability and two received other classifications.

I. Rivaroxaban 10 mg once daily

Performing a sensitivity analysis was not possible because only four studies were available, and all were not at low risk of bias and low concern of inapplicability.

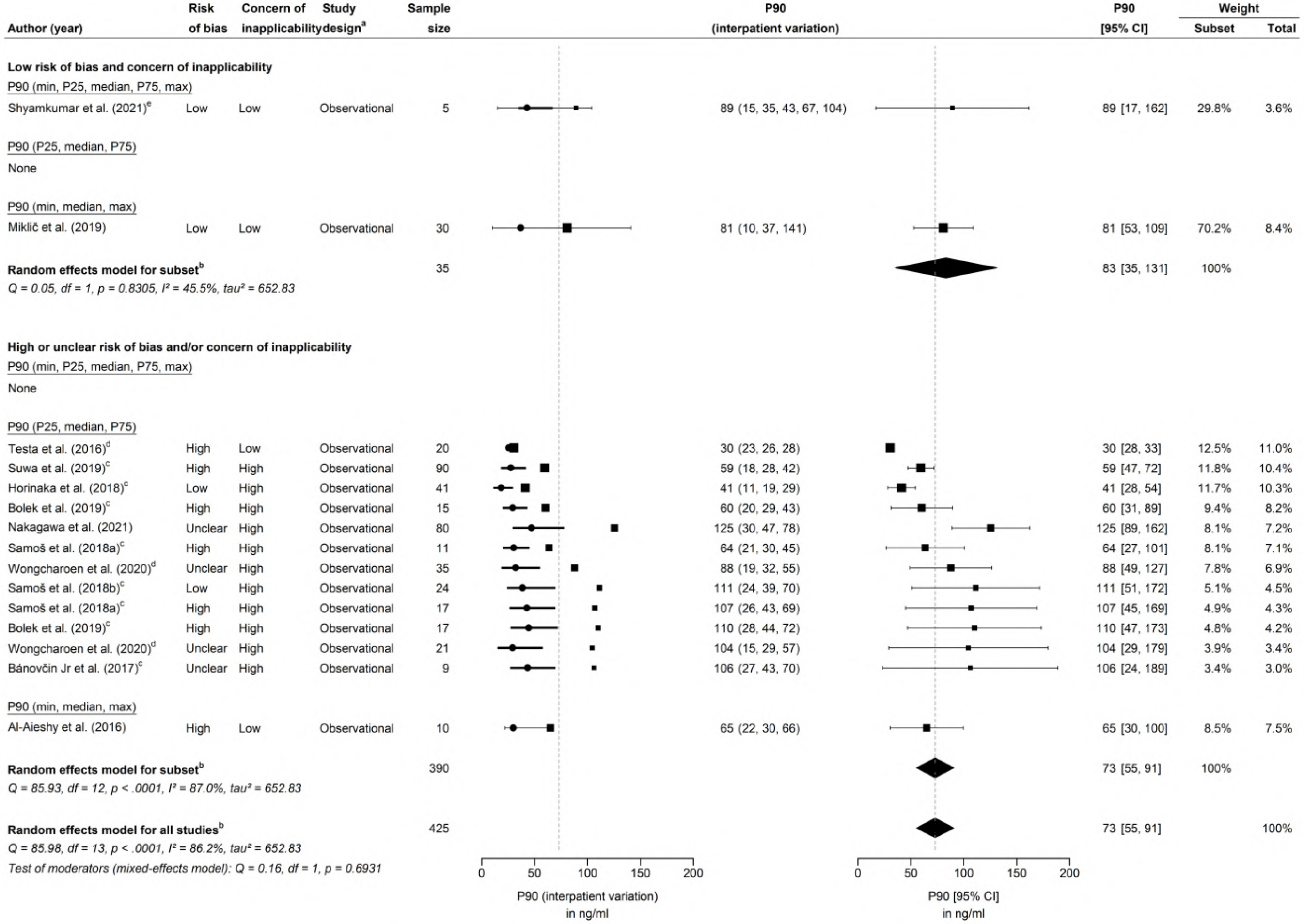

J. Rivaroxaban 15 mg once daily

[a] All analyses of interest were cross-sectional; [b] Random effects model using the quantile-estimation method;[2-5] [c] Simulated values were used because only the mean and standard deviation were available; [d] Simulated values were used because available parameters could not readily be included in the QE-method; [e] Percentiles were calculated directly from the original dataset if they were published by the authors of the current review.[6-8]

K. Rivaroxaban 20 mg once daily

We did not perform a sensitivity analysis because fewer than ten studies were available (see **Supporting Information File 2**).[1] Two studies were at low risk of bias and low concern of inapplicability and six received other classifications.

Figure S3. Sensitivity analysis 1 (risk of bias and concern of inapplicability): Estimating the pooled 10th percentile of peak levels of each direct oral anticoagulant stratified by administered dose using the modified QE-method

The forest plots below illustrate the results of our analyses to estimate the 10th percentile of peak levels of each DOAC type, stratified by dosing regimen and by risk of bias and concern of inapplicability classification (i.e., low risk of bias and low concern of inapplicability vs. other classifications). The squares represent the 10th percentile values, the circles the median values, the solid bold lines the 25th to 75th percentile range, and the whiskers either the minimum to maximum value interval (left side of the plot) or the 95% of the confidence interval of percentile value of interest (right side of the plot).

A. Apixaban 2.5 mg twice daily

Performing a sensitivity analysis was not possible because none of the 12 studies were at both low risk of bias and low concern of inapplicability.

B. Apixaban 5 mg twice daily

Performing a sensitivity analysis was not possible because none of the 16 studies were at both low risk of bias and low concern of inapplicability.

C. Dabigatran 75 mg twice daily

Performing a sensitivity analysis was not possible because only a single study was available.

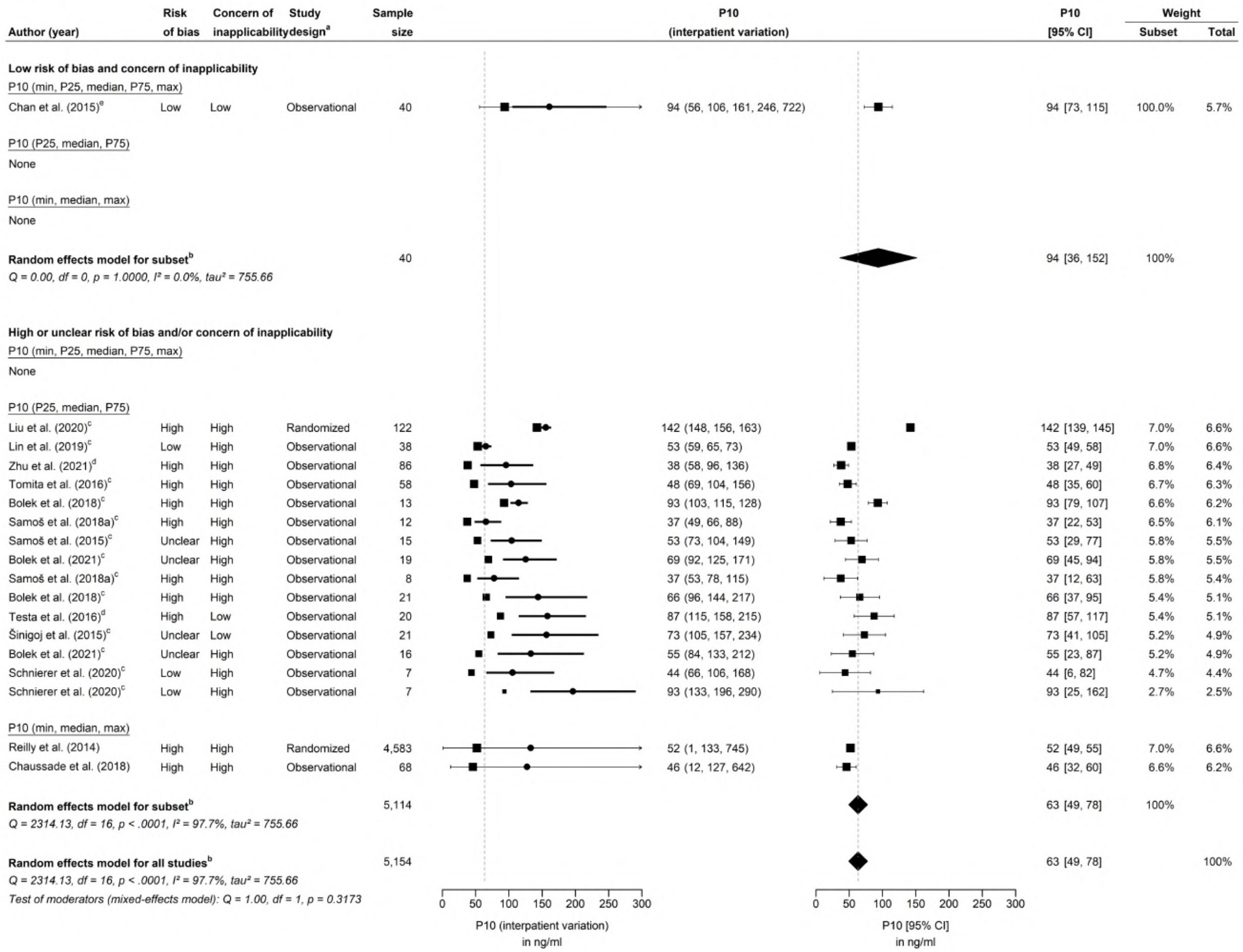

D. Dabigatran 110 mg twice daily

[a] All analyses of interest were cross-sectional; [b] Random effects model using the quantile-estimation method;[2-5] [c] Simulated values were used because only the mean and standard deviation were available; [d] Simulated values were used because available parameters could not readily be included in the QE-method; [e] Percentiles were calculated directly from the original dataset if they were published by the authors of the current review.[6-8]

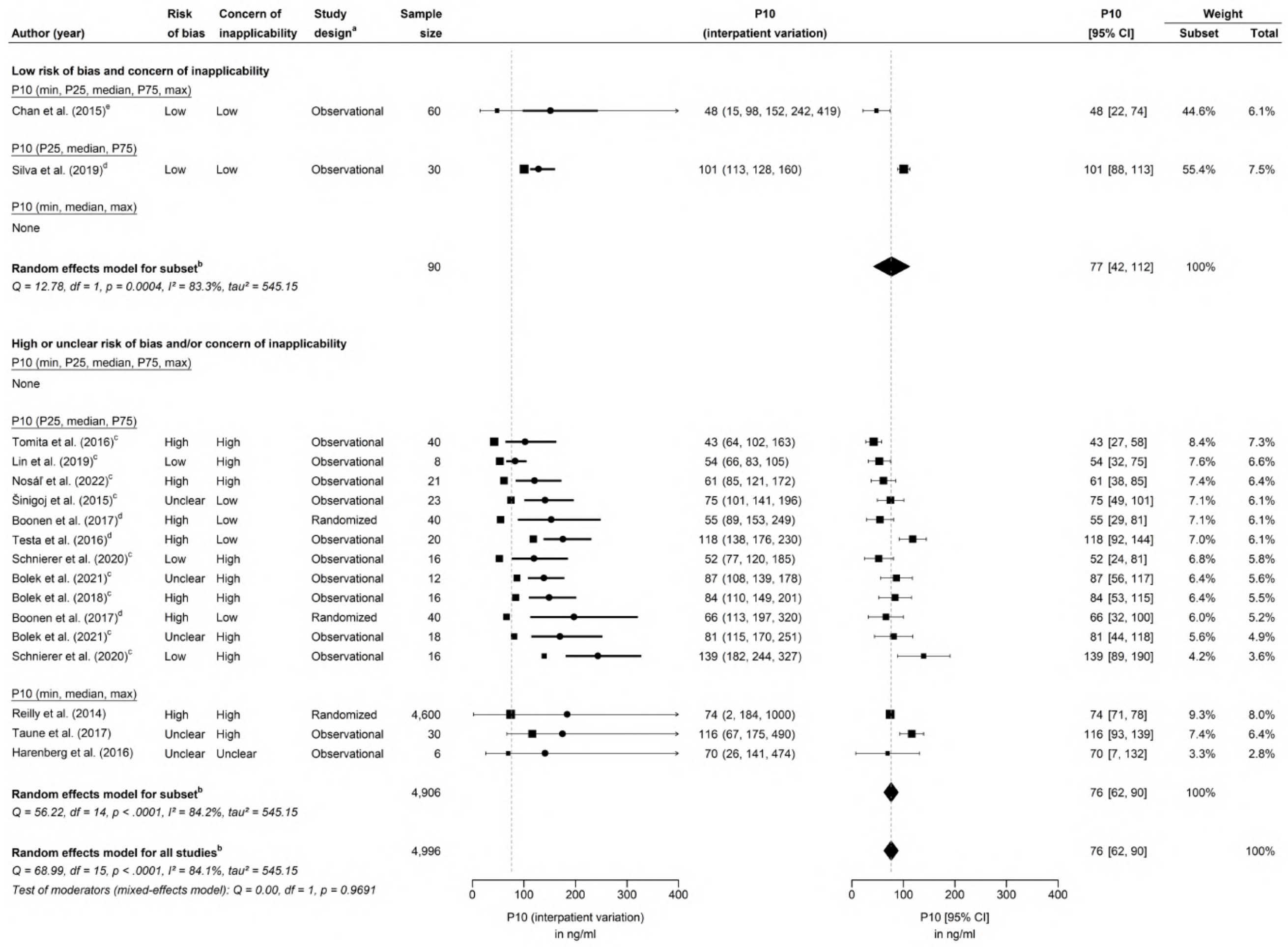

E. Dabigatran 150 mg twice daily

[a] All analyses of interest were cross-sectional; [b] Random effects model using the quantile-estimation method;[2-5] [c] Simulated values were used because only the mean and standard deviation were available; [d] Simulated values were used because available parameters could not readily be included in the QE-method; [e] Percentiles were calculated directly from the original dataset if they were published by the authors of the current review.[6-8]

F. Edoxaban 15 mg once daily

Performing a sensitivity analysis was not possible because only a single study was available.

G. Edoxaban 30 mg once daily

We did not perform a sensitivity analysis because fewer than ten studies were available (see **Supporting Information File 2**).[1] One study was at low risk of bias and low concern of inapplicability and one other received other classifications.

H. Edoxaban 60 mg once daily

We did not perform a sensitivity analysis because fewer than ten studies were available (see **Supporting Information File 2**).[1] One study was at low risk of bias and low concern of inapplicability and one other received other classifications.

I. Rivaroxaban 10 mg once daily

Performing a sensitivity analysis was not possible because none of the four studies were at both low risk of bias and low concern of inapplicability.

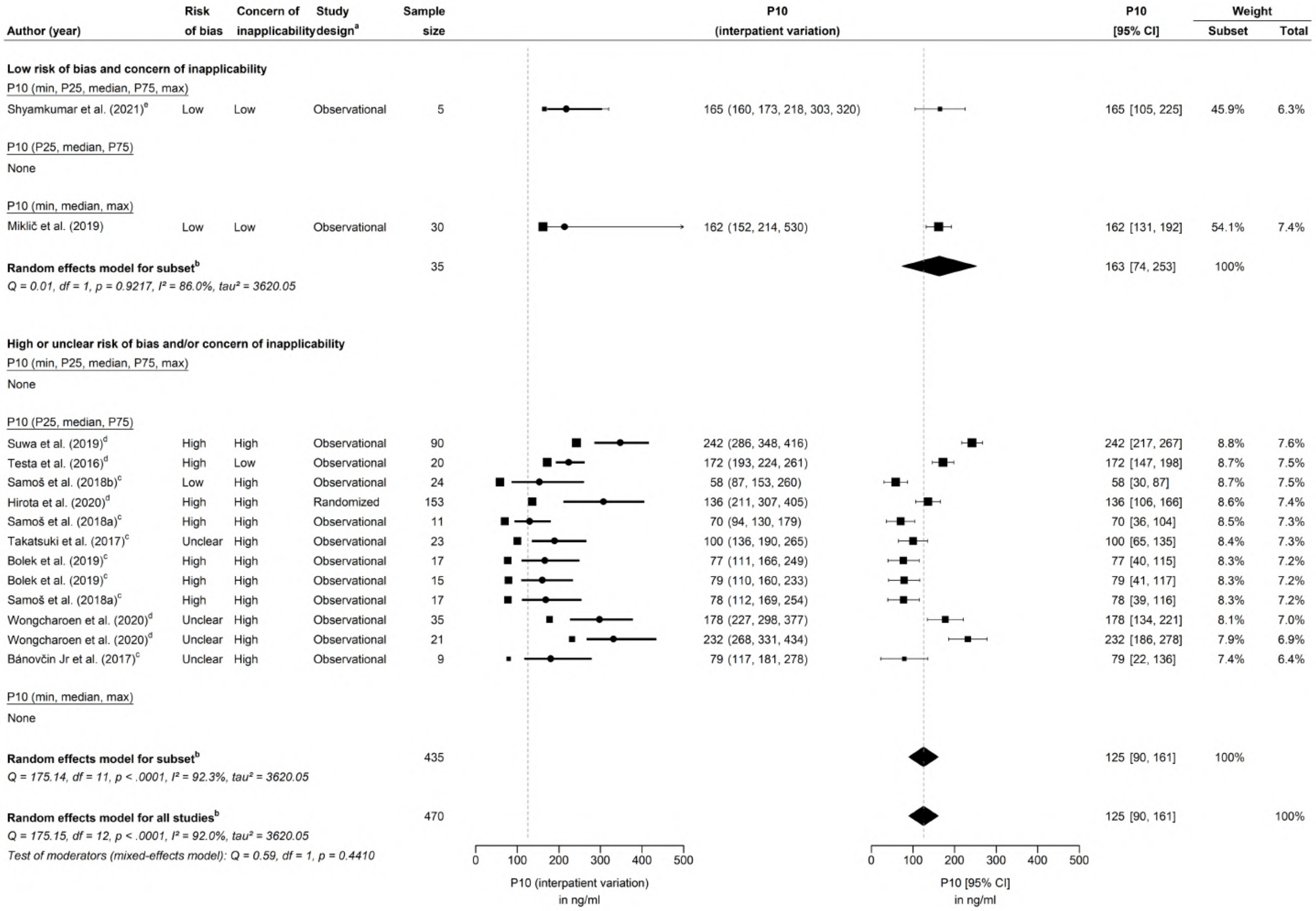

J. Rivaroxaban 15 mg once daily

[a] All analyses of interest were cross-sectional; [b] Random effects model using the quantile-estimation method;[2-5] [c] Simulated values were used because only the mean and standard deviation were available; [d] Simulated values were used because available parameters could not readily be included in the QE-method; [e] Percentiles were calculated directly from the original dataset if they were published by the authors of the current review.[6-8]

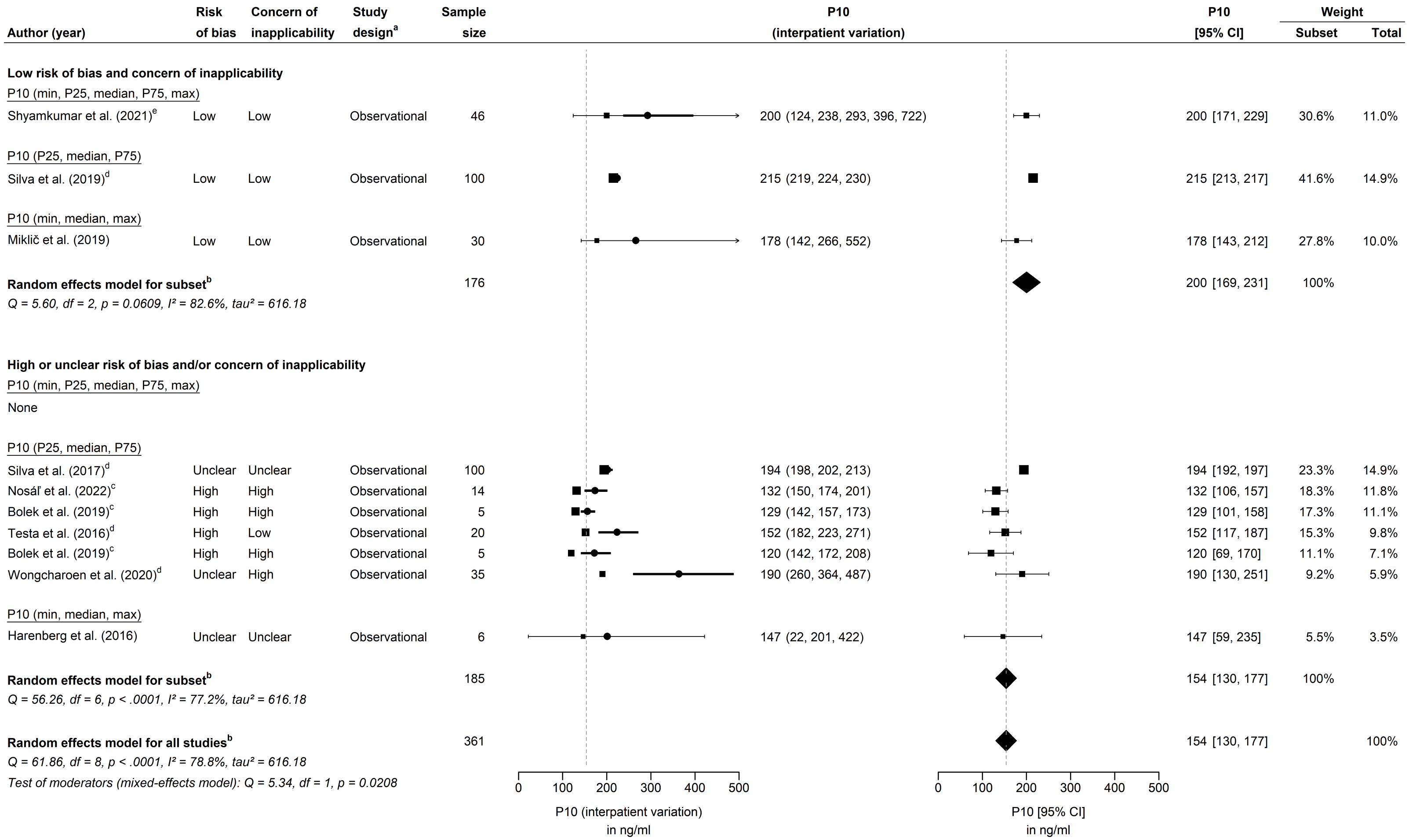

K. Rivaroxaban 20 mg once daily

[a] All analyses of interest were cross-sectional; [b] Random effects model using the quantile-estimation method;[2-5] [c] Simulated values were used because only the mean and standard deviation were available; [d] Simulated values were used because available parameters could not readily be included in the QE-method; [e] Percentiles were calculated directly from the original dataset if they were published by the authors of the current review.[6-8]

Figure S4. Sensitivity analysis 1 (risk of bias and concern of inapplicability): Estimating the pooled 90th percentile of peak levels of each direct oral anticoagulant stratified by administered dose using the modified QE-method

The forest plots below illustrate the results of our analyses to estimate the 90th percentile of peak levels of each DOAC type, stratified by dosing regimen and by risk of bias and concern of inapplicability classification (i.e., low risk of bias and low concern of inapplicability vs. other classifications). The squares represent the 90th percentile values, the circles the median values, the solid bold lines the 25th to 75th percentile range, and the whiskers either the minimum to maximum value interval (left side of the plot) or the 95% of the confidence interval of the percentile value of interest (right side of the plot).

A. Apixaban 2.5 mg twice daily

Performing a sensitivity analysis was not possible because none of the 12 studies were at both low risk of bias and low concern of inapplicability.

B. Apixaban 5 mg twice daily

Performing a sensitivity analysis was not possible because none of the 16 studies were at both low risk of bias and low concern of inapplicability.

C. Dabigatran 75 mg twice daily

Performing a sensitivity analysis was not possible because only a single study was available.

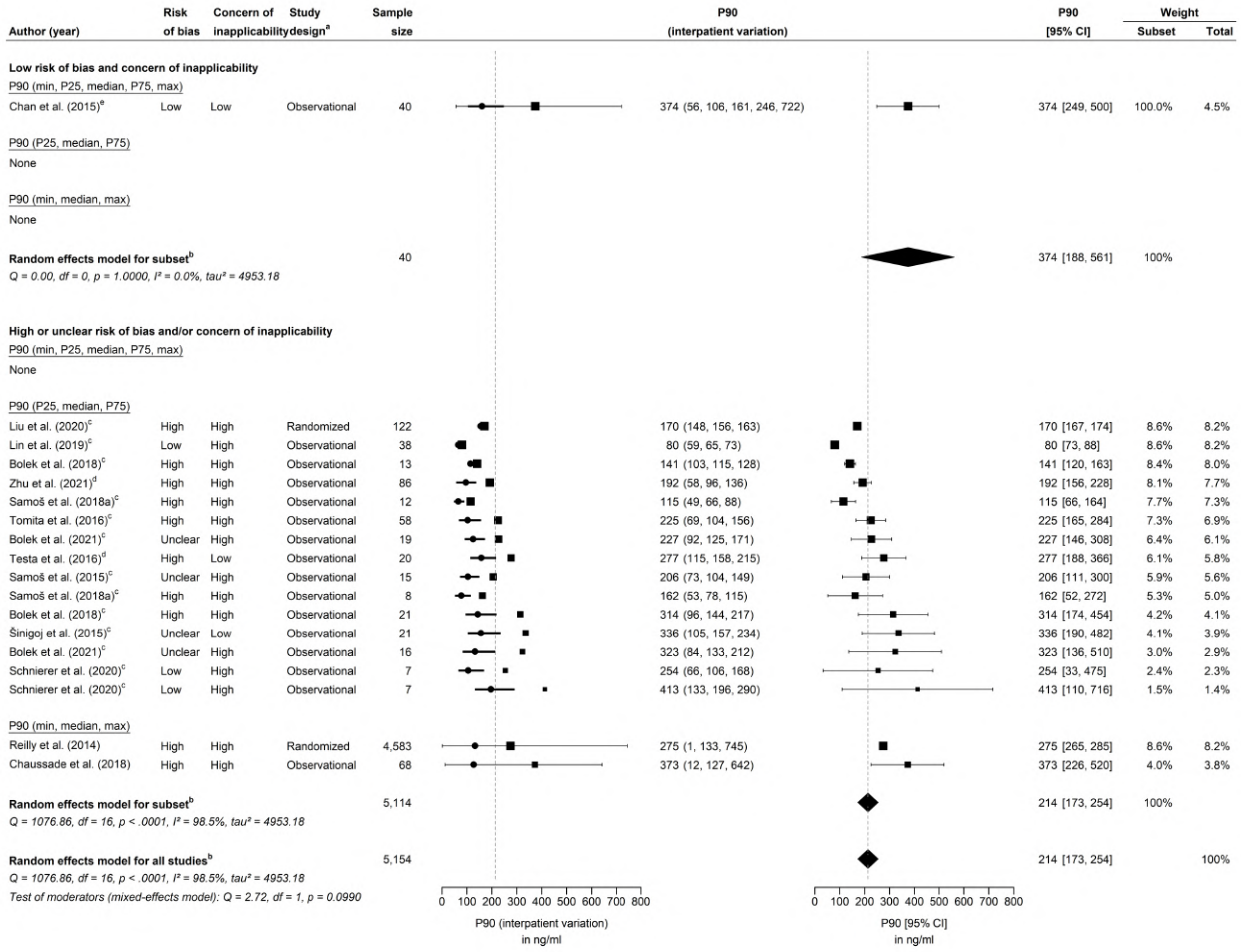

D. Dabigatran 110 mg twice daily

[a] All analyses of interest were cross-sectional; [b] Random effects model using the quantile-estimation method;[2-5] [c] Simulated values were used because only the mean and standard deviation were available; [d] Simulated values were used because available parameters could not readily be included in the QE-method; [e] Percentiles were calculated directly from the original dataset if they were published by the authors of the current review.[6-8]

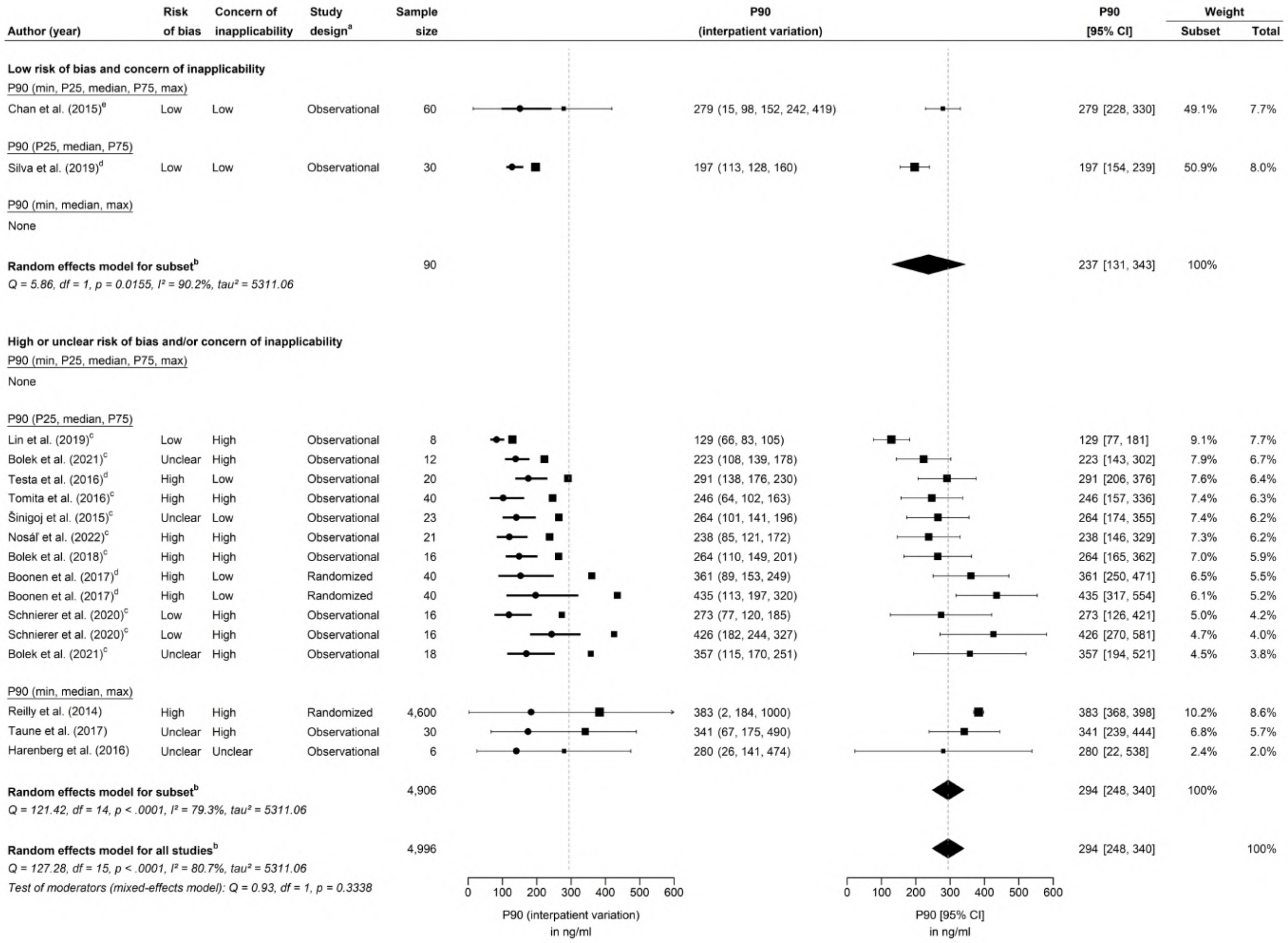

E. Dabigatran 150 mg twice daily

[a] All analyses of interest were cross-sectional; [b] Random effects model using the quantile-estimation method;[2-5] [c] Simulated values were used because only the mean and standard deviation were available; [d] Simulated values were used because available parameters could not readily be included in the QE-method; [e] Percentiles were calculated directly from the original dataset if they were published by the authors of the current review.[6-8]

F. Edoxaban 15 mg once daily

Performing a sensitivity analysis was not possible because only a single study was available.

G. Edoxaban 30 mg once daily

We did not perform a sensitivity analysis because fewer than ten studies were available (see **Supporting Information File 2**).[1] One study was at low risk of bias and low concern of inapplicability and one other received other classifications.

H. Edoxaban 60 mg once daily

We did not perform a sensitivity analysis because fewer than ten studies were available (see **Supporting Information File 2**).[1] One study was at low risk of bias and low concern of inapplicability and one other received other classifications.

I. Rivaroxaban 10 mg once daily

Performing a sensitivity analysis was not possible because none of the four studies were at both low risk of bias and low concern of inapplicability.

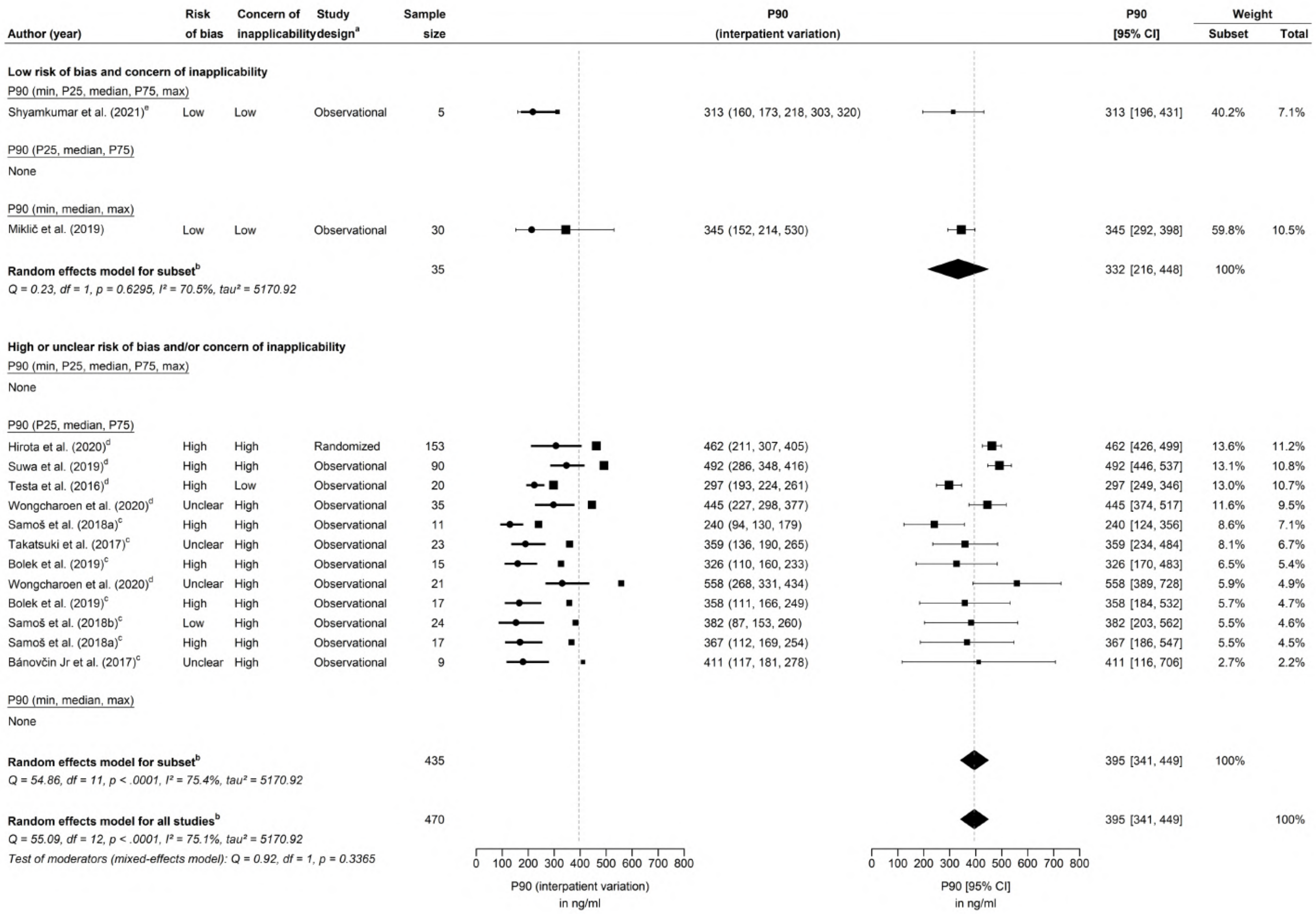

J. Rivaroxaban 15 mg once daily

[a] All analyses of interest were cross-sectional; [b] Random effects model using the quantile-estimation method;[2-5] [c] Simulated values were used because only the mean and standard deviation were available; [d] Simulated values were used because available parameters could not readily be included in the QE-method; [e] Percentiles were calculated directly from the original dataset if they were published by the authors of the current review.[6-8]

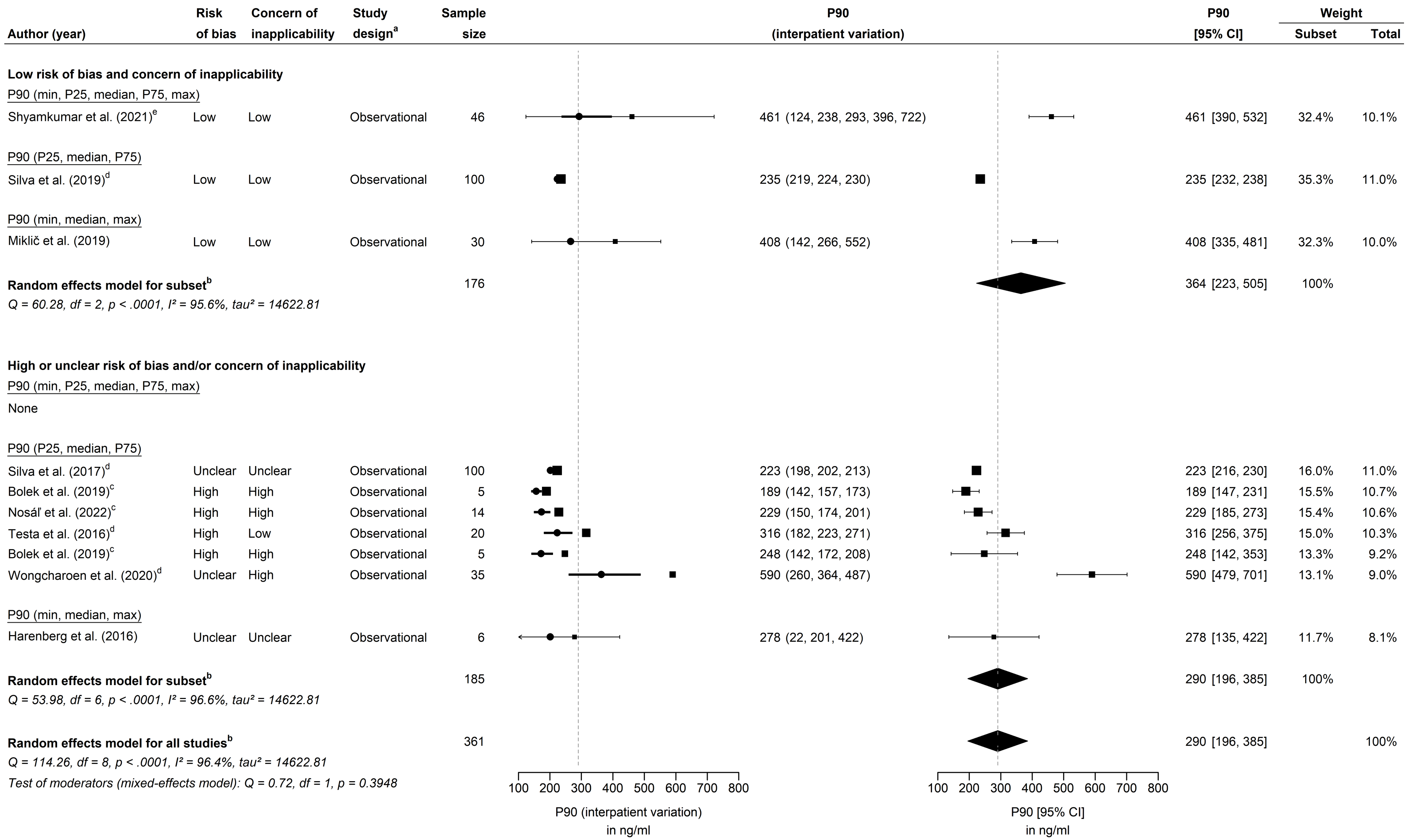

K. Rivaroxaban 20 mg once daily

[a] All analyses of interest were cross-sectional; [b] Random effects model using the quantile-estimation method;[2-5] [c] Simulated values were used because only the mean and standard deviation were available; [d] Simulated values were used because available parameters could not readily be included in the QE-method; [e] Percentiles were calculated directly from the original dataset if they were published by the authors of the current review.[6-8]

Sensitivity analysis 2: Data extraction method

Table S2. Summary of sensitivity analysis 2: Data extraction method

	Pooled 10th percentile [95% CI]				Pooled 90th percentile [95% CI]			
DOAC dosing regimen	Reported[a]	Simulated[a]	Total	p-value[b]	Reported[a]	Simulated[a]	Total	p-value[b]
**Trough**								
Apixaban								
2.5 mg twice daily	31 [20, 43]	42 [33, 50]	42 [33, 50]	0.1621	158 [100, 216]	142 [126, 158]	156 [120, 191]	0.9873
5 mg twice daily	45 [22, 67]	62 [49, 75]	62 [49, 75]	0.1998	228 [155, 302]	193 [166, 220]	197 [161, 234]	0.3824
Dabigatran								
75 mg twice daily	NA	NA	NA	NA	NA	NA	NA	NA
110 mg twice daily	28 [21, 36]	39 [23, 44]	39 [23, 44]	0.0705	169 [124, 213]	126 [107, 146]	128 [106, 150]	0.1054
150 mg twice daily	31 [22, 40]	34 [27, 40]	34 [27, 40]	0.6388	173 [130, 216]	138 [117, 158]	140 [117, 164]	0.1128
Edoxaban								
15 mg once daily	NA	NA	NA	NA	NA	NA	NA	NA
30 mg once daily	NA	NA	NA	NA	NA	NA	NA	NA
60 mg once daily	NA	NA	NA	NA	NA	NA	NA	NA
Rivaroxaban								
10 mg once daily	NA	NA	NA	NA	NA	NA	NA	NA
15 mg once daily	22 [13, 32]	15 [12, 18]	15 [12, 18]	0.1666	73 [22, 123]	75 [57, 92]	75 [57, 92]	0.9523
20 mg once daily	NA	NA	NA	NA	NA	NA	NA	NA
**Peak**								
Apixaban								
2.5 mg twice daily	76 [40, 111]	99 [87, 111]	99 [87, 111]	0.2211	228 [157, 299]	253 [233, 274]	253 [233, 274]	0.4974
5 mg twice daily	138 [47, 230]	132 [107, 156]	132 [107, 156]	0.8868	358 [264, 452]	342 [310, 375]	342 [310, 375]	0.7559
Dabigatran								

	Pooled 10th percentile [95% CI]				Pooled 90th percentile [95% CI]			
**DOAC dosing regimen**	**Reported[a]**	**Simulated[a]**	**Total**	***p*-value[b]**	**Reported[a]**	**Simulated[a]**	**Total**	***p*-value[b]**
75 mg twice daily	NA	NA	NA	NA	NA	NA	NA	NA
110 mg twice daily	57 [28, 85]	68 [52, 84]	68 [52, 84]	0.4933	282 [203, 361]	200 [156, 245]	200 [156, 245]	0.0788
150 mg twice daily	63 [29, 97]	79 [65, 92]	79 [65, 92]	0.4026	334 [233, 435]	275 [230, 320]	275 [230, 320]	0.2938
Edoxaban								
15 mg once daily	NA	NA	NA	NA	NA	NA	NA	NA
30 mg once daily	NA	NA	NA	NA	NA	NA	NA	NA
60 mg once daily	NA	NA	NA	NA	NA	NA	NA	NA
Rivaroxaban								
10 mg once daily	NA	NA	NA	NA	NA	NA	NA	NA
15 mg once daily	165 [32, 299]	128 [94, 163]	128 [94, 163]	0.6000	313 [128, 498]	389 [338, 440]	389 [338, 440]	0.4378
20 mg once daily	200 [130, 270]	165 [140, 190]	165 [140, 190]	0.3562	461 [229. 693]	296 [218, 374]	296 [218, 374]	0.1861

This table summarizes the results of the sensitivity analyses on the method of data extraction (i.e., reported vs. simulated), to assess the robustness of our findings on the 10th and 90th percentiles. We defined a significant difference as a *p*-value of <0.05 of the test of moderators (**bold** in table), though we acknowledge this has major limitations. We urge readers to appraise the individual forest plots (**Fig S5 through S8**).

NA not applicable.

[a] We kept Tau constant if there were five or fewer studies in either category;[1] [b] P-value of the test of moderators (two-tailed Wald-test).

Figure S5. Sensitivity analysis 2 (by data extraction method): Estimating the pooled 10th percentile of trough levels of each direct oral anticoagulant stratified by administered dose using the modified QE-method

The forest plots below illustrate the results of our analyses to estimate the 10th percentile of trough levels of each DOAC type, stratified by dosing regimen and by method of data extraction (reported vs. simulated). The squares represent the 10th percentile values, the circles the median values, the solid bold lines the 25th to 75th percentile range, and the whiskers either the minimum to maximum value interval (left side of the plot) or the 95% of the confidence interval of the percentile value of interest (right side of the plot).

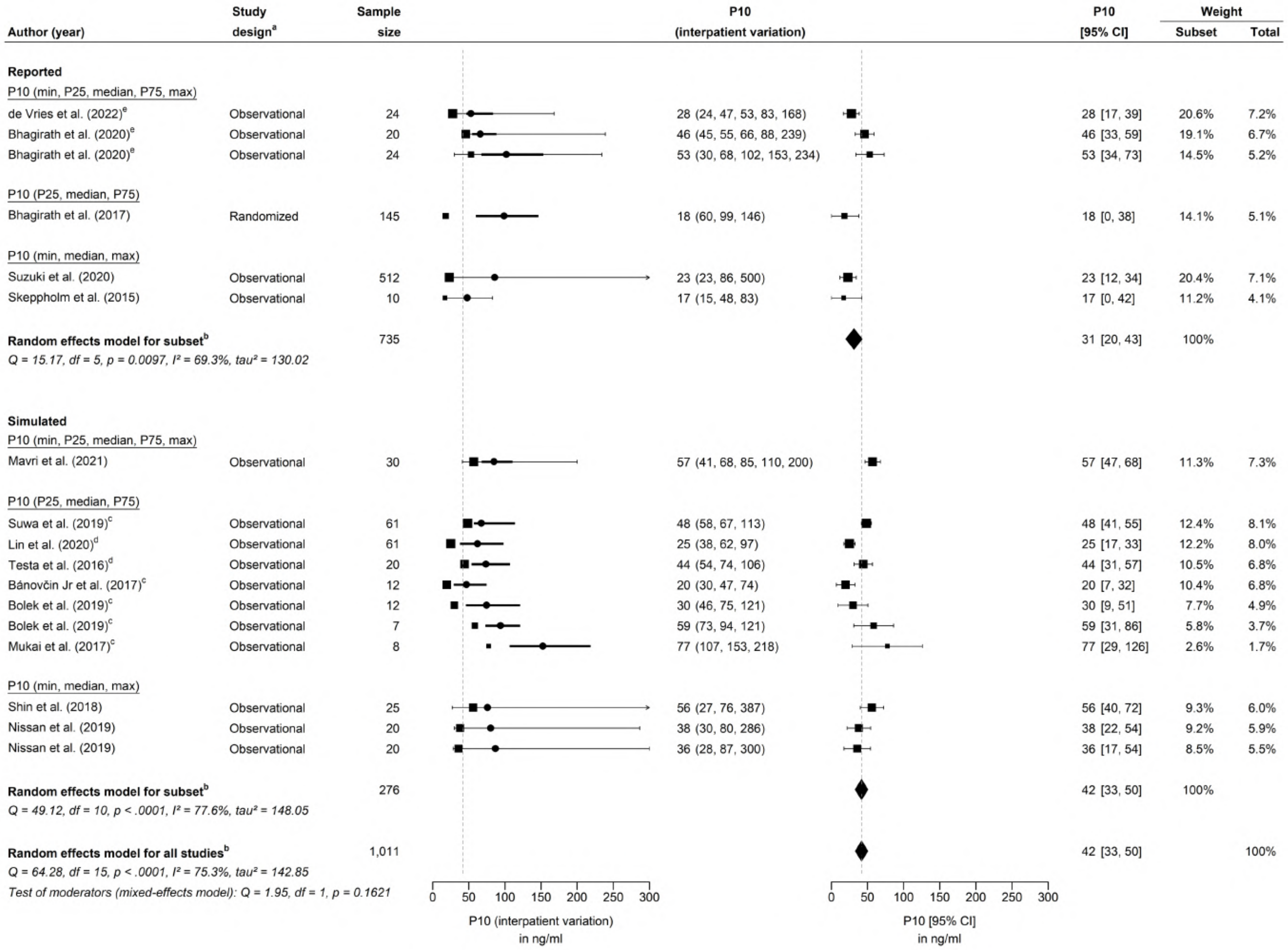

A. Apixaban 2.5 mg twice daily

[a] All analyses of interest were cross-sectional; [b] Random effects model using the quantile-estimation method;[2-5] [c] Simulated values were used because only the mean and standard deviation were available; [d] Simulated values were used because available parameters could not readily be included in the QE-method; [e] Percentiles were calculated directly from the original dataset if they were published by the authors of the current review.[6-8]

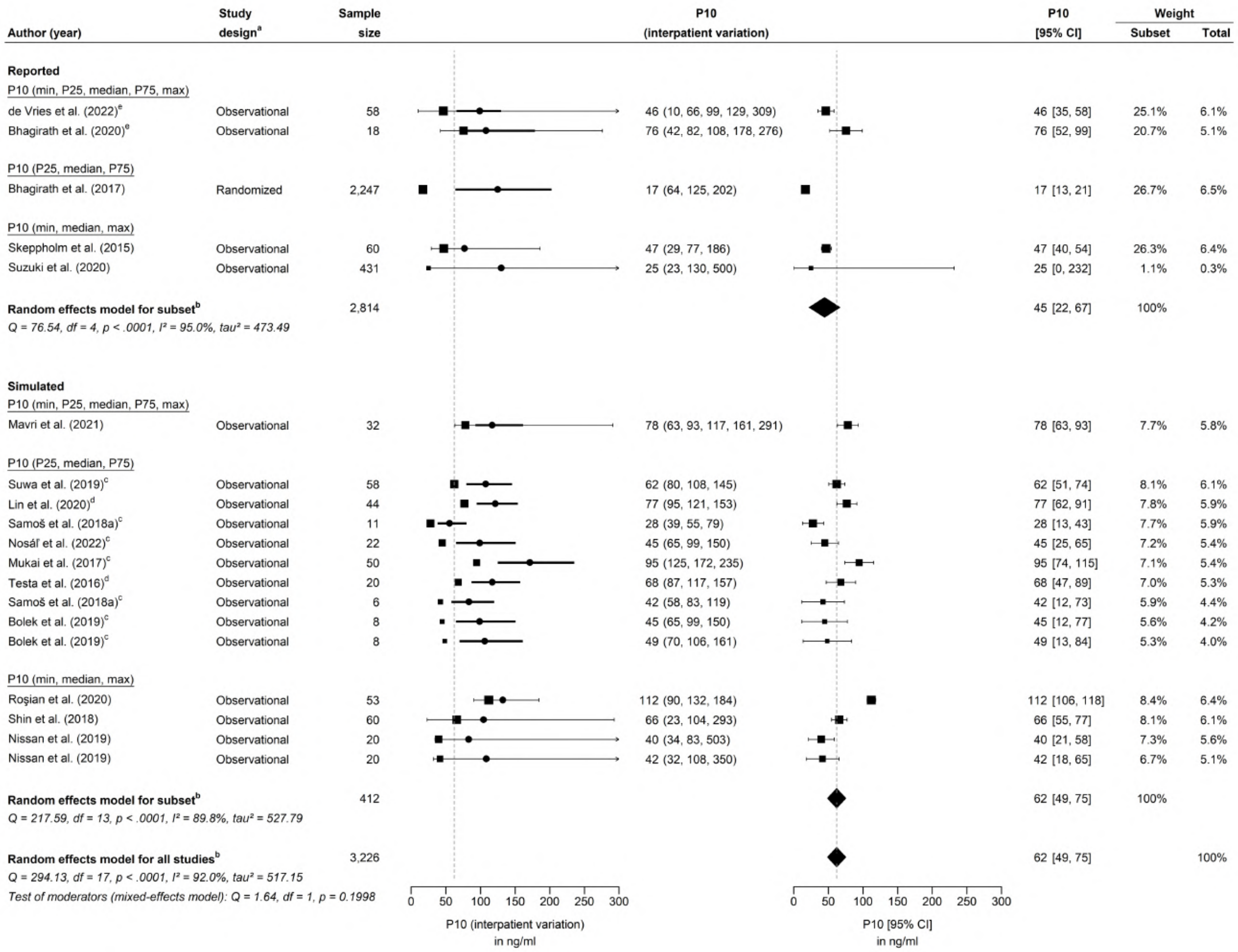

B. Apixaban 5 mg twice daily

[a] All analyses of interest were cross-sectional; [b] Random effects model using the quantile-estimation method;[2-5] [c] Simulated values were used because only the mean and standard deviation were available; [d] Simulated values were used because available parameters could not readily be included in the QE-method; [e] Percentiles were calculated directly from the original dataset if they were published by the authors of the current review.[6-8]

C. Dabigatran 75 mg twice daily

Performing a sensitivity analysis was not possible because only a single study was available.

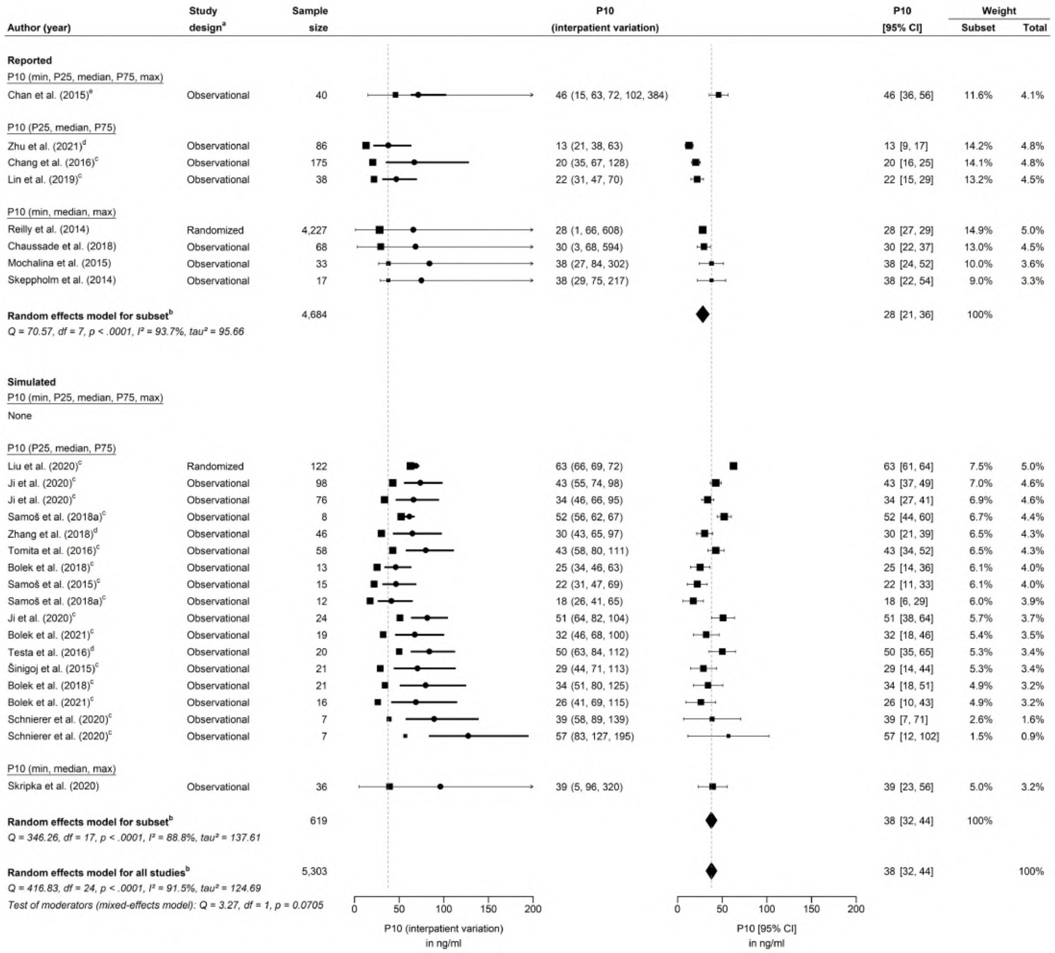

D. Dabigatran 110 mg twice daily

[a] All analyses of interest were cross-sectional; [b] Random effects model using the quantile-estimation method;[2-5] [c] Simulated values were used because only the mean and standard deviation were available; [d] Simulated values were used because available parameters could not readily be included in the QE-method; [e] Percentiles were calculated directly from the original dataset if they were published by the authors of the current review.[6-8]

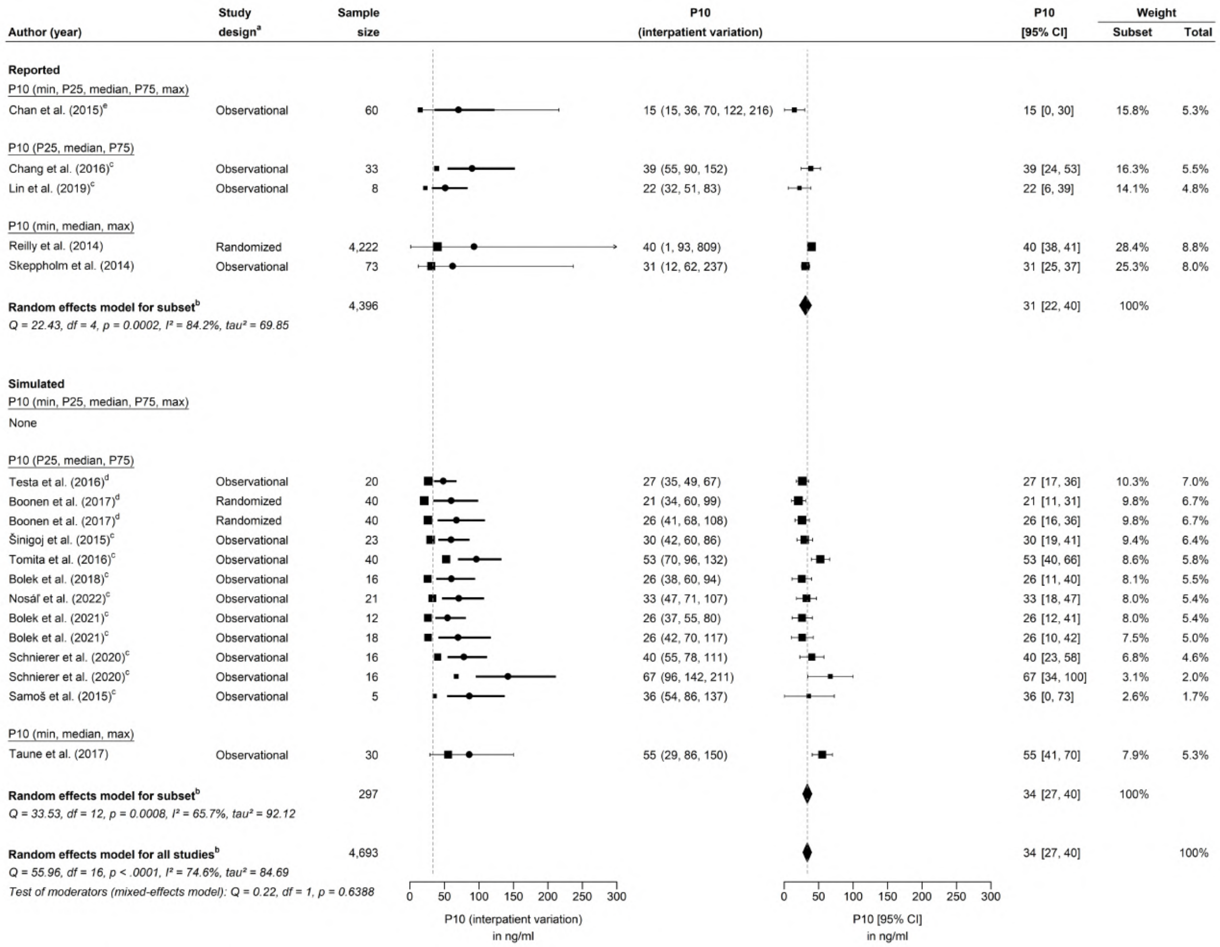

E. Dabigatran 150 mg twice daily

[a] All analyses of interest were cross-sectional; [b] Random effects model using the quantile-estimation method;[2-5] [c] Simulated values were used because only the mean and standard deviation were available; [d] Simulated values were used because available parameters could not readily be included in the QE-method; [e] Percentiles were calculated directly from the original dataset if they were published by the authors of the current review.[6-8]

F. Edoxaban 15 mg once daily

Performing a sensitivity analysis was not possible because none of the two studies reported the 10th percentile.

G. Edoxaban 30 mg once daily

Performing a sensitivity analysis was not possible because none of the four studies reported the 10th percentile.

H. Edoxaban 60 mg once daily

Performing a sensitivity analysis was not possible because none of the three studies reported the 10th percentile.

I. Rivaroxaban 10 mg once daily

Performing a sensitivity analysis was not possible because none of the four studies reported the 10th percentile.

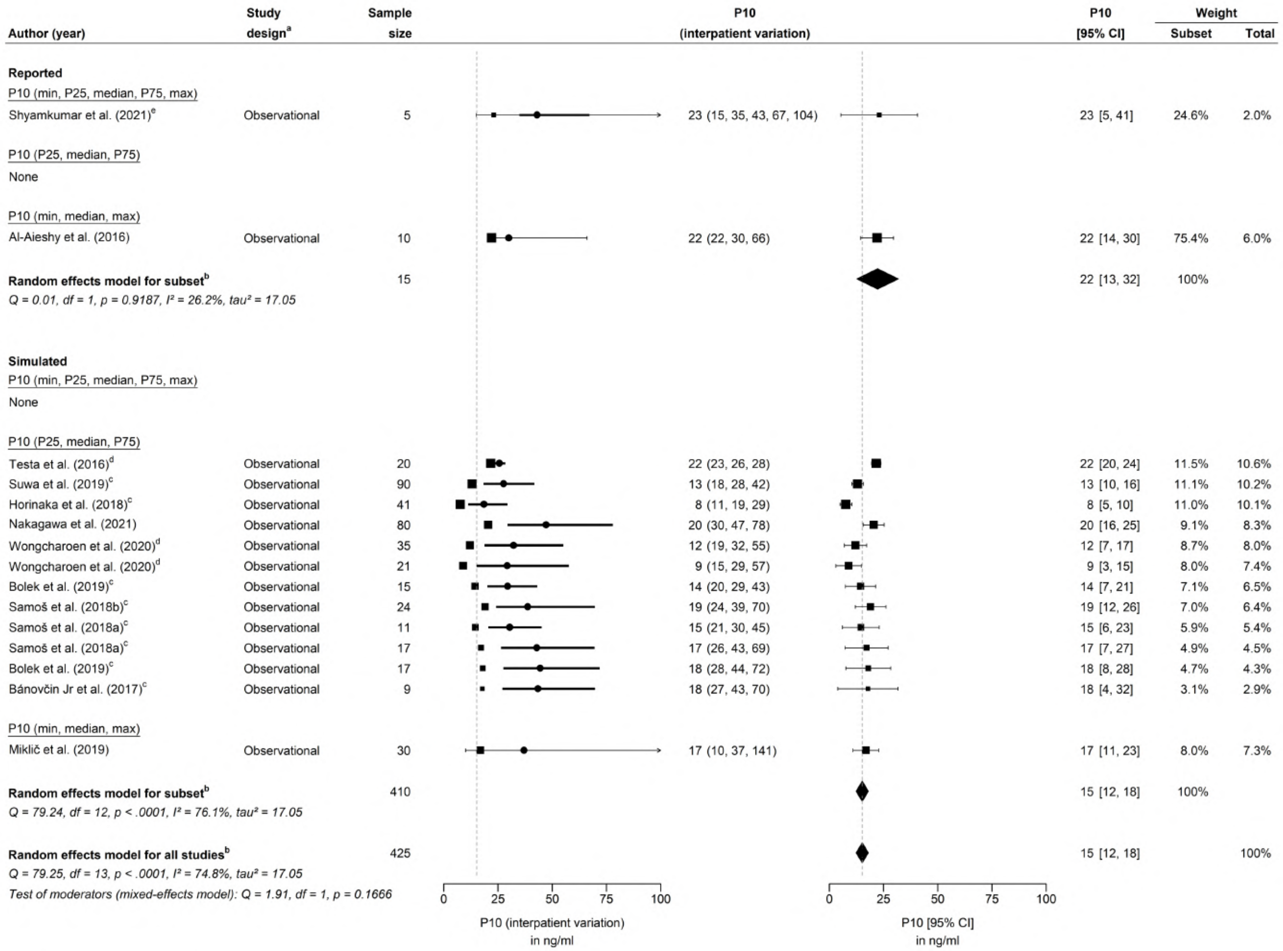

J. Rivaroxaban 15 mg once daily

[a] All analyses of interest were cross-sectional; [b] Random effects model using the quantile-estimation method;[2-5] [c] Simulated values were used because only the mean and standard deviation were available; [d] Simulated values were used because available parameters could not readily be included in the QE-method; [e] Percentiles were calculated directly from the original dataset if they were published by the authors of the current review.[6-8]

K. Rivaroxaban 20 mg once daily

We did not perform a sensitivity analysis because fewer than ten studies were available (see **Supporting Information File 2**).[1] Two studies reported the 10th percentile and we simulated this percentile for the other six studies.

Figure S6. Sensitivity analysis 2 (by data extraction method): Estimating the pooled 90th percentile of trough levels of each direct oral anticoagulant stratified by administered dose using the modified QE-method

The forest plots below illustrate the results of our analyses to estimate the 90th percentile of trough levels of each DOAC type, stratified by dosing regimen and by method of data extraction (reported vs. simulated). The squares represent the 90th percentile values, the circles the median values, the solid bold lines the 25th to 75th percentile range, and the whiskers either the minimum to maximum value interval (left side of the plot) or the 95% of the confidence interval of percentile value of interest (right side of the plot).

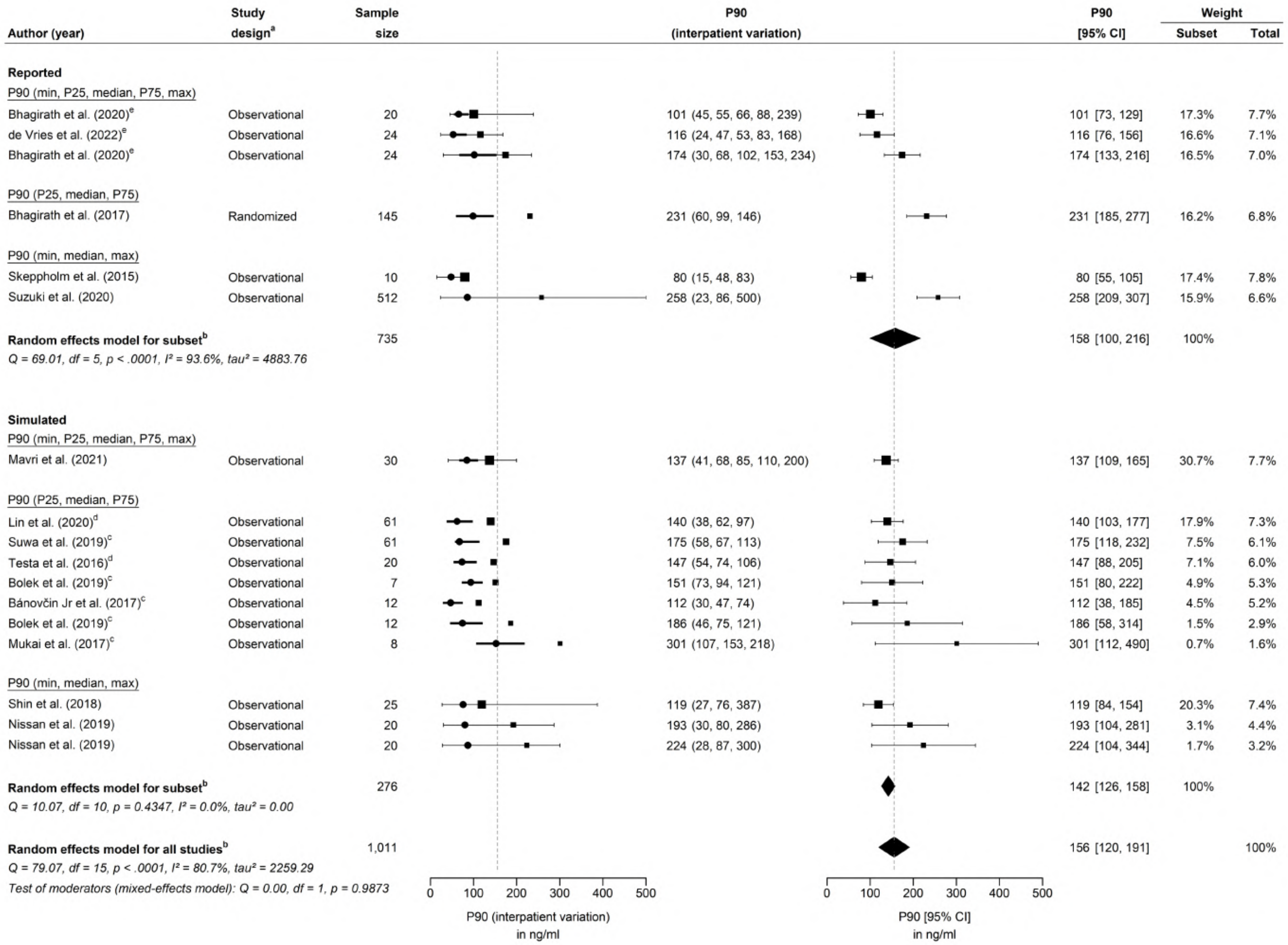

A. Apixaban 2.5 mg twice daily

[a] All analyses of interest were cross-sectional; [b] Random effects model using the quantile-estimation method;[2-5] [c] Simulated values were used because only the mean and standard deviation were available; [d] Simulated values were used because available parameters could not readily be included in the QE-method; [e] Percentiles were calculated directly from the original dataset if they were published by the authors of the current review.[6-8]

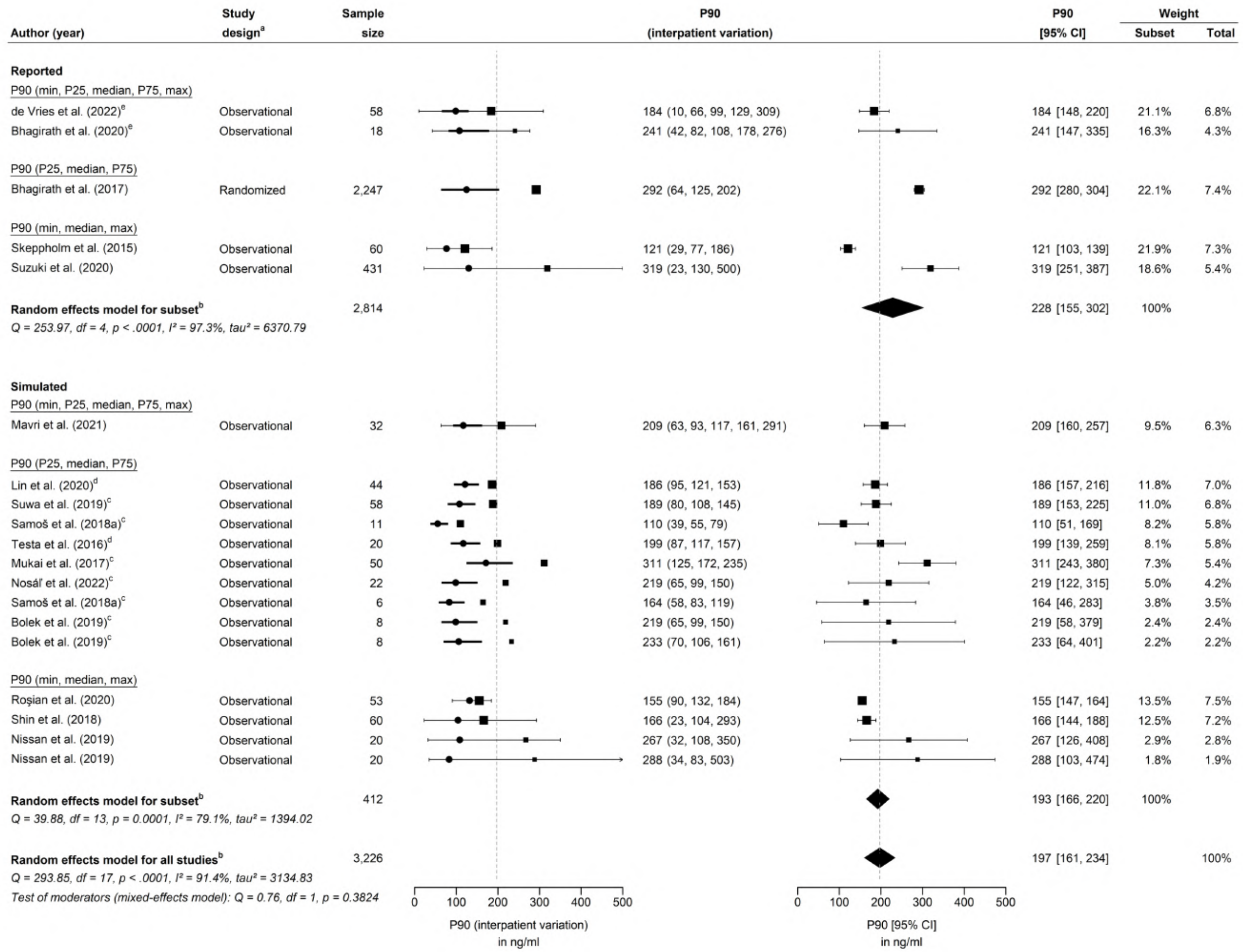

B. Apixaban 5 mg twice daily

[a] All analyses of interest were cross-sectional; [b] Random effects model using the quantile-estimation method;[2-5] [c] Simulated values were used because only the mean and standard deviation were available; [d] Simulated values were used because available parameters could not readily be included in the QE-method; [e] Percentiles were calculated directly from the original dataset if they were published by the authors of the current review.[6-8]

C. Dabigatran 75 mg twice daily

Performing a sensitivity analysis was not possible because only a single study was available.

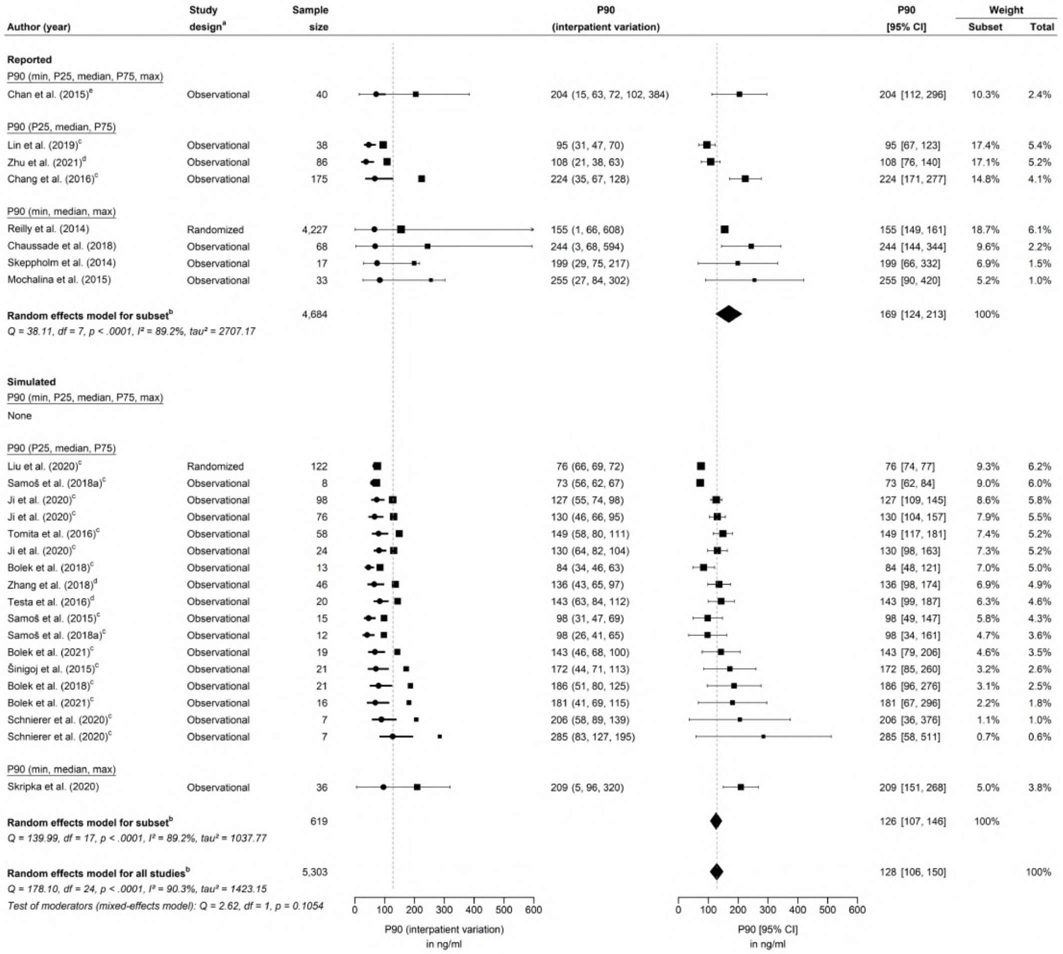

D. Dabigatran 110 mg twice daily

[a] All analyses of interest were cross-sectional; [b] Random effects model using the quantile-estimation method;[2-5] [c] Simulated values were used because only the mean and standard deviation were available; [d] Simulated values were used because available parameters could not readily be included in the QE-method; [e] Percentiles were calculated directly from the original dataset if they were published by the authors of the current review.[6-8]

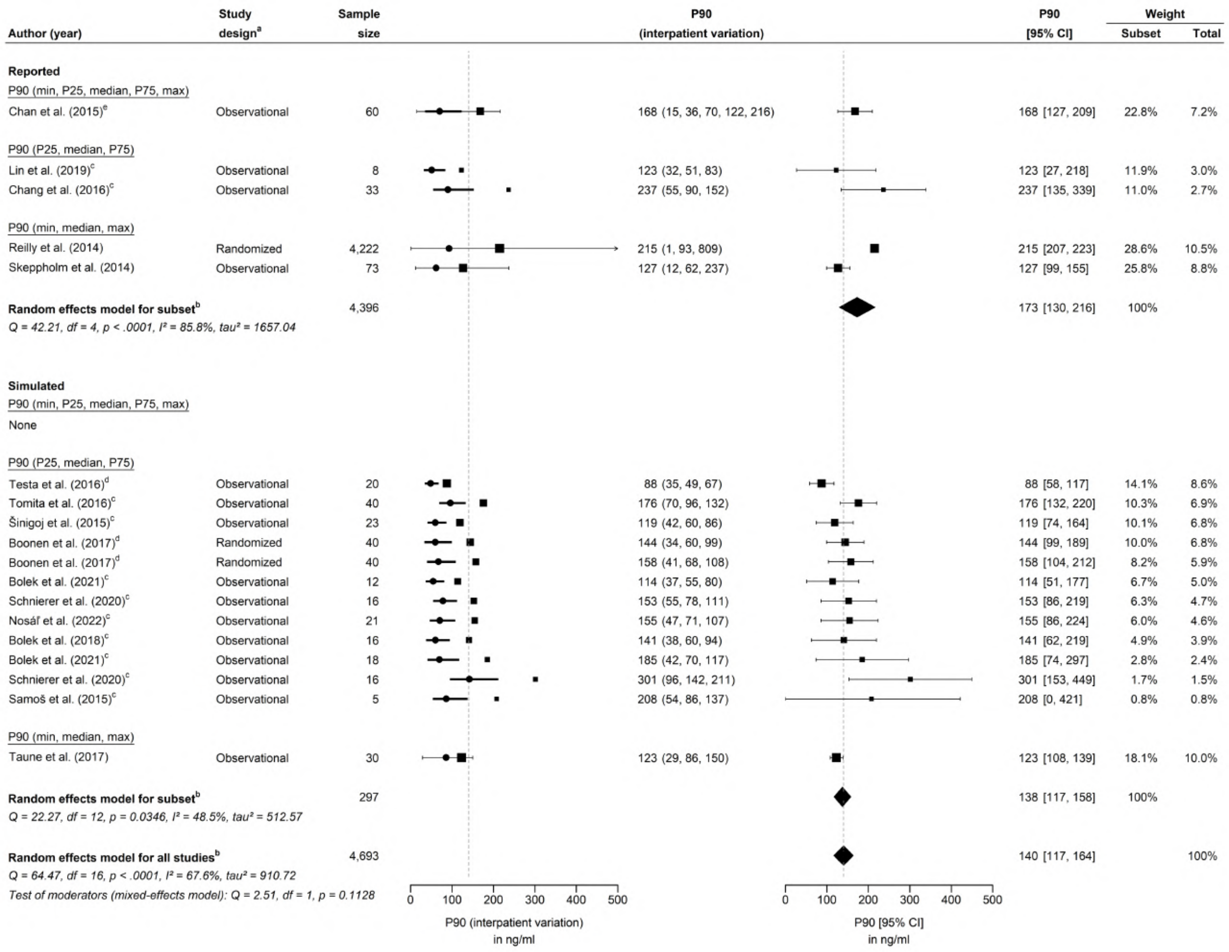

E. Dabigatran 150 mg twice daily

[a] All analyses of interest were cross-sectional; [b] Random effects model using the quantile-estimation method;[2-5] [c] Simulated values were used because only the mean and standard deviation were available; [d] Simulated values were used because available parameters could not readily be included in the QE-method; [e] Percentiles were calculated directly from the original dataset if they were published by the authors of the current review.[6-8]

F. Edoxaban 15 mg once daily

Performing a sensitivity analysis was not possible because none of the two studies reported the 90th percentile.

G. Edoxaban 30 mg once daily

Performing a sensitivity analysis was not possible because none of the four studies reported the 90th percentile.

H. Edoxaban 60 mg once daily

Performing a sensitivity analysis was not possible because none of the three studies reported the 90th percentile.

I. Rivaroxaban 10 mg once daily

Performing a sensitivity analysis was not possible because none of the four studies reported the 90th percentile.

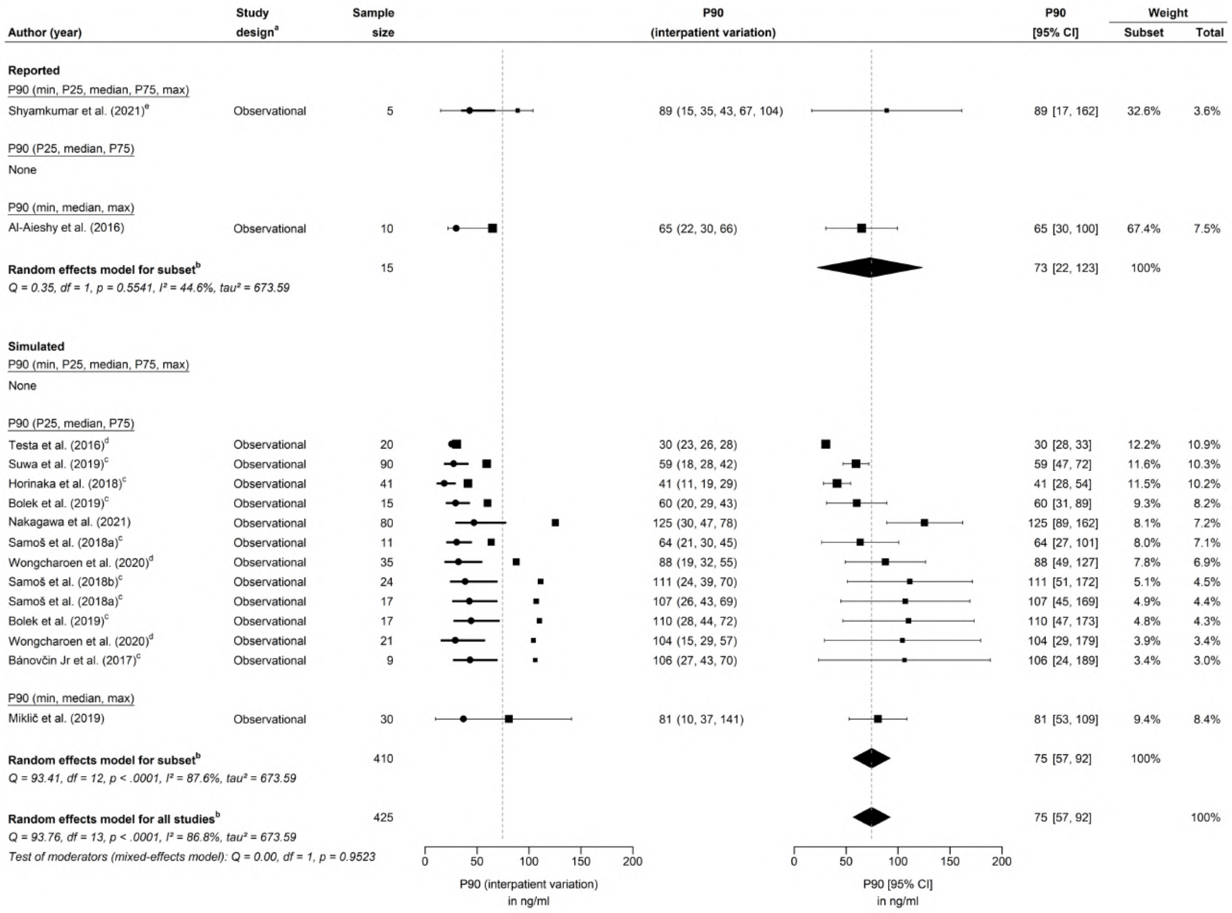

J. Rivaroxaban 15 mg once daily

[a] All analyses of interest were cross-sectional; [b] Random effects model using the quantile-estimation method;[2-5] [c] Simulated values were used because only the mean and standard deviation were available; [d] Simulated values were used because available parameters could not readily be included in the QE-method; [e] Percentiles were calculated directly from the original dataset if they were published by the authors of the current review.[6-8]

K. Rivaroxaban 20 mg once daily

We did not perform a sensitivity analysis because fewer than ten studies were available (see **Supporting Information File 2**).[1] Two studies reported the 90th percentile and we simulated this percentile for the other six studies.

Figure S7. Sensitivity analysis 2 (by data extraction method): Estimating the pooled 10th percentile of peak levels of each direct oral anticoagulant stratified by administered dose using the modified QE-method

The forest plots below illustrate the results of our analyses to estimate the 10th percentile of peak levels of each DOAC type, stratified by dosing regimen and by method of data extraction (reported vs. simulated). The squares represent the 10th percentile values, the circles the median values, the solid bold lines the 25th to 75th percentile range, and the whiskers either the minimum to maximum value interval (left side of the plot) or the 95% of the confidence interval of percentile value of interest (right side of the plot).

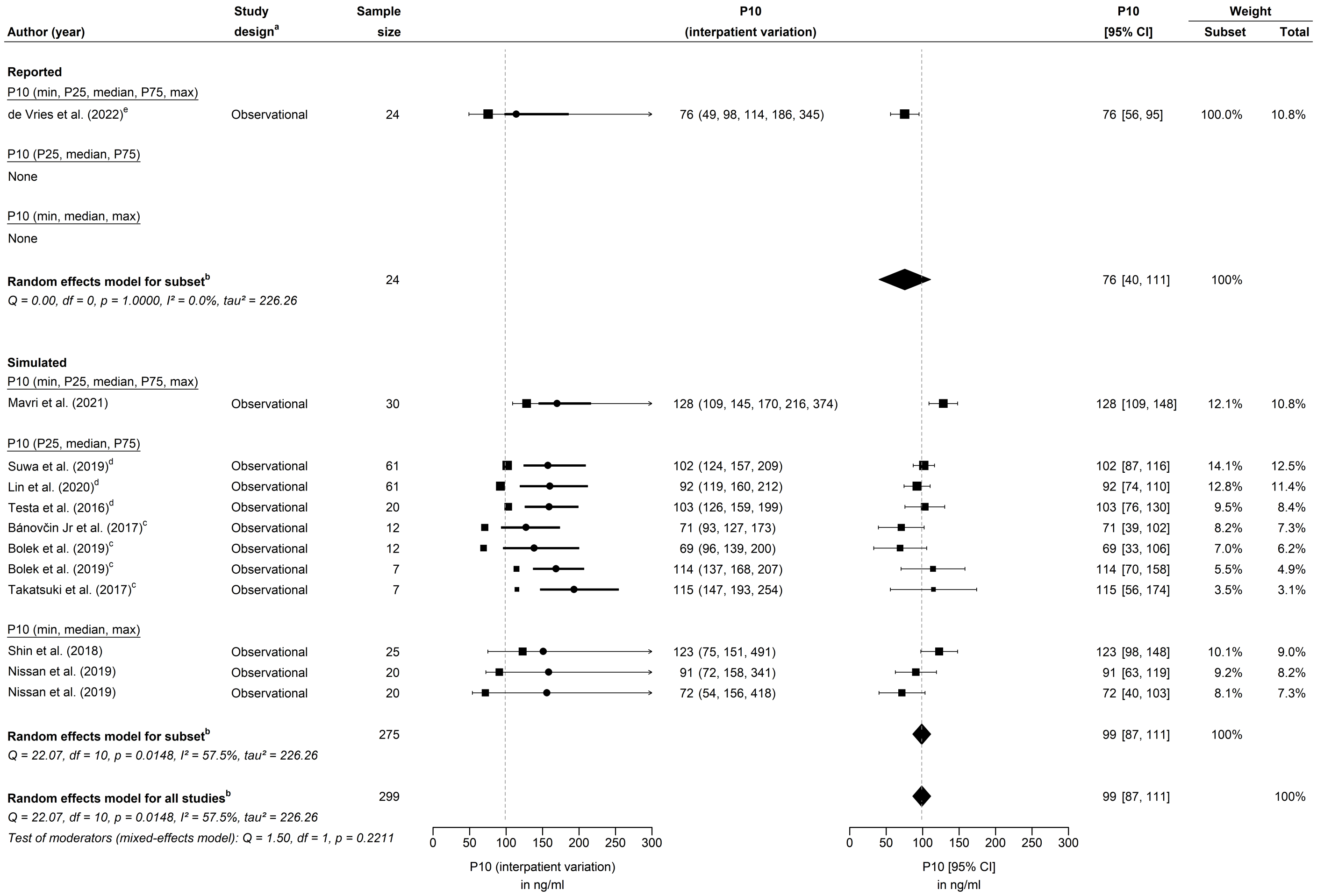

A. Apixaban 2.5 mg twice daily

[a] All analyses of interest were cross-sectional; [b] Random effects model using the quantile-estimation method;[2-5] [c] Simulated values were used because only the mean and standard deviation were available; [d] Simulated values were used because available parameters could not readily be included in the QE-method; [e] Percentiles were calculated directly from the original dataset if they were published by the authors of the current review.[6-8]

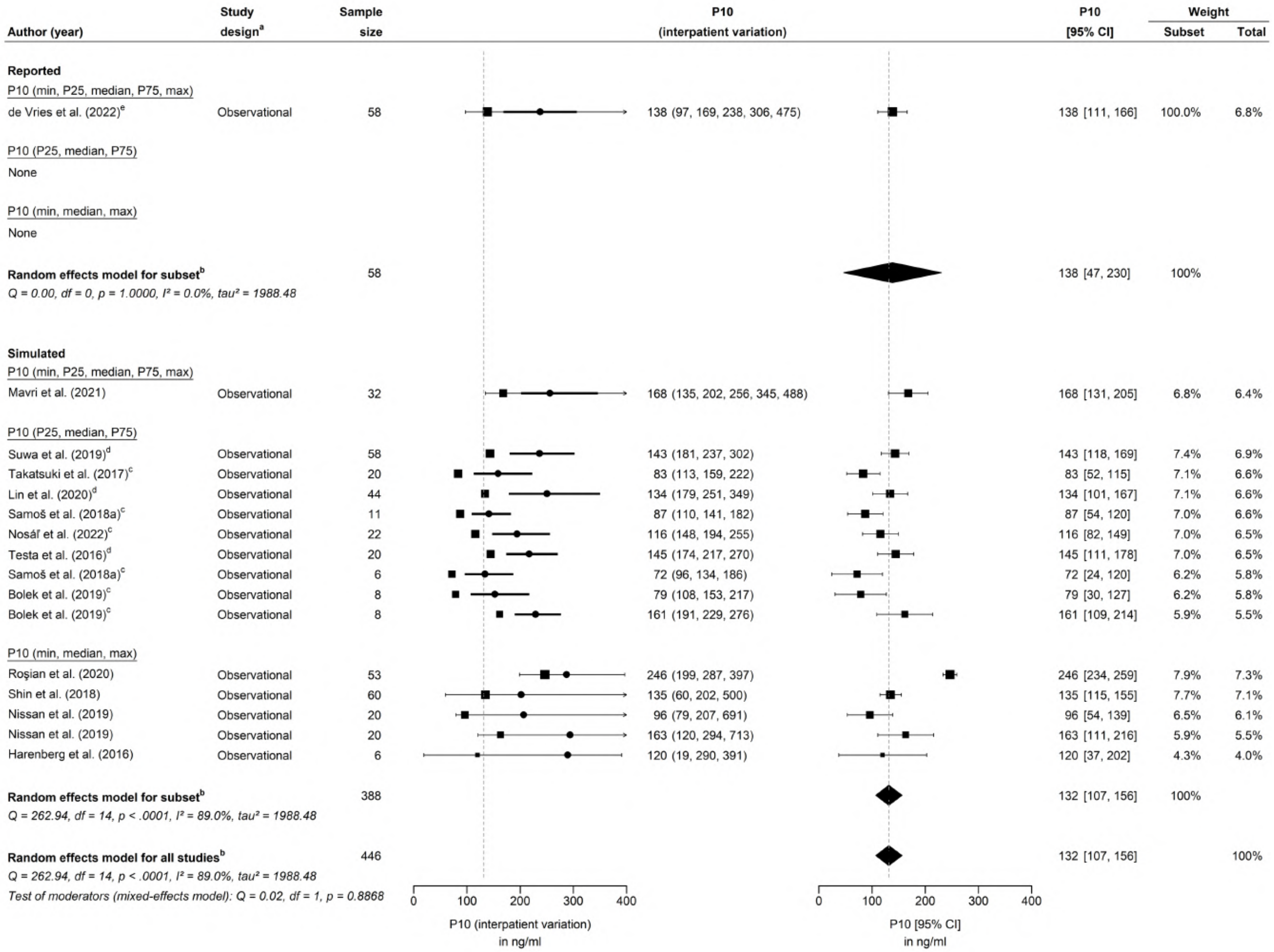

B. Apixaban 5 mg twice daily

[a] All analyses of interest were cross-sectional; [b] Random effects model using the quantile-estimation method;[2-5] [c] Simulated values were used because only the mean and standard deviation were available; [d] Simulated values were used because available parameters could not readily be included in the QE-method; [e] Percentiles were calculated directly from the original dataset if they were published by the authors of the current review.[6-8]

C. Dabigatran 75 mg twice daily

Performing a sensitivity analysis was not possible because only a single study was available.

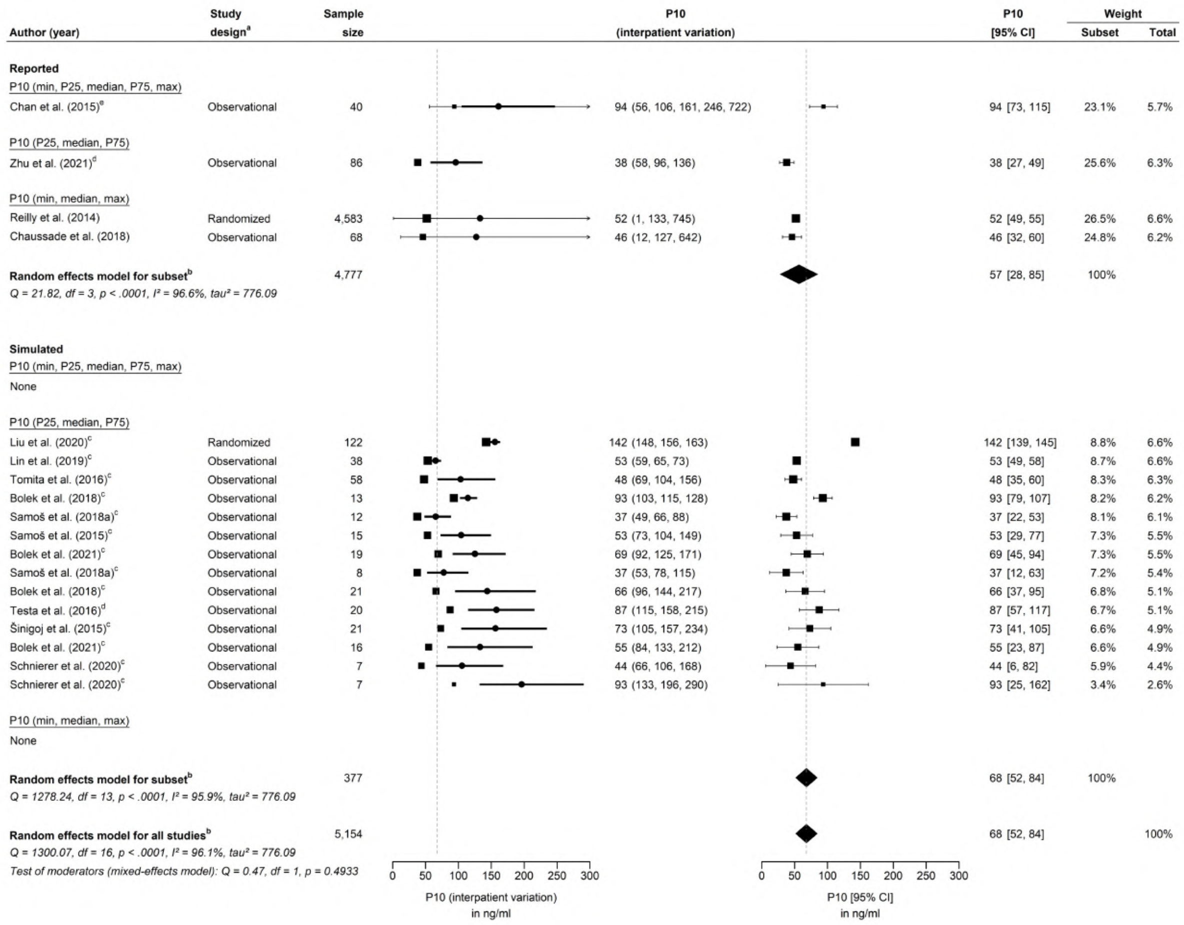

D. Dabigatran 110 mg twice daily

[a] All analyses of interest were cross-sectional; [b] Random effects model using the quantile-estimation method;[2-5] [c] Simulated values were used because only the mean and standard deviation were available; [d] Simulated values were used because available parameters could not readily be included in the QE-method; [e] Percentiles were calculated directly from the original dataset if they were published by the authors of the current review.[6-8]

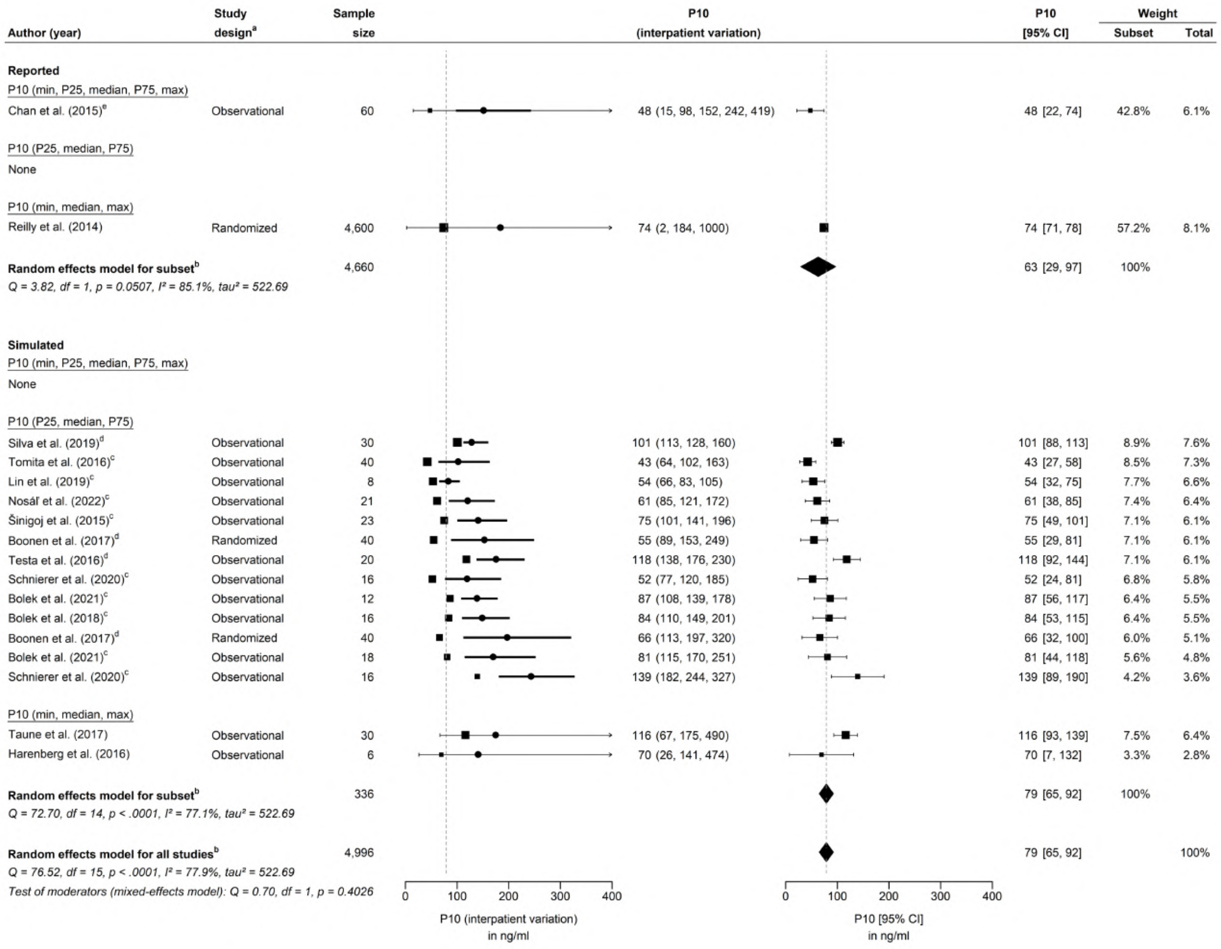

E. Dabigatran 150 mg twice daily

[a] All analyses of interest were cross-sectional; [b] Random effects model using the quantile-estimation method;[2-5] [c] Simulated values were used because only the mean and standard deviation were available; [d] Simulated values were used because available parameters could not readily be included in the QE-method; [e] Percentiles were calculated directly from the original dataset if they were published by the authors of the current review.[6-8]

F. Edoxaban 15 mg once daily

Performing a sensitivity analysis was not possible because only a single study was available.

G. Edoxaban 30 mg once daily

Performing a sensitivity analysis was not possible because none of the two studies reported the 10th percentile.

H. Edoxaban 60 mg once daily

Performing a sensitivity analysis was not possible because none of the two studies reported the 10th percentile.

I. Rivaroxaban 10 mg once daily

Performing a sensitivity analysis was not possible because none of the four studies reported the 10th percentile.

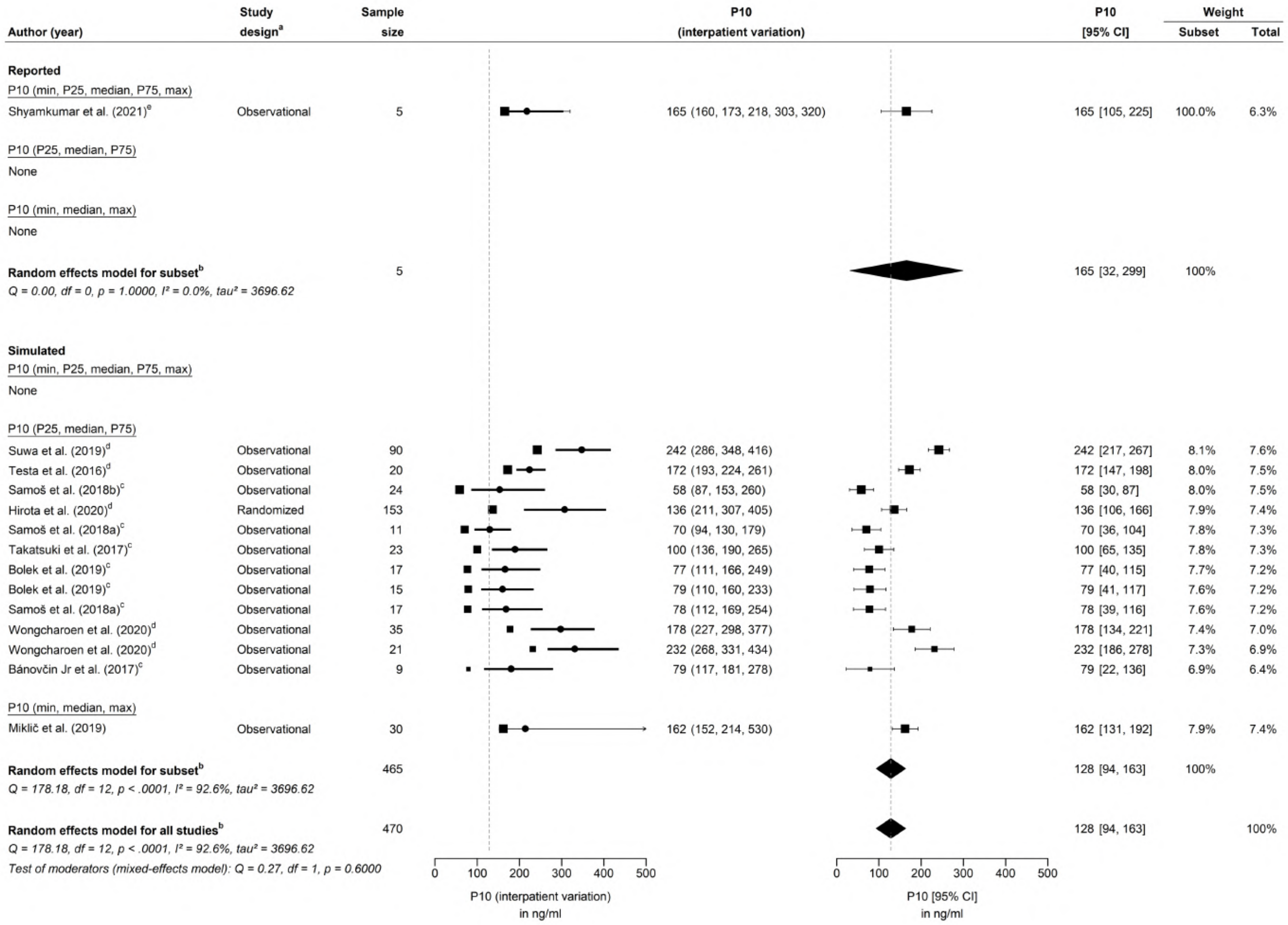

J. Rivaroxaban 15 mg once daily

[a] All analyses of interest were cross-sectional; [b] Random effects model using the quantile-estimation method;[2-5] [c] Simulated values were used because only the mean and standard deviation were available; [d] Simulated values were used because available parameters could not readily be included in the QE-method; [e] Percentiles were calculated directly from the original dataset if they were published by the authors of the current review.[6-8]

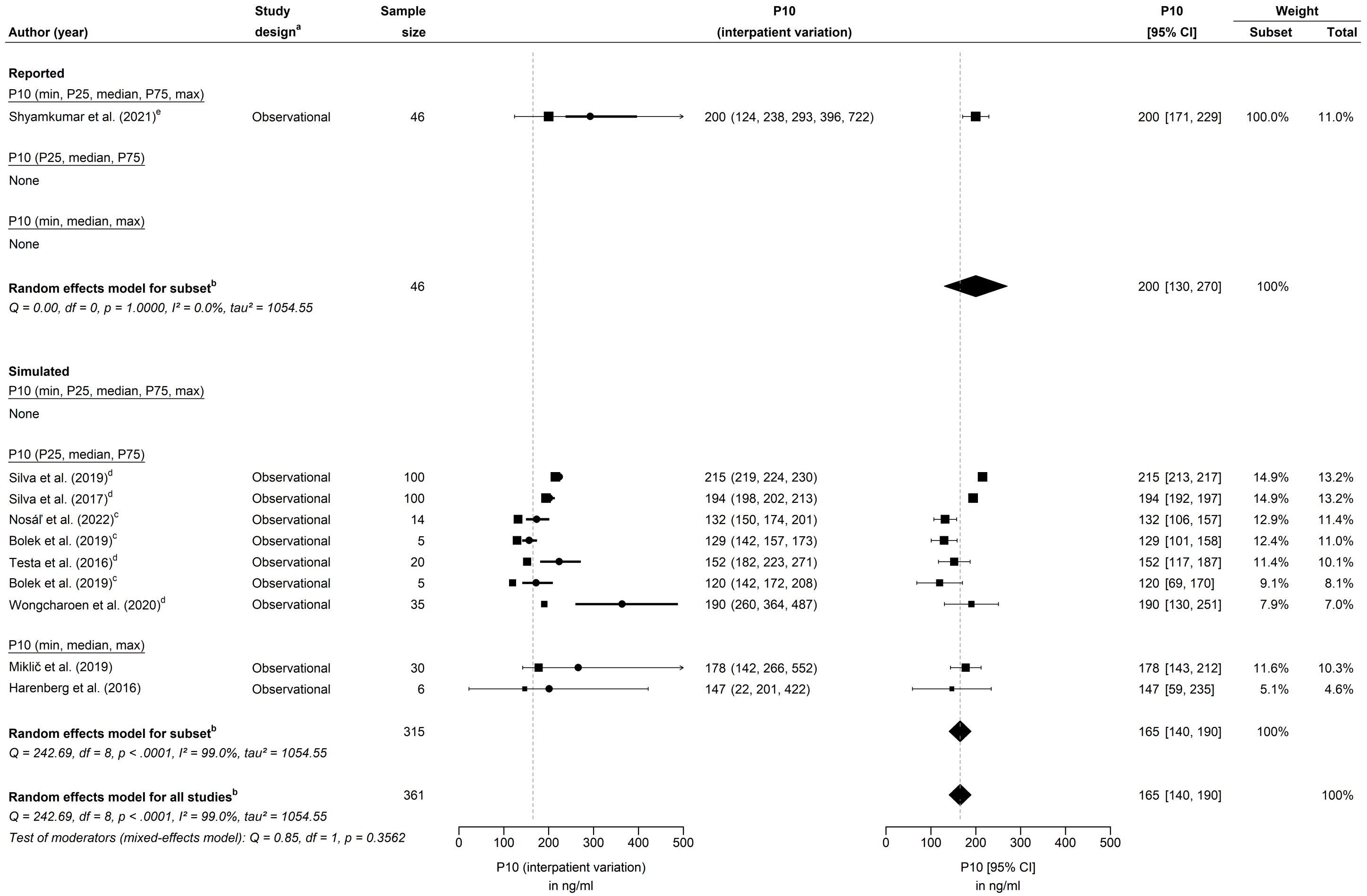

K. Rivaroxaban 20 mg once daily

[a] All analyses of interest were cross-sectional; [b] Random effects model using the quantile-estimation method;[2-5] [c] Simulated values were used because only the mean and standard deviation were available; [d] Simulated values were used because available parameters could not readily be included in the QE-method; [e] Percentiles were calculated directly from the original dataset if they were published by the authors of the current review.[6-8]

Figure S8. Sensitivity analysis 2 (by data extraction method): Estimating the pooled 90th percentile of peak levels of each direct oral anticoagulant stratified by administered dose using the modified QE-method

The forest plots below illustrate the results of our analyses to estimate the 90th percentile of peak levels of each DOAC type, stratified by dosing regimen and by method of data extraction (reported vs. simulated). The squares represent the 90th percentile values, the circles the median values, the solid bold lines the 25th to 75th percentile range, and the whiskers either the minimum to maximum value interval (left side of the plot) or the 95% of the confidence interval of percentile value of interest (right side of the plot).

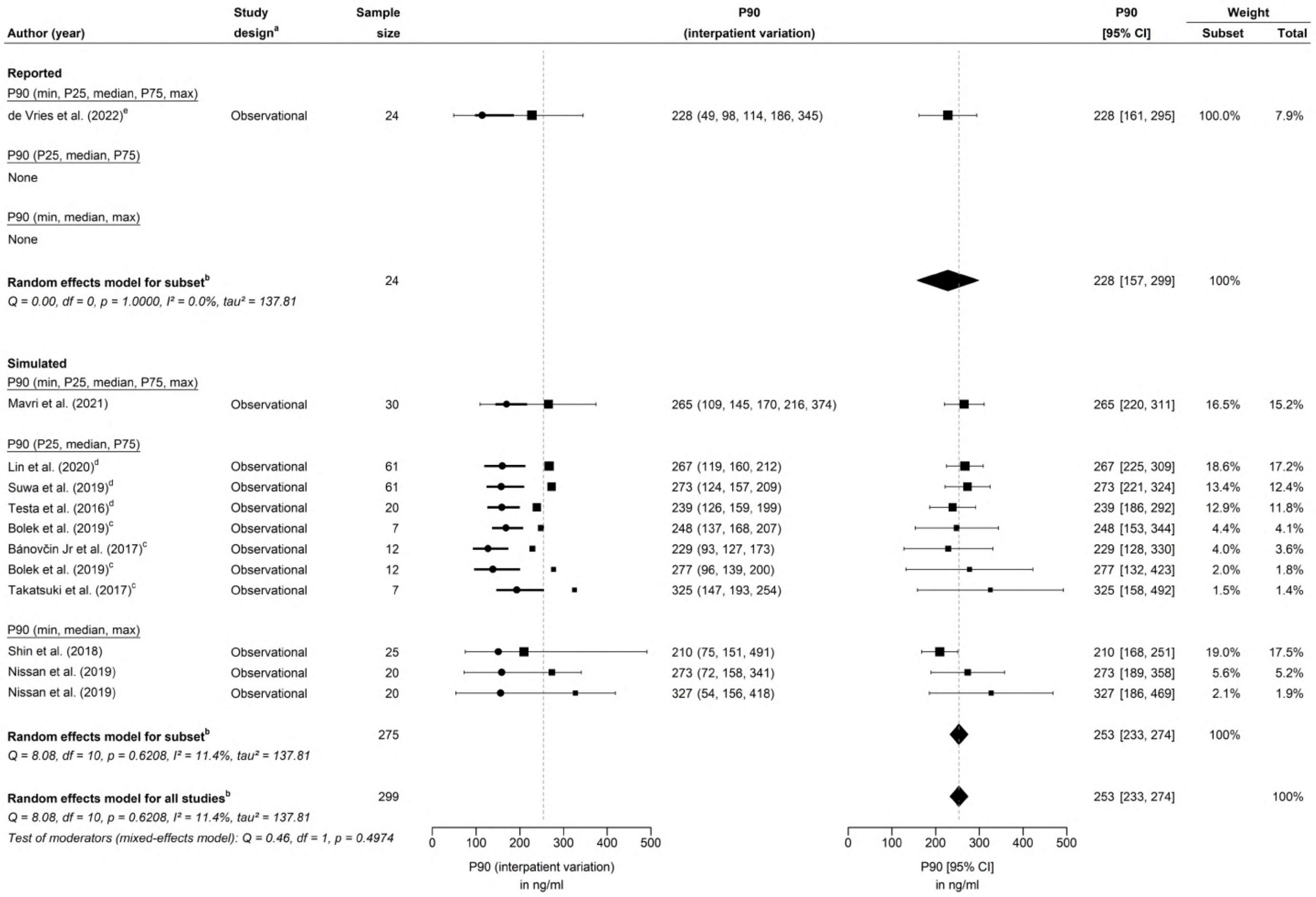

A. Apixaban 2.5 mg twice daily

[a] All analyses of interest were cross-sectional; [b] Random effects model using the quantile-estimation method;[2-5] [c] Simulated values were used because only the mean and standard deviation were available; [d] Simulated values were used because available parameters could not readily be included in the QE-method; [e] Percentiles were calculated directly from the original dataset if they were published by the authors of the current review.[6-8]

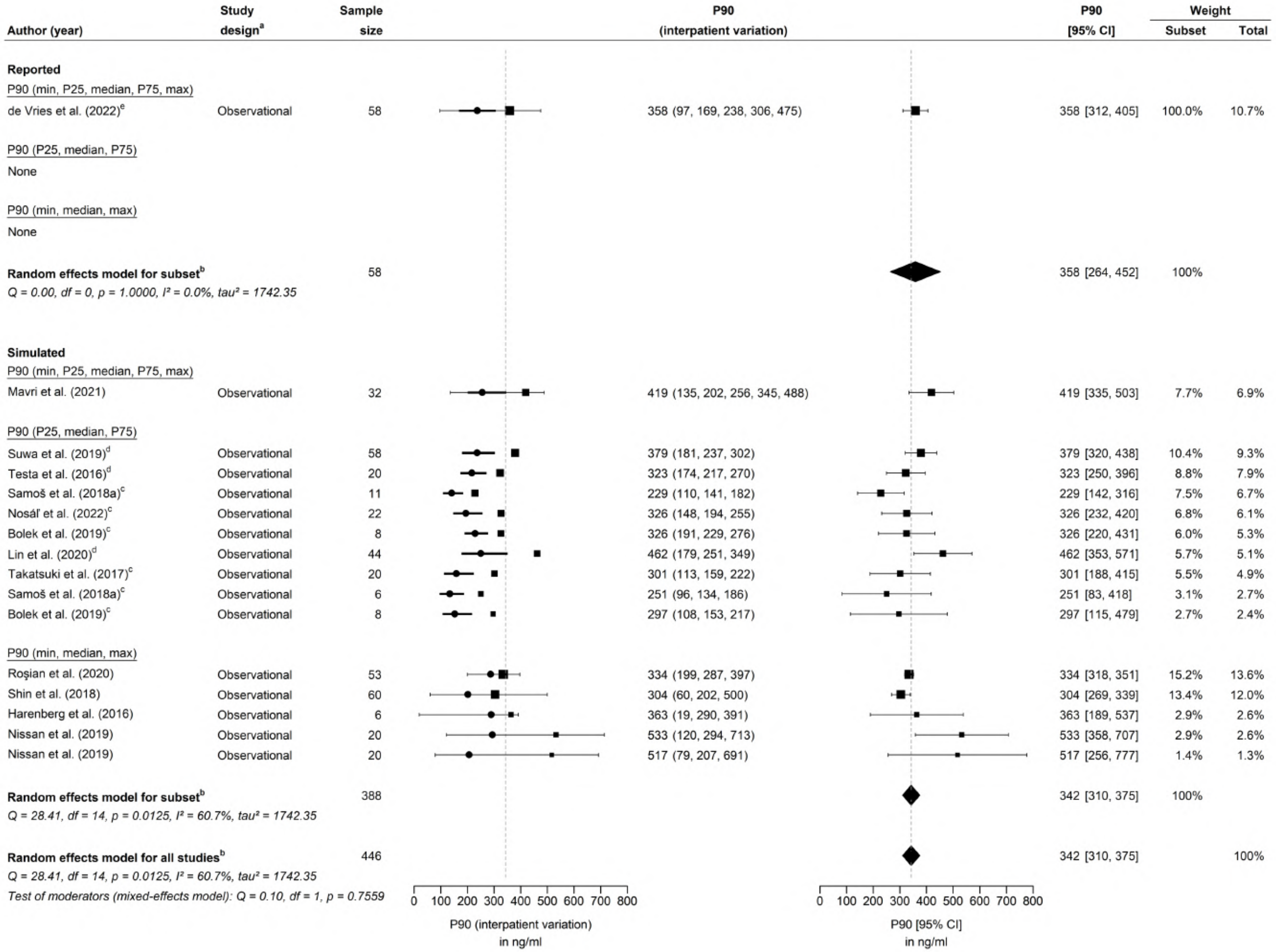

B. Apixaban 5 mg twice daily

[a] All analyses of interest were cross-sectional; [b] Random effects model using the quantile-estimation method;[2-5] [c] Simulated values were used because only the mean and standard deviation were available; [d] Simulated values were used because available parameters could not readily be included in the QE-method; [e] Percentiles were calculated directly from the original dataset if they were published by the authors of the current review.[6-8]

C. Dabigatran 75 mg twice daily

Performing a sensitivity analysis was not possible because only a single study was available.

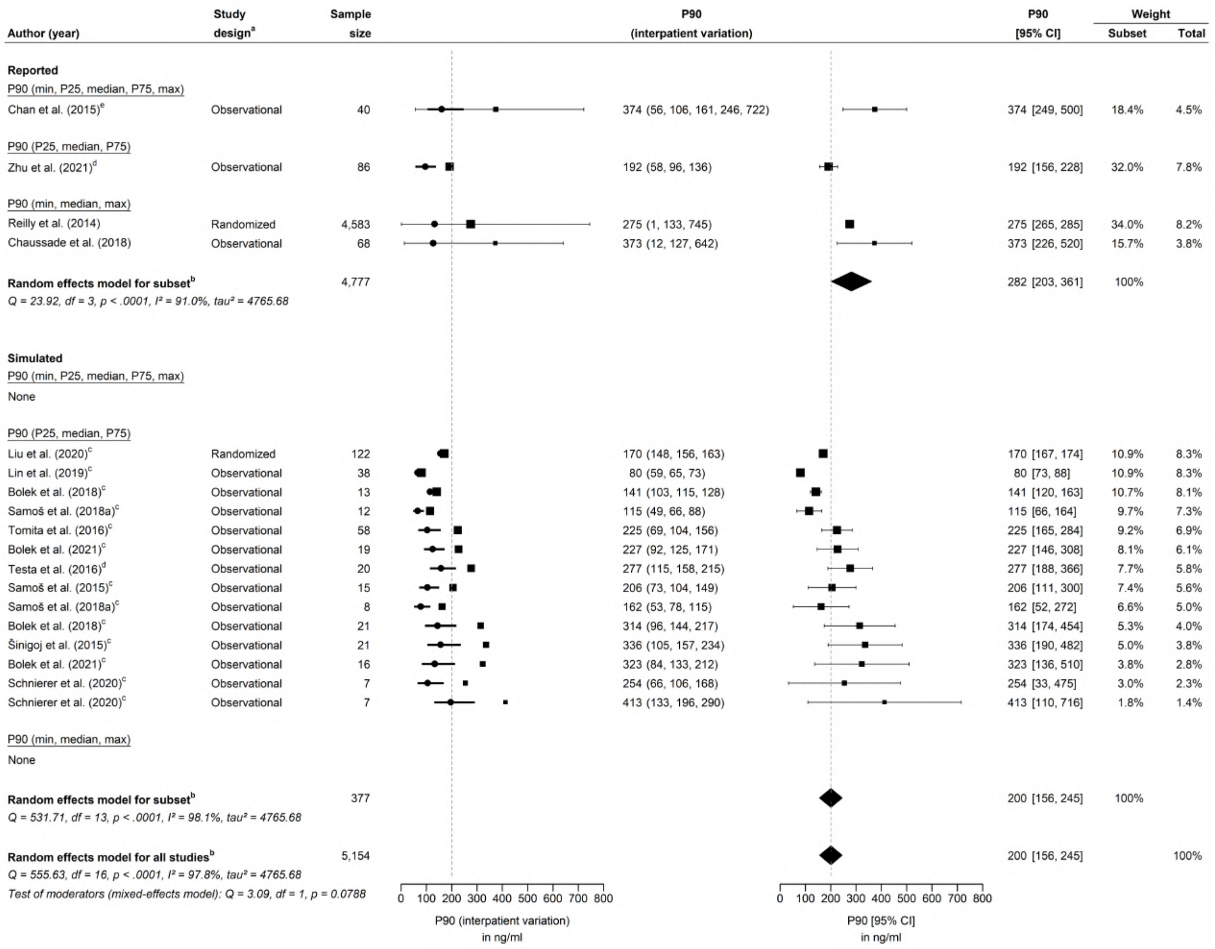

D. Dabigatran 110 mg twice daily

[a] All analyses of interest were cross-sectional; [b] Random effects model using the quantile-estimation method;[2-5] [c] Simulated values were used because only the mean and standard deviation were available; [d] Simulated values were used because available parameters could not readily be included in the QE-method; [e] Percentiles were calculated directly from the original dataset if they were published by the authors of the current review.[6-8]

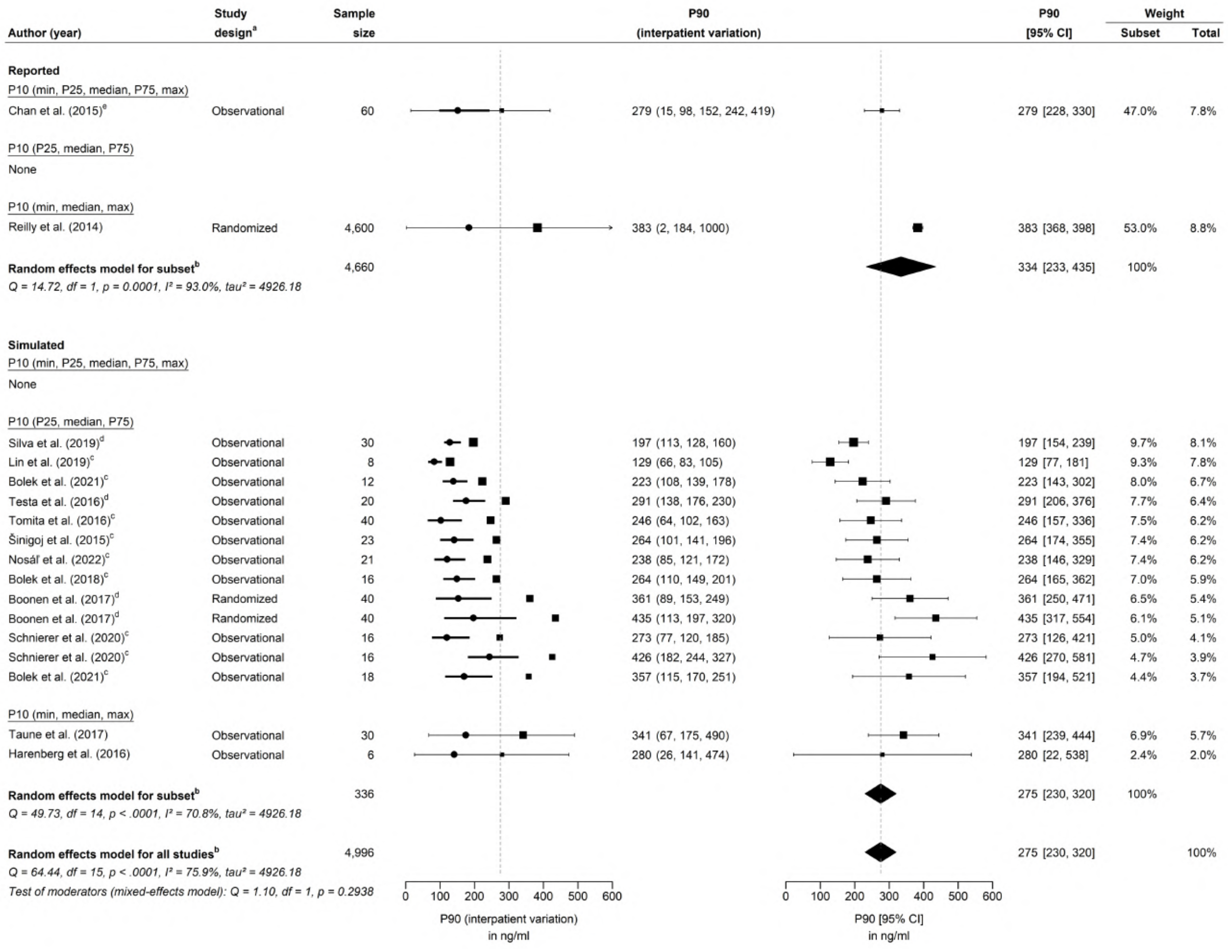

E. Dabigatran 150 mg twice daily

[a] All analyses of interest were cross-sectional; [b] Random effects model using the quantile-estimation method;[2-5] [c] Simulated values were used because only the mean and standard deviation were available; [d] Simulated values were used because available parameters could not readily be included in the QE-method; [e] Percentiles were calculated directly from the original dataset if they were published by the authors of the current review.[6-8]

F. Edoxaban 15 mg once daily

Performing a sensitivity analysis was not possible because only a single study was available.

G. Edoxaban 30 mg once daily

Performing a sensitivity analysis was not possible because none of the two studies reported the 10th percentile.

H. Edoxaban 60 mg once daily

Performing a sensitivity analysis was not possible because none of the two studies reported the 10th percentile.

I. Rivaroxaban 10 mg once daily

Performing a sensitivity analysis was not possible because none of the four studies reported the 10th percentile.

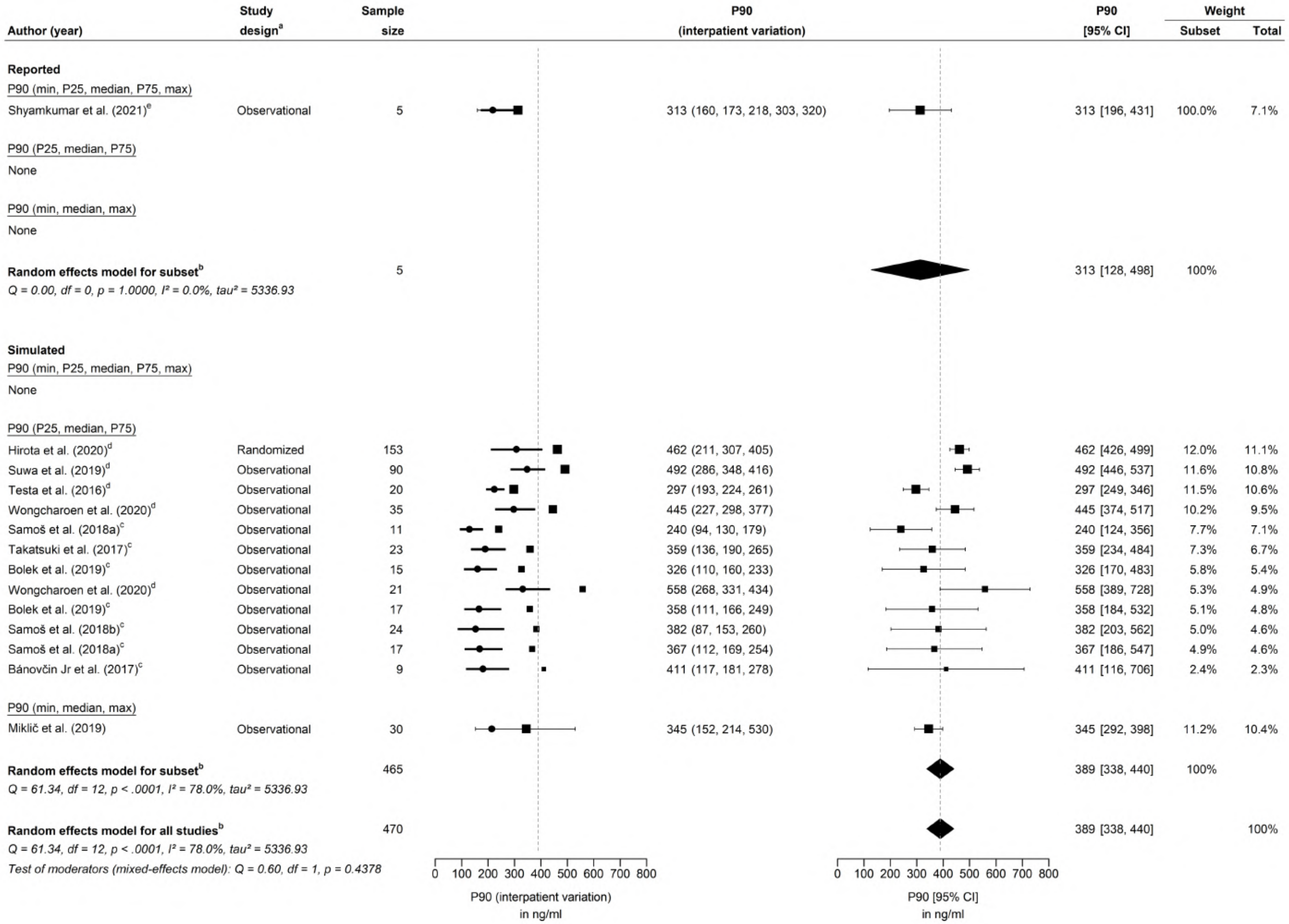

J. Rivaroxaban 15 mg once daily

[a] All analyses of interest were cross-sectional; [b] Random effects model using the quantile-estimation method;[2-5] [c] Simulated values were used because only the mean and standard deviation were available; [d] Simulated values were used because available parameters could not readily be included in the QE-method; [e] Percentiles were calculated directly from the original dataset if they were published by the authors of the current review.[6-8]

Author (year)	Study design[a]	Sample size	P90 (interpatient variation)	P90 [95% CI]	Weight Subset	Weight Total
**Reported**						
P90 (min, P25, median, P75, max)						
Shyamkumar et al. (2021)[e]	Observational	46	461 (124, 238, 293, 396, 722)	461 [390, 532]	100.0%	10.1%
P90 (P25, median, P75)						
None						
P90 (min, median, max)						
None						
**Random effects model for subset[b]**		46		461 [229, 693]	100%	
Q = 0.00, df = 0, p = 1.0000, I² = 0.0%, tau² = 12682.50						
**Simulated**						
P90 (min, P25, median, P75, max)						
None						
P90 (P25, median, P75)						
Silva et al. (2019)[d]	Observational	100	235 (219, 224, 230)	235 [232, 238]	12.4%	11.1%
Silva et al. (2017)[d]	Observational	100	223 (198, 202, 213)	223 [216, 230]	12.4%	11.1%
Bolek et al. (2019)[c]	Observational	5	189 (142, 157, 173)	189 [147, 231]	12.0%	10.7%
Nosáľ et al. (2022)[c]	Observational	14	229 (150, 174, 201)	229 [185, 273]	11.9%	10.7%
Testa et al. (2016)[d]	Observational	20	316 (182, 223, 271)	316 [256, 375]	11.5%	10.4%
Bolek et al. (2019)[c]	Observational	5	248 (142, 172, 208)	248 [142, 353]	10.1%	9.1%
Wongcharoen et al. (2020)[d]	Observational	35	590 (260, 364, 487)	590 [479, 701]	9.9%	8.9%
P90 (min, median, max)						
Miklič et al. (2019)	Observational	30	408 (142, 266, 552)	408 [335, 481]	11.2%	10.0%
Harenberg et al. (2016)	Observational	6	278 (22, 201, 422)	278 [135, 422]	8.7%	7.8%
**Random effects model for subset[b]**		315		296 [218, 374]	100%	
Q = 83.30, df = 8, p < .0001, I² = 99.6%, tau² = 12682.50						
**Random effects model for all studies[b]**		361		296 [218, 374]		100%
Q = 83.30, df = 8, p < .0001, I² = 99.6%, tau² = 12682.50						
Test of moderators (mixed-effects model): Q = 1.75, df = 1, p = 0.1861						

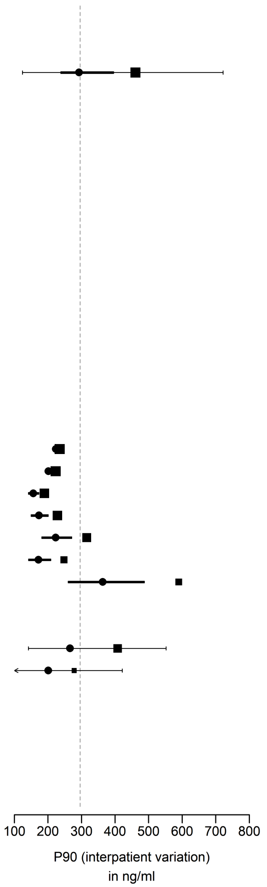

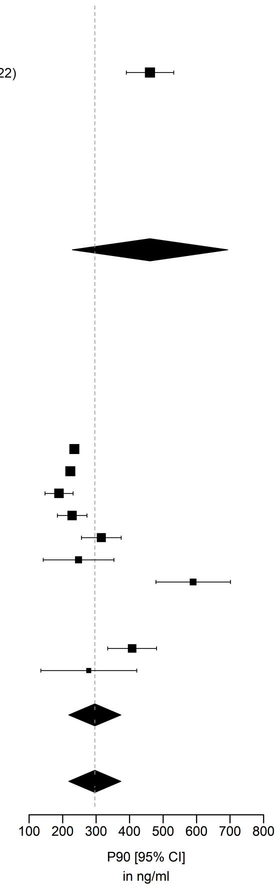

K. Rivaroxaban 20 mg once daily

[a] All analyses of interest were cross-sectional; [b] Random effects model using the quantile-estimation method;[2-5] [c] Simulated values were used because only the mean and standard deviation were available; [d] Simulated values were used because available parameters could not readily be included in the QE-method; [e] Percentiles were calculated directly from the original dataset if they were published by the authors of the current review.[6-8]

Sensitivity analysis 3: Laboratory method

Table S3. Summary of sensitivity analysis 3: Laboratory method (post hoc defined)

	Pooled 10th percentile [95% CI]				Pooled 90th percentile [95% CI]			
**DOAC dosing regimen**	**LC-MS/MS[a]**	**Chromogenic[a]**	**Total**	***p*-value[b]**	**LC-MS/MS[a]**	**Chromogenic[a]**	**Total**	***p*-value[b]**
**Trough**								
Apixaban								
2.5 mg twice daily	39 [23, 55]	38 [30, 46]	38 [30, 46]	0.9040	129 [78, 180]	163 [134, 193]	163 [134, 193]	0.2519
5 mg twice daily	81 [59, 104]	47 [37, 58]	48 [36, 59]	**0.0016**	191 [132, 249]	214 [179, 250]	215 [178, 252]	0.4394
Dabigatran								
75 mg twice daily	NA	NA	NA	NA	NA	NA	NA	NA
110 mg twice daily	29 [22, 36]	37 [31, 44]	37 [31 ,43]	0.1628	29 [22, 36]	37 [31, 44]	37 [31, 43]	0.1628
150 mg twice daily	35 [26, 43]	29 [24, 34]	30 [23, 38]	0.4414	35 [26, 43]	29 [24, 34]	30 [23, 38]	0.4414
Edoxaban								
15 mg once daily	NA	NA	NA	NA	NA	NA	NA	NA
30 mg once daily	NA	NA	NA	NA	NA	NA	NA	NA
60 mg once daily	NA	NA	NA	NA	NA	NA	NA	NA
Rivaroxaban								
10 mg once daily	NA	NA	NA	NA	NA	NA	NA	NA
15 mg once daily	16 [11, 21]	16 [12, 19]	16 [12, 19]	0.9413	16 [11, 21]	16 [12, 19]	16 [12, 19]	0.9413
20 mg once daily	NA	NA	NA	NA	NA	NA	NA	NA
**Peak**								
Apixaban								
2.5 mg twice daily	110 [85, 135]	92 [79, 106]	92 [79, 106]	0.2329	266 [235, 297]	243 [220, 265]	243 [220, 265]	0.2240
5 mg twice daily	187 [146, 228]	119 [97, 140]	119 [97, 140]	**0.0035**	381 [322, 439]	331 [296, 366]	331 [296, 366]	0.1492
Dabigatran								

	Pooled 10th percentile [95% CI]				Pooled 90th percentile [95% CI]			
**DOAC dosing regimen**	**LC-MS/MS[a]**	**Chromogenic[a]**	**Total**	***p*-value[b]**	**LC-MS/MS[a]**	**Chromogenic[a]**	**Total**	***p*-value[b]**
75 mg twice daily	NA	NA	NA	NA	NA	NA	NA	NA
110 mg twice daily	50 [45, 56]	71 [53, 89]	71 [55, 87]	0.1975	50 [45, 56]	71 [53, 89]	71 [55, 87]	0.1975
150 mg twice daily	69 [51, 87]	83 [66, 100]	83 [66, 100]	0.2658	69 [51, 87]	83 [66, 100]	83 [66, 100]	0.2658
Edoxaban								
15 mg once daily	NA	NA	NA	NA	NA	NA	NA	NA
30 mg once daily	NA	NA	NA	NA	NA	NA	NA	NA
60 mg once daily	NA	NA	NA	NA	NA	NA	NA	NA
Rivaroxaban								
10 mg once daily	NA	NA	NA	NA	NA	NA	NA	NA
15 mg once daily	149 [61, 236]	127 [91, 164]	127 [91, 164]	0.6580	149 [61, 236]	127 [91, 164]	127 [91, 164]	0.6580
20 mg once daily	178 [103, 253]	168 [142, 193]	168 [142, 193]	0.8034	178 [103, 253]	168 [142, 193]	168 [142, 193]	0.8034

This table summarizes the results of the sensitivity analyses on the laboratory method used to determine levels (i.e., liquid chromatography-mass spectrometry/mass spectrometry vs. chromogenic assays), to assess the robustness of our findings on the 10th and 90th percentiles. This analysis was defined post hoc and was requested by one of the peer-reviewers. We defined a significant difference as a *p*-value of <0.05 of the test of moderators (**bold** in table), though we acknowledge this has major limitations. We urge readers to appraise the individual forest plots (**Fig S9 through S12**).

LC-MS/MS liquid chromatography-mass spectrometry/mass spectrometry; *NA* not applicable.

[a] We kept Tau constant if there were five or fewer studies in either category;[1] [b] P-value of the test of moderators (two-tailed Wald-test).

Figure S9. Sensitivity analysis 3 (by laboratory method): Estimating the pooled 10th percentile of trough levels of each direct oral anticoagulant stratified by administered dose using the modified QE-method

The forest plots below illustrate the results of our analyses to estimate the 10th percentile of trough levels of each DOAC type, stratified by dosing regimen and by laboratory method (i.e., liquid chromatography–mass spectrometry/mass spectrometry vs. chromogenic assays). The squares represent the 10th percentile values, the circles the median values, the solid bold lines the 25th to 75th percentile range, and the whiskers either the minimum to maximum value interval (left side of the plot) or the 95% of the confidence interval of percentile value of interest (right side of the plot).

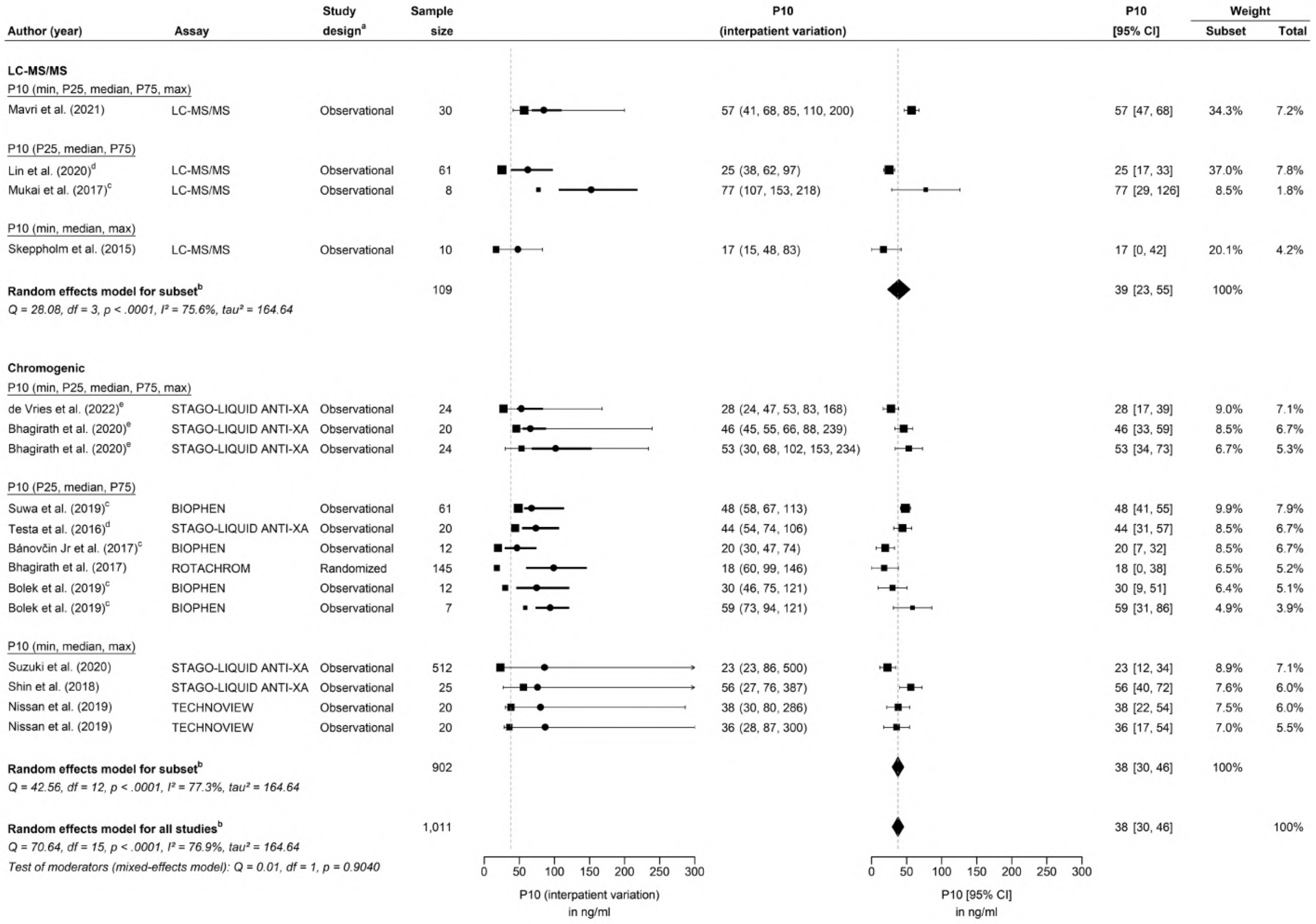

A. Apixaban 2.5 mg twice daily

[a] All analyses of interest were cross-sectional; [b] Random effects model using the quantile-estimation method;[2-5] [c] Simulated values were used because only the mean and standard deviation were available; [d] Simulated values were used because available parameters could not readily be included in the QE-method; [e] Percentiles were calculated directly from the original dataset if they were published by the authors of the current review.[6-8]

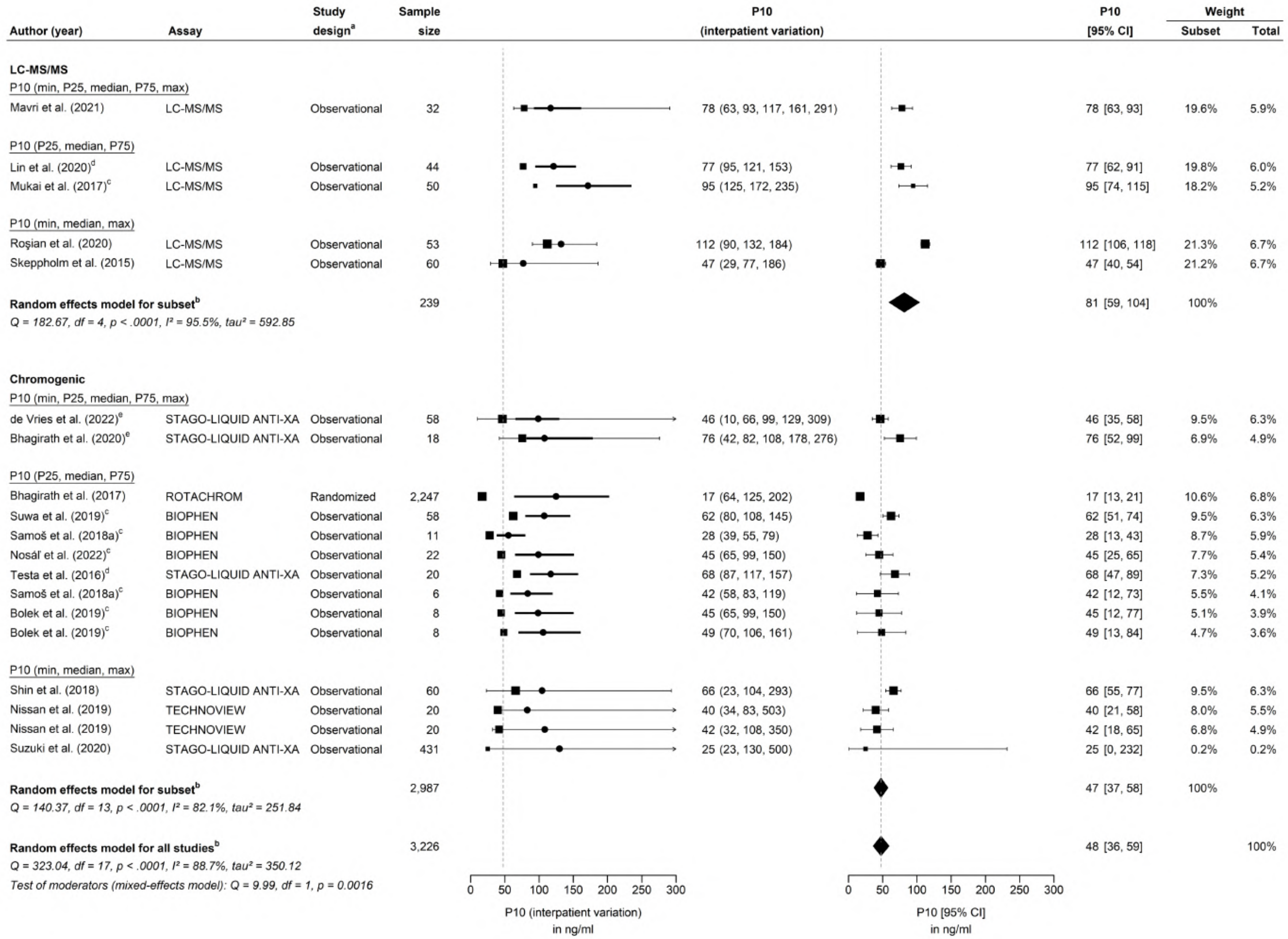

B. Apixaban 5 mg twice daily

[a] All analyses of interest were cross-sectional; [b] Random effects model using the quantile-estimation method;[2-5] [c] Simulated values were used because only the mean and standard deviation were available; [d] Simulated values were used because available parameters could not readily be included in the QE-method; [e] Percentiles were calculated directly from the original dataset if they were published by the authors of the current review.[6-8]

C. Dabigatran 75 mg twice daily

Performing a sensitivity analysis was not possible because only a single study was available.

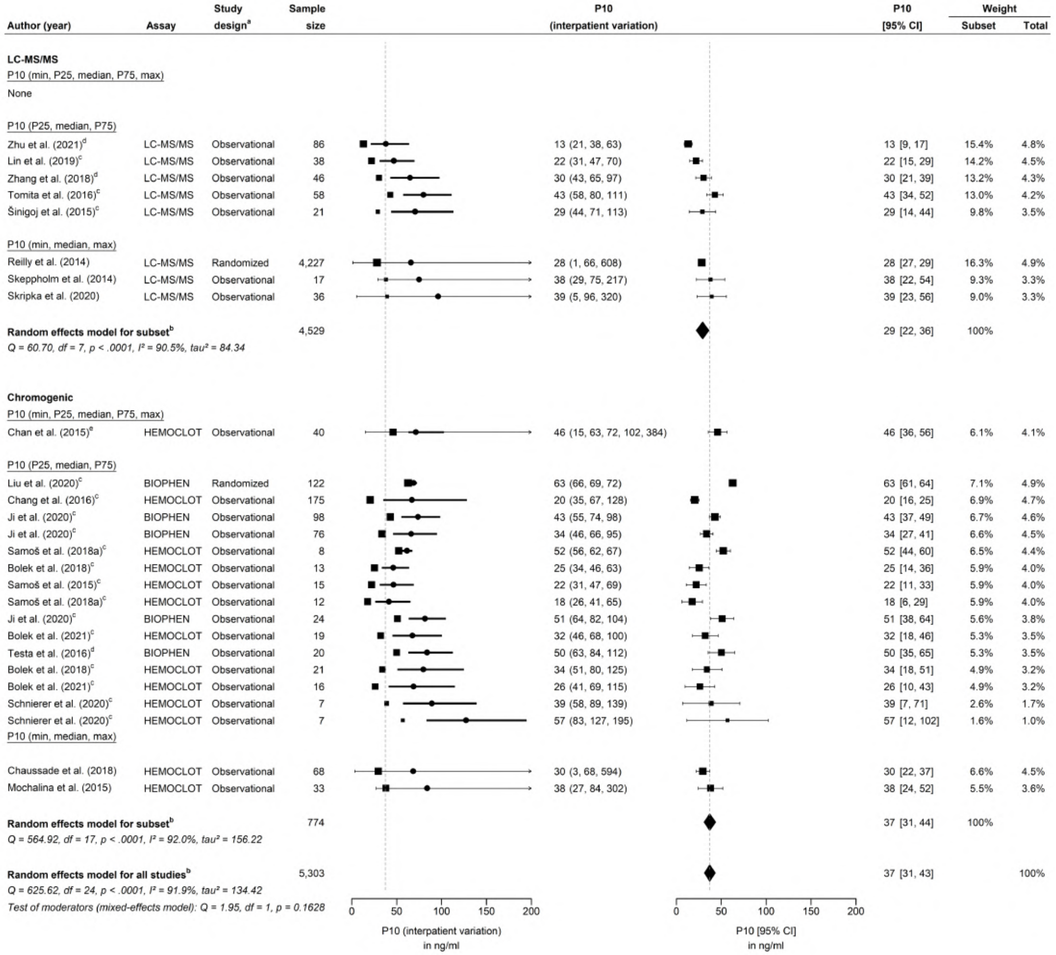

D. Dabigatran 110 mg twice daily

[a] All analyses of interest were cross-sectional; [b] Random effects model using the quantile-estimation method;[2-5] [c] Simulated values were used because only the mean and standard deviation were available; [d] Simulated values were used because available parameters could not readily be included in the QE-method; [e] Percentiles were calculated directly from the original dataset if they were published by the authors of the current review.[6-8]

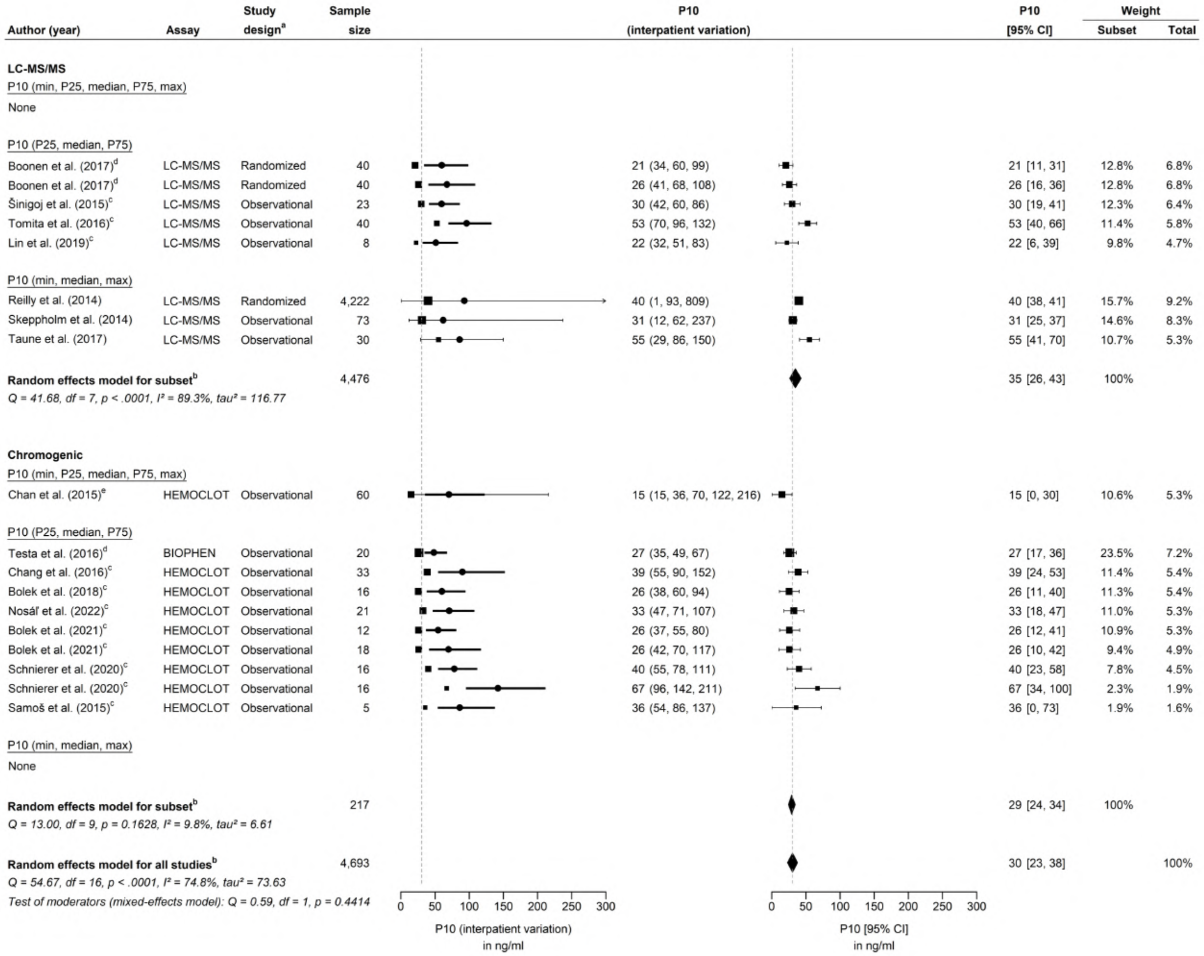

E. Dabigatran 150 mg twice daily

[a] All analyses of interest were cross-sectional; [b] Random effects model using the quantile-estimation method;[2-5] [c] Simulated values were used because only the mean and standard deviation were available; [d] Simulated values were used because available parameters could not readily be included in the QE-method; [e] Percentiles were calculated directly from the original dataset if they were published by the authors of the current review.[6-8]

F. Edoxaban 15 mg once daily

Performing a sensitivity analysis was not possible because both studies used liquid chromatography–mass spectrometry/mass spectrometry to determine drug levels.

G. Edoxaban 30 mg once daily

We did not perform a sensitivity analysis because fewer than ten studies were available (see **Supporting Information File 2**).[1] Three studies used liquid chromatography–mass spectrometry/mass spectrometry to determine drug levels and one used a chromogenic assay.

H. Edoxaban 60 mg once daily

We did not perform a sensitivity analysis because fewer than ten studies were available (see **Supporting Information File 2**).[1] Two studies used liquid chromatography–mass spectrometry/mass spectrometry to determine drug levels and one used a chromogenic assay.

I. Rivaroxaban 10 mg once daily

We did not perform a sensitivity analysis because fewer than ten studies were available (see **Supporting Information File 2**).[1] Two studies used liquid chromatography–mass spectrometry/mass spectrometry to determine drug levels and two used a chromogenic assay.

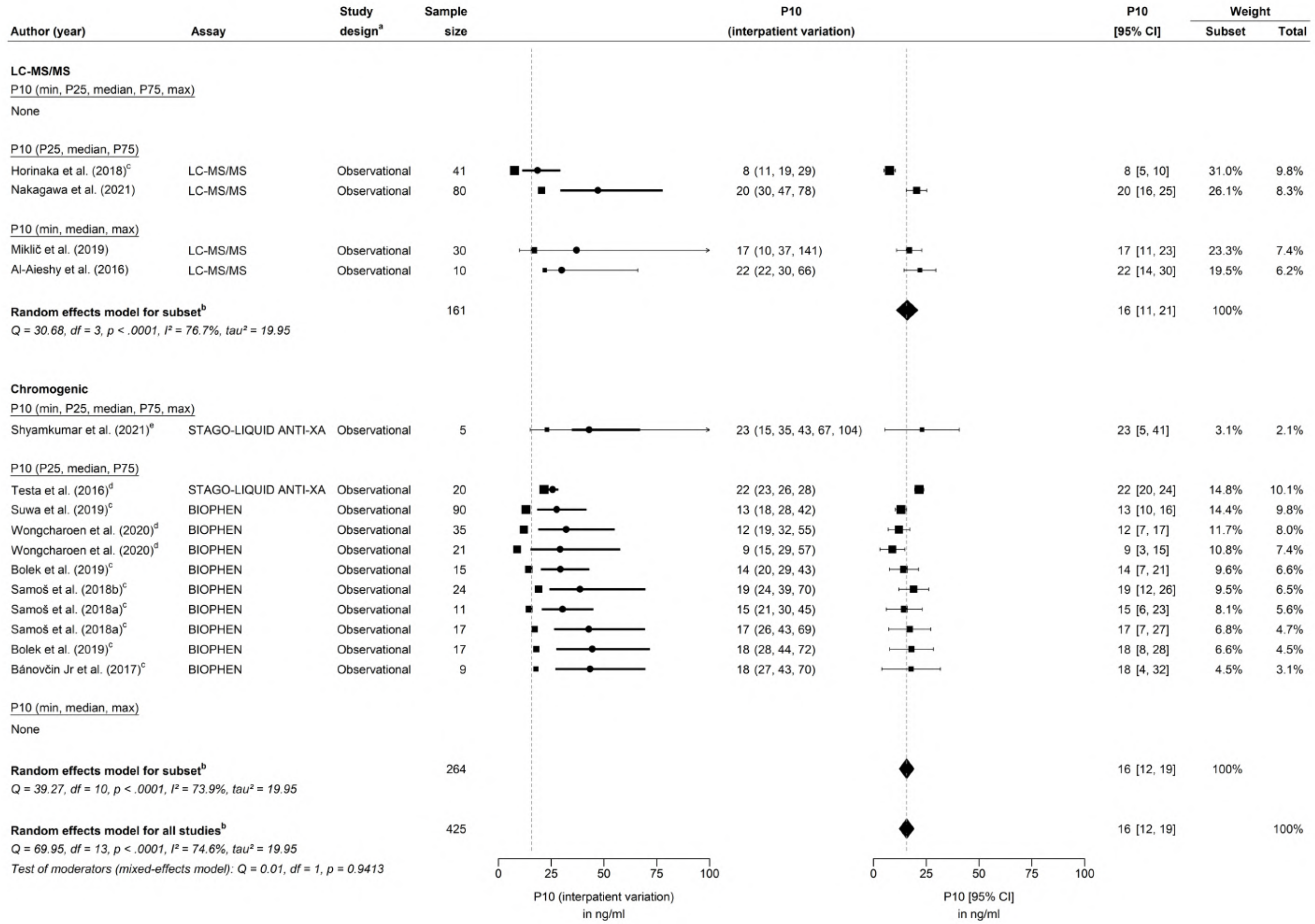

Author (year)	Assay	Study design[a]	Sample size	P10 (interpatient variation)	P10 [95% CI]	Weight Subset	Weight Total
**LC-MS/MS**							
P10 (min, P25, median, P75, max)							
None							
P10 (P25, median, P75)							
Horinaka et al. (2018)[c]	LC-MS/MS	Observational	41	8 (11, 19, 29)	8 [5, 10]	31.0%	9.8%
Nakagawa et al. (2021)	LC-MS/MS	Observational	80	20 (30, 47, 78)	20 [16, 25]	26.1%	8.3%
P10 (min, median, max)							
Miklič et al. (2019)	LC-MS/MS	Observational	30	17 (10, 37, 141)	17 [11, 23]	23.3%	7.4%
Al-Aieshy et al. (2016)	LC-MS/MS	Observational	10	22 (22, 30, 66)	22 [14, 30]	19.5%	6.2%
**Random effects model for subset[b]**			161		16 [11, 21]	100%	
Q = 30.68, df = 3, p < .0001, I² = 76.7%, tau² = 19.95							
**Chromogenic**							
P10 (min, P25, median, P75, max)							
Shyamkumar et al. (2021)[e]	STAGO-LIQUID ANTI-XA	Observational	5	23 (15, 35, 43, 67, 104)	23 [5, 41]	3.1%	2.1%
P10 (P25, median, P75)							
Testa et al. (2016)[d]	STAGO-LIQUID ANTI-XA	Observational	20	22 (23, 26, 28)	22 [20, 24]	14.8%	10.1%
Suwa et al. (2019)[c]	BIOPHEN	Observational	90	13 (18, 28, 42)	13 [10, 16]	14.4%	9.8%
Wongcharoen et al. (2020)[d]	BIOPHEN	Observational	35	12 (19, 32, 55)	12 [7, 17]	11.7%	8.0%
Wongcharoen et al. (2020)[d]	BIOPHEN	Observational	21	9 (15, 29, 57)	9 [3, 15]	10.8%	7.4%
Bolek et al. (2019)[c]	BIOPHEN	Observational	15	14 (20, 29, 43)	14 [7, 21]	9.6%	6.6%
Samoš et al. (2018b)[c]	BIOPHEN	Observational	24	19 (24, 39, 70)	19 [12, 26]	9.5%	6.5%
Samoš et al. (2018a)[c]	BIOPHEN	Observational	11	15 (21, 30, 45)	15 [6, 23]	8.1%	5.6%
Samoš et al. (2018a)[c]	BIOPHEN	Observational	17	17 (26, 43, 69)	17 [7, 27]	6.8%	4.7%
Bolek et al. (2019)[c]	BIOPHEN	Observational	17	18 (28, 44, 72)	18 [8, 28]	6.6%	4.5%
Bánovčin Jr et al. (2017)[c]	BIOPHEN	Observational	9	18 (27, 43, 70)	18 [4, 32]	4.5%	3.1%
P10 (min, median, max)							
None							
**Random effects model for subset[b]**			264		16 [12, 19]	100%	
Q = 39.27, df = 10, p < .0001, I² = 73.9%, tau² = 19.95							
**Random effects model for all studies[b]**			425		16 [12, 19]		100%
Q = 69.95, df = 13, p < .0001, I² = 74.6%, tau² = 19.95							
Test of moderators (mixed-effects model): Q = 0.01, df = 1, p = 0.9413							

J. Rivaroxaban 15 mg once daily

[a] All analyses of interest were cross-sectional; [b] Random effects model using the quantile-estimation method;[2-5] [c] Simulated values were used because only the mean and standard deviation were available; [d] Simulated values were used because available parameters could not readily be included in the QE-method; [e] Percentiles were calculated directly from the original dataset if they were published by the authors of the current review.[6-8]

K. Rivaroxaban 20 mg once daily

We did not perform a sensitivity analysis because fewer than ten studies were available (see **Supporting Information File 2**).[1] Two studies used liquid chromatography–mass spectrometry/mass spectrometry to determine drug levels and six used a chromogenic assay.

Figure S10. Sensitivity analysis 3 (by laboratory method): Estimating the pooled 90th percentile of trough levels of each direct oral anticoagulant stratified by administered dose using the modified QE-method

The forest plots below illustrate the results of our analyses to estimate the 90th percentile of trough levels of each DOAC type, stratified by dosing regimen and by laboratory method (i.e., liquid chromatography–mass spectrometry/mass spectrometry vs. chromogenic assays). The squares represent the 90th percentile values, the circles the median values, the solid bold lines the 25th to 75th percentile range, and the whiskers either the minimum to maximum value interval (left side of the plot) or the 95% of the confidence interval of the percentile value of interest (right side of the plot).

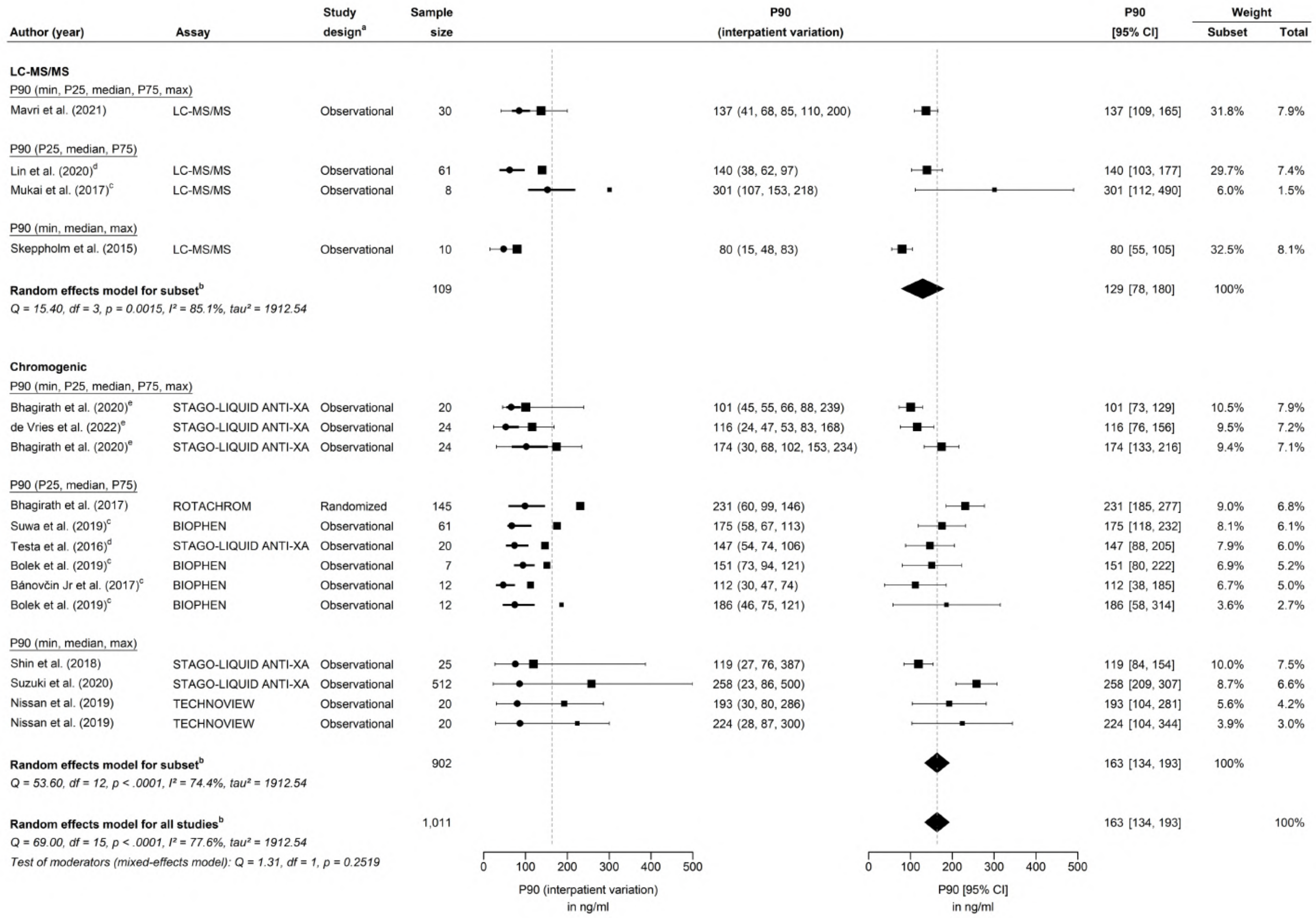

A. Apixaban 2.5 mg twice daily

[a] All analyses of interest were cross-sectional; [b] Random effects model using the quantile-estimation method;[2-5] [c] Simulated values were used because only the mean and standard deviation were available; [d] Simulated values were used because available parameters could not readily be included in the QE-method; [e] Percentiles were calculated directly from the original dataset if they were published by the authors of the current review.[6-8]

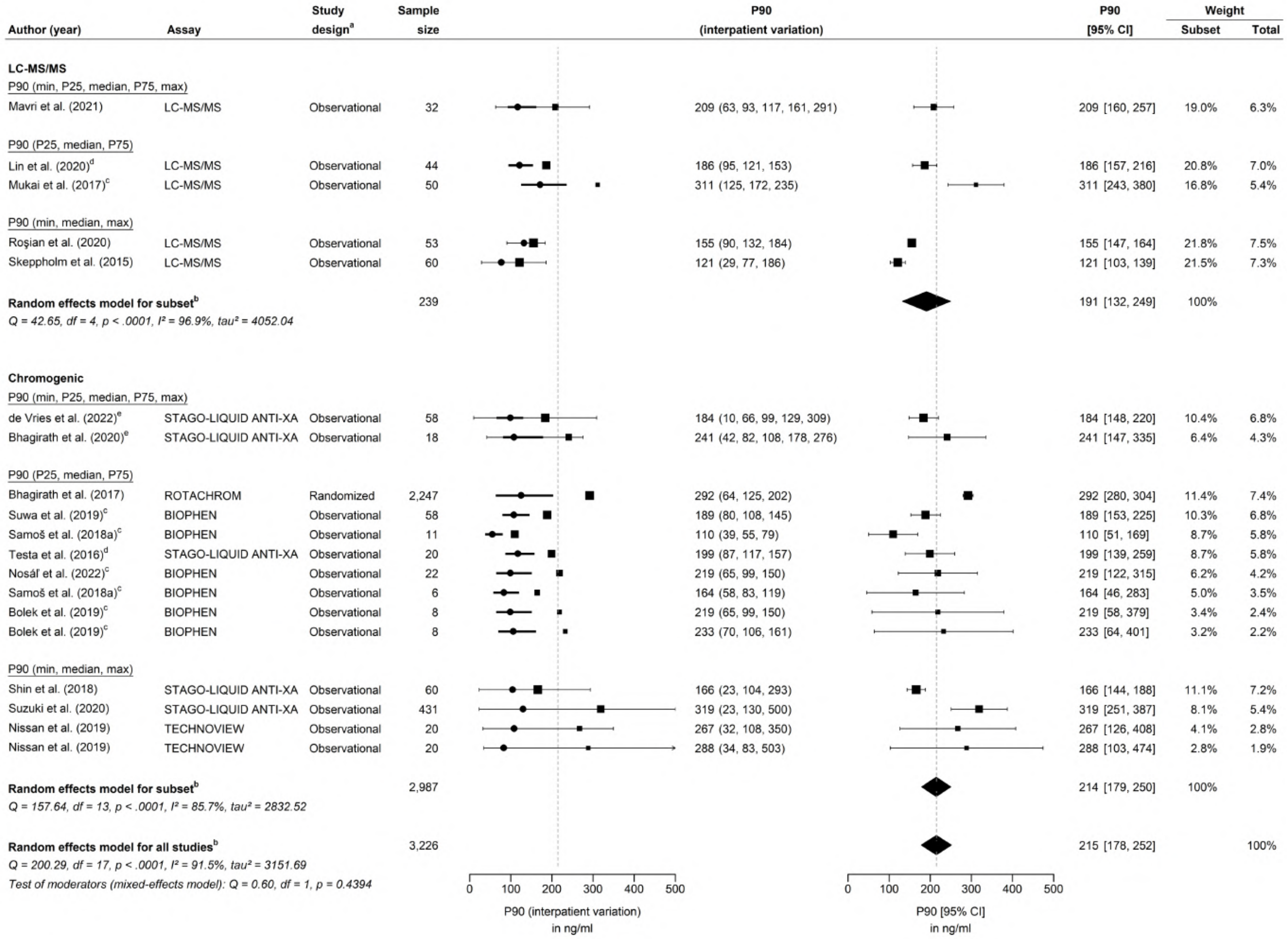

B. Apixaban 5 mg twice daily

[a] All analyses of interest were cross-sectional; [b] Random effects model using the quantile-estimation method;[2-5] [c] Simulated values were used because only the mean and standard deviation were available; [d] Simulated values were used because available parameters could not readily be included in the QE-method; [e] Percentiles were calculated directly from the original dataset if they were published by the authors of the current review.[6-8]

C. Dabigatran 75 mg twice daily

Performing a sensitivity analysis was not possible because only a single study was available.

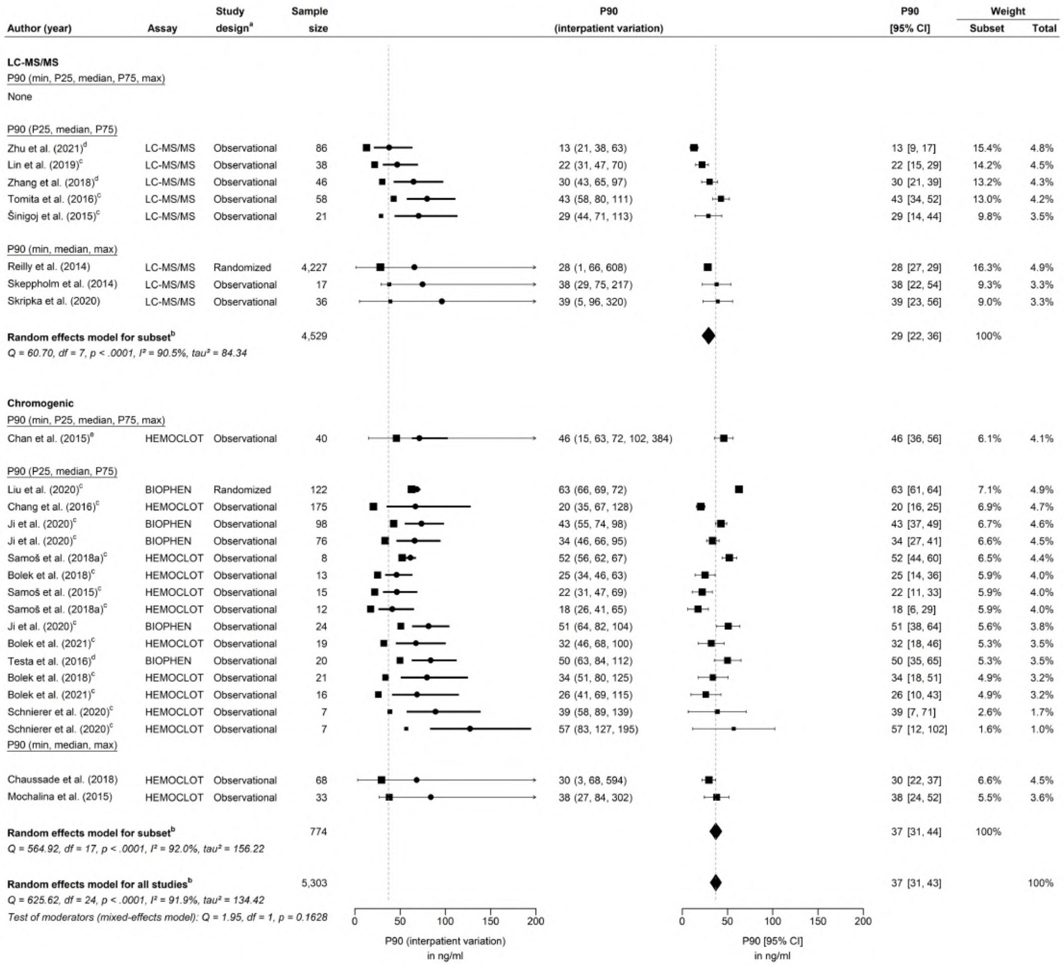

D. Dabigatran 110 mg twice daily

[a] All analyses of interest were cross-sectional; [b] Random effects model using the quantile-estimation method;[2-5] [c] Simulated values were used because only the mean and standard deviation were available; [d] Simulated values were used because available parameters could not readily be included in the QE-method; [e] Percentiles were calculated directly from the original dataset if they were published by the authors of the current review.[6-8]

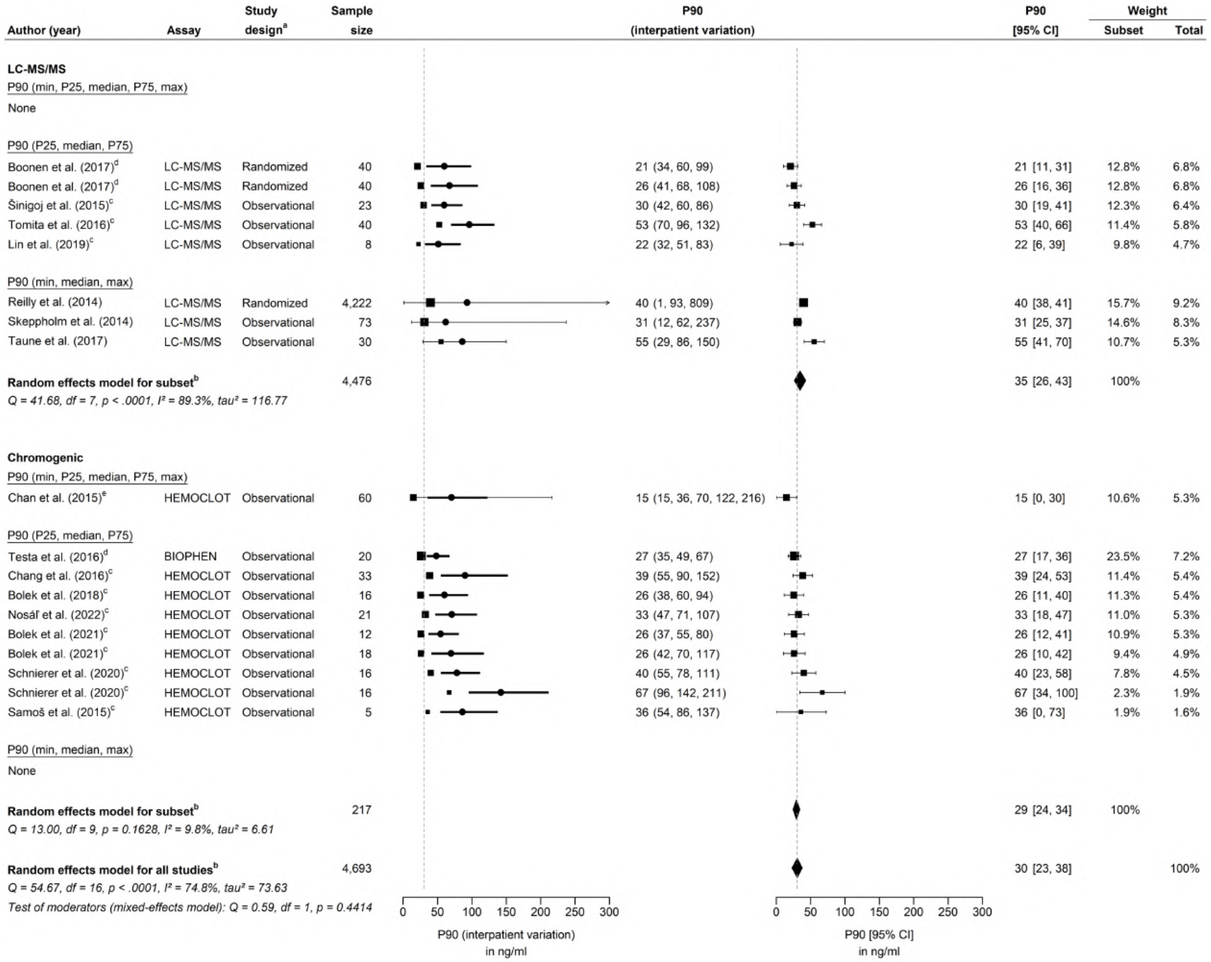

E. Dabigatran 150 mg twice daily

[a] All analyses of interest were cross-sectional; [b] Random effects model using the quantile-estimation method;[2-5] [c] Simulated values were used because only the mean and standard deviation were available; [d] Simulated values were used because available parameters could not readily be included in the QE-method; [e] Percentiles were calculated directly from the original dataset if they were published by the authors of the current review.[6-8]

F. Edoxaban 15 mg once daily

Performing a sensitivity analysis was not possible because both studies used liquid chromatography–mass spectrometry/mass spectrometry to determine drug levels.

G. Edoxaban 30 mg once daily

We did not perform a sensitivity analysis because fewer than ten studies were available (see **Supporting Information File 2**).[1] Three studies used liquid chromatography–mass spectrometry/mass spectrometry to determine drug levels and one used a chromogenic assay.

H. Edoxaban 60 mg once daily

We did not perform a sensitivity analysis because fewer than ten studies were available (see **Supporting Information File 2**).[1] Two studies used liquid chromatography–mass spectrometry/mass spectrometry to determine drug levels and one used a chromogenic assay.

I. Rivaroxaban 10 mg once daily

We did not perform a sensitivity analysis because fewer than ten studies were available (see **Supporting Information File 2**).[1] Two studies used liquid chromatography–mass spectrometry/mass spectrometry to determine drug levels and two used a chromogenic assay.

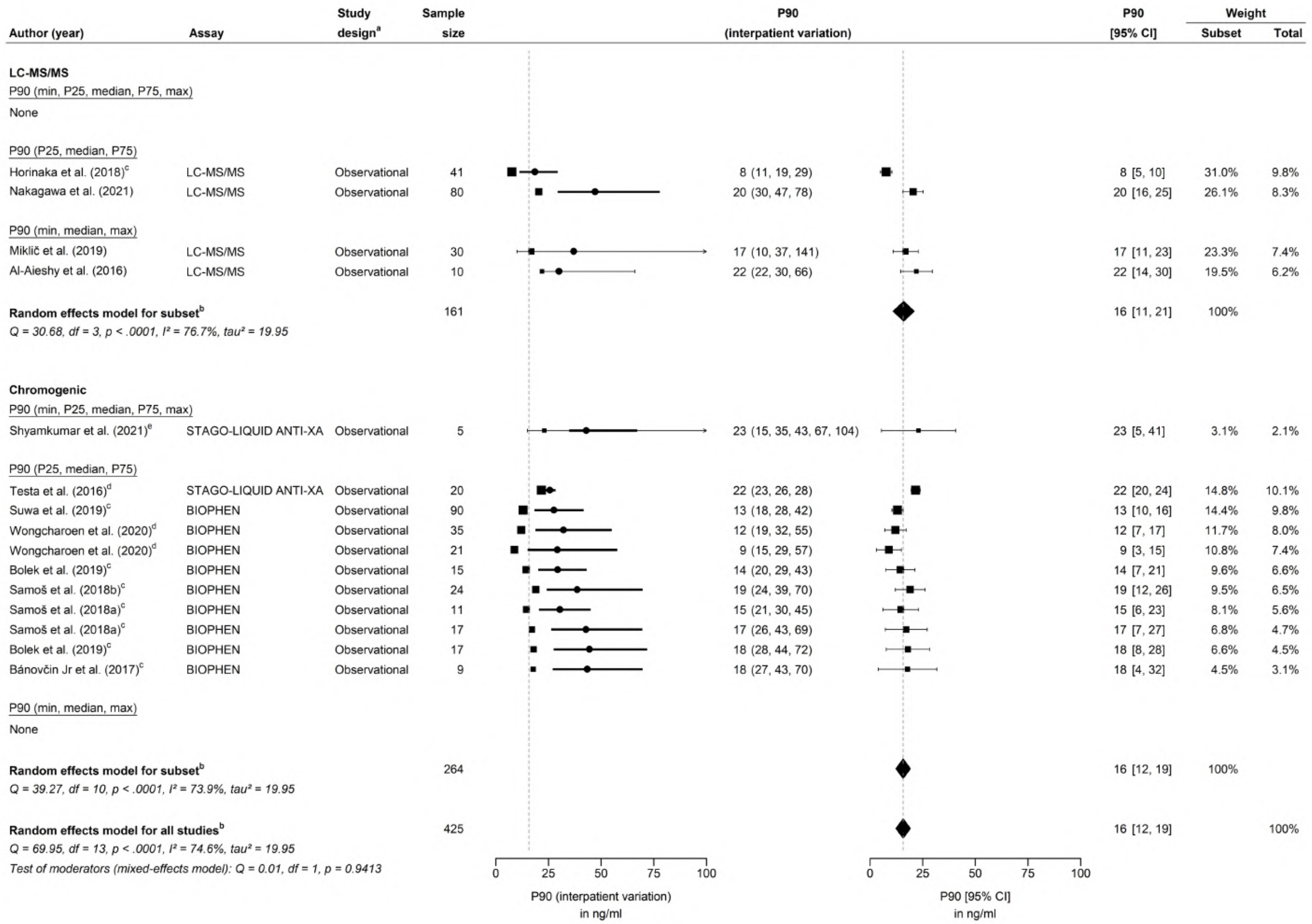

J. Rivaroxaban 15 mg once daily

[a] All analyses of interest were cross-sectional; [b] Random effects model using the quantile-estimation method;[2-5] [c] Simulated values were used because only the mean and standard deviation were available; [d] Simulated values were used because available parameters could not readily be included in the QE-method; [e] Percentiles were calculated directly from the original dataset if they were published by the authors of the current review.[6-8]

K. Rivaroxaban 20 mg once daily

We did not perform a sensitivity analysis because fewer than ten studies were available (see **Supporting Information File 2**).[1] Two studies used liquid chromatography–mass spectrometry/mass spectrometry to determine drug levels and six used a chromogenic assay.

Figure S11. Sensitivity analysis 3 (by laboratory method): Estimating the pooled 10th percentile of peak levels of each direct oral anticoagulant stratified by administered dose using the modified QE-method

The forest plots below illustrate the results of our analyses to estimate the 10th percentile of peak levels of each DOAC type, stratified by dosing regimen and by laboratory method (i.e., liquid chromatography–mass spectrometry/mass spectrometry vs. chromogenic assays). The squares represent the 10th percentile values, the circles the median values, the solid bold lines the 25th to 75th percentile range, and the whiskers either the minimum to maximum value interval (left side of the plot) or the 95% of the confidence interval of the percentile value of interest (right side of the plot).

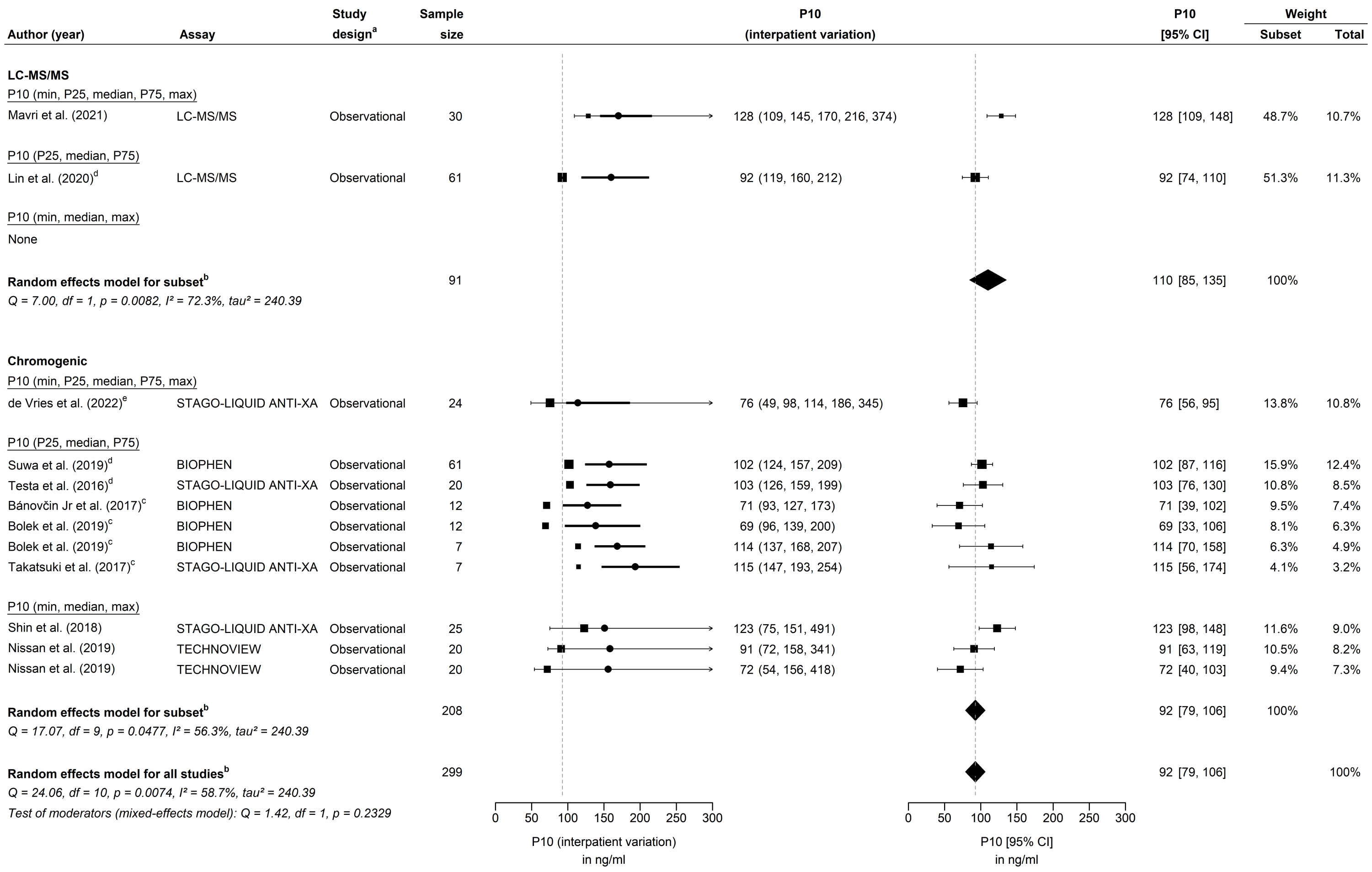

A. Apixaban 2.5 mg twice daily

[a] All analyses of interest were cross-sectional; [b] Random effects model using the quantile-estimation method;[2-5] [c] Simulated values were used because only the mean and standard deviation were available; [d] Simulated values were used because available parameters could not readily be included in the QE-method; [e] Percentiles were calculated directly from the original dataset if they were published by the authors of the current review.[6-8]

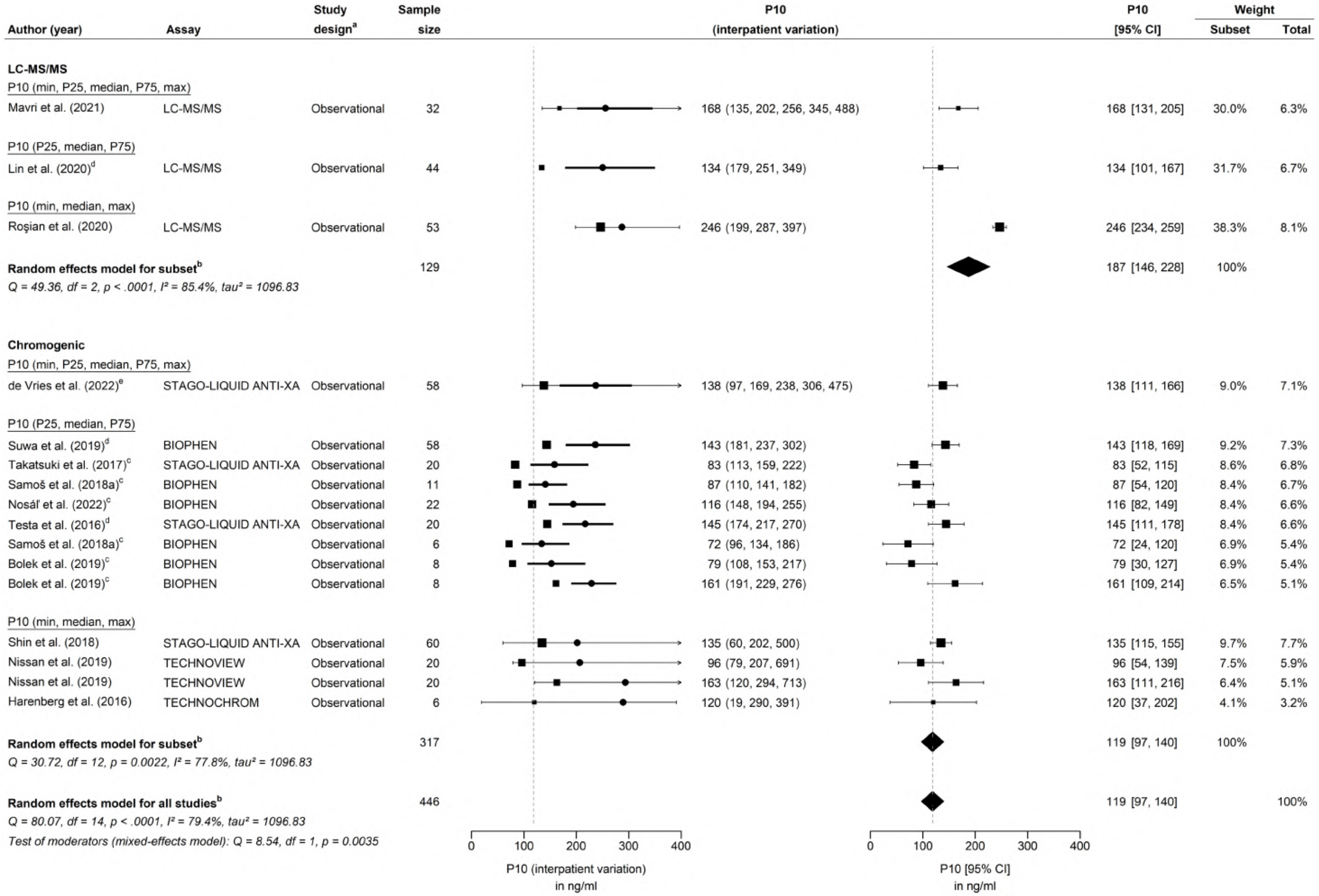

B. Apixaban 5 mg twice daily

[a] All analyses of interest were cross-sectional; [b] Random effects model using the quantile-estimation method;[2-5] [c] Simulated values were used because only the mean and standard deviation were available; [d] Simulated values were used because available parameters could not readily be included in the QE-method; [e] Percentiles were calculated directly from the original dataset if they were published by the authors of the current review.[6-8]

C. Dabigatran 75 mg twice daily

Performing a sensitivity analysis was not possible because only a single study was available.

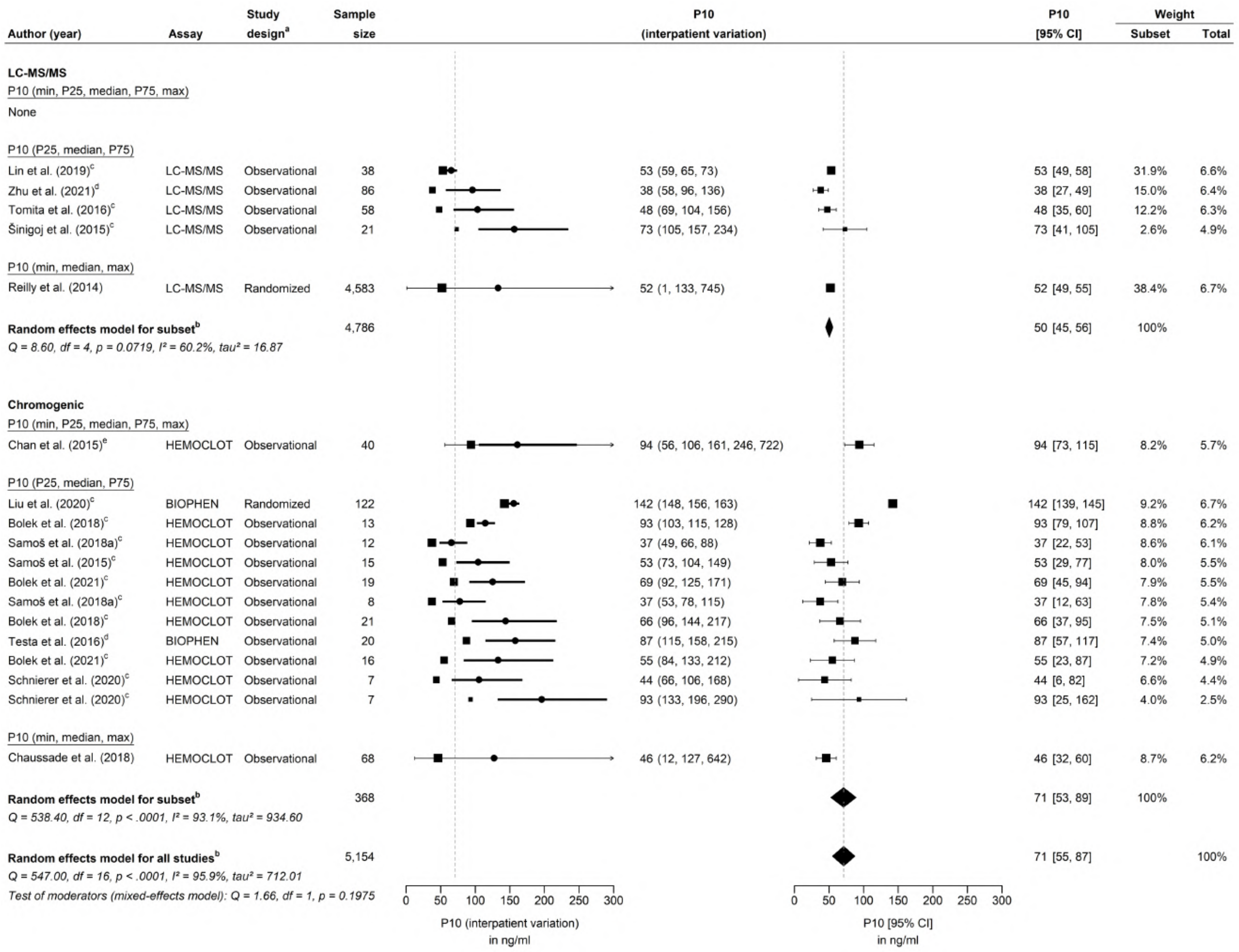

D. Dabigatran 110 mg twice daily

[a] All analyses of interest were cross-sectional; [b] Random effects model using the quantile-estimation method;[2-5] [c] Simulated values were used because only the mean and standard deviation were available; [d] Simulated values were used because available parameters could not readily be included in the QE-method; [e] Percentiles were calculated directly from the original dataset if they were published by the authors of the current review.[6-8]

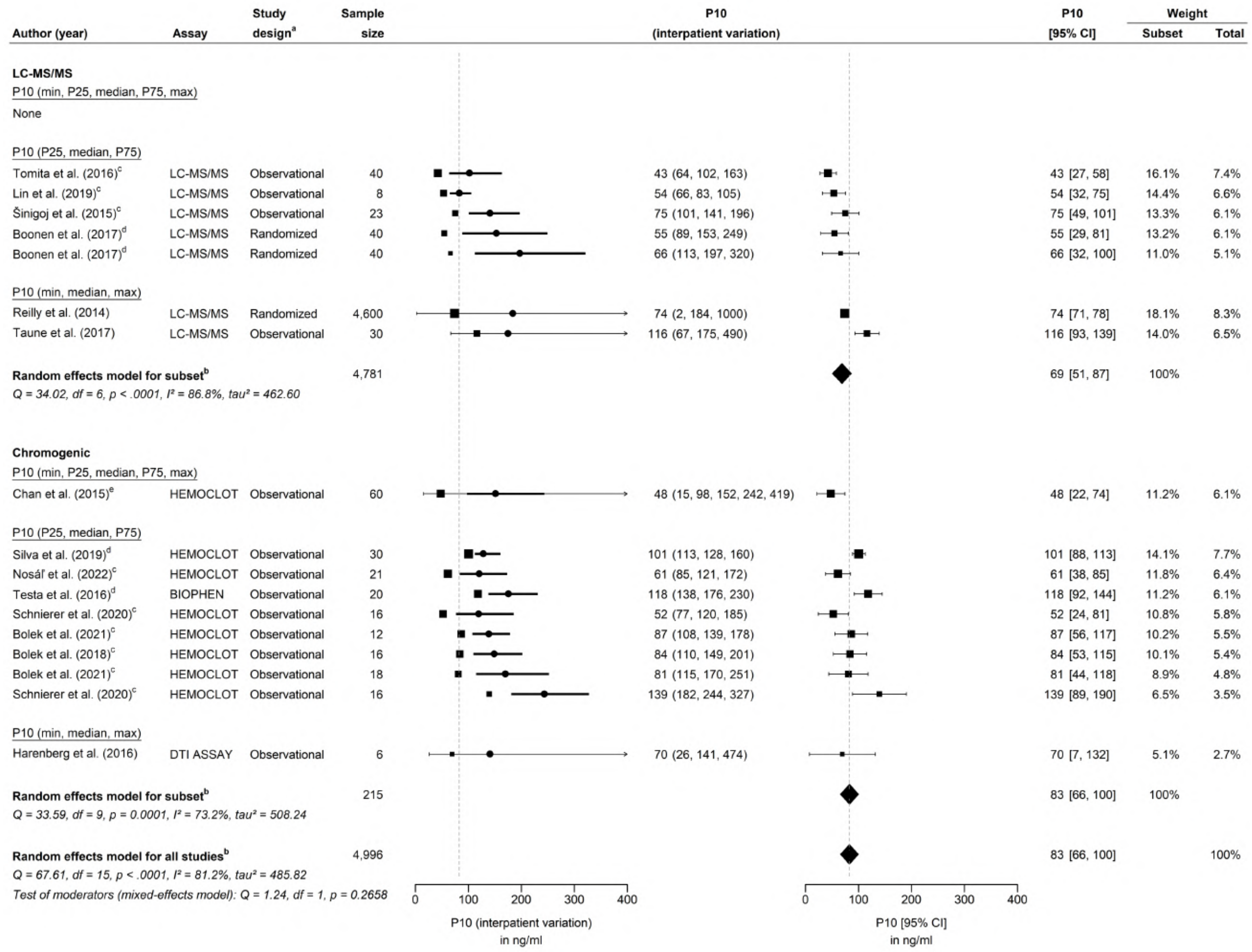

E. Dabigatran 150 mg twice daily

[a] All analyses of interest were cross-sectional; [b] Random effects model using the quantile-estimation method;[2-5] [c] Simulated values were used because only the mean and standard deviation were available; [d] Simulated values were used because available parameters could not readily be included in the QE-method; [e] Percentiles were calculated directly from the original dataset if they were published by the authors of the current review.[6-8]

F. Edoxaban 15 mg once daily

Performing a sensitivity analysis was not possible because only a single study was available.

G. Edoxaban 30 mg once daily

We did not perform a sensitivity analysis because fewer than ten studies were available (see **Supporting Information File 2**).[1] One study used liquid chromatography–mass spectrometry/mass spectrometry to determine drug levels and one used a chromogenic assay.

H. Edoxaban 60 mg once daily

We did not perform a sensitivity analysis because fewer than ten studies were available (see **Supporting Information File 2**).[1] One study used liquid chromatography–mass spectrometry/mass spectrometry to determine drug levels and one used a chromogenic assay.

I. Rivaroxaban 10 mg once daily

We did not perform a sensitivity analysis because fewer than ten studies were available (see **Supporting Information File 2**).[1] One study used liquid chromatography–mass spectrometry/mass spectrometry to determine drug levels and three used a chromogenic assay.

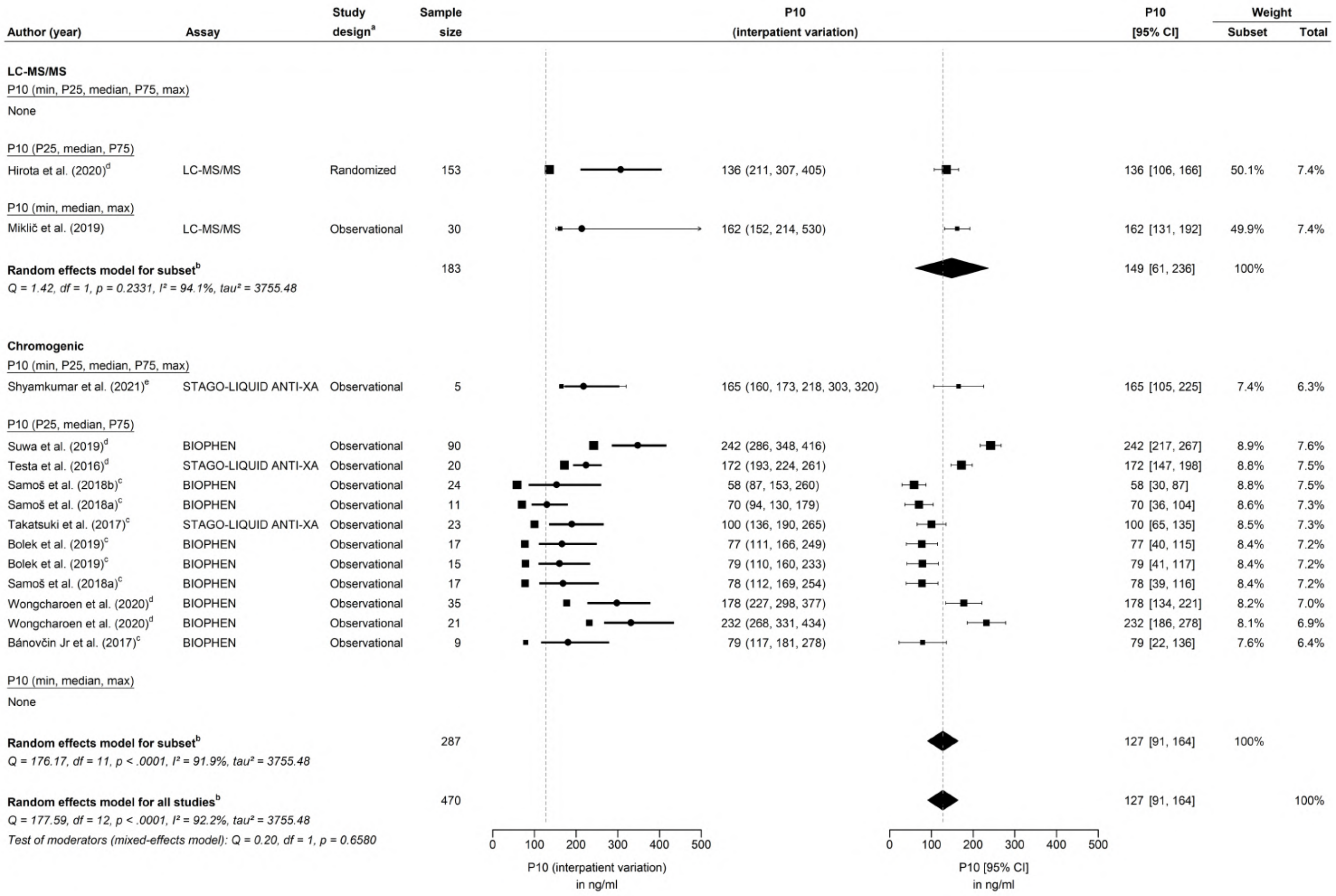

J. Rivaroxaban 15 mg once daily

[a] All analyses of interest were cross-sectional; [b] Random effects model using the quantile-estimation method;[2-5] [c] Simulated values were used because only the mean and standard deviation were available; [d] Simulated values were used because available parameters could not readily be included in the QE-method; [e] Percentiles were calculated directly from the original dataset if they were published by the authors of the current review.[6-8]

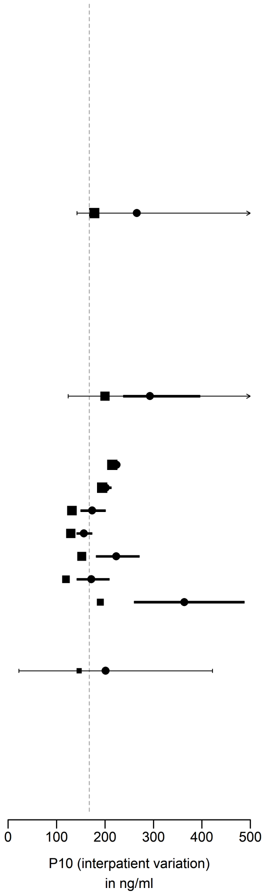

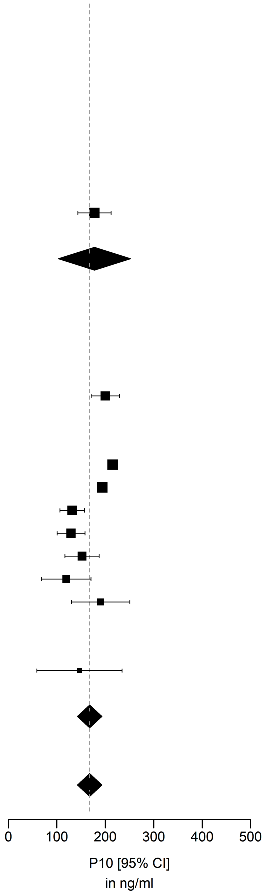

Author (year)	Assay	Study design[a]	Sample size	P10 (interpatient variation)	P10 [95% CI]	Weight Subset	Weight Total
**LC-MS/MS**							
P10 (min, P25, median, P75, max)							
None							
P10 (P25, median, P75)							
None							
P10 (min, median, max)							
Miklič et al. (2019)	LC-MS/MS	Observational	30	178 (142, 266, 552)	178 [143, 212]	100.0%	10.3%
**Random effects model for subset[b]**			30		178 [103, 253]	100%	
Q = 0.00, df = 0, p = 1.0000, I² = 0.0%, tau² = 1153.39							
**Chromogenic**							
P10 (min, P25, median, P75, max)							
Shyamkumar et al. (2021)[e]	STAGO-LIQUID ANTI-XA	Observational	46	200 (124, 238, 293, 396, 722)	200 [171, 229]	12.2%	10.9%
P10 (P25, median, P75)							
Silva et al. (2019)[d]	BIOPHEN	Observational	100	215 (219, 224, 230)	215 [213, 217]	14.5%	13.0%
Silva et al. (2017)[d]	BIOPHEN	Observational	100	194 (198, 202, 213)	194 [192, 197]	14.5%	13.0%
Nosáľ et al. (2022)[c]	BIOPHEN	Observational	14	132 (150, 174, 201)	132 [106, 157]	12.7%	11.4%
Bolek et al. (2019)[c]	BIOPHEN	Observational	5	129 (142, 157, 173)	129 [101, 158]	12.3%	11.0%
Testa et al. (2016)[d]	STAGO-LIQUID ANTI-XA	Observational	20	152 (182, 223, 271)	152 [117, 187]	11.3%	10.2%
Bolek et al. (2019)[c]	BIOPHEN	Observational	5	120 (142, 172, 208)	120 [69, 170]	9.2%	8.2%
Wongcharoen et al. (2020)[d]	BIOPHEN	Observational	35	190 (260, 364, 487)	190 [130, 251]	8.0%	7.2%
P10 (min, median, max)							
Harenberg et al. (2016)	HEMOSIL	Observational	6	147 (22, 201, 422)	147 [59, 235]	5.3%	4.8%
**Random effects model for subset[b]**			331		168 [142, 193]	100%	
Q = 240.43, df = 8, p < .0001, I² = 99.1%, tau² = 1153.39							
**Random effects model for all studies[b]**			361		168 [142, 193]		100%
Q = 240.43, df = 8, p < .0001, I² = 99.1%, tau² = 1153.39							
Test of moderators (mixed-effects model): Q = 0.06, df = 1, p = 0.8034							

K. Rivaroxaban 20 mg once daily

[a] All analyses of interest were cross-sectional; [b] Random effects model using the quantile-estimation method;[2-5] [c] Simulated values were used because only the mean and standard deviation were available; [d] Simulated values were used because available parameters could not readily be included in the QE-method; [e] Percentiles were calculated directly from the original dataset if they were published by the authors of the current review.[6-8]

Figure S12. Sensitivity analysis 3 (by laboratory method): Estimating the pooled 90th percentile of peak levels of each direct oral anticoagulant stratified by administered dose using the modified QE-method

The forest plots below illustrate the results of our analyses to estimate the 90th percentile of peak levels of each DOAC type, stratified by dosing regimen and by laboratory method (i.e., liquid chromatography–mass spectrometry/mass spectrometry vs. chromogenic assays). The squares represent the 90th percentile values, the circles the median values, the solid bold lines the 25th to 75th percentile range, and the whiskers either the minimum to maximum value interval (left side of the plot) or the 95% of the confidence interval of the percentile value of interest (right side of the plot).

Author (year)	Assay	Study design[a]	Sample size	P90 (interpatient variation)	P90 [95% CI]	Weight Subset	Weight Total
**LC-MS/MS**							
P90 (min, P25, median, P75, max)							
Mavri et al. (2021)	LC-MS/MS	Observational	30	265 (109, 145, 170, 216, 374)	265 [220, 311]	46.1%	15.8%
P90 (P25, median, P75)							
Lin et al. (2020)[d]	LC-MS/MS	Observational	61	267 (119, 160, 212)	267 [225, 309]	53.9%	18.4%
P90 (min, median, max)							
None							
**Random effects model for subset[b]**			91		266 [235, 297]	100%	
Q = 0.00, df = 1, p = 0.9600, I² = 0.0%, tau² = 0.00							
**Chromogenic**							
P90 (min, P25, median, P75, max)							
de Vries et al. (2022)[e]	STAGO-LIQUID ANTI-XA	Observational	24	228 (49, 98, 114, 186, 345)	228 [161, 295]	11.1%	7.3%
P90 (P25, median, P75)							
Suwa et al. (2019)[d]	BIOPHEN	Observational	61	273 (124, 157, 209)	273 [221, 324]	18.6%	12.3%
Testa et al. (2016)[d]	STAGO-LIQUID ANTI-XA	Observational	20	239 (126, 159, 199)	239 [186, 292]	17.7%	11.6%
Bolek et al. (2019)[c]	BIOPHEN	Observational	7	248 (137, 168, 207)	248 [153, 344]	5.4%	3.6%
Bánovčin Jr et al. (2017)[c]	BIOPHEN	Observational	12	229 (93, 127, 173)	229 [128, 330]	4.8%	3.2%
Bolek et al. (2019)[c]	BIOPHEN	Observational	12	277 (96, 139, 200)	277 [132, 423]	2.3%	1.5%
Takatsuki et al. (2017)[c]	STAGO-LIQUID ANTI-XA	Observational	7	325 (147, 193, 254)	325 [158, 492]	1.8%	1.2%
P90 (min, median, max)							
Shin et al. (2018)	STAGO-LIQUID ANTI-XA	Observational	25	210 (75, 151, 491)	210 [168, 251]	28.7%	18.9%
Nissan et al. (2019)	TECHNOVIEW	Observational	20	273 (72, 158, 341)	273 [189, 358]	7.0%	4.6%
Nissan et al. (2019)	TECHNOVIEW	Observational	20	327 (54, 156, 418)	327 [186, 469]	2.5%	1.6%
**Random effects model for subset[b]**			208		243 [220, 265]	100%	
Q = 7.09, df = 9, p = 0.6281, I² = 0.0%, tau² = 0.00							
**Random effects model for all studies[b]**			299		243 [220, 265]		100%
Q = 7.09, df = 10, p = 0.7170, I² = 0.0%, tau² = 0.00							
Test of moderators (mixed-effects model): Q = 1.48, df = 1, p = 0.2240							

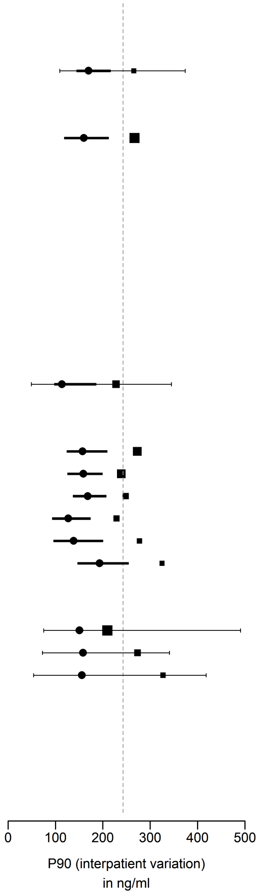

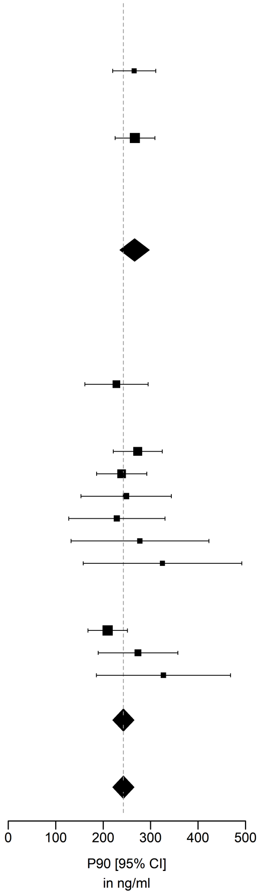

A. Apixaban 2.5 mg twice daily

[a] All analyses of interest were cross-sectional; [b] Random effects model using the quantile-estimation method;[2-5] [c] Simulated values were used because only the mean and standard deviation were available; [d] Simulated values were used because available parameters could not readily be included in the QE-method; [e] Percentiles were calculated directly from the original dataset if they were published by the authors of the current review.[6-8]

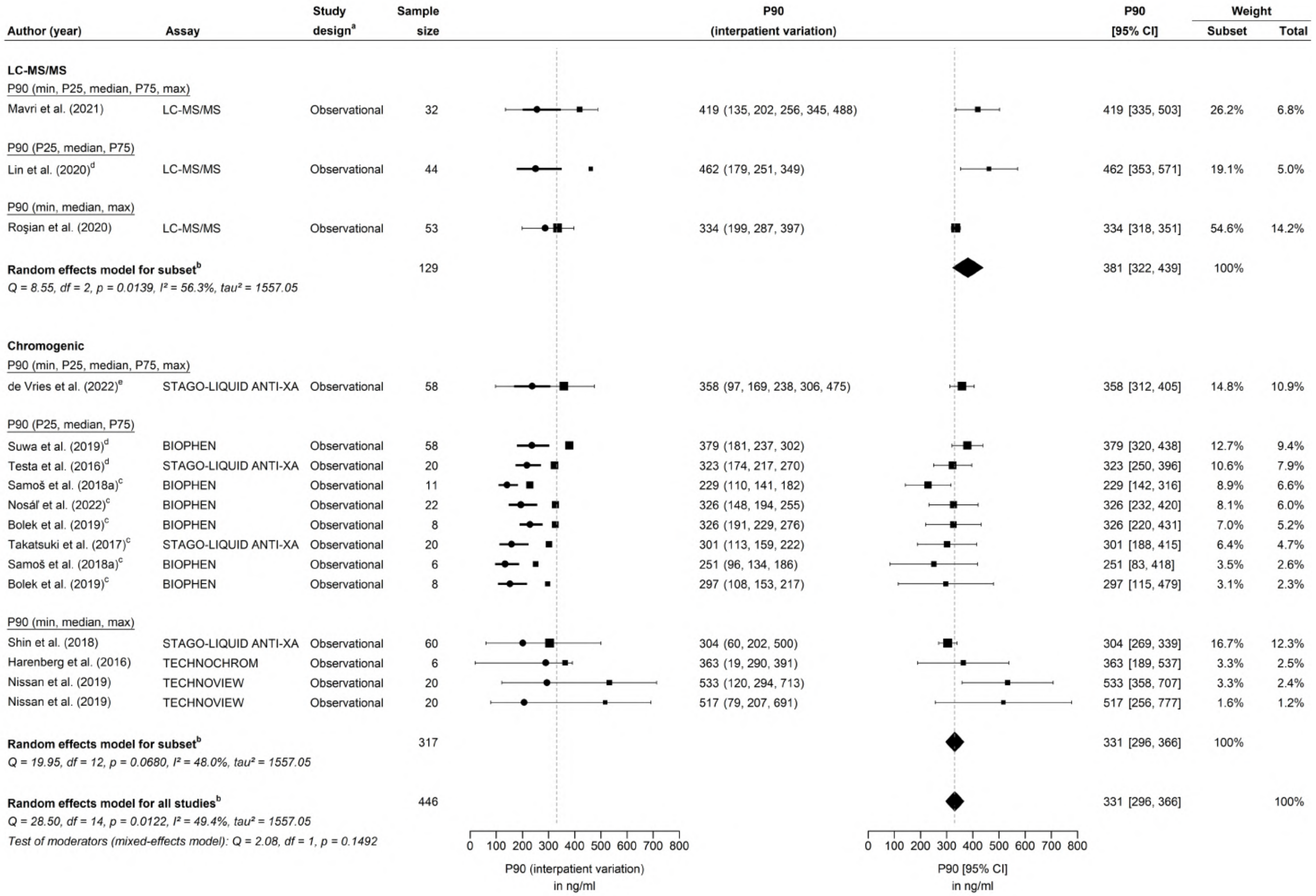

B. Apixaban 5 mg twice daily

[a] All analyses of interest were cross-sectional; [b] Random effects model using the quantile-estimation method;[2-5] [c] Simulated values were used because only the mean and standard deviation were available; [d] Simulated values were used because available parameters could not readily be included in the QE-method; [e] Percentiles were calculated directly from the original dataset if they were published by the authors of the current review.[6-8]

C. Dabigatran 75 mg twice daily

Performing a sensitivity analysis was not possible because only a single study was available.

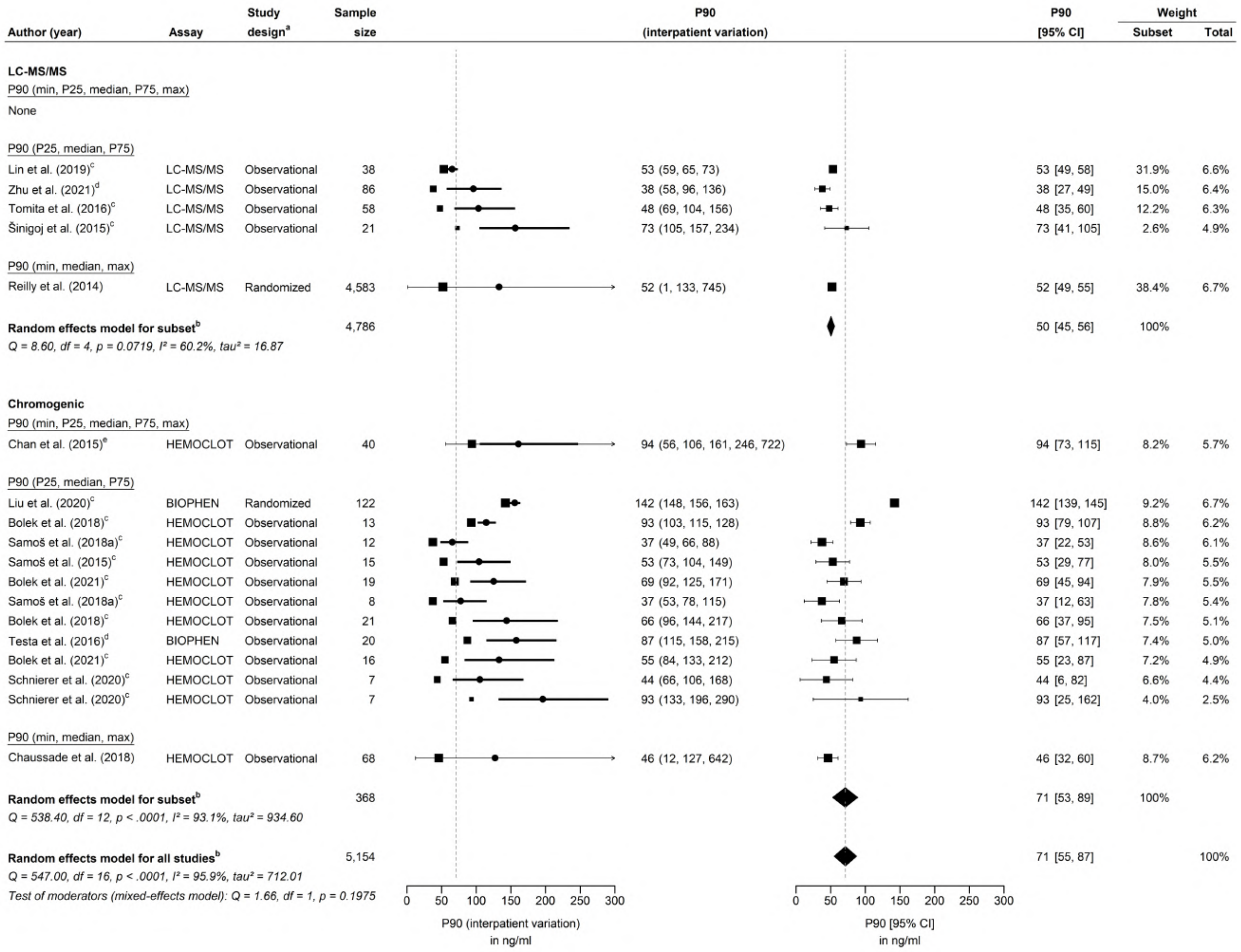

D. Dabigatran 110 mg twice daily

[a] All analyses of interest were cross-sectional; [b] Random effects model using the quantile-estimation method;[2-5] [c] Simulated values were used because only the mean and standard deviation were available; [d] Simulated values were used because available parameters could not readily be included in the QE-method; [e] Percentiles were calculated directly from the original dataset if they were published by the authors of the current review.[6-8]

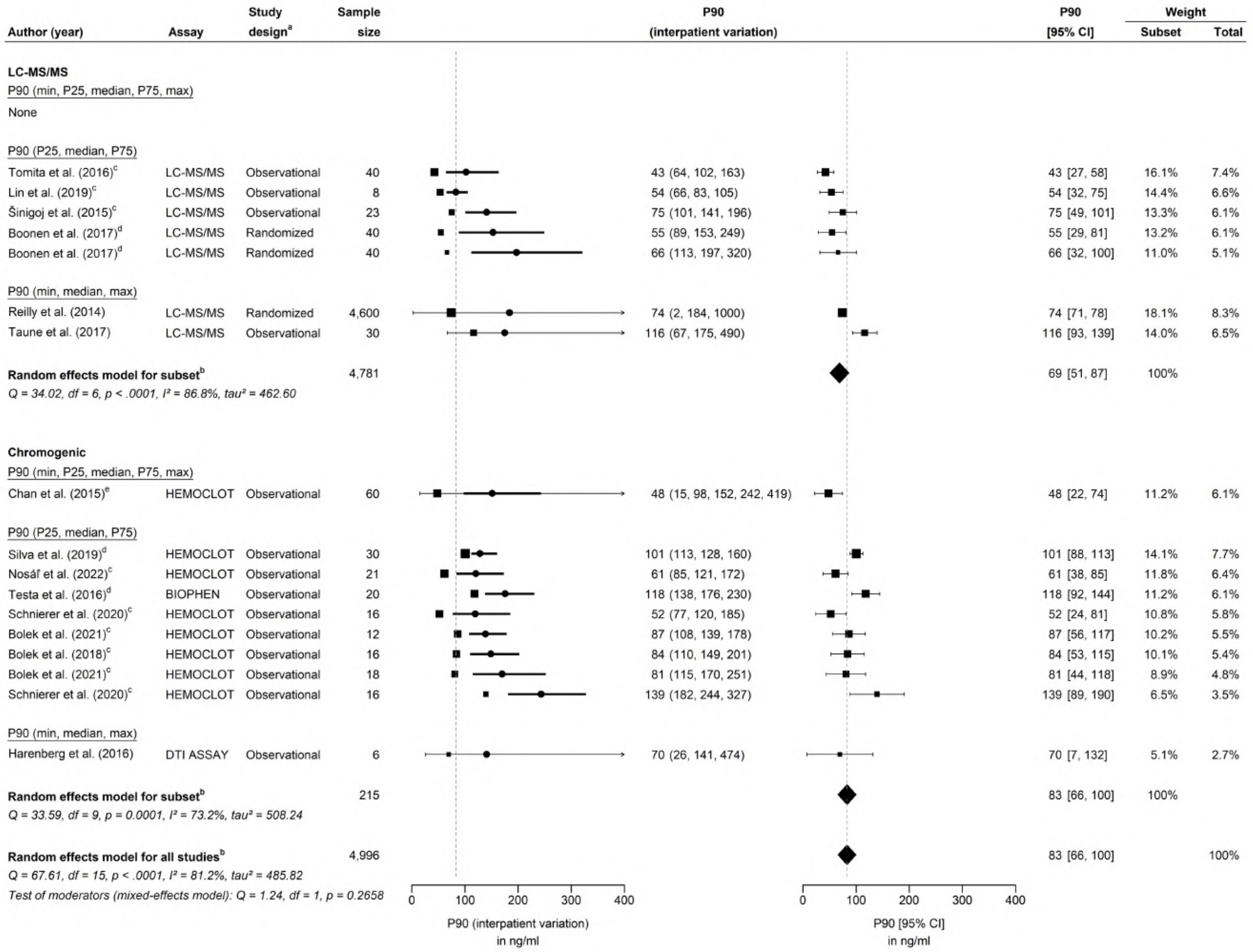

E. Dabigatran 150 mg twice daily

[a] All analyses of interest were cross-sectional; [b] Random effects model using the quantile-estimation method;[2-5] [c] Simulated values were used because only the mean and standard deviation were available; [d] Simulated values were used because available parameters could not readily be included in the QE-method; [e] Percentiles were calculated directly from the original dataset if they were published by the authors of the current review.[6-8]

F. Edoxaban 15 mg once daily

Performing a sensitivity analysis was not possible because only a single study was available.

G. Edoxaban 30 mg once daily

We did not perform a sensitivity analysis because fewer than ten studies were available (see **Supporting Information File 2**).[1] One study used liquid chromatography–mass spectrometry/mass spectrometry to determine drug levels and one used a chromogenic assay.

H. Edoxaban 60 mg once daily

We did not perform a sensitivity analysis because fewer than ten studies were available (see **Supporting Information File 2**).[1] One study used liquid chromatography–mass spectrometry/mass spectrometry to determine drug levels and one used a chromogenic assay.

I. Rivaroxaban 10 mg once daily

We did not perform a sensitivity analysis because fewer than ten studies were available (see **Supporting Information File 2**).[1] One study used liquid chromatography–mass spectrometry/mass spectrometry to determine drug levels and three used a chromogenic assay.

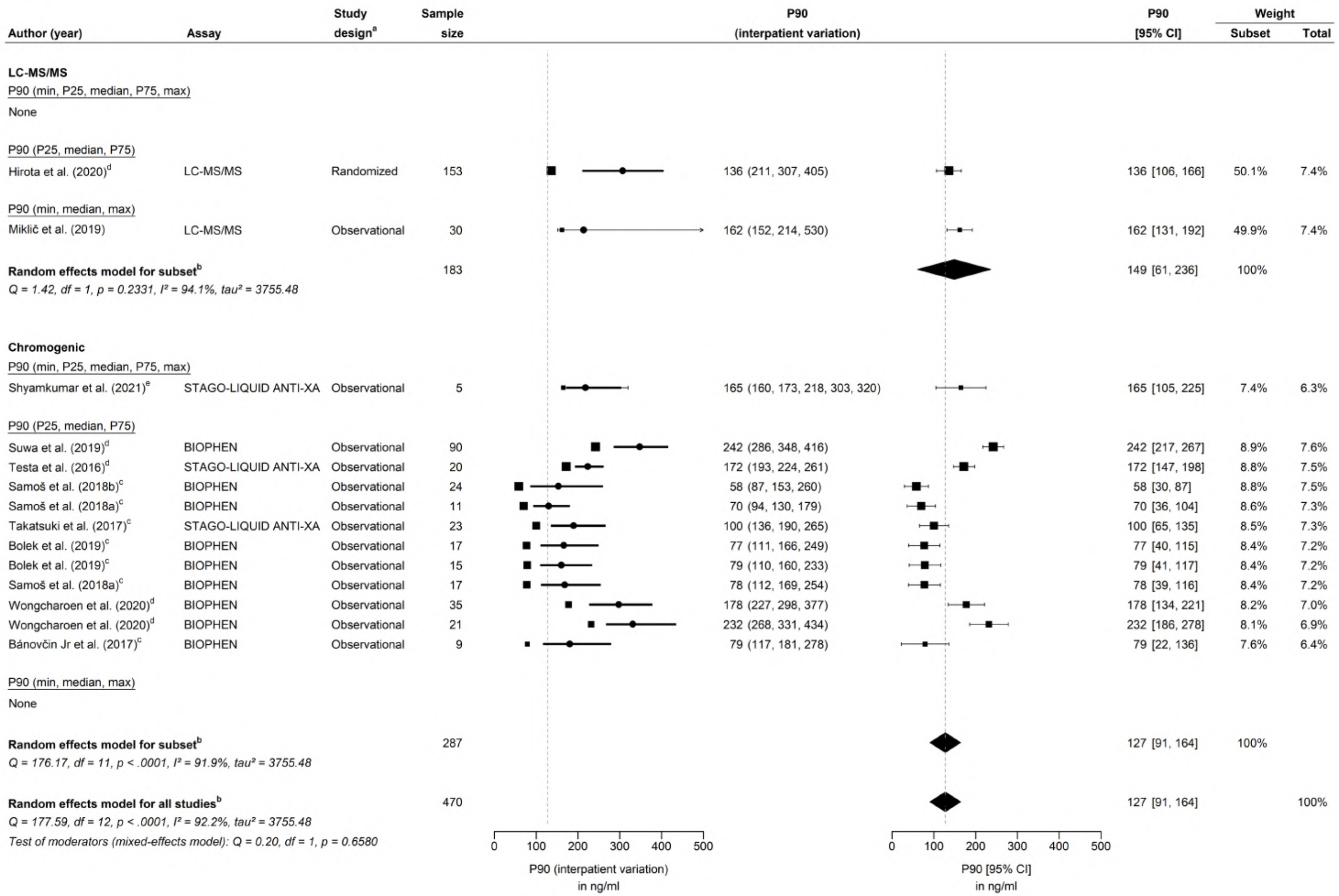

J. Rivaroxaban 15 mg once daily

[a] All analyses of interest were cross-sectional; [b] Random effects model using the quantile-estimation method;[2-5] [c] Simulated values were used because only the mean and standard deviation were available; [d] Simulated values were used because available parameters could not readily be included in the QE-method; [e] Percentiles were calculated directly from the original dataset if they were published by the authors of the current review.[6-8]

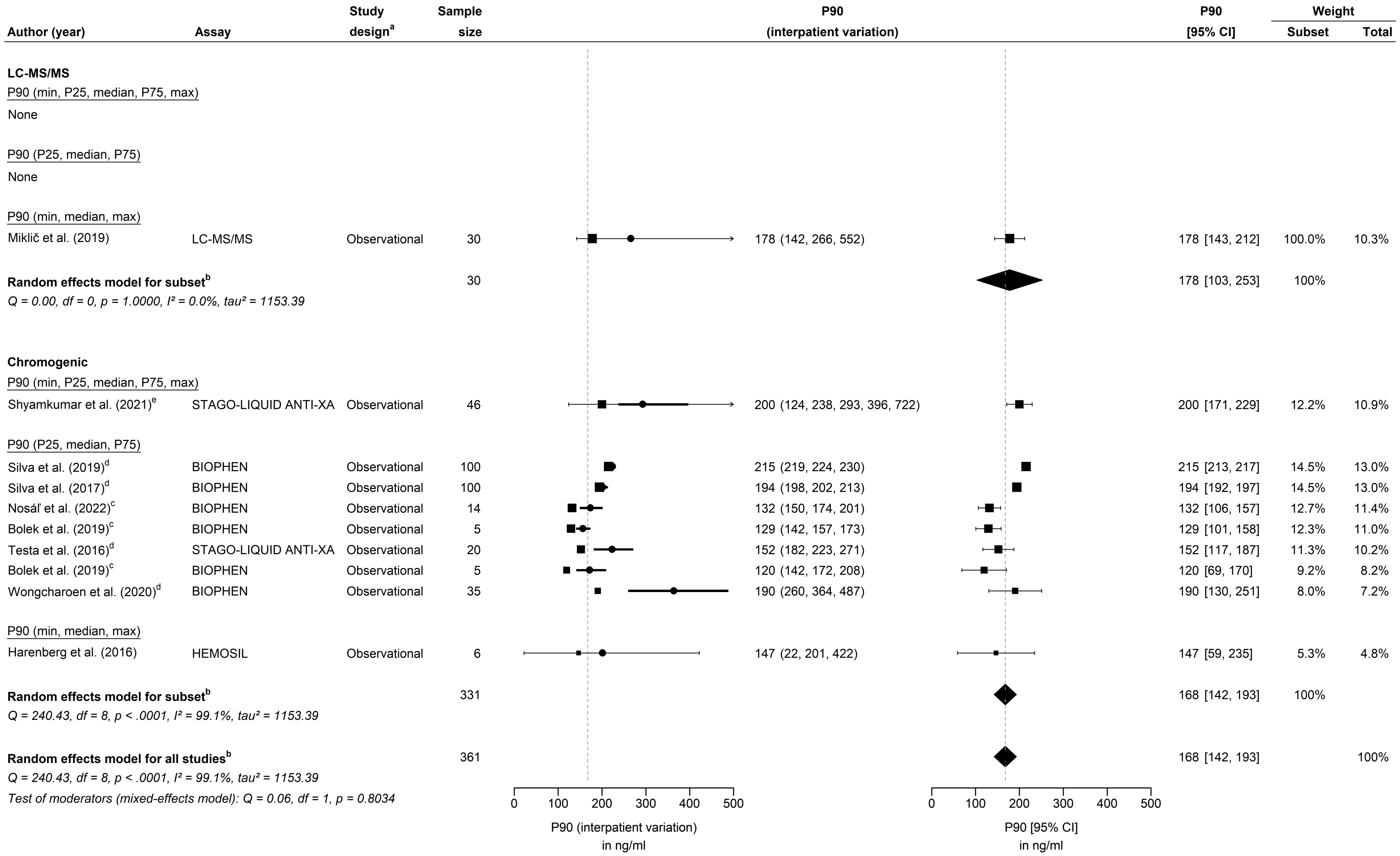

K. Rivaroxaban 20 mg once daily

[a] All analyses of interest were cross-sectional; [b] Random effects model using the quantile-estimation method;[2-5] [c] Simulated values were used because only the mean and standard deviation were available; [d] Simulated values were used because available parameters could not readily be included in the QE-method; [e] Percentiles were calculated directly from the original dataset if they were published by the authors of the current review.[6-8]

References

1. Harrer M, Cuijpers P, Furukawa TA, Ebert DD. Doing Meta-Analysis With R: A Hands-On Guide. 1st ed. Boca Raton, FL and London: Chapman & Hall/CRC Press; 2021
2. McGrath S, Sohn H, Steele R, Benedetti A. Meta-analysis of the difference of medians. *Biom J*. 2020;62(1):69-98
3. McGrath S, Zhao X, Qin ZZ, Steele R, Benedetti A. One-sample aggregate data meta-analysis of medians. *Stat Med*. 2019;38(6):969-984
4. McGrath S, Zhao X, Steele R, Thombs BD, Benedetti A. Estimating the sample mean and standard deviation from commonly reported quantiles in meta-analysis. *Stat Methods Med Res*. 2020;29(9):2520-2537
5. Borenstein M, Hedges LV, Higgins JP, Rothstein HR. A basic introduction to fixed-effect and random-effects models for meta-analysis. *Res Synth Methods*. 2010;1(2):97-111
6. de Vries TAC, Hirsh J, Bhagirath VC*, et al.* Can a Single Measurement of Apixaban Levels Identify Patients at Risk of Overexposure? A Prospective Cohort Study. *TH Open*. 2022;06(01):e10-e17
7. Shyamkumar K, Hirsh J, Bhagirath VC*, et al.* Plasma Rivaroxaban Level to Identify Patients at Risk of Drug Overexposure: Is a Single Measurement of Drug Level Reliable? *TH Open*. 2021;5(1):e84-e88
8. Bhagirath VC, Chan N, Hirsh J*, et al.* Plasma Apixaban Levels in Patients Treated Off Label With the Lower Dose. *J Am Coll Cardiol*. 2020;76(24):2906-2907
